# Structural basis of ammonium transport and coordinated regulation of AMT1;1 in *Arabidopsis thaliana*

**DOI:** 10.1038/s41467-026-78457-1

**Published:** 2026-10-10

**Authors:** Bangguo He, Ting Bai, Liyang Wang, Shuai Zhang, Wei Ying

**Affiliations:** School of Life Sciences, Anhui Agricultural University, Hefei, China

**Keywords:** Plant sciences, Electron microscopy, Permeation and transport

## Abstract

Efficient ammonium uptake is essential for plant growth and nitrogen-use efficiency, yet the structural basis of ammonium transport and regulation in plant AMTs remains unclear. Here, we determined multiple cryo-electron microscopy structures of the *Arabidopsis thaliana* ammonium transporter AMT1;1 in apo, ammonium-bound, methylammonium-bound, low-pH and phosphomimetic states. These structures reveal a conserved ammonium conduction pathway comprising an aromatic recruitment vestibule, a Phe-gate/Twin-His-containing pore, and a polar intracellular exit region. We further identified a conserved extracellular N-terminal disulfide bond required for oligomer stability and transport activity, suggesting potential redox-dependent regulation. On the cytoplasmic side, phosphorylation modulates C-terminal interactions with intracellular loops, revealing an allosteric regulatory mechanism. Comparison with the bacterial ammonium-sensing protein *Sd*-Amt1 uncovers a conserved ammonium-recognition region in *At*AMT1;1 that contributes to transport activity but lacks the signaling module and ligand-dependent conformational switching observed in *Sd*-Amt1. Together, these findings provide structural insight into ammonium transport and coordinated regulation in plant AMT transporters.

## Abstract (web summary)

He et al. determine multiple cryo-EM structures of *Arabidopsis thaliana* AMT1;1, revealing a conserved ammonium transport pathway and distinct regulatory mechanisms. An extracellular N-terminal disulfide bond contributes to oligomeric stability, while C-terminal phosphomimetic mutation remodels cytoplasmic regulatory interactions. These findings provide a structural framework for ammonium transport and regulation in plants.

## Introduction

Nitrogen is an essential macronutrient that limits plant growth, development, and agricultural productivity^1–3^. In most soils, bioavailable nitrogen is scarce and highly dynamic, forcing plants to use high-affinity uptake systems to capture reduced nitrogen species^4–6^. Ammonium (NH_4_^+^) is the main reduced nitrogen species found in soils and the preferred nitrogen source of many plants because it requires less energy for assimilation than nitrate^7–9^. The availability of ammonium differs spatially and temporally, and excessive accumulation of ammonium can cause damage to cells^10,11^. These challenges highlight the importance of accurately regulating the uptake of ammonium for plant survival and increased nitrogen use efficiency.

Ammonium transport across the plasma membrane of root epidermal and cortical cells is controlled by the Ammonium Transporter (AMT) family of high-affinity ammonium transporters conserved across land plants^12–14^. Plant AMT proteins comprise two clades, AMT1 and AMT2, belonging to the AMT/MEP/Rh family of ammonium transporter^5^. In *Arabidopsis thaliana*, AMT1;1 is the most abundantly expressed AMT1 and contributes to high-affinity ammonium uptake under nitrogen limitation^4,15,16^. Genetic studies show that loss of AMT1 function negatively affects ammonium acquisition and plant growth under low nitrogen conditions, and hence it plays a central role in the physiological function^4,15–17^. Although several transport models have been proposed, experimentally validating the precise chemical mechanism underlying ammonium translocation remains a major challenge.

The structure and biochemical analysis of the bacterial Amt and yeast MEP proteins reveal that the transporter is a homotrimer, with each subunit conducting transport through a narrow pore containing a conserved Phenylalanine-gate (Phe-gate) and twin-histidine (Twin-His) motif^18–23^. This leads to the assumption that NH_4_^+^ is initially recruited at the extracellular vestibule and subsequently deprotonated, allowing neutral NH_3_ to traverse the hydrophobic pore^18,21^. While solid-supported membrane (SSM)-based electrophysiology results indicate that the transport mediated by *Archaeoglobus fulgidus* Amt1 and Amt3 is conductive and pH-dependent, which appears to challenge the ammonia transport model^24^. Previous electrophysiological and functional studies indicate that plant AMT transporters may use multiple transport mechanisms, including electrogenic NH_4_^+^ uniport, NH_3_/H^+^ symport, electroneutral NH_3_ transport, and NH_4_^+^/H^+^ cotransport^13,25^. Several AMT1 members, such as *Le*AMT1;1, *At*AMT1;1, and *Os*AMT1;1, exhibit strong electrogenic ammonium currents consistent with direct NH_4_^+^ uniport^26–29^. In contrast, *Ta*AMT1;1 has been proposed to function as an NH_3_/H^+^ symporter based on its acid-stimulated electrogenic transport activity^30^, whereas *Pv*AMT1;1 appears to mediate NH_4_^+^/H^+^ cotransport as supported by reversal potential analysis and ammonium-induced cytoplasmic acidification^31^. Meanwhile, *At*AMT2 and related AMT2-type transporters show non-electrogenic transport behavior consistent with electroneutral NH_3_ transport^32^. Overall, these findings indicate that plant ammonium transport uses mechanistically diverse but evolutionarily related strategies, while the molecular basis of the complete pathway for cargo recognition, deprotonation, and coupling regulation remains unresolved.

Several studies have shown that the function of plant ammonium transporters (AMTs) is controlled by a precise and dynamic phosphorylation network^33–37^. In *Arabidopsis thaliana*, AMT1;1 has a unique cytosolic C-terminal regulatory domain (CRD), which is not present in bacteria and is responsible for regulating the transporter^38,39^. Phosphorylation of its Ser450 boosts transport activity^36^. Meanwhile, the phosphorylation of the conserved threonine residue (Thr460) in the CRD is a key switch that inactivates transport^33–35^. This specific dual regulatory system allows AMT1;1 to promptly react to ammonium levels in the rhizosphere and the plant’s nutritional condition, playing a vital role in sustaining plant nitrogen utilization efficiency^33–35,39^. Despite the recognition of its functional importance, the structural basis of CRD-dependent regulation and its coupling to the transport cycle have remained elusive^39^.

Beyond their established roles as ammonium transporters, AMT family proteins have also been proposed to function as transceptors, integrating nutrient transport with signal perception^40–42^. Recent studies of bacterial Amt proteins identified conserved ammonium-binding sites, termed N1 and N2, where ammonium binding is associated with conformational changes and downstream signaling^42^. Whether plant AMT1 proteins, as established ammonium transporters, also couple ammonium recognition to signaling and thereby function as transceptors remains unclear. Given their conserved cargo-recognition architecture and additional regulatory domains, structural investigation of plant AMT1 proteins may provide insights into the potential relationship between ammonium transport and signaling.

In this study, we combined cryo-electron microscopy and biochemical experiments to elucidate the molecular mechanism of ammonium transport and regulation by *At*AMT1;1. Structures of wild-type *At*AMT1;1 were determined in the presence or absence of ammonium under different pH conditions, providing structural insights into ammonium conduction and regulatory mechanisms. Furthermore, the structure of the T460D mutant was determined, indicating that the phosphomimetic mutation of the CRD reshapes the inter-subunit interactions, which allosterically silences the transporter. Collectively, these results provide a conserved structure for ammonium transport in plants and coordinated regulation of AMT1;1. By describing how environmental factors and post-translational changes affect transporter architecture, we provide a molecular basis for rational design of ammonium uptake and nitrogen utilization efficiency in crops^12,13,43^.

## Results

### AtAMT1;1 mediates ammonium and methylammonium uptake

**Fig. 1:**
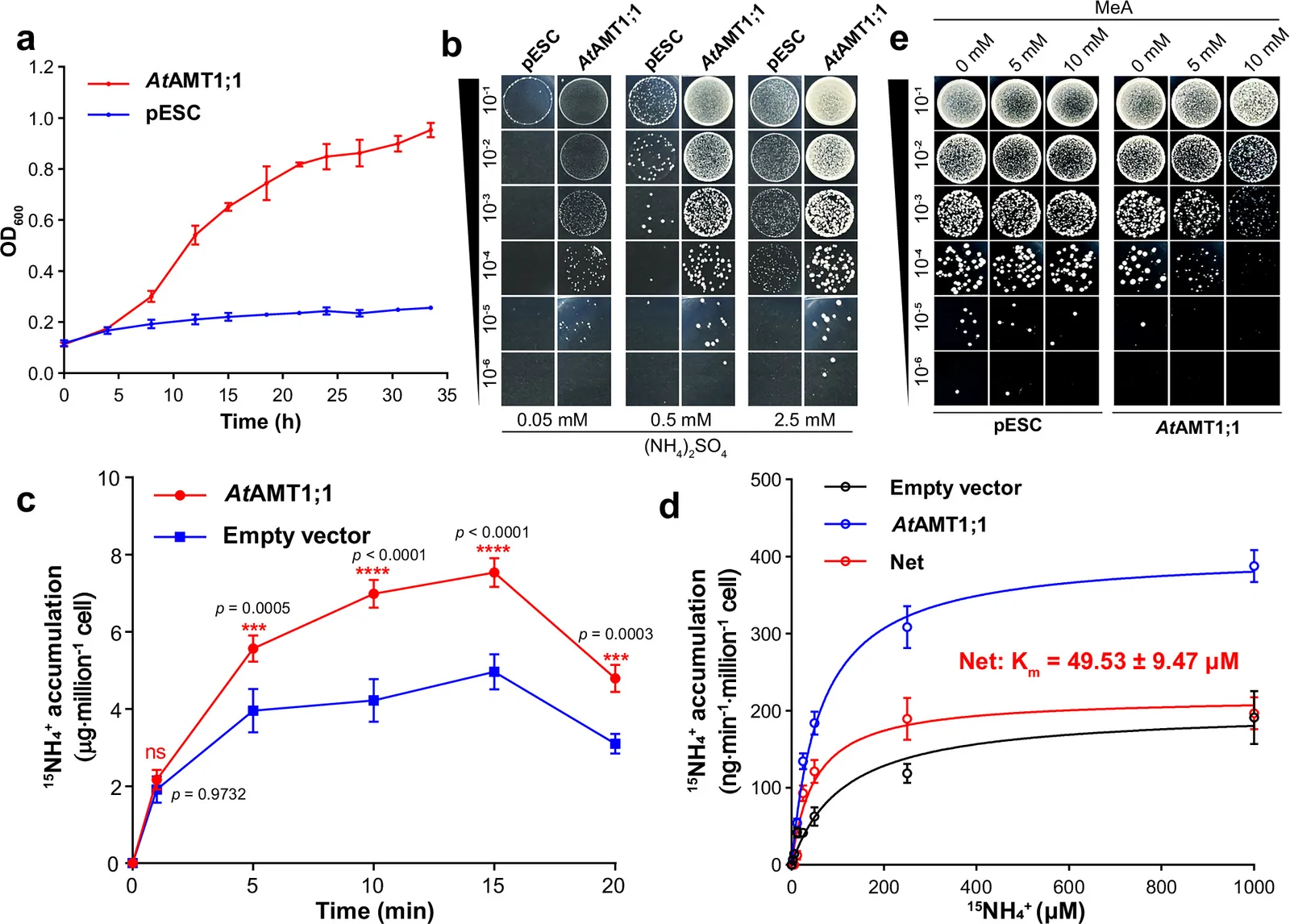
Characterization of *At*AMT1;1-mediated ammonium transport activity. **a** Growth curves measured at 600 nm (OD_600_) for the 31019b (*Δmep1 Δmep2 Δmep3 ura3*) strain expressing *At*AMT1;1 and empty vector pESC-URA control. Cultures were adjusted to an OD_600_ of 0.1 and grown at 30 °C for 33 hours in ammonium sulfate- and amino acid-deficient YNB medium (YNB-N) supplemented with 0.5 mM (NH_4_)_2_SO_4_. Data are presented as mean ± SD; *n* = 3 independent experiments. **b** Complementation of the yeast ammonium transporter mutant 31019b by *At*AMT1;1 and pESC-URA. Ten microliters of tenfold serial dilutions were spotted on YNB-N medium supplemented with 0.05, 0.5 or 2.5 mM (NH_4_)_2_SO_4_ at pH 5.5 and incubated at 30 °C for 3 days. **c** Cell-based ammonium uptake assay showing that accumulation of ^15^NH_4_^+^ is increased in cells expressing *At*AMT1;1 compared with control cells. Data are mean ± SD; *n* = 3 independent experiments. Significant differences between *At*AMT1;1 and the control group were assessed using one-way ANOVA with Sidak’s multiple comparisons test. Exact *P* values are indicated in the graph; ns, not significant. **d** Concentration-dependent ^15^NH_4_^+^ uptake by HEK293F cells. Net transport activity (red) was calculated by subtracting uptake in empty-vector cells from that in *At*AMT1;1-expressing cells and fitted using the Michaelis–Menten equation, yielding an apparent Km value of 49.53 ± 9.47 μM. All data points represent three independent measurements. Data are mean ± SD; *n* = 3 independent experiments. **e** Effects of MeA on the growth of *At*AMT1;1-expressing cells. Yeast cell suspensions were spotted on YNB-N medium (pH 5.5, containing 0.5 mM L-Arg) supplemented with 0, 5, and 10 mM MeA and incubated at 30 °C for 3 days.

To evaluate the ammonium transport function of AMT proteins, we employed a heterologous expression complementation system in the ammonium transport-deficient yeast strain 31019b (Δ*mep1*, Δ*mep2*, Δ*mep3*, *ura3*), which enables the restoration of yeast growth^11,22,39,44^. Under low ammonium conditions of 50 μM, the yeast strain expressing *At*AMT1;1 exhibited partial growth restoration compared to the empty-vector control. With increasing ammonium concentrations, both the control and *At*AMT1;1-expressing strains exhibited enhanced growth, with the latter demonstrating significantly superior growth (Fig. 1a, b). SDS-PAGE and size-exclusion chromatography have confirmed the successful expression and favorable biophysical properties of the purified *At*AMT1;1 protein in human embryonic kidney 293 F (HEK293F) cells (Supplementary Fig. 1a). Consequently, we sought to develop a transport assay utilizing HEK293F cells to directly evaluate AMT transport activity. This was achieved by quantifying the accumulation of stable isotope-labeled ^15^NH_4_^+^ in HEK293F cells through mass spectrometry^5,33–35,45^. The isotope accumulation increased over time in both the experimental group expressing *At*AMT1;1 and the empty-vector control (Fig. 1c). However, the accumulation was notably higher in the experimental group, suggesting that *At*AMT1;1 exhibits significant ammonium transport activity in HEK293F cells (Fig. 1c). The cellular accumulation rates were measured at various ammonium concentrations. The difference between the accumulation rate of the overexpressing cells and that of the empty-vector control was fitted to determine a net transport K_m_ value for *At*AMT1;1 of 49.53 ± 9.47 μM (Fig. 1d). This finding indicates that *At*AMT1;1 functions as a high-affinity transporter, which is consistent with previous measurements observed in plants and yeast^16,46,47^. AMTs are permeable to the toxic methylammonium (MeA), an NH_4_^+^ analog that inhibits cell growth and reduces plant biomass production^11,44,48^. Yeast growth was significantly more inhibited in yeast expressing *At*AMT1;1 than in the empty-vector control, due to the accumulation of this toxic analog (Fig. 1e). These results show that *At*AMT1;1 functions as a high-affinity ammonium transporter that mediates uptake of ammonium and its structural analog MeA, thereby providing a functional framework for dissecting cargo recognition and selectivity.

### The overall architecture of *At*AMT1;1

**Fig. 2:**
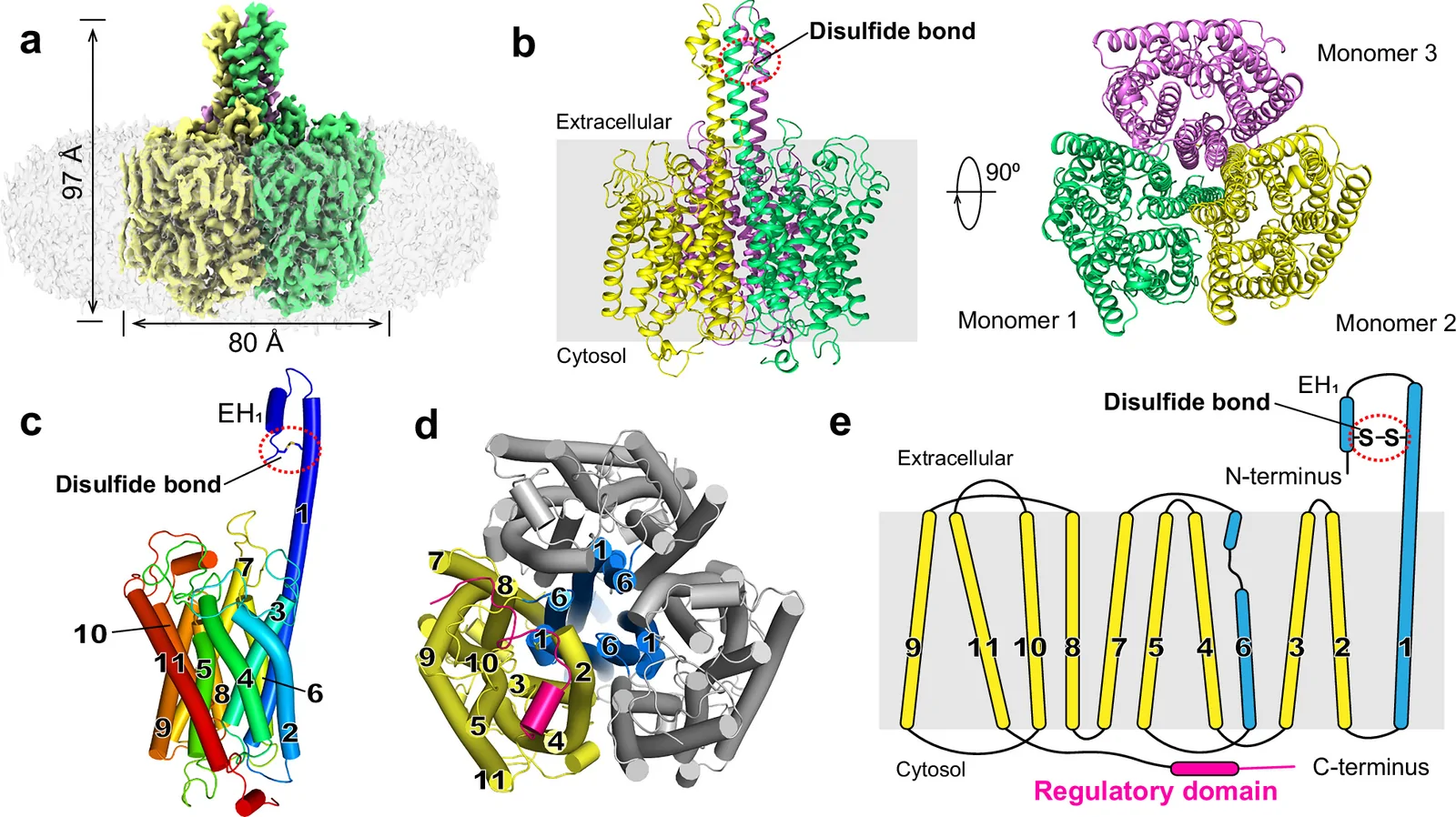
Architecture of wild-type *At*AMT1;1 in apo state. **a** Cryo-EM density map of the *At*AMT1;1 trimer showing the overall reconstruction quality and architecture. Densities corresponding to the *At*AMT1;1 protomers are colored green, purple, and yellow, respectively. The detergent density is shown in gray. **b** Atomic model of the *At*AMT1;1 trimer viewed from the side and extracellular side, highlighting the structural organization and the conserved N-terminal disulfide bond. **c** Cartoon representation of the protomer structure of wild-type *At*AMT1;1. **d** Cartoon representation of wild-type *At*AMT1;1 in the apo-state showing the top view from the cytoplasmic side. The regulatory domain of one protomer of wild-type *At*AMT1;1 is colored magenta, while the TM1 and TM6 are colored blue. **e** A topology diagram of wild-type *At*AMT1;1.

To define the structural basis of high-affinity ammonium transport in plants, we determined the cryo-EM structure of full-length *At*AMT1;1. Using cryo-electron microscopy (cryo-EM) single-particle analysis, we determined the cryo-EM map of wild-type *At*AMT1;1 at an overall resolution of 2.9 Å (Fig. 2a and Supplementary Fig. 1b–f). The EM map shows C3 symmetry, suggesting a homo-trimeric structure for *At*AMT1;1 (Fig. 2a). Utilizing the high-resolution EM density, we constructed a structural model (Fig. 2b). Each protomer comprises 11 transmembrane helices (Fig. 2c), with TM1 and TM6 playing key roles in trimer assembly (Fig. 2d). Notably, the C-terminus of *At*AMT1;1 has been shown to be involved in phosphorylation-dependent regulation^34–36,39^. We included the C-terminal segment in our model and designated it as the regulatory domain (Fig. 2d, e). The structure revealed a trimeric architecture with a conserved transmembrane core and plant-specific regulatory features.

### A plant-specific N-terminal disulfide bond stabilizes AMT1 and may function as a redox-sensitive regulatory element

**Fig. 3:**
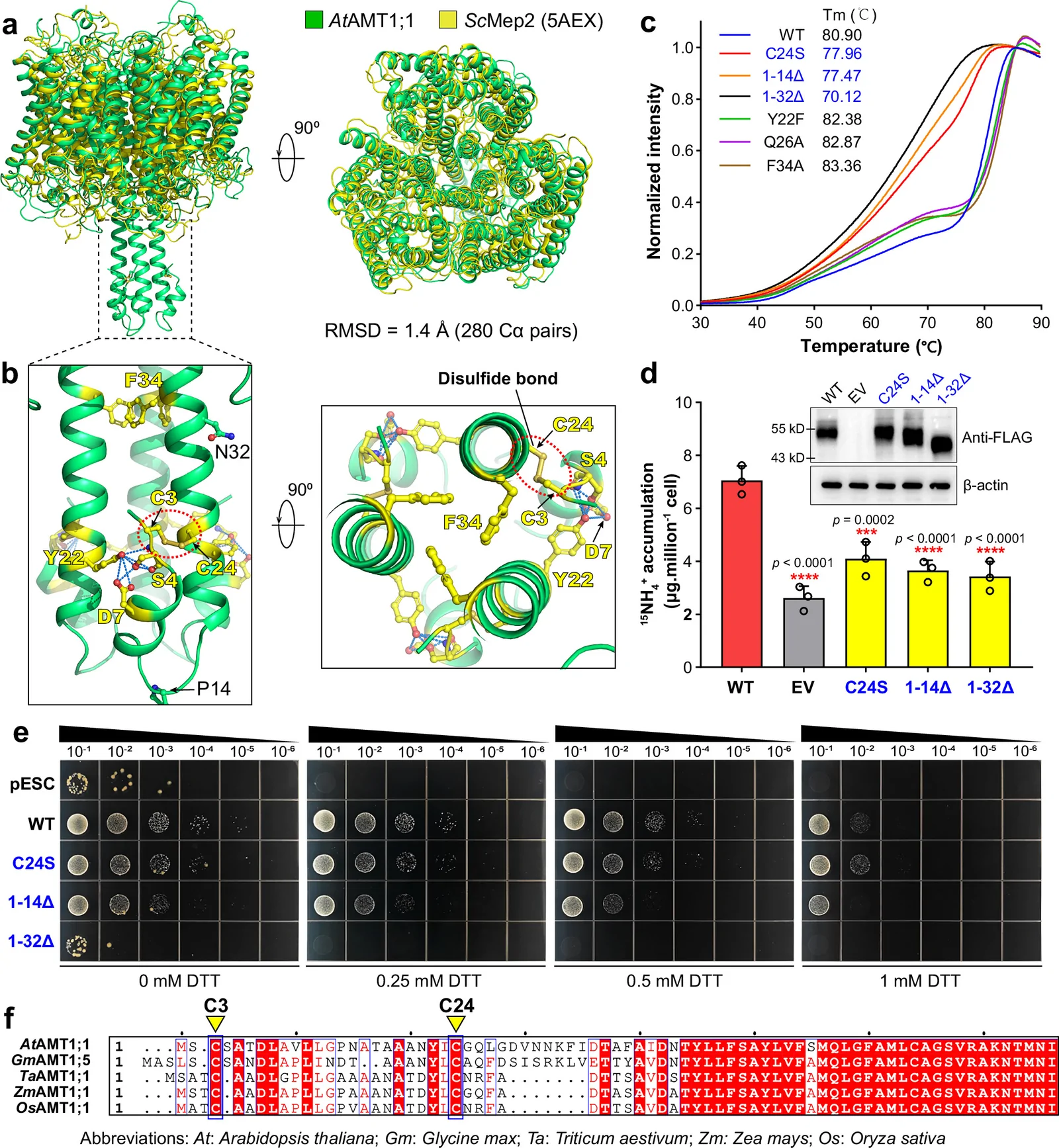
Structural features and functional analysis of the N-terminal motif. **a** Structural alignment of *At*AMT1;1 and *Saccharomyces cerevisiae* Mep2 (PDB 5AEX). The structures of *At*AMT1;1 and Mep2 are colored green and yellow, respectively. The dashed box indicates the N-terminal segment shown in (**b**). **b** Different views are presented here for the N-terminal motif of wild-type *At*AMT1;1. The side chains of the key amino acids are displayed in yellow. Conserved residues C3 and C24 form an intramolecular disulfide bond that is shown in a red dashed circle. **c** Microscale fluorescent thermal stability assay of wild-type (WT) *At*AMT1;1 and the indicated N-terminal mutants. Apparent melting temperatures (Tm) are indicated. Each data point represents the mean of four independent experiments. **d** Results from the cell-based ammonium uptake assay of *At*AMT1;1 N-terminal motif mutants. Data are means ± SD; *n* = 3 independent experiments. Significance was determined using one-way ANOVA with Dunnett’s multiple comparisons test. Exact *P* values are indicated in the graph for WT versus mutants. The expression level of these mutants was detected by whole-cell Western Blot (WB) using an anti-Flag antibody. **e** Yeast complementation assays were performed under increasing concentrations of the reducing agent DTT. Ten microliters of tenfold serial dilutions were spotted on YNB-N medium supplemented with 0.5 mM (NH_4_)_2_SO_4_ at pH 5.5 and incubated at 30 °C for 3 days. **f** Sequence alignments of the N-terminal region of AMT1;1 in various plant species. Sequences for *At*AMT1;1, *Gm*AMT1;5, *Ta*AMT1;1, *Zm*AMT1;1 and *Os*AMT1;1 are publicly available at Uniprot (https://www.uniprot.org) with the following accession codes: *At*AMT1;1: P54144, *Gm*AMT1;5: I1LBS7, *Ta*AMT1;1: Q676V6, *Zm*AMT1;1: A0A3L6G6C1 and *Os*AMT1;1: Q7XQ12. Sequences are aligned using the ClustalW method. Conserved cysteines corresponding to C3 and C24 of *At*AMT1;1 are indicated by yellow triangles.

The AMT1 protein family is widespread in plants, with *Arabidopsis thaliana* having five members and rice having three^5,49–51^. These members are highly conserved across different species, indicating conserved transport and regulatory mechanisms in plants^13,52^. While the sequence similarity between *Arabidopsis thaliana* AMT1 family proteins and AMT2 family proteins is relatively low, a notable distinction is the additional N-terminal sequence (Supplementary Fig. 2a, b and Supplementary Fig. 3). Sequence and structural comparisons of *At*AMT1;1 with homologous proteins from other species, such as yeast MEP protein, *Escherichia coli* AmtB protein, and human RHCG protein, revealed that the N-terminal motif is specific to plant AMT1 family members^18,22,53^ (Fig. 3a and Supplementary Fig. 3, 4), suggesting potential unique regulatory functions within AMT1 subfamily in plants. Previous studies have demonstrated that a conserved N-terminal cysteine of *Le*AMT1;1 plays a critical role in maintaining oligomer assembly and transport^52^. However, the structural characteristics of the N-terminal domain remain unknown, and its regulatory function in plants is still unclear.

In wild-type *At*AMT1;1, the N-terminal motif exhibits a local resolution below 3.5 Å in the EM map (Supplementary Fig. 1d), revealing clear structural details. The TM1 region spans from the cell membrane to the extracellular space, forming a distinctive ‘hook’ structure with a short helix at the N-terminus (Figs. 2c, e, 3a, b). An intramolecular disulfide bond forms between C3 and C24 in each monomer (Fig. 3b, e and Supplementary Fig. 6a, b), which are highly conserved within the plant AMT1 family (Supplementary Fig. 2a). Moreover, the extended segment of TM1 participates in trimer interactions by establishing hydrogen bonds between D7 and S4 of one monomer and Y22 of another, while the F34 residues from the three monomers engage in π-π stacking interactions, collectively promoting protein stability through these extensive interactions (Fig. 3b).

To evaluate the role of the N-terminal region, we examined the transport activity, trimer assembly, and thermal stability of various N-terminal truncated and mutant variants. SDS-PAGE and size-exclusion chromatography of the purified proteins indicate favorable biophysical properties (Supplementary Fig. 7a). Further fitting of the thermal unfolding curves revealed that truncation of the N-terminal segment (1-14Δ and 1-32Δ) or mutation of C24 (C24S) resulted in reduced thermal stability compared to wild-type and other single mutants, as determined by a microscale fluorescent thermal stability assay^54,55^ (Fig. 3c). Mass photometry determines the molecular mass of individual protein particles by measuring the interferometric light scattering signal generated when molecules transiently land on a glass surface, thereby providing a direct readout of sample homogeneity and oligomeric stability^56–58^. We employed this method to investigate the effects of conserved disulfide bonds and the N-terminal domain on oligomer stability. Under identical LMNG conditions, detergent-only controls produced a symmetric mass distribution centered at −213 ± 41 kDa and 213 ± 41 kDa, corresponding to reversible detergent micelle-associated signals (Supplementary Fig. 7b). WT *At*AMT1;1 displayed two distinct populations (Supplementary Fig. 7b). The first population showed a symmetric distribution ( − 157 ± 32 kDa and 165 ± 41 kDa) comparable to the detergent control, suggesting that this signal primarily originates from detergent micelles. In contrast, the second population ( ~ 382 ± 79 kDa) lacked a corresponding negative signal and therefore represents a stable protein-associated particle population (Supplementary Fig. 7b). This molecular mass is consistent with a detergent-stabilized *At*AMT1;1 oligomeric assembly and agrees with the trimeric architecture independently revealed by SEC and cryo-EM reconstruction (Supplementary Fig. 1). To examine the contribution of the N-terminal region to oligomer stability, we analyzed the C24S mutant and N-terminal truncation variants. Unlike WT *At*AMT1;1, these mutants exhibited broadened and heterogeneous protein-associated distributions, with reduced stable oligomeric populations (Supplementary Fig. 7b). These results indicate that the N-terminal disulfide-linked region contributes to maintaining the stable oligomeric assembly of *At*AMT1;1. Consistently, yeast complementation and isotope transport assays demonstrated that the ammonium transport capacity of the N-terminal truncated proteins and the C24S mutant was significantly lower than that of the wild-type protein and other single mutants (Fig. 3d and Supplementary Fig. 7c).

The disulfide bond is located on the extracellular face of the transporter, raising the possibility that it may contribute to regulation of transporter stability under different redox environments. Consistent with this possibility, yeast growth assays showed that treatment with the reducing agent DTT inhibited cells expressing wild-type *At*AMT1;1 more strongly than those expressing the C24S mutant (Fig. 3e). To further examine whether the N-terminal cysteine pair influences the oligomeric stability of *At*AMT1;1 under different redox conditions, we performed mass photometry using a sequential reduction–oxidation protocol (Supplementary Fig. 8a). Purified WT *At*AMT1;1 was treated with DTT to reduce cysteine residues, followed by desalting to remove residual reducing reagent before oxidation treatment (Supplementary Fig. 8a). DTT treatment substantially reduced the predominant high-molecular-weight *At*AMT1;1 oligomeric population, whereas subsequent incubation with low concentrations of H₂O₂ partially restored this population (Supplementary Fig. 8b). In contrast, the N-terminal truncation variant (1–32Δ), which lacks the conserved cysteine pair, showed no comparable redox-dependent changes (Supplementary Fig. 8c). Together, these results indicate that the conserved N-terminal cysteine pair contributes to the redox-dependent stability of the *At*AMT1;1 oligomeric assembly. Moreover, sequence alignment of AMT1 family members from diverse plant species showed that the two cysteine residues forming the N-terminal disulfide bond are highly conserved across plant AMT1 transporters (Fig. 3f). These observations suggest that the conserved disulfide-linked N-terminal region may provide a structural basis for redox-dependent modulation of AMT1 transporter stability and activity.

### Transport pathway of *At*AMT1;1

Early studies of bacterial Amt proteins proposed an NH_3_ gas-channel mechanism, whereas subsequent electrophysiological analyses revealed electrogenic, pH-dependent transport properties, indicating more complex behavior^24^. Plant AMT proteins appear to employ even more diverse transport mechanisms^13,25^. Electrophysiological studies indicate that *At*AMT1;1 may function as an NH_4_^+^ uniporter^27,31^. However, the detailed structural mechanisms underlying ammonium translocation remain unresolved. The amino acid residues involved in ammonium selectivity and transport are highly conserved among ammonium transporters of different species (Supplementary Fig. 4a–c). *At*AMT1;1 shows the highest sequence and structural similarity to *Escherichia coli* AmtB^18^, with an RMSD of only 1.2 Å in their structural alignment (Supplementary Fig. 3, 4a, b). We could not definitively ascertain whether AMT1 family proteins transport ammonium or ammonia, or identify the stage at which the interconversion between the two occurs. Therefore, we collectively denote them (ammonium and ammonia) as Am in the figures and text. To elucidate the transport pathway of plant AMT1 proteins, we resolved structures of *At*AMT1;1 under three conditions: the apo-state (pH 7.4), in the presence of 1 mM ammonium sulfate (pH 7.4), and in the presence of 10 mM MeA (pH 7.4) (Supplementary Figs. 1a, 5a–c). Notably, the EM density reconstructed with C3 symmetry was nearly identical to the EM density computed without symmetry (C1), but the C3-symmetry reconstruction exhibited higher resolution (Supplementary Fig. 5b). Therefore, subsequent discussions are based on the densities reconstructed with C3 symmetry.

Careful inspection of the cryo-EM densities revealed a consistent non-protein density adjacent to H372 within the central pore region in both the apo, the structure determined in the presence of 1 mM ammonium sulfate (pH 7.4), and low-pH states (Supplementary Fig. 11a–c, e). The position of this density is close to the cargo-associated densities previously observed near the conserved Twin-His motif in bacterial AmtB structures determined in the presence of NH_4_^+^, where this region was proposed to participate in cargo stabilization during conduction^18,19^ (Supplementary Fig. 11g, h). However, given the limited resolution and the absence of direct chemical identification, the density cannot be unambiguously assigned as an ammonium molecule and may also represent an ordered water molecule (Supplementary Fig. 11a–c, e). Notably, the imidazole NE2 atom of H372 is oriented toward this density across different *At*AMT1;1 structures (Supplementary Fig. 11a–c, e), consistent with a potential role of H372 in coordinating or stabilizing a molecule within the pore. Notably, the distance between H372 NE2 and the density varies among different conditions, with a shorter distance observed under acidic conditions (Supplementary Fig. 11a–c, e). These variations may reflect different occupancy states or coordination modes of small molecules during the transport cycle. In contrast, equivalent densities were not observed in fungal Mep structures, including *Candida albicans* Mep2, in which the pore architecture appears partially occluded by a tyrosine residue positioned near the conserved histidine pair^22^ (Supplementary Fig. 11i, l). To further examine the structural role of this region, we determined the structure of the H372F mutant (Supplementary Fig. 9a). The corresponding non-protein density near the Twin-His region was substantially reduced in the H372F mutant structure (Supplementary Fig. 11d), indicating that H372 is important for maintaining the local environment of this site. Similar densities were observed in the structure determined in the presence of 10 mM methylammonium (Supplementary Fig. 11c), suggesting that this region may remain occupied under different conditions. In contrast, equivalent densities were absent in the previously reported methylammonium-bound bacterial AmtB structure^18^ (Supplementary Fig. 11j), despite the overall conservation of the pore architecture (Supplementary Fig. 11k). This difference suggests that cargo coordination near the conserved Twin-His region may differ between plant AMT1 and bacterial AmtB transporters.

Consistent with previous studies showing that ammonium transporters recruit cargos through a conserved aromatic entrance region, additional EM densities were observed near W175 and S262 in all structures (Supplementary Fig. 12a–f). Similar densities have been reported in bacterial AmtB and fungal Mep2 structures and were proposed to correspond to cargo-associated molecules or coordinated water molecules positioned near the extracellular recruitment site^18,19,22^ (Supplementary Fig. 12g–i). However, given the limited resolution and the lack of direct chemical information, the precise identity of these densities cannot be unambiguously assigned. Notably, the density observed in the methylammonium-bound structure exhibited an elongated feature distinct from that observed in the ammonium-containing structures (Supplementary Fig. 12c). Although this feature resembles the methylammonium-associated density previously reported in a bacterial AmtB mutant structure^18^ (Supplementary Fig. 12j), the current cryo-EM data are insufficient to conclusively assign this density as methylammonium.

**Fig. 4:**
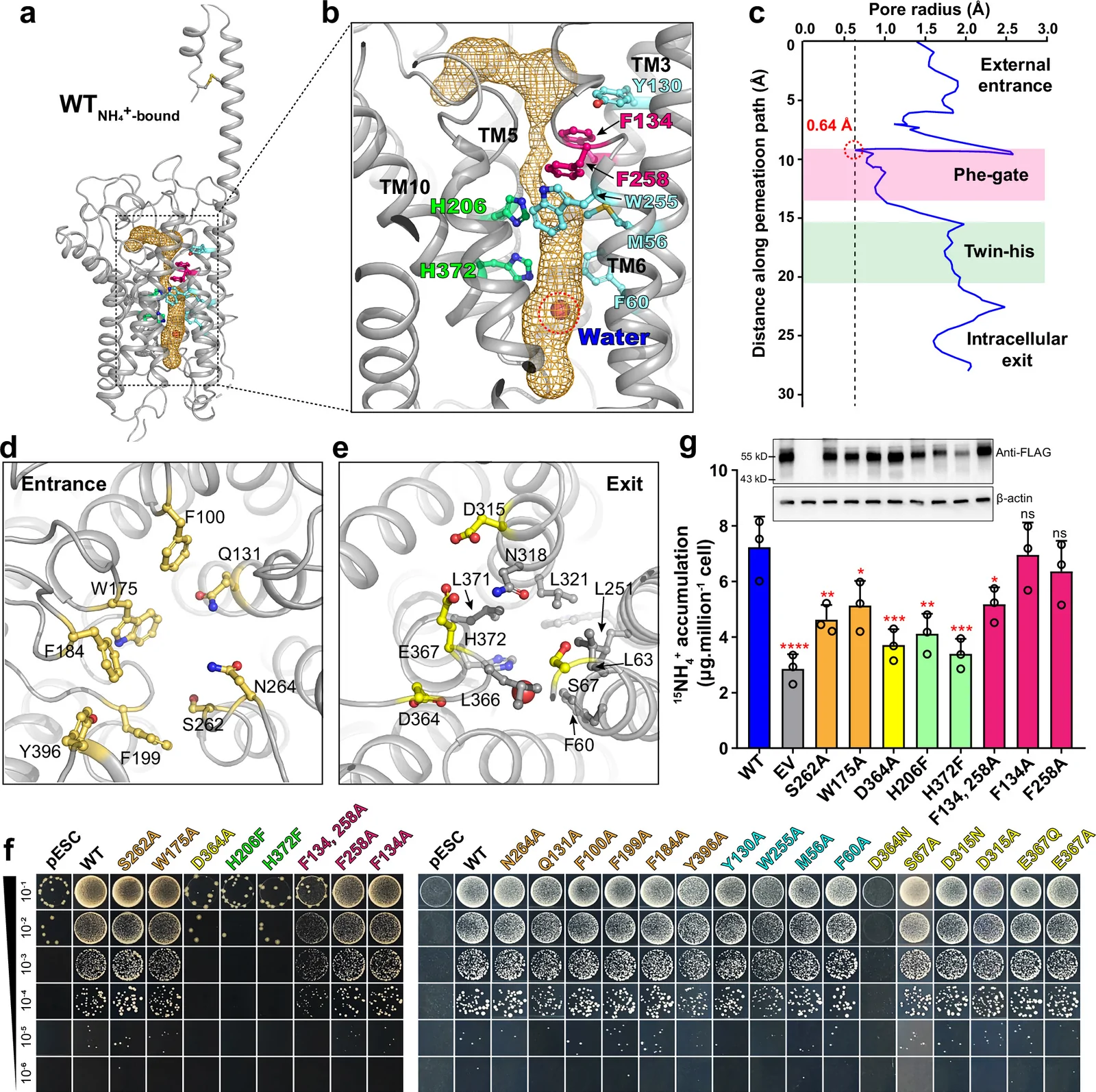
The transport pathway of *At*AMT1;1. **a** Overall structure of *At*AMT1;1 showing the putative cargo conduction pathway, where the pore cavity calculated by Caver 3.0.3 is shown as an orange mesh^77^. Conserved residues lining the pathway are highlighted. **b** Enlarged view of the central pore region. Residues lining the pathway are shown as sticks. **c** Pore radius calculated along the transmembrane region is shown. The Phe-gate site is the narrowest, with a diameter of only 0.64 Å. **d** Top view of the extracellular cargo recruitment vestibule. Conserved aromatic and polar residues are shown as orange sticks. **e** Bottom view of the intracellular exit region. These charged and polar residues are shown as yellow sticks and hydrophobic residues are shown as gray sticks. **f** Growth of the yeast strain 31019b transformed with transport pathway mutants of *At*AMT1;1 in YNB-N medium supplemented with 0.5 mM (NH_4_)_2_SO_4_ at pH 5.5 and incubated at 30 °C for 3 days. The colors of the mutants correspond to the colors of the residues shown in (**b**,**d**,**e**). **g** Results of the cell-based ammonium uptake assay for *At*AMT1;1 transport pathway mutant. Data are means ± SD, *n* = 3 independent experiments. Significances were determined using one-way ANOVA with Dunnett’s multiple comparisons test. ns, not significant, *p < 0.05, **p < 0.01, ***p < 0.001, ****p < 0.0001 for WT versus mutants. The expression level of these mutants was detected by whole-cell WB using anti-Flag antibody.

Pore analysis of the protein structure reveals that, similar to AmtB, the *At*AMT1;1 channel comprises aromatic amino acid residues^18,20^ (Fig. 4a, b). The channel at the Phe-gate is the narrowest, with a diameter of only 0.64 Å (Fig. 4c), which cannot allow the passage of hydrated ammonium ions, which exhibit a flexible diffuse hydration structure with the first hydration shell extending up to 3.5 Å^59^. At the extracellular entrance, in addition to the conserved W175, several aromatic residues F100, F184, and F199 together with the polar residues Q131 and S262 form a cargo recruitment vestibule (Fig. 4d). Structural comparison revealed that W175, S262, Q131, and F199 are highly conserved in bacterial AmtB and fungal Mep2 transporters (Supplementary Fig. 12k, l), suggesting a conserved cargo recognition architecture across AMT family members. At the intracellular side, residues below H372 form a hydrophobic environment similar to that observed in bacterial AmtB and fungal Mep2 (Fig. 4e and Supplementary Fig. 13a, b). However, the outer exit region of *At*AMT1;1 contains several negatively charged and polar residues that are poorly conserved in bacterial AmtB but resemble the corresponding region in fungal Mep2 (Supplementary Fig. 13a, b). Notably, in Mep2, this region adopts a constricted and partially blocked conformation due to tyrosine-mediated occlusion (Supplementary Fig. 13c), which has been proposed to represent an inhibited state^22^.

To evaluate the functional contribution of these residues, we performed yeast complementation and ^15^NH_4_^+^ uptake assay. Mutations at the extracellular entrance generally had limited effects on yeast growth (Fig. 4f), suggesting that these residues are not absolutely required for cargo translocation. Nevertheless, W175A and S262A exhibited moderately reduced ammonium uptake activity compared with the wild type (Fig. 4g). This observation contrasts with previous reports in bacterial AmtB^21,60^, where the corresponding W148A and S219A mutations enhanced transport activity, but is consistent with earlier functional studies of plant AMT1 transporters^29,61^. Together with previous biochemical and computational studies proposing that the conserved W/S vestibule primarily modulates cargo recruitment and transport kinetics rather than serving as a strict selectivity filter^21,62^, these results suggest that the extracellular vestibule mainly facilitates cargo coordination before pore entry. In contrast, disruption of the central conduction elements strongly impaired activity. Single mutations within the Twin-His motif markedly reduced yeast growth and ammonium uptake (Fig. 4f, g), consistent with previous structural and computational studies suggesting that the Twin-His region plays a central role in cargo coordination and may participate in deprotonation/reprotonation events during transport^19,23,63^. Similarly, mutations within the conserved Phe-gate revealed a cooperative functional role for the aromatic constriction. Consistent with previous observations in bacterial AmtB and *At*AMT1;2^21,61^, single mutations of the Phe-gate residues retained substantial transport activity, whereas simultaneous disruption of both phenylalanines strongly impaired ammonium transport (Fig. 4f, g). Previous molecular dynamics simulations proposed that transient tilting or rotamer rearrangement of the phenylalanine side chains is sufficient to permit cargo passage without large-scale pore opening^64,65^. Our functional analyses are consistent with this model and support the idea that the conserved Phe-gate functions as a dynamic aromatic constriction that maintains pore integrity while allowing controlled cargo permeation. W255A mutant displayed only a modest reduction in activity, while most other single-channel mutations had minimal effects (Fig. 4g). Notably, W255 corresponds to W212 in *Escherichia coli* AmtB (Supplementary Fig. 11k), and previous mutational analyses similarly showed that the W212A mutant exhibited reduced transport activity^21^. At the intracellular exit region, systematic mutagenesis further identified D364 as a critical determinant for transport function (Fig. 4f, g), as both D364A and D364N substantially impaired activity (Fig. 4f), suggesting an important role in cargo release or stabilization of the cytoplasmic exit architecture, whereas mutations of neighboring acidic or polar residues showed little effect (Fig. 4f). At present, our structural data do not allow us to assign a precise mechanistic role to D364. However, based on its conserved position and functional importance, we speculate that D364 may contribute to maintaining the local electrostatic environment or structural organization required for efficient cargo release. Together, these results support a model in which the conserved entrance vestibule primarily facilitates cargo recruitment, whereas the Twin-His motif, Phe-gate, and specific intracellular exit residues collectively form the major functional determinants governing selective ammonium permeation in *At*AMT1;1.

### Ammonium transport activity is influenced by pH

**Fig. 5:**
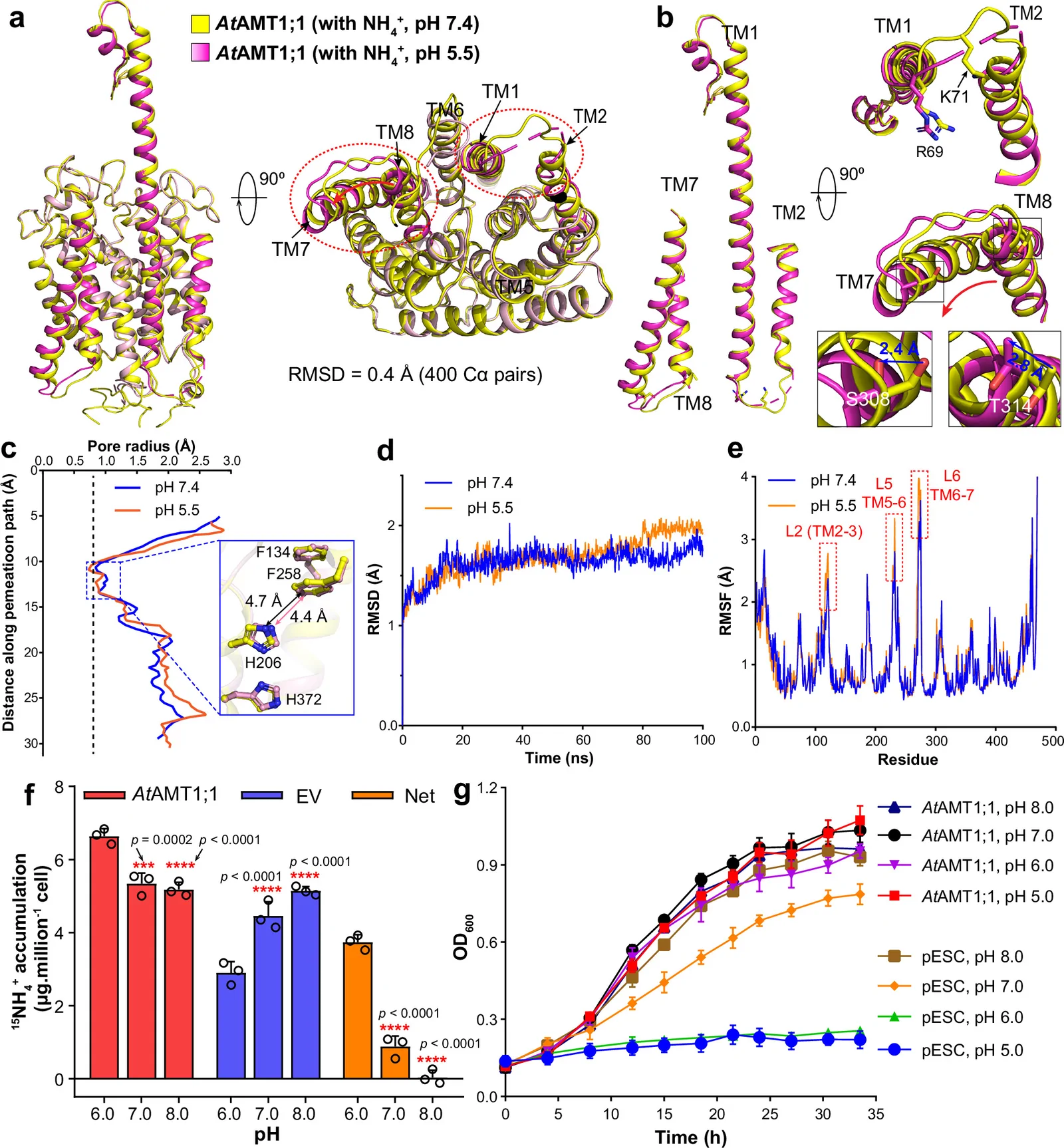
Low pH promotes the transport of ammonium. **a** Structural alignments of wild-type *At*AMT1;1 under different pH conditions, with an RMSD of 0.4 Å (400 Cα atoms aligned). The structure of *At*AMT1;1 under pH 7.4 and pH 5.5 conditions is colored yellow and pink, respectively. The TM1, TM2, TM7, and TM8 of *At*AMT1;1 (pH 5.5) are colored dark pink. **b** Structural alignment of the TM7-TM8 and TM1-TM2 regions of *At*AMT1;1 under varying pH conditions. The red arrow indicates the movement direction of the transmembrane helix. The lower-left panel shows that TM7 and TM8 are displaced by 2.4 Å and 2.8 Å, respectively. **c** Pore-radius analysis of the ammonium conduction pathway in structures of *At*AMT1;1 determined at pH 7.4 and pH 5.5. The inset shows the structural superposition of the Phe-gate and twin-His regions between the two pH conditions. The distance between F258 and H206 decreases from 4.7 Å at pH 7.4 (yellow) to 4.4 Å at pH 5.5 (pink). **d**,**e** RMSD (**d**) and RMSF (**e**) profiles of *At*AMT1;1 at pH 7.4 and 5.5. Curves show mean values calculated from three independent 100-ns MD simulations. **f** Results of the cell-based ammonium uptake assay for *At*AMT1;1 under varying pH conditions. Net accumulation (orange) was calculated by subtracting uptake in empty-vector cells from that in *At*AMT1;1-expressing cells. Data are means ± SD; *n* = 3 independent experiments. Significance was determined using one-way ANOVA with Sidak’s multiple comparisons test. Exact *P* values are indicated in the graph for pH 6.0 versus others. **g** Growth curves in varying pH conditions were measured at OD_600_ for the 31019b strain expressing *At*AMT1;1, and pESC-URA served as a control. Cultures were adjusted to OD_600_ = 0.1 and grown in YNB-N medium supplemented with 0.5 mM (NH_4_)_2_SO_4_ at 30 °C for 33 h. Data are means ± SD; *n* = 3 independent experiments.

Previous studies have suggested that AMT1;1-mediated Am transport may be associated with proton concentration^30,31^. To investigate how environmental proton concentration modulates transport activity, we determined the structure of *At*AMT1;1 under acidic conditions (Supplementary Figs. 9b, 10). We found that in the presence of 1 mM ammonium sulfate (pH 7.4), the non-protein density adjacent to H372 was not significantly enhanced compared to the apo-state (Supplementary Fig. 11a, b). However, in the state with 1 mM ammonium sulfate added at pH 5.5, the non-protein density adjacent to H372 was significantly enhanced (Supplementary Figs. 10b, 11e). Structural alignment of the structures determined at different pH values yielded an RMSD of 0.4 Å (Fig. 5a), indicating that their overall structures are highly similar. For clarity in the following discussion, the loops linking TM1–TM2, TM2–TM3, and so on are denoted L1, L2, etc. At low pH, the TM1–TM2 and TM7–TM8 segments showed an overall outward displacement, and L1 and L5 became more flexible (Fig. 5a, b). TM7 and TM8 were displaced by 2.4 Å and 2.8 Å, respectively, producing a modest widening of the extracellular exit (Fig. 5a). However, we cannot exclude the possibility that pH alters the protonation states of local residues, thereby inducing these subtle conformational changes. Pore-radius analysis revealed subtle pH-dependent remodeling of the conduction pathway. At low pH, the Phe-gate region displayed a modest constriction associated with a small rearrangement between F258 and the adjacent H206 residue of the twin-his motif, while the intracellular exit region became slightly more open (Fig. 5c). Molecular dynamics simulations showed that *At*AMT1;1 maintained comparable overall structural stability under different pH conditions, as reflected by similar RMSD profiles (Fig. 5d). In contrast, RMSF analysis revealed markedly increased fluctuations in the L2, L5, and L6 loop regions under acidic conditions, suggesting enhanced local conformational dynamics without large-scale structural rearrangement (Fig. 5e). To investigate the effect of extracellular pH on AMT1;1-mediated ammonium transport, we performed ^15^NH_4_^+^ uptake assays and yeast complementation analyses under different pH conditions. Cells expressing *At*AMT1;1 accumulated substantially more ^15^NH_4_^+^ than the empty-vector control at all tested pH values, confirming efficient ammonium uptake activity (Fig. 5f). Notably, ^15^NH_4_^+^ accumulation in *At*AMT1;1-expressing cells gradually decreased as the external pH increased, whereas the empty-vector control showed the opposite trend, with higher ammonium accumulation under alkaline conditions (Fig. 5f). Consistently, yeast expressing *At*AMT1;1 exhibited robust growth across different pH conditions, while the empty-vector strain displayed markedly improved growth under alkaline conditions compared with acidic conditions (Fig. 5g). These results may support a model in which extracellular pH modulates local structural dynamics rather than the overall architecture of *At*AMT1;1, thereby influencing cargo transport efficiency.

## Phosphorylation-dependent inhibition by the C-terminal regulatory domain

To prevent ammonium toxicity resulting from excessive uptake, AMT1 transport activity must be tightly regulated^33–35,37^. Studies have shown that *At*AMT1;1 transport activity is modulated by C-terminal phosphorylation, which either enhances or attenuates ammonium uptake^33^. To evaluate the impact of phosphorylation sites on the transport activity of *At*AMT1;1, we generated mutants mimicking phosphorylation, dephosphorylation, and C-terminal truncation. Consistent with previous findings reported by Loqué et al.^39^, both the phosphomimetic mutant T460D and the non-phosphorylatable mutant T460A displayed strongly impaired yeast growth compared with wild-type *At*AMT1;1 (Supplementary Fig. 14a), supporting the importance of the conserved Thr460 regulatory site for transporter function. Notably, the pronounced inhibitory effect observed for T460A differs from the minor functional impairment reported by Neuhäuser et al.^38^ In contrast, mutations at Ser450, including S450D and S450A, showed growth phenotypes similar to wild type (Supplementary Fig. 14a), in agreement with earlier observations^38,39^. Furthermore, we produced C-terminal truncations of different lengths (471–501Δ and 458–501Δ) (Supplementary Fig. 14a). The results of mass photometry demonstrate that truncating the C-terminal region does not affect the stability of *At*AMT1;1 (Supplementary Fig. 14b–d). However, yeast complementation assays revealed a significant reduction in their transport activity (Supplementary Fig. 14a). Together, these results further support the critical role of the conserved Thr460-centered regulatory region in controlling *At*AMT1;1 activity.

**Fig. 6:**
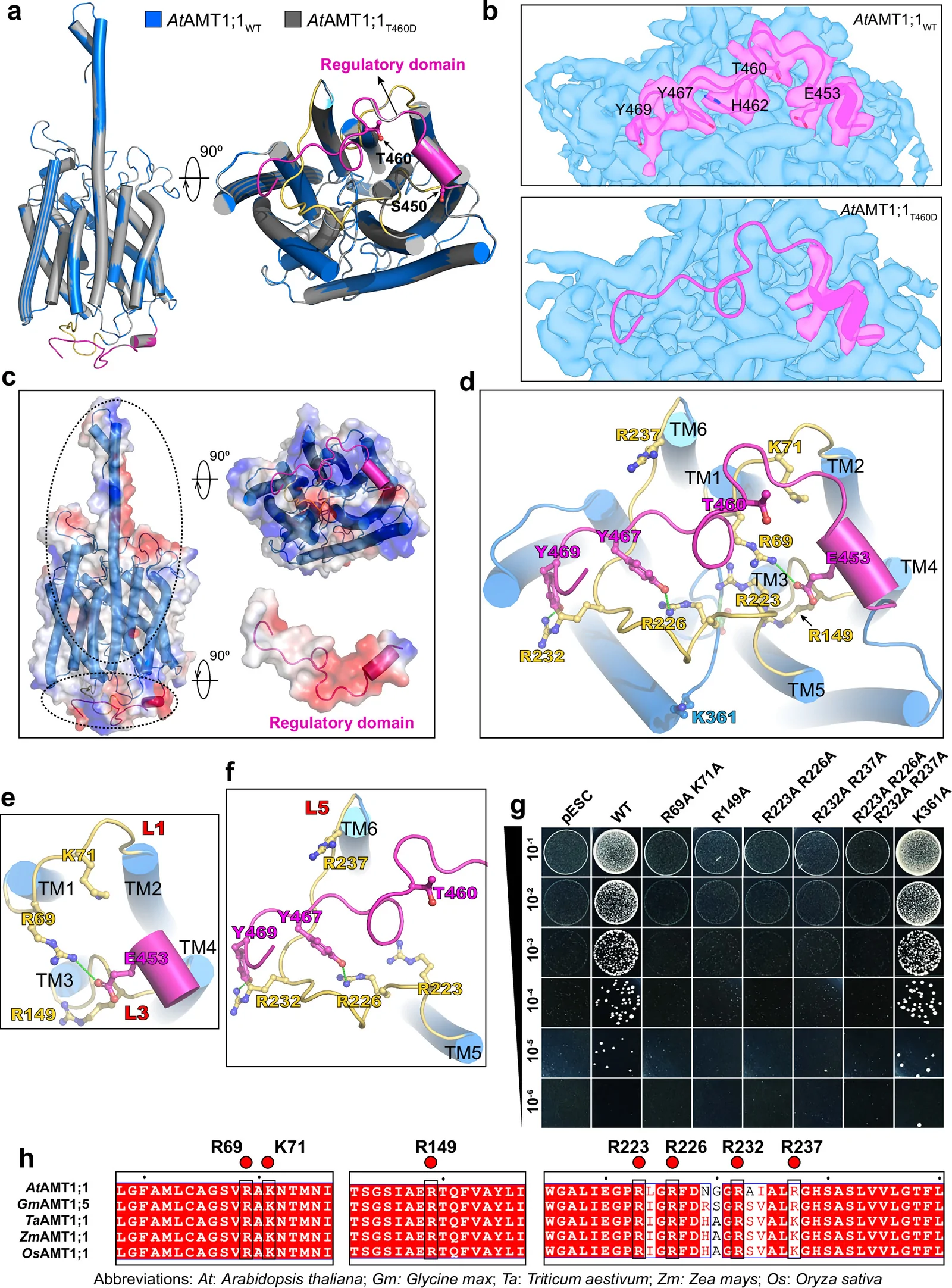
Structural features and functional analysis of the C-terminal regulatory domain. **a** Structural alignments of *At*AMT1;1_WT_ and *At*AMT1;1_T460D_. The amino acids of the regulatory domain and associated intracellular loops are marked in magenta and orange, respectively. **b** Cryo-EM density of the regulatory domain of *At*AMT1;1_WT_ and *At*AMT1;1_T460D_. The contour level for both EM densities is 0.18. **c** A cartoon representation of *At*AMT1;1_WT_ monomer, along with its complete electrostatic surface representation. The electrostatic surface representation of the regulatory domain and other regions are displayed separately on the right side. **d** Details of the overall interaction between the regulatory domain and intracellular loops (L1, L3 and L5), which are colored magenta and orange, respectively. Hydrogen bonds are shown in green dashed lines. **e** Details of the interaction between loop L1, L3 and the regulatory domain. **f** Details of the interaction between loop L5 and the regulatory domain. **g** Growth of the yeast 31019b strain transformed with the mutants of the intracellular loop regions in YNB-N medium supplemented with 0.5 mM (NH_4_)_2_SO_4_ at pH 5.5 and incubated at 30 °C for 3 days. **h** Sequence alignments of the intracellular loop regions of AMT1;1 in various plant species. Sequences for *At*AMT1;1, *Gm*AMT1;5, *Ta*AMT1;1, *Zm*AMT1;1 and *Os*AMT1;1 are publicly available at Uniprot (https://www.uniprot.org) with the following accession codes: *At*AMT1;1: P54144, *Gm*AMT1;5: I1LBS7, *Ta*AMT1;1: Q676V6, *Zm*AMT1;1: A0A3L6G6C1 and *Os*AMT1;1: Q7XQ12.Sequences are aligned using the ClustalW method. The targeted amino acids of the intracellular loop regions are highlighted with a red circle.

To elucidate the phosphorylation-dependent regulatory mechanism, we determined the structure of the phosphomimetic T460D mutant in the presence of 1 mM ammonium sulfate (pH 7.4) (Supplementary Fig. 9c). Structural alignment indicates that the T460D mutant is largely congruent with the wild-type protein (Fig. 6a). In the cryo-EM density of wild-type protein, the C-terminus is well resolved, enabling model building (Fig. 6b and Supplementary Fig. 6c). In contrast, the corresponding region in the T460D mutant exhibits markedly weaker density (Fig. 6b). Notably, the intracellular loops adjacent to the C-terminus are better defined in the wild-type density, particularly the loop L5, than that in the T460D variant (Supplementary Fig. 14e). Structural analysis revealed that the cytosolic C-terminal regulatory domain (CRD) of *At*AMT1;1 establishes extensive electrostatic and hydrogen-bonding interactions with the intracellular loop regions L1, L3 and L5 (Fig. 6c–f). Several positively charged residues within these loops, including R69, K71, R149, R223, R226, R232 and R237, participate in stabilizing the interaction network surrounding the conserved phosphorylation site T460 (Fig. 6d–f). To assess the impact of positively charged amino acid residues on transport activity, we constructed a series of alanine substitution mutants. For several spatially adjacent residues, we designed double or multiple alanine-substitution mutants to minimize potential functional redundancy and better assess the collective contribution of the positively charged interaction surface to transporter regulation. Most mutations markedly impaired yeast growth compared with wild-type *At*AMT1;1 (Fig. 6g). Although K361 is also a positively charged residue, it is located far from the C-terminal regulatory interaction network (Fig. 6d). Consistently, the K361A mutant showed no obvious growth defect compared with wild-type *At*AMT1;1 and therefore served as a positive control (Fig. 6g), further supporting the specificity and reliability of the identified CRD-loop interaction network in regulating transporter activity.

To further investigate the effect of the phosphomimetic mutant on *At*AMT1;1 dynamics, we performed molecular dynamics simulations for both the wild-type and the T460D mutant. RMSD analysis showed that the overall structural stability of the T460D mutant was comparable to that of the wild-type protein throughout the simulation period, indicating that the phosphomimetic mutation does not induce large-scale conformational rearrangements (Supplementary Fig. 14f). In contrast, RMSF analysis revealed increased fluctuations in the L5 loop region (residues 220–240) in the T460D mutant relative to wild type (Supplementary Fig. 14g), suggesting enhanced local conformational flexibility upon phosphorylation mimicry. Given that L5 forms extensive interactions with the C-terminal regulatory domain, these results support the idea that the phosphomimetic mutant at T460 perturbs the CRD-mediated interaction network and alters local loop dynamics, thereby contributing to allosteric inhibition of transporter activity rather than global structural destabilization. Sequence alignment further showed that these positively charged residues are highly conserved among plant AMT1 homologs from diverse species (Fig. 6h), suggesting an evolutionarily conserved regulatory mechanism.

**Fig. 7:**
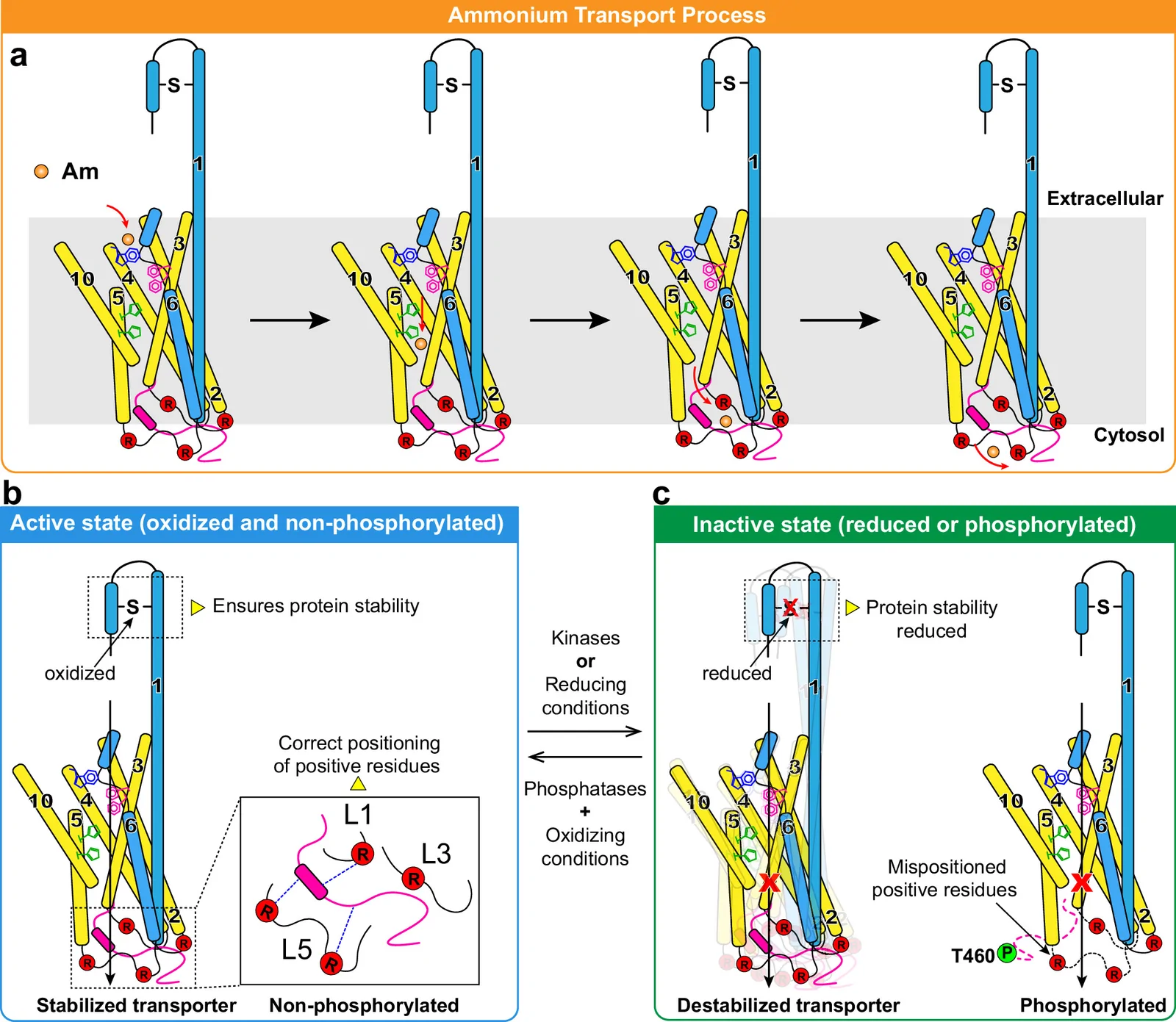
The proposed model for cargo transport and regulation of AMT1 in plants. **a** Schematic representation of the proposed ammonium transport pathway in *At*AMT1;1. Cargos are recruited through an extracellular vestibule enriched in aromatic and polar residues, pass through a narrow central pore containing the Phe-gate and Twin-His motif, and are subsequently released through a cytosolic exit region lined by hydrophobic, polar and charged residues. **b** Proposed active state of *At*AMT1;1. Under oxidizing conditions, the conserved extracellular N-terminal disulfide bond is formed, contributing to transporter stability. In the non-phosphorylated state, the C-terminal regulatory domain (CRD) establishes extensive electrostatic interactions with conserved positively charged intracellular loops, maintaining their proper organization and supporting efficient ammonium transport. **c** Proposed inactive or attenuated state of *At*AMT1;1. Reduction of the N-terminal disulfide bond destabilizes the transporter and compromises transport activity. In parallel, phosphorylation of T460 may weaken interactions between the CRD and intracellular loops, increase local conformational flexibility, and thereby reduce transport activity. Together, extracellular redox conditions and intracellular phosphorylation may coordinately regulate the activity of plant AMT1 transporters.

Structural comparison with bacterial and fungal ammonium transporters further suggests that positively charged intracellular loops may represent a conserved regulatory hotspot within the AMT/MEP/Rh superfamily (Supplementary Fig. 15a–e). In both bacterial AmtB and fungal Mep2, intracellular loop regions enriched in positively charged residues occupy positions analogous to the L1, L3 and L5 loops observed in plant *At*AMT1;1 (Fig. 6c, d and Supplementary Fig. 15a–e). Despite this structural conservation, the regulatory strategies appear to differ substantially among kingdoms. In bacterial AmtB, transport activity is regulated through interaction with the signal transduction protein GlnK^14,66,67^. Comparison between the apo AmtB structure and the AmtB–GlnK complex revealed stabilization of the C-terminal region together with pronounced rearrangements of intracellular loops, particularly L1, L3 and L5, suggesting that loop reorientation is associated with transporter regulation (Supplementary Fig. 15a, b). In fungal Mep2, phosphorylation-dependent regulation appears to involve a distinct mechanism. Although phosphomimetic mutations do not induce large-scale displacement of intracellular loops, phosphorylation triggers substantial rearrangement of the C-terminal region (Supplementary Fig. 15c, d), and previous studies proposed that intracellular loops function as key elements of an autoinhibitory network^22^. These observations are reminiscent of our findings in plant AMT1;1, in which phosphorylation of the C-terminal regulatory domain remodels electrostatic interactions with conserved positively charged intracellular loops and alters their local dynamics (Fig. 6b–h and Fig. 7b, c). Together, these structural comparisons support the idea that intracellular positively charged loops constitute a conserved allosteric regulatory module in AMT-family transporters.

### The transport pathway and regulation mechanism of *At*AMT1;1

Based on the structural, biochemical and functional analyses, we propose a mechanistic model for ammonium transport and regulation in plant AMT1 transporters (Fig. 7). In the active state, extracellular ammonium is initially recruited at the entrance vestibule, where the conserved tryptophan residue W175 may contribute to cargo recognition through aromatic interactions near the extracellular vestibule^21,62,64^ (Fig. 7a). The cargo subsequently enters the narrow pore containing the conserved Twin-His motif and traverses the transmembrane pathway toward the cytosolic side^19–21,27,63^ (Fig. 7a). Structural and mutational analyses reveal a conserved transport pathway lined by key pore residues that likely mediate ammonium translocation.

Our data further suggest that AMT1 activity is coordinately regulated by both the N-terminal extracellular region and the cytosolic C-terminal regulatory domain (Fig. 7b, c). The conserved N-terminal disulfide bond stabilizes the transporter architecture and may function as a redox-sensitive switch (Fig. 7b). Under oxidizing conditions, the disulfide bond maintains the structural integrity and oligomeric stability of AMT1 (Fig. 7b), whereas reducing conditions disrupt transporter stability and impair activity (Fig. 7c). In parallel, the cytosolic C-terminal regulatory domain forms extensive electrostatic interactions with conserved positively charged residues located in intracellular loops (Fig. 7b). Phosphorylation at T460 likely disrupts this interaction network, alters loop positioning and enhances local conformational flexibility, thereby allosterically inhibiting transport activity (Fig. 7c). Together, these findings support a dual regulatory model in which extracellular redox conditions and intracellular phosphorylation cooperatively modulate AMT1 activity to maintain ammonium homeostasis in plants (Fig. 7b, c).

### Conserved ammonium-recognition residues are required for *At*AMT1;1 transport activity

**Fig. 8:**
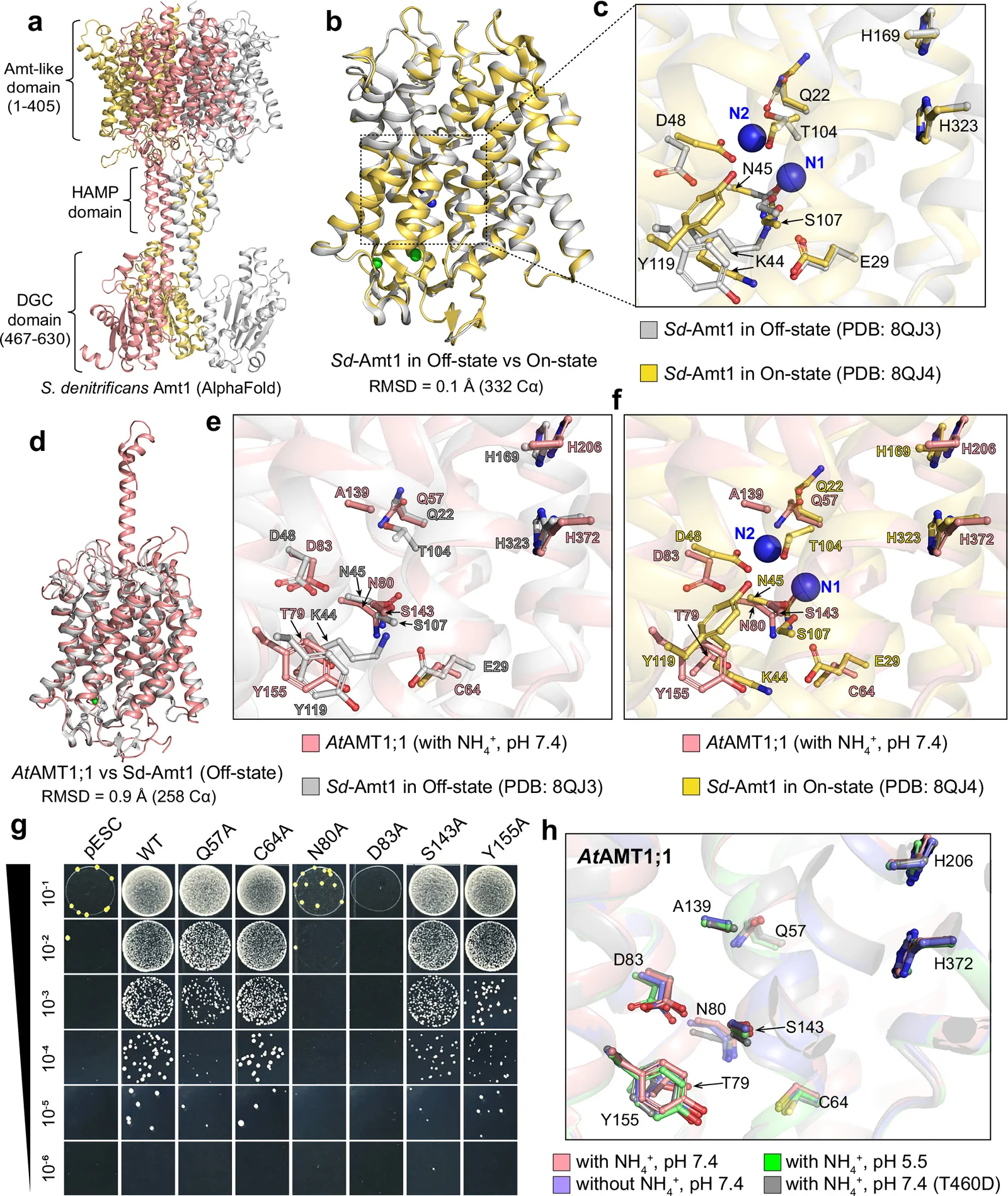
Conserved ammonium-recognition architecture contributes to *At*AMT1;1-mediated ammonium transport. **a** AlphaFold-predicted full-length structure of *Shewanella denitrificans* Amt1 (*Sd*-Amt1), consisting of an N-terminal Amt-like transmembrane domain connected through a HAMP linker to a C-terminal diguanylate cyclase (DGC) signaling domain. **b** Structural superposition of the reported Off-state (PDB: 8QJ3) and On-state (PDB: 8QJ4) *Sd*-Amt1 structures. **c** Close-up views of the N1 and N2 ammonium-binding sites in the Off-state and On-state *Sd*-Amt1 structures. **d** Structural superposition of *At*AMT1;1 and the Off-state *Sd*-Amt1 transmembrane domains, revealing a highly conserved overall fold. **e**,**f** Detailed comparison of the N1 and N2 binding-site regions between *At*AMT1;1 and the Off-state (**e**) or On-state (**f**) *Sd*-Amt1 structures. **g** Growth of the yeast strain 31019b transformed with mutants within the N1/N2-equivalent region of *At*AMT1;1 in YNB-N medium supplemented with 0.5 mM (NH_4_)_2_SO_4_ at pH 5.5 and incubated at 30 °C for 3 days. **h** Structural comparison of *At*AMT1;1 determined in the presence or absence of NH_4_^+^, as well as in low-pH and T460D states at the corresponding N1/N2 regions.

Recent structural studies characterized *Shewanella denitrificans* Amt1 (*Sd*-Amt1) as an ammonium-sensing Amt protein in which the Amt-like transmembrane domain is coupled to a downstream signaling module containing HAMP and diguanylate cyclase (DGC) domains^40,42^ (Fig. 8a). In *Sd*-Amt1, two conserved ammonium-binding sites, termed N1 and N2, were proposed to mediate ligand-dependent switching between Off- and On-state^42^ (Fig. 8b, c). We therefore compared the transmembrane region of *At*AMT1;1 with the corresponding regions of the Off- and On-state structures of *Sd*-Amt1. The transmembrane region of *At*AMT1;1 closely resembles that of *Sd*-Amt1 (Fig. 8d), with several residues surrounding the N1/N2 sites, including Q57, N80, D83, S143 and Y155, conserved between the two proteins (Fig. 8e, f). The ammonium-bound *At*AMT1;1 structure more closely resembles the Off-state of *Sd*-Amt1, with an RMSD of 0.9 Å (Fig. 8d–f). In contrast, the Off- and On-state of *Sd*-Amt1 exhibit a distinct conformational difference associated with its signaling state (Fig. 8b, c).

We next examined the functional contribution of residues within this conserved region. Yeast complementation assays showed that substitution of Q57, N80 and D83 substantially impaired growth under ammonium-limiting conditions, whereas mutations of C64, S143 and Y155 had little or no apparent effect under the same conditions (Fig. 8g). These results indicate that several residues conserved within the N1/N2-associated ammonium-recognition region are functionally important for *At*AMT1;1-mediated ammonium transport.

We further compared the corresponding regions across the different structures of *At*AMT1;1 determined under distinct ligand and regulatory conditions. In *Sd*-Amt1, distinct non-protein densities are associated with the N1 and N2 sites in the On-state structure, whereas comparable densities are not observed in the Off-state (Supplementary Fig. 16a, b). In *At*AMT1;1, recurring non-protein densities were observed near Q57, N80 and D83 in structures determined in the presence or absence of NH_4_^+^, as well as in the methylammonium-bound, low-pH and T460D states (Supplementary Fig. 16c–h). Where sufficiently resolved, these densities were consistent with ordered water molecules and were modeled accordingly. By contrast, no corresponding additional density was apparent in the H372F structure (Supplementary Fig. 16f). However, the occurrence of these densities did not show a consistent relationship with ammonium or methylammonium binding, and we did not observe an evident state-dependent rearrangement comparable to that associated with the N1/N2 sites in *Sd*-Amt1 (Fig. 8h).

Together, these structural and functional analyses identify a conserved region in *At*AMT1;1 that contributes to ammonium transport and contains recurring non-protein densities across multiple structural states.

## Discussion

In this study, we combined cryo-EM, biochemical analyses, and molecular dynamics simulations to establish a structural framework for ammonium transport and regulation in the plant AMT1 family. By resolving *At*AMT1;1 in multiple transport- and regulation-related states (Supplementary Fig. 1d, Supplementary Fig. 5 and Supplementary Fig. 9), we identified the cargo conduction pathway (Fig. 4a–e and Fig. 7a), characterized pH-associated conformational dynamics (Fig. 5), and revealed how phosphorylation-dependent rearrangement of the C-terminal regulatory domain allosterically inhibits transport activity (Fig. 6 and Fig. 7b, c). In addition, our structures reveal the conserved extracellular N-terminal disulfide bond within plant AMT1 proteins, providing a structural basis for previous observations that N-terminal cysteine residues contribute to AMT1 stability and oligomerization^52^. Our biochemical analyses further suggest that this disulfide bond may couple extracellular redox conditions to transporter stability and activity (Figs. 3, 7b, c and Supplementary Fig. 8). Together, these findings support a multi-layered regulatory model that couples transport with environmental and post-translational signals in plant ammonium transporters.

Our structures reveal that the overall transport core of *At*AMT1;1 is highly conserved relative to bacterial Amt proteins, supporting a common structural framework for ammonium conduction (Supplementary Fig. 4c and Supplementary Figs. 11k, 12k). However, whether plant AMTs operate through the same mechanism remains debated, particularly given the diverse electrophysiological properties reported for different plant AMTs^26–31,68^. Rather than defining the transported chemical species, our structures delineate a cargo pathway extending from the extracellular vestibule to the cytosolic exit (Fig. 4a–e). Elucidating the transport mechanism of AMT proteins remains a major challenge, particularly with respect to the identity of the transported species and whether ammonium undergoes protonation or deprotonation during translocation, as well as where these processes occur. Addressing these questions will require future studies using more precise structural, biophysical, and electrophysiological approaches.

One notable finding of this study is the identification of two spatially separated regulatory modules that cooperatively control *At*AMT1;1 activity. On the extracellular side, the conserved intra-monomer N-terminal disulfide bond is required for maintaining oligomeric stability and transport activity (Fig. 3c–e and Supplementary Figs. 7b–d, 8). The sensitivity of the transporter to reducing and oxidizing conditions further raises the possibility that AMT1 proteins may function as redox-responsive nutrient transporters (Fig. 7b, c). However, the precise molecular mechanisms by which this intramolecular disulfide bond, located distal to the transmembrane region, modulates protein stability and transport activity still require further experimental and computational validation. One possibility is that the N-termini of neighboring protomers are positioned in close proximity, such that disruption of the disulfide bond may increase local flexibility and indirectly perturb inter-subunit coupling required for efficient transport. On the cytosolic side, phosphorylation of T460 remodels the interaction network between the C-terminal regulatory domain and intracellular loops (Fig. 6c–f), leading to increased local flexibility and transport inhibition (Fig. 6b and Supplementary Fig. 14a, e–g). The strong evolutionary conservation of the positively charged residues involved in this interaction network suggests that this regulatory mechanism may be broadly conserved among plant AMT1 proteins (Fig. 6h). Together, these findings support a model in which extracellular redox cues and intracellular phosphorylation converge to modulate transporter activity through distinct yet coordinated structural mechanisms.

Our findings place *At*AMT1;1 within a conserved structural framework shared with the ammonium-sensing bacterial protein *Sd*-Amt1 (Fig. 8e, f). The N1/N2-equivalent region is conserved in *At*AMT1;1, and several residues within it contribute to ammonium transport (Fig. 8g), highlighting its functional importance in the plant transporter. However, the conserved recognition architecture of *At*AMT1;1 is not accompanied by the ligand-dependent conformational changes observed in *Sd*-Amt1 (Fig. 8e, f, h). Moreover, *At*AMT1;1 lacks the HAMP–DGC module that connects the bacterial Amt domain to downstream signaling^42^. Thus, our data do not demonstrate a transceptor function for *At*AMT1;1, but raise the possibility that the conserved substrate-recognition region may contribute to regulatory functions beyond transport through mechanisms distinct from the bacterial signaling pathway. Whether this conserved region has additional roles in regulating plant AMT activity, or functions primarily within the transport process, remains unresolved. Direct biochemical and cellular analyses will be required to determine whether ligand binding to this region is coupled to regulatory or signaling outputs in plant AMT proteins.

Given the central role of AMT1 transporters in high-affinity ammonium uptake^4,13,16,47,49^. Understanding how plants precisely regulate ammonium acquisition is essential for improving nitrogen-use efficiency. Our work provides a mechanistic framework for engineering AMT activity through rational manipulation of transport and regulatory modules. Moreover, these findings establish plant AMTs as dynamically regulated nutrient transporters that integrate environmental and intracellular signals to maintain nitrogen homeostasis.

## Methods

### Protein expression and purification

The cDNA sequence encoding full-length wild-type (WT) *Arabidopsis thaliana* AMT1;1 (UniProt accession code: P54144) or its variants was subcloned into a pCAG vector containing a C-terminal FLAG tag. A list of the primers used is provided in Supplementary Data 1. For protein expression, the plasmid was transfected into HEK293F cells cultured in SMM 293T-II medium (M293TII, Sino Biological) at 37 °C, 130 rpm, and 5% CO₂. When the cell density reached approximately 3 × 10⁶ cells ml⁻¹ (in 800 ml total culture volume), 1.5 mg of plasmid DNA and 4 mg of polyethylenimine (PEI, Yeasen) were pre-incubated in 45 ml of fresh medium for 15 min. The mixture was then added to the culture, followed by static incubation for 15 min. Transfected cells were cultured at 37 °C, 130 rpm, and 5% CO₂ for 12 h. Subsequently, 10 mM sodium butyrate (Macklin) was added, and the cells were further cultured at 30 °C for 48 h before harvesting by centrifugation at 3000 × g for 10 min.

Cell pellets were resuspended in lysis buffer containing 25 mM HEPES-NaOH (pH 7.4) and 150 mM NaCl. Following the addition of 1.5% (w/v) n-dodecyl-β-D-maltopyranoside (DDM, Anatrace), 0.3% (w/v) cholesteryl hemisuccinate (CHS, Sigma-Aldrich), and a protease inhibitor cocktail containing 1 mM phenylmethylsulfonyl fluoride (PMSF) and Protease Inhibitor Cocktail (TargetMol), the suspension was incubated at 4 °C with gentle rotation at 25 rpm for 2 h. The insoluble fraction was pelleted by centrifugation at 100,000 × *g* for 60 min (Beckman, Type 70 Ti rotor), and the supernatant was incubated with anti-FLAG M2 affinity resin (Sigma-Aldrich) at 4 °C for 1 h.

The resin was rinsed with 40 ml of buffer A containing 25 mM HEPES-NaOH (pH 7.4), 150 mM NaCl, and 0.01% (w/v) lauryl maltose neopentyl glycol (LMNG; Anatrace), and the bound protein was eluted with the same buffer supplemented with 200 μg ml⁻¹ FLAG peptide (GL Biochem). The protein eluent was further concentrated using a 50 kDa cut-off Centricon (Millipore) and subjected to size-exclusion chromatography on a Superose 6 Increase 10/300 column (Cytiva) equilibrated in buffer B containing 25 mM HEPES-NaOH (pH 7.4), 150 mM NaCl, and 0.005% (w/v) LMNG. For purification of the cargo-bound AMT1;1 protein, 1 mM ammonium sulfate or 10 mM MeA (Macklin) was maintained in all buffers throughout the process, while all other procedures remained identical. For purification of AMT1;1 protein under pH 5.5 condition, the buffer B was substituted with buffer C containing 25 mM MES-NaOH (pH 5.5), 150 mM NaCl, and 0.005% (w/v) LMNG, while all other procedures remained unchanged. Peak fractions were pooled and concentrated using a 50 kDa cut-off Centricon (Millipore) to a final concentration of 12 mg ml⁻¹ for cryo-EM analysis.

To prepare protein samples for the microscale fluorescent thermal stability and mass photometry assay, the protein was expressed and purified in the same manner. Peak fractions were pooled and concentrated to 1 mg ml^−1^.

### Yeast complementation and transport activity assays

The wild-type and mutant forms of *Arabidopsis thaliana* AMT1;1 were cloned into the pESC-URA vector (Agilent) and transformed into the ammonium uptake-deficient yeast strain 31019b (*Δmep1 Δmep2 Δmep3 ura3*) using the polyethylene glycol/lithium acetate (PEG/LiAc)-mediated transformation method^11,22,39^. Following transformation, yeast transformants were cultured at 30 °C for 3 days on solid synthetic complete medium lacking uracil (SC-URA; Fungenome) supplemented with 2% D-glucose (Biosharp) for positive screening. The positive yeast cells were precultured at 30 °C in liquid SC-URA medium supplemented with 2% (w/v) D-galactose (Macklin) until the OD_600_ reached about 0.6. The cells were then harvested, washed three times with sterile water, and resuspended to an OD_600_ of 1.

For complementation growth assays, serial dilutions (10⁻¹ to 10⁻⁶) of the cell suspension (10 µl per spot) were plated onto solid yeast nitrogen base medium lacking ammonium sulfate and amino acids (YNB-N; Difco, pH 5.5), but supplemented with 0.5 mM ammonium sulfate (Macklin) as the sole nitrogen source. The plates were incubated at 30 °C for 3 days. Yeast cells transformed with the empty pESC-URA vector were used as negative controls. To measure growth curves, cultures were adjusted to an OD₆₀₀ of 0.1 and grown at 30 °C for 33 hs in YNB-N medium adjusted to the indicated pH values and supplemented with 0.5 mM ammonium sulfate. All experiments included three independent biological replicates. To observe the toxic effect of MeA, yeast cell suspensions were spotted on YNB-N medium (pH 5.5, containing 0.5 mM L-Arg) supplemented with 0, 5 and 10 mM MeA and incubated at 30 °C for 3 days.

### Microscale fluorescent thermal stability assay

The thermal stability of both WT and mutant AMT1;1 was assessed using the fluorescent probe *N*-(4-(7-diethylamino-4-methyl-3-coumarinyl) phenyl) maleimide (CPM; Sigma). The CPM dye was first dissolved in dimethyl sulfoxide (DMSO; Sigma) to a final concentration of 4 mg ml⁻¹ and subsequently diluted 40-fold in a buffer composed of 25 mM HEPES-NaOH (pH 7.4), 150 mM NaCl, and 0.01% (w/v) DDM immediately before use. For each reaction, 1.5 μl of the diluted CPM solution was mixed with 2 μg of either WT or mutant AMT1;1 protein on ice, protected from light. The final reaction volume was adjusted to 20 μl. Thermal unfolding was monitored in a Roche LightCycler480 real-time PCR instrument by increasing the temperature at a constant rate of 5 °C min⁻¹. Fluorescence signals were continuously recorded with excitation and emission wavelengths set at 440 nm and 488 nm, respectively. The temperature range for the assay extended from room temperature to 90 °C. Fluorescence signals were continuously recorded during heating. The first derivative of the fluorescence signal −d(RFU)/dT was calculated to generate melting curves. The temperature corresponding to the maximal unfolding transition was defined as the apparent melting temperatures (Tm) value. All measurements were performed with four independent replicates.

### Cryo-EM sample preparation and data acquisition

For all AMT1;1 sample preparation, 4 µl aliquots of purified proteins were applied to the holey carbon grid (Quantifoil Au R1.2/1.3, 300 mesh) glow-discharged for 10 s using H_2_ and O_2_. The grid was then blotted with a Vitrobot Mark IV (Thermo Fisher Scientific) operating at 8 °C and 100% humidity with a blotting time of 3 s, and plunged into liquid ethane cooled by liquid nitrogen. The grid was loaded into a Titan Krios (FEI) electron microscope operating at 300 kV, equipped with the GIF Quantum energy filter and a K3 or Falcon4 direct electron detector (Gatan). Images were recorded using EPU in the super-resolution mode. Defocus values varied from −1.0 to −2.0 μm. Each micrograph stack was acquired dose-fractionated into 32 frames with a total dose of 50 *e*^-^ Å^−2^.

### Image processing

Micrograph stacks were imported into cryoSPARC (v.4.6.2)^69^ and processed by Patch Motion Correction for beam-induced motion correction, followed by Patch CTF Estimation for determination of the contrast transfer function parameters. Data processing for WT, mutant or cargo-bound AMT1;1 followed a similar approach, with the detailed procedure for wild-type AMT1;1 provided below as an example.

For wild-type AMT1;1 in apo-state, 414,032 particles were automatically picked by the template picker in cryoSPARC from 435 micrograph stacks. After multiple rounds of 2D classification, 178,359 selected particles were subjected to ab initio reconstruction into five classes with C1 symmetry imposed. 65,843 particles were selected for non-uniform refinement, finally yielding a reconstruction map at 2.9 Å resolution with C3 symmetry. Detailed flowcharts for all data processing can be found in Supplementary Figs. 1, 5, 9.

### Model building and refinement

The initial protein model was built by fitting predicted models of AlphaFold3 into the cryo-EM maps with UCSF Chimera^70,71^. Models were then adjusted and built de novo based on the cryo-EM density features using Coot^72^. The initial atomic model was built into the C1-symmetry reconstructed map and refined using Phenix real-space refinement^73^. Each protomer was individually inspected and adjusted according to the corresponding density. The final structural analyses were performed using the C3-symmetry refined model because of its improved map quality in the core transporter region. Statistics of the 3D reconstruction and model refinement can be found in Supplementary Table 1.

### Cell-based ammonium uptake assays

HEK293F cells at a density of 2 × 10^6^ cells per ml were transfected with WT, mutant *At*AMT1;1 or the empty-vector plasmid. After 36 hours of culture at 37 °C, 130 rpm, and 5% CO_2_, cells were harvested by centrifugation and resuspended for the ammonium transport assay. For time course ^15^NH_4_^+^ uptake assay, 3 × 10^6^ cells were resuspended with 500 μl of uptake buffer (10 mM Na_2_HPO_4_, 1.8 mM KH_2_PO_4_, 2.7 mM KCl, 137 mM NaCl, pH adjusted to 5.5 by HCl) containing 1 mM stable isotope-labeled ^15^N-ammonium nitrate (Macklin) at room temperature. At the designated time points, the uptake process was terminated by rapid centrifugation followed by two washes with ice-cold PBS buffer (pH 7.4). For the concentration-dependent ^15^NH_4_^+^ uptake assay, 3 × 10^6^ cells were resuspended with 500 μl of uptake buffer containing increasing concentrations of stable isotope-labeled ^15^N-ammonium (0–1000 μM) at room temperature for 10 min. Uptake process was terminated by rapid centrifugation followed by two washes with ice-cold PBS buffer (pH 7.4). For ammonium uptake assays at different pH values, the pH value of PBS buffer was adjusted to 6.0, 7.0, and 8.0, while all other procedures remained identical.

The ^15^N content was analyzed using a continuous-flow isotope ratio mass spectrometer (DELTA V Advantage) coupled with an elemental analyzer (EA-HT, Thermo Fisher Scientific, Inc., Bremen, Germany). The ^15^N-ammonium accumulation was calculated as the total amount of ^15^N accumulated per million cells. For the concentration-dependent ^15^NH_4_^+^ uptake assay, uptake rates were normalized to cell number and expressed as ng min⁻¹ million⁻¹ cells. Net *At*AMT1;1-mediated transport activity was calculated by subtracting the uptake measured in empty-vector cells from that measured in *At*AMT1;1-expressing cells. Concentration-dependent uptake curves were fitted using the Michaelis–Menten equation in GraphPad Prism.

### Mass photometry

Mass photometry (MP) experiments were conducted using a Refeyn TwoMP instrument^58^. Prior to data acquisition, 15 μl of MP-assay buffer was added onto a clean slide and focused until a stable background signal was obtained. MP-assay buffer B contained 25 mM HEPES-NaOH (pH 7.4), 150 mM NaCl, and 0.005% (w/v) LMNG. For sample measurements, purified protein was rapidly added and mixed directly on the slide to a final concentration of 50 nM. Movies were recorded immediately after sample addition using Refeyn AcquireMP with an acquisition time of 60 s. All samples and calibration standards were prepared in the same buffer conditions used for the corresponding measurements. To calibrate molecular mass, a mixed protein standard containing bovine serum albumin (BSA, 66 kDa) and thyroglobulin (660 kDa) was diluted to a final concentration of 20 nM in the corresponding MP-assay buffer immediately before measurement. The resulting calibration curve was generated according to the manufacturer’s instructions and subsequently applied to convert scattering contrast values into molecular mass estimates.

For redox-treatment experiments, purified *At*AMT1;1 was incubated with 20 mM DTT at 4 °C for 2.5 h. The protein sample was subsequently loaded onto a prepacked desalting column (Smart-Lifesciences) equilibrated with buffer B. The peak fraction was then incubated with hydrogen peroxide (H₂O₂) at different concentrations for 30 min and subsequently subjected to mass photometry analysis. Mass distributions were obtained from particle landing events recorded in the movies and analyzed using Refeyn DiscoverMP software. Peak positions corresponding to different oligomeric states were determined from the mass histograms generated by DiscoverMP.

### Molecular dynamics (MD) simulation

The 2.9 Å cryo-EM structure of AMT1;1 was used as the initial model, the wild-type and T460D mutant structures were constructed and incorporated into a planar lipid bilayer constituted by 1-palmitoyl-2-oleoyl-sn-glycero-3-phosphorylcholine using the CHARMM-GUI software, respectively^74^. Each system was then hydrated using the TIP3P water model and neutralized with 150 mM NaCl. A periodic rectangular box with dimensions of 120 × 120 × 210 Å³ was generated, which has approximately 292,500 atoms. The CHARMM36m force field was applied for the protein and lipids. To initiate the relaxation of the system, the energy minimization was performed using the steepest descent algorithm. The simulation system was then pre-equilibrated under multistep isothermal-isovolumetric (NVT) and isothermal-isobaric (NPT) conditions, with the restraints progressively reduced at each step. Unrestrained production simulations were conducted with a 2-fs time step at 303.5 K. The WT simulations were run for 100 ns at pH 7.4 and pH 5.5, respectively. The T460D simulations were run for 100 ns at pH 7.4. Each structure was simulated for three repeats with different initial velocities. The simulations were carried out using the GROMACS 2022.5 package^75,76^, figures were made using GraphPad Prism software.

### Western blotting

For analysis of protein expression, 5 ml of HEK293F cells expressing wild-type *At*AMT1;1 or the indicated variants were harvested at 3000 × *g* for 10 min. Cell pellets were resuspended in 1 ml of the membrane-solubilization buffer used for protein purification and incubated at 25 rpm for 2 h. Insoluble material was removed by centrifugation at 12,000 × *g* for 30 min at 4 °C, and 0.5 μl of each supernatant was resolved by 14% SDS–PAGE. Proteins were transferred to PVDF membranes (Millipore) by wet electroblotting at 100 V for 90 min. Membranes were blocked with 5% nonfat milk in TBST for 1 h and incubated for 1 h with anti-FLAG (TransGen Biotech, HT201; 1:3,000) or anti-β-actin (TransGen Biotech, HC201; 1:3000) primary antibodies. After five 5-min washes with TBST, membranes were incubated for 1 h with HRP-conjugated goat anti-mouse IgG secondary antibody (TransGen Biotech, HS201-01; 1:5,000), followed by five additional 5-min washes with TBST. Immunoreactive bands were visualized using an enhanced chemiluminescence detection kit (Seven Biotech, SW134).

### Reporting summary

Further information on research design is available in the Nature Portfolio Reporting Summary linked to this article.

## Supplementary information

Supplementary Information

Description of Additional Supplementary Files

Supplementary Data 1

Reporting Summary

Peer Review file

## Source data

Source Data

## Data Availability

The 3D cryo-EM maps of the WT (without NH_4_^+^, pH 7.4), WT (with NH_4_^+^, pH 7.4), WT (with MeA, pH 7.4), H372F (without NH_4_^+^, pH 7.4), WT (with NH_4_^+^, pH 5.5), and T460D (with NH_4_^+^, pH 7.4) of *At*AMT1;1 have been deposited in the Electron Microscopy Data Bank (EMDB, https://www.ebi.ac.uk/emdb/) under the accession number **EMD-68466**, **EMD-68512**, **EMD-68580**, **EMD-68504**, **EMD-81185** and **EMD-68515**, respectively. The corresponding atomic coordinates have been deposited in the Protein Data Bank (PDB, https://www.rcsb.org/) under the accession code **22MG**, **22NK**, **22OY**, **22NG**, **27IF** and **22NN**, respectively. Source data are provided with this paper.
