# Supplementary material for: Structural basis of ammonium transport and coordinated regulation of AMT1;1 in Arabidopsis thaliana
Source: Nat Commun. 2026 Oct 10;17:11526. doi: 10.1038/s41467-026-78457-1 (PMC13745463; doi:10.1038/s41467-026-78457-1)
Supplement: Supplementary file 1 [file 41467_2026_78457_MOESM1_ESM.pdf]

Supplementary Figures and Figure Legends

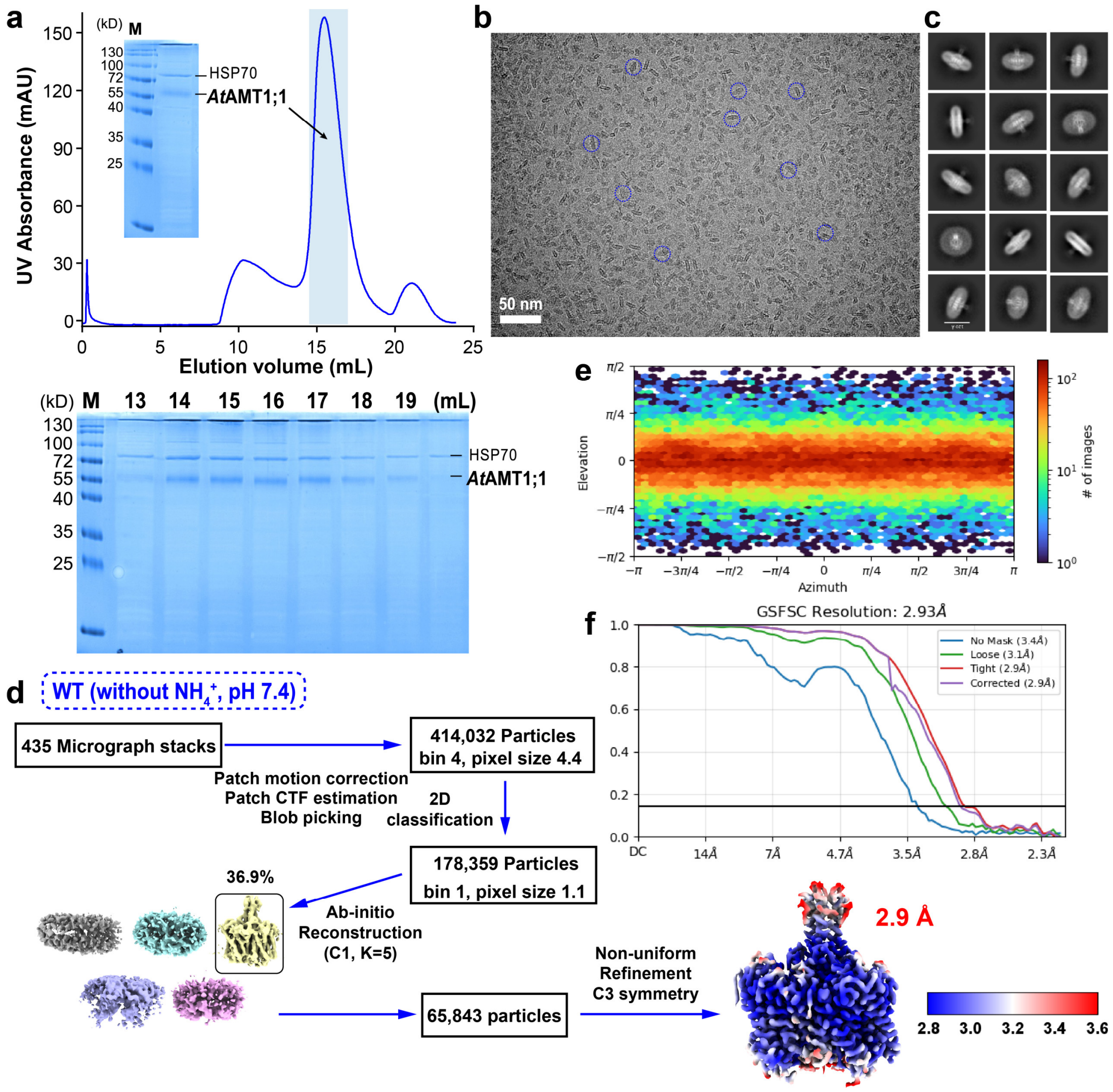

Supplementary Fig. 1 Protein purification and Cryo-EM analysis of apo *At*AMT1;1$_{WT}$ at pH 7.4.

a, Size-exclusion chromatography (SEC) profile of purified *At*AMT1;1. The peak fraction used for cryo-EM analysis is highlighted. The inset shows SDS–PAGE analysis of the affinity-purified eluate, and the lower panel shows SDS–PAGE analysis of the indicated SEC fractions.

b, Representative cryo-EM micrograph of the apo *At*AMT1;1$_{WT}$ at pH 7.4. Representative particles are indicated by blue circles.

c, Representative 2D classification images of the apo *At*AMT1;1$_{WT}$ at pH 7.4.

d, Cryo-EM data-processing workflows for apo *At*AMT1;1$_{WT}$ at pH 7.4. The local-resolution map of the final reconstruction is shown below.

e, Angular distribution of particles contributing to the final reconstruction.

f, Gold-standard Fourier shell correlation (GSFSC) curves of the final reconstruction. The overall resolution was estimated to be 2.9 Å using the FSC = 0.143 criterion.

a

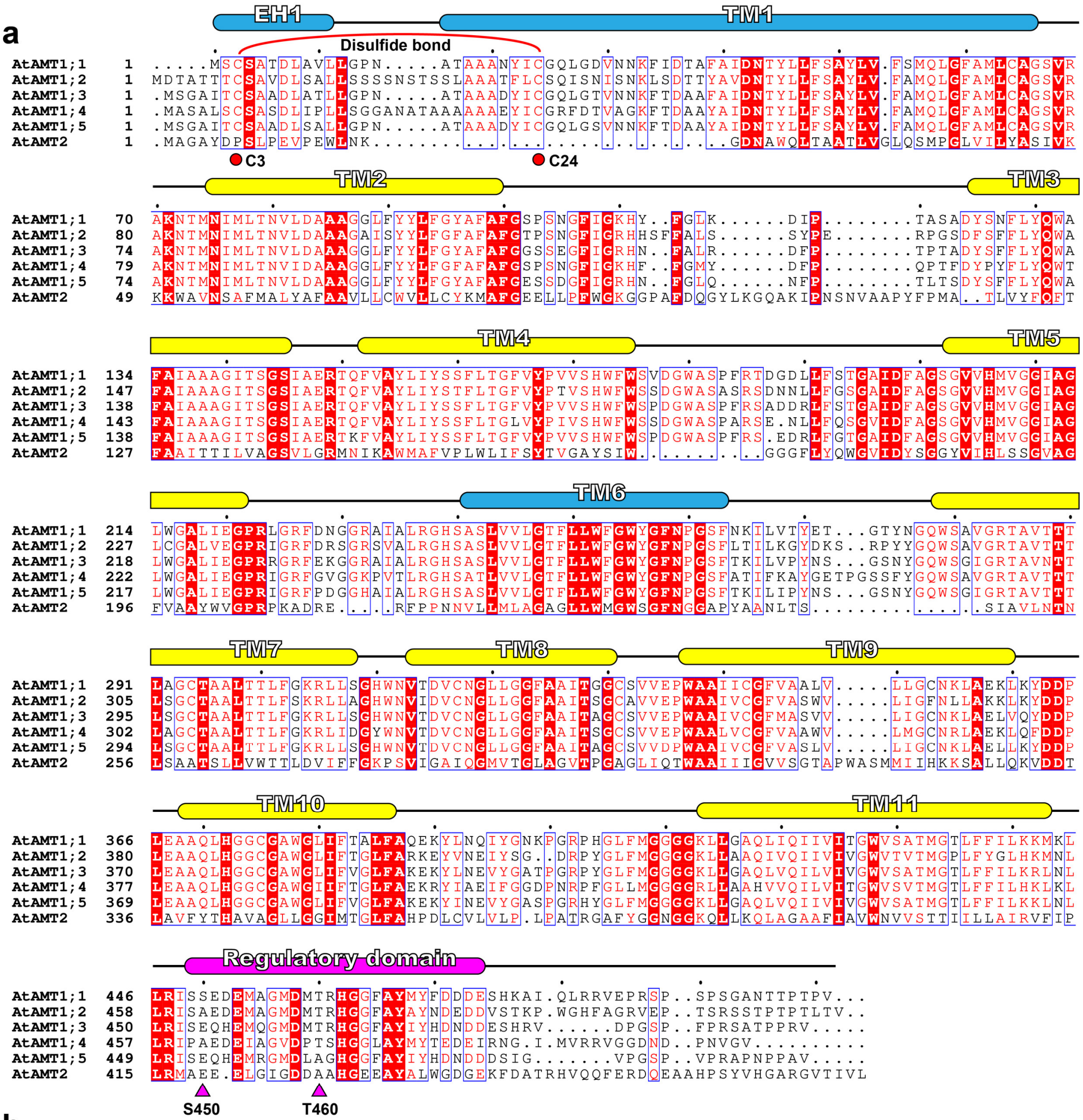

b

	*At*AMT1;1	*At*AMT1;2	*At*AMT1;3	*At*AMT1;4	*At*AMT1;5	*At*AMT2
***At*AMT1;1**	—	73.9%	82.7%	75.6%	81.9%	27.9%
***At*AMT1;2**	—	—	74.1%	71.4%	74.7%	25.6%
***At*AMT1;3**	—	—	—	73.5%	93.3%	27.7%
***At*AMT1;4**	—	—	—	—	73.6%	26.7%
***At*AMT1;5**	—	—	—	—	—	27.8%

Supplementary Fig. 2 Sequence comparison of the *Arabidopsis thaliana* AMT family.

a, Sequence alignments of the six *Arabidopsis thaliana* AMTs proteins. The amino acid sequences are available at UniProt (https://www.uniprot.org) with the following accession codes: *At*AMT1;1: P54144, *At*AMT1;2: Q9ZPJ8, *At*AMT1;3: Q9SQH9, *At*AMT1;4: Q9SVT8, *At*AMT1;5: Q9LK16 and *At*AMT2: Q9M6N7. Sequences were aligned using ClustalW and visualized using ESPript 3.0. Secondary structural elements

of *At*AMT1;1 are indicated above the sequence. Invariant amino acids are shaded red. The cysteine residues forming the disulfide bond and the putative phosphorylation sites are indicated by red circles and purple triangles, respectively.

b, Amino acid sequence identities between the six AMTs in *Arabidopsis thaliana*.

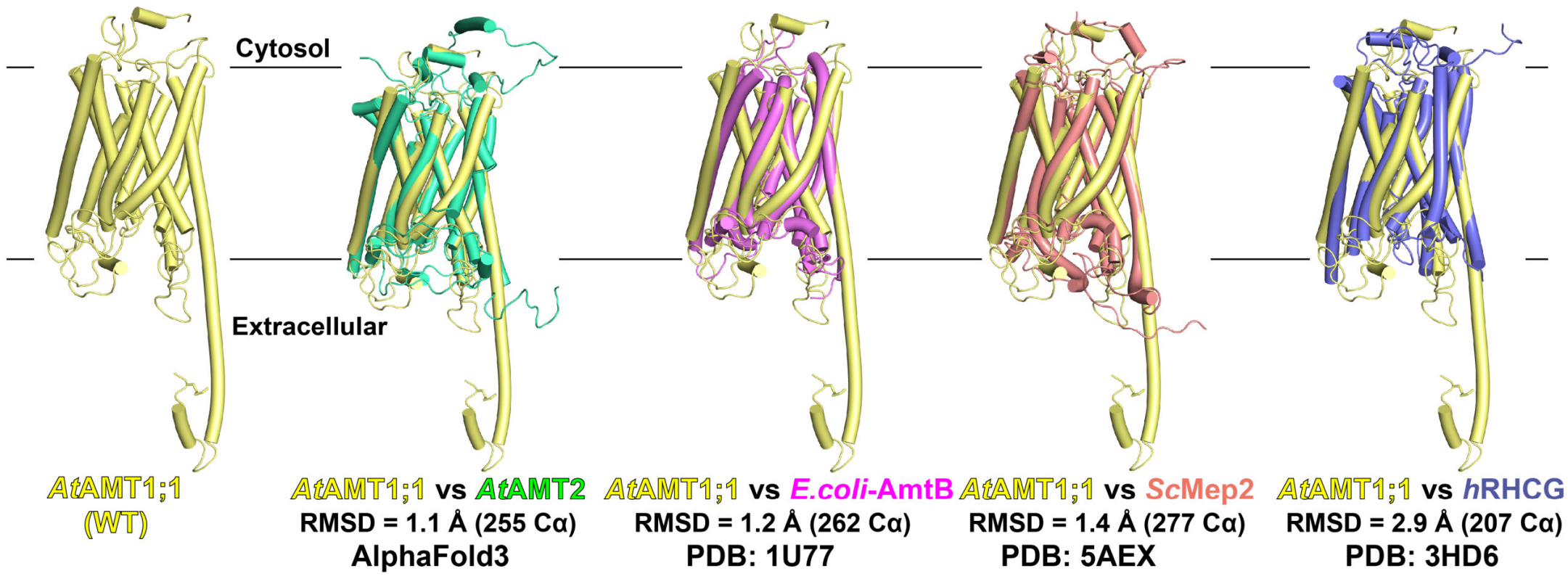

Supplementary Fig. 3 Structural comparison between *At*AMT1;1 and orthologs. Structural superposition of *At*AMT1;1 with *At*AMT2 (AlphaFold 3 prediction), *E. coli* AmtB (PDB: 1U77), *S. cerevisiae* Mep2 (PDB: 5AEX) and human RHCG (PDB: 3HD6). The corresponding root mean square deviation (RMSD) values are 1.1 Å (255 Cα atoms), 1.2 Å (262 Cα atoms), 1.4 Å (277 Cα atoms) and 2.9 Å (207 Cα atoms), respectively.

a

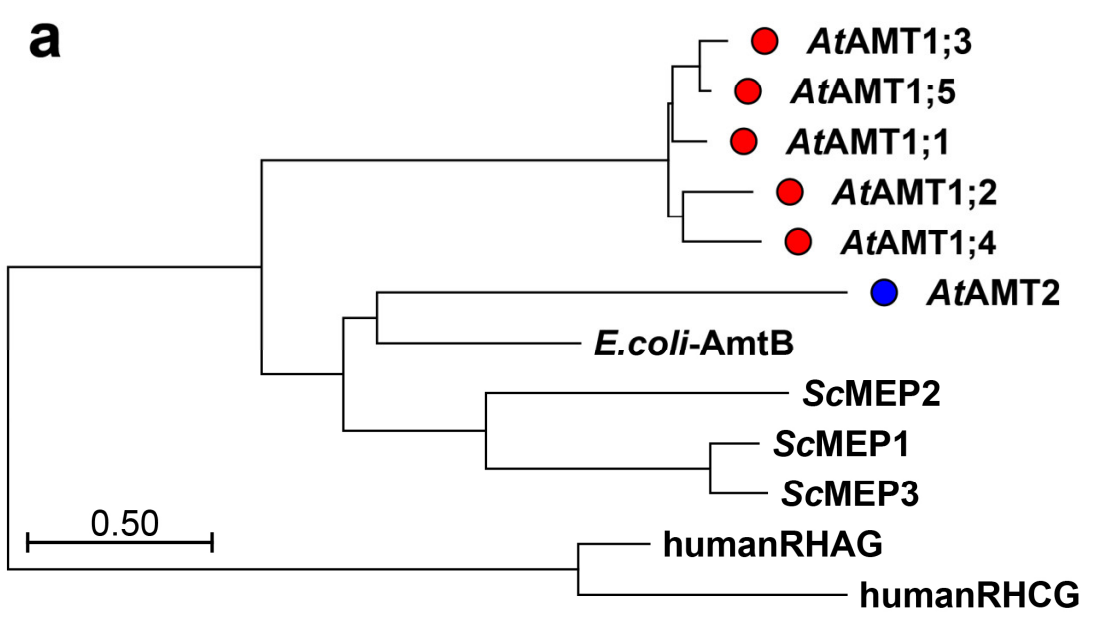

b

	*At*AMT1;1	*E.coli*-AmtB	*Sc*MEP2	humanRHCG
***At*AMT1;1**	—	31.2%	27.6%	20.4%
***E.coli*-AmtB**	—	—	34.6%	22.6%
***Sc*MEP2**	—	—	—	22.8%

c

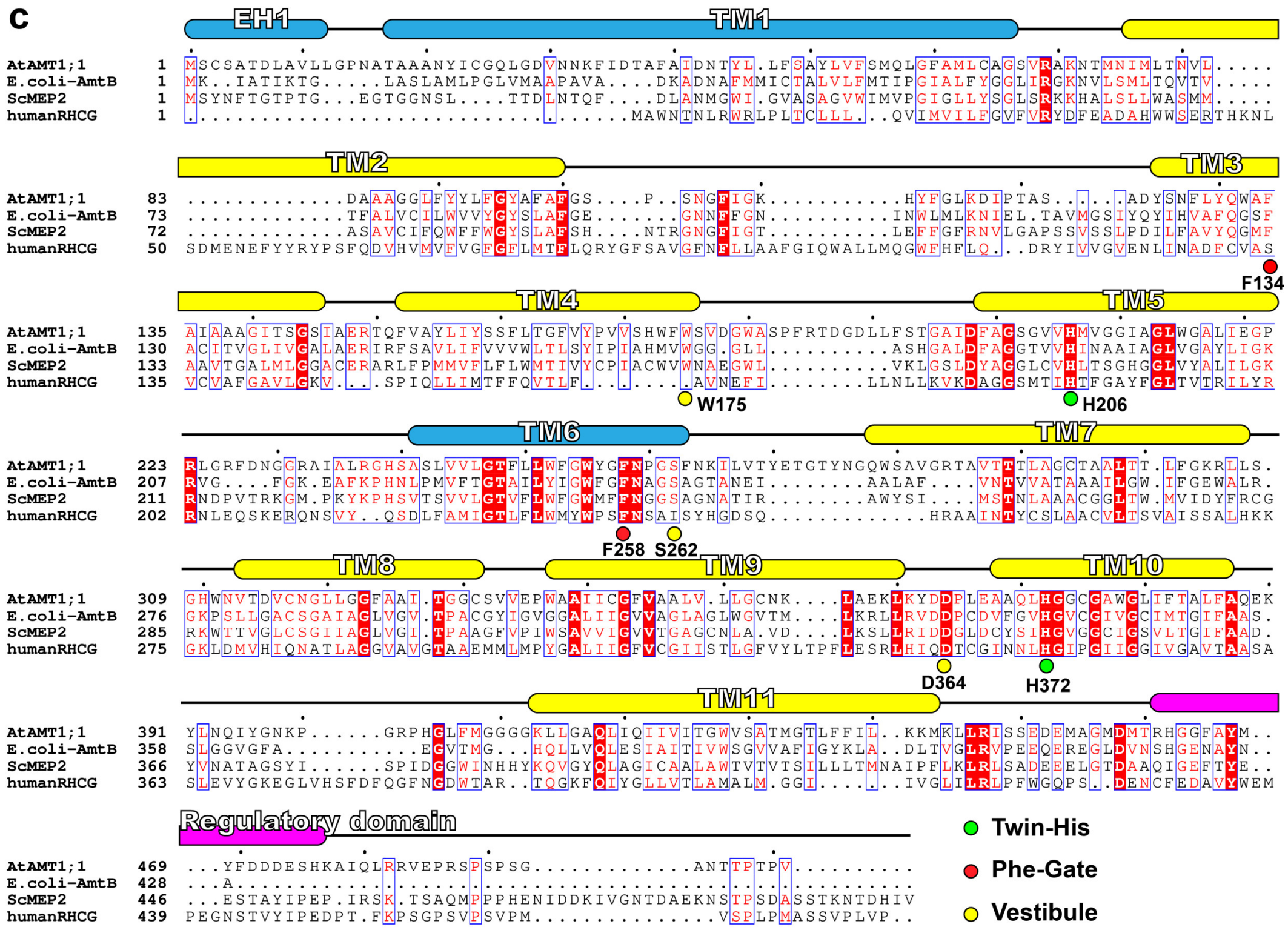

Supplementary Fig. 4 Sequence alignments of *At*AMT1;1 with orthologs.

a, b, Phylogenetic analysis **(a)** and amino acid sequence identities **(b)** among *At*AMTs and selected orthologs, including *E. coli* AmtB protein, *S. cerevisiae* MEP protein, and human RHCG protein. The amino acid sequences are available at UniProt (https://www.uniprot.org) with the following accession codes: *At*AMT1;1: P54144, *At*AMT1;2: Q9ZPJ8, *At*AMT1;3: Q9SQH9, *At*AMT1;4: Q9SVT8, *At*AMT1;5: Q9LK16, *At*AMT2: Q9M6N7, *Ec*AmtB: P69681, *Sc*MEP1: P40260, *Sc*MEP2: P41948, *Sc*MEP3: P53390, *h*RHAG: Q02094 and *h*RHCG: Q9UBD6. Phylogenetic analysis was performed using MEGA11 (v.11.0.13).

c, Sequence alignment of *At*AMT1;1 and its orthologs. Sequences were aligned using ClustalW and visualized using ESPript 3.0. Secondary structural elements of

*At*AMT1;1 are indicated above the sequence. Invariant amino acids are shaded red. Residues forming the Twin-His motif, Phe-gate and extracellular vestibule are indicated by green, red and yellow circles, respectively.

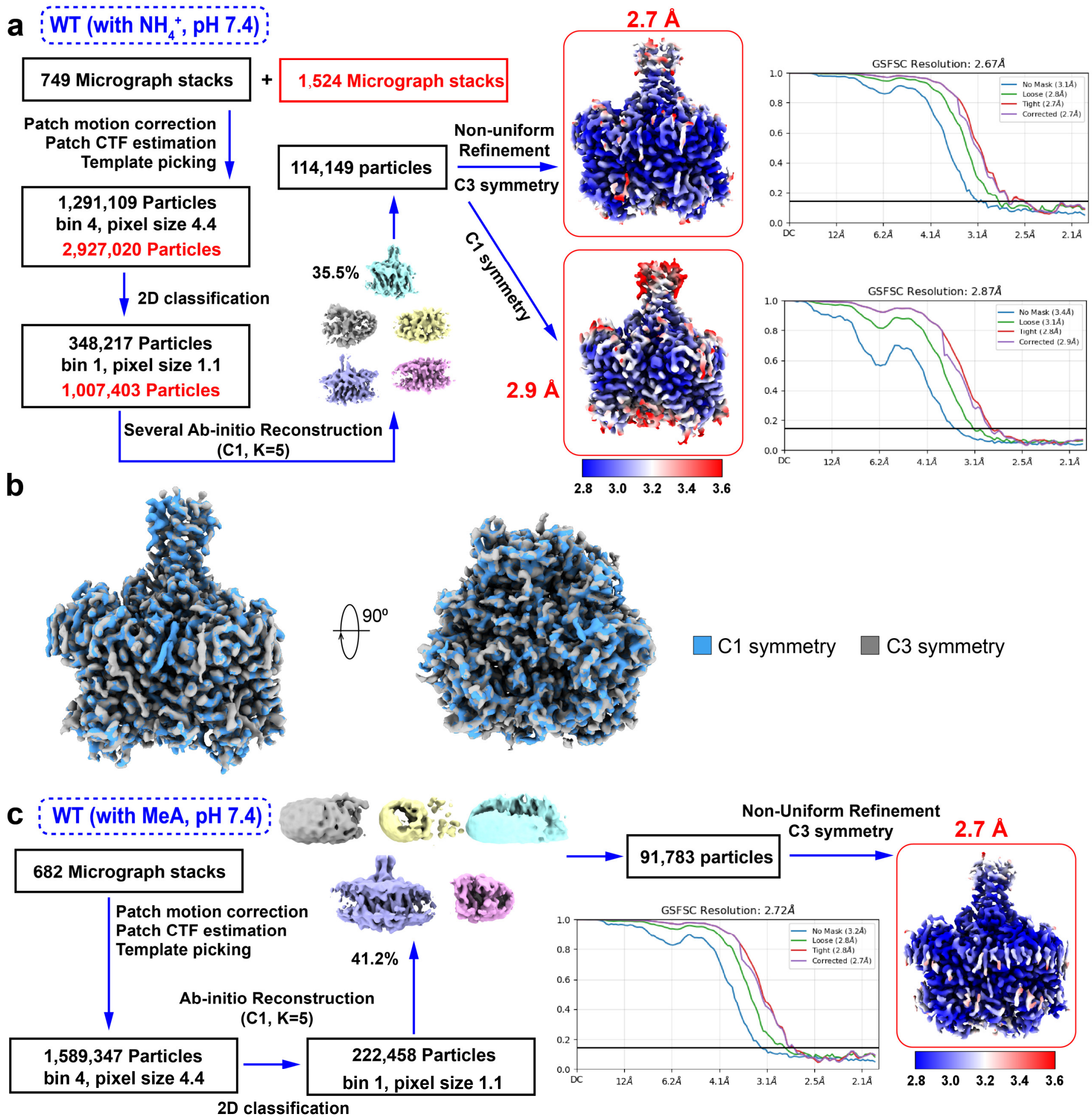

Supplementary Fig. 5 Cryo-EM analysis of WT *At*AMT1;1 in different states.

a, c, A Cryo-EM data-processing workflows for *At*AMT1;1_{WT} in the presence of 1 mM ammonium sulfate (pH 7.4) **(a)** and *At*AMT1;1_{WT} in the presence of 10 mM MeA (pH 7.4) **(c)**. Representative micrographs, 2D class averages, local-resolution maps, particle angular distributions and GSFSC curves are shown. The final reconstructions were obtained at overall resolutions of 2.7 Å for the NH_4^+-supplemented structure with C3 symmetry, 2.9 Å for the NH_4^+-supplemented structure with C1 symmetry and 2.7 Å for the MeA-supplemented structure with C3 symmetry, using the FSC = 0.143 criterion.

b, Comparison of the cryo-EM maps of NH_4^+-supplemented WT *At*AMT1;1 reconstructed with C1 (blue) and C3 (grey) symmetry in two orthogonal views.

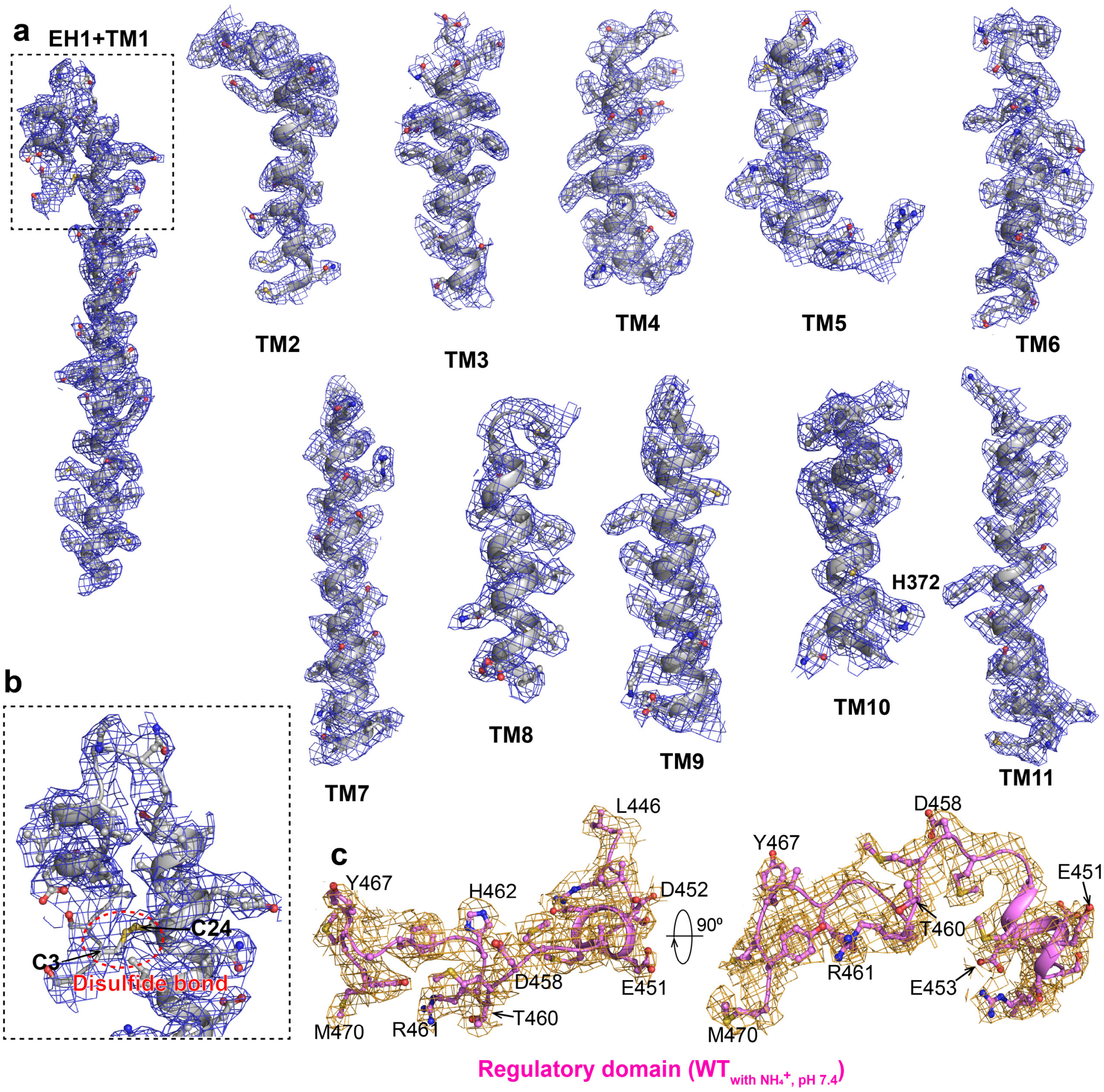

Supplementary Fig. 6 Cryo-EM densities of the transmembrane, N-terminal and regulatory regions of *At*AMT1;1$_{WT}$ at pH 7.4.

a, Cryo-EM densities for the eleven transmembrane helices of the apo *At*AMT1;1$_{WT}$ at pH 7.4.

b, Cryo-EM density of the EH1 region of the apo *At*AMT1;1$_{WT}$ at pH 7.4. The disulfide bond is highlighted with a red dashed circle.

c, Cryo-EM densities for the regulatory domain of the *At*AMT1;1$_{WT}$ (with NH_4^+, pH 7.4). Residues with notable side chains are labelled aside.

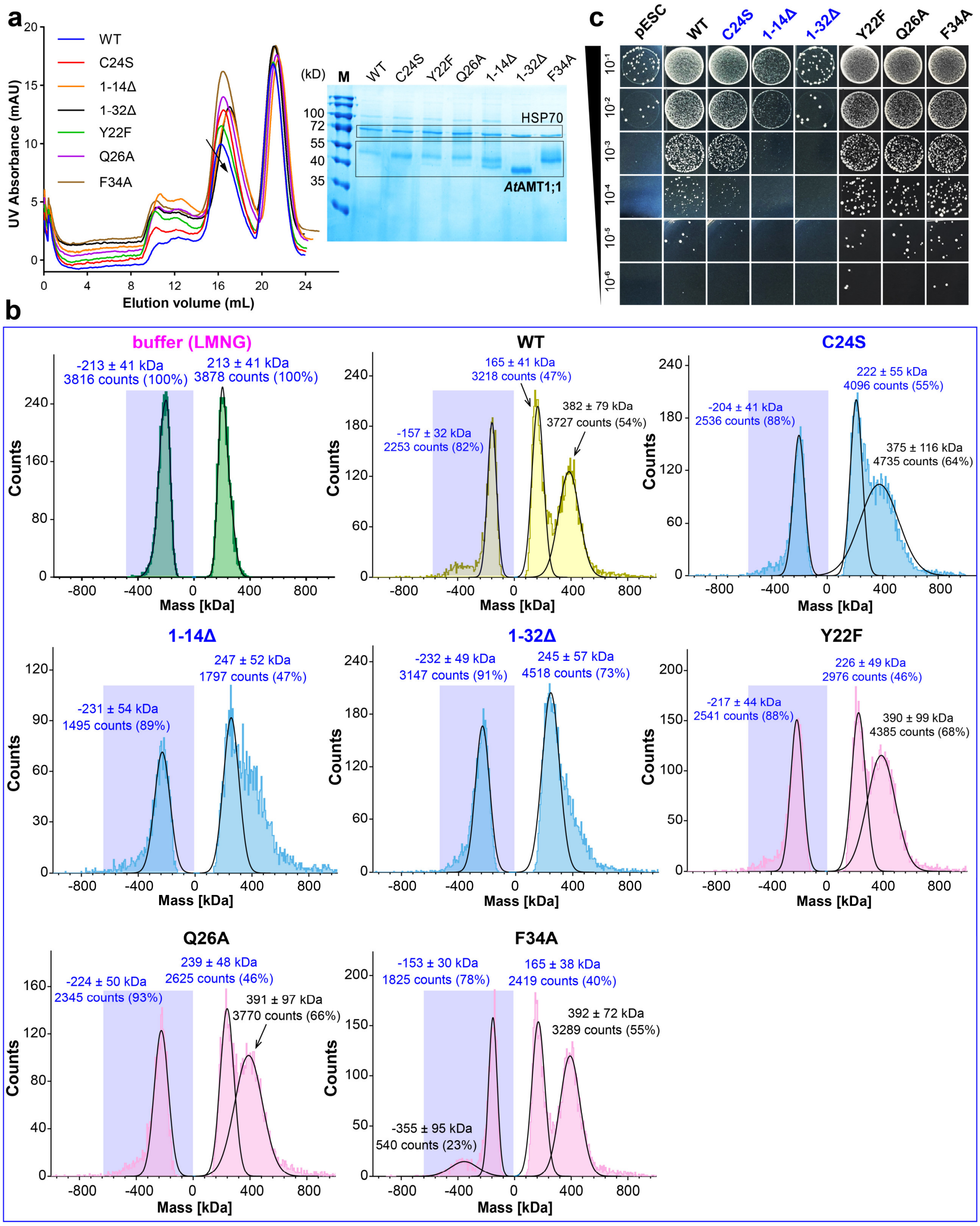

Supplementary Fig. 7 The N-terminal region stabilizes the oligomeric assembly of *At*AMT1;1 revealed by biochemical and mass photometry analyses.

a, Representative SEC profiles and Coomassie blue-stained SDS–PAGE analyses of WT *At*AMT1;1 and the indicated N-terminal mutants and truncation variants.

b, Complete positive and negative mass photometry distributions of LMNG detergent control, WT *At*AMT1;1, C24S mutant, and N-terminal truncation mutants. Symmetric positive and negative signals observed in detergent control and the first WT population

indicate detergent micelle-associated background, whereas the major WT protein-associated population lacks a corresponding negative signal, consistent with a stable oligomeric assembly.

c, Growth of the yeast strain 31019b transformed with the N-terminal motif mutants of *At*AMT1;1 in YNB-N medium supplemented with 0.5 mM $(NH_4)_2SO_4$ at pH 5.5 and incubated at 30 °C for 3 days.

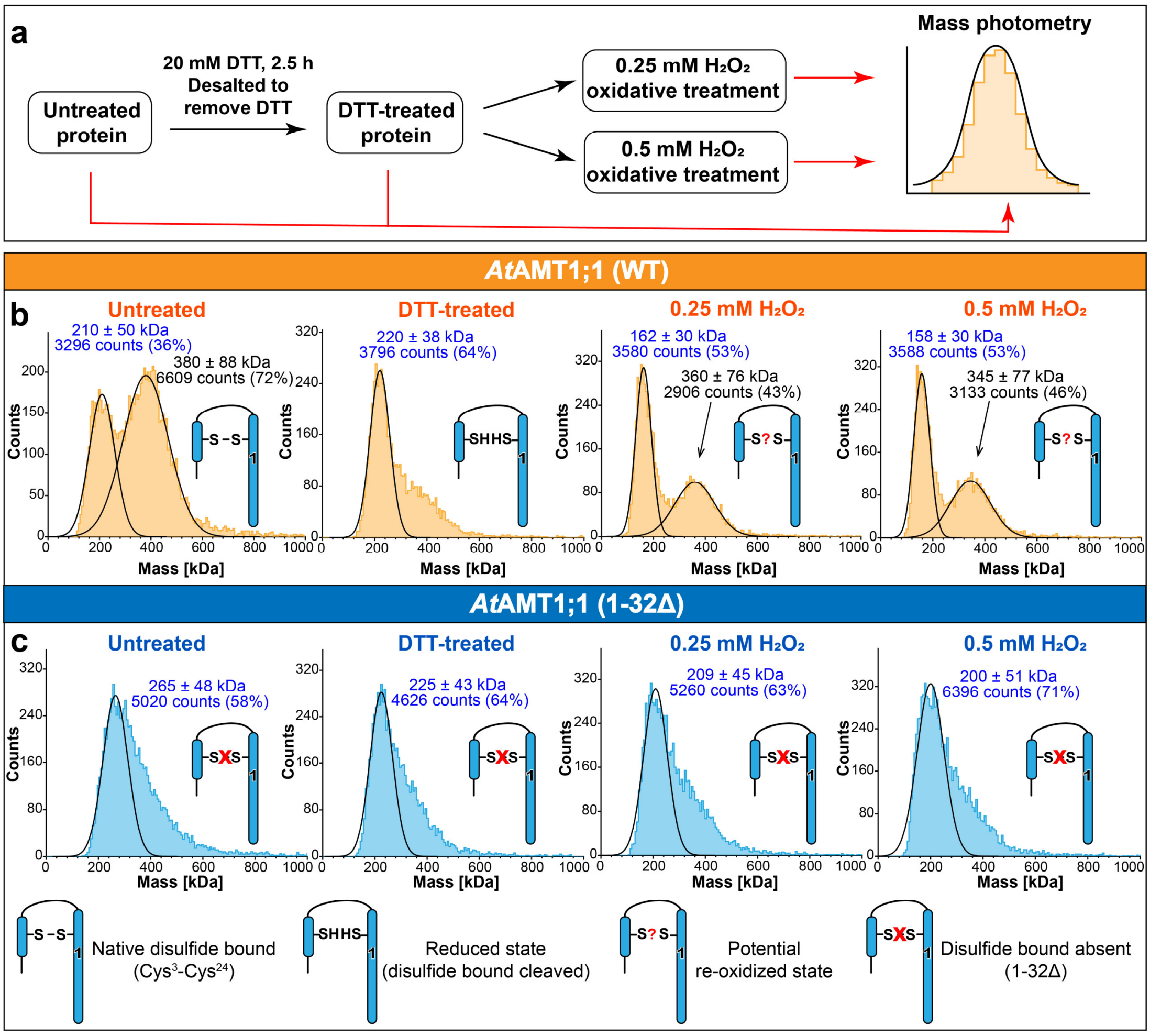

Supplementary Fig. 8 The N-terminal disulfide bond contributes to redox-dependent stabilization of the *At*AMT1;1 oligomeric assembly.

a, Schematic of the sequential redox treatment used for mass photometry analysis. Purified protein was treated with 20 mM DTT for 2.5 hours, followed by desalting to remove residual DTT. The desalted protein was subsequently subjected to oxidative treatment with 0.25 or 0.5 mM H_2O_2 before mass photometry analysis.

b, Mass photometry analysis of WT *At*AMT1;1 under untreated, DTT-treated, and subsequent H_2O_2-treated conditions. DTT treatment markedly reduced the high-molecular-weight protein-associated population, whereas subsequent treatment with 0.25 or 0.5 mM H_2O_2 partially restored this population.

c, Corresponding mass photometry analysis of the N-terminal truncation variant *At*AMT1;1 (1–32Δ). No apparent redox-dependent redistribution of the mass population was observed following DTT treatment or subsequent H_2O_2 treatment.

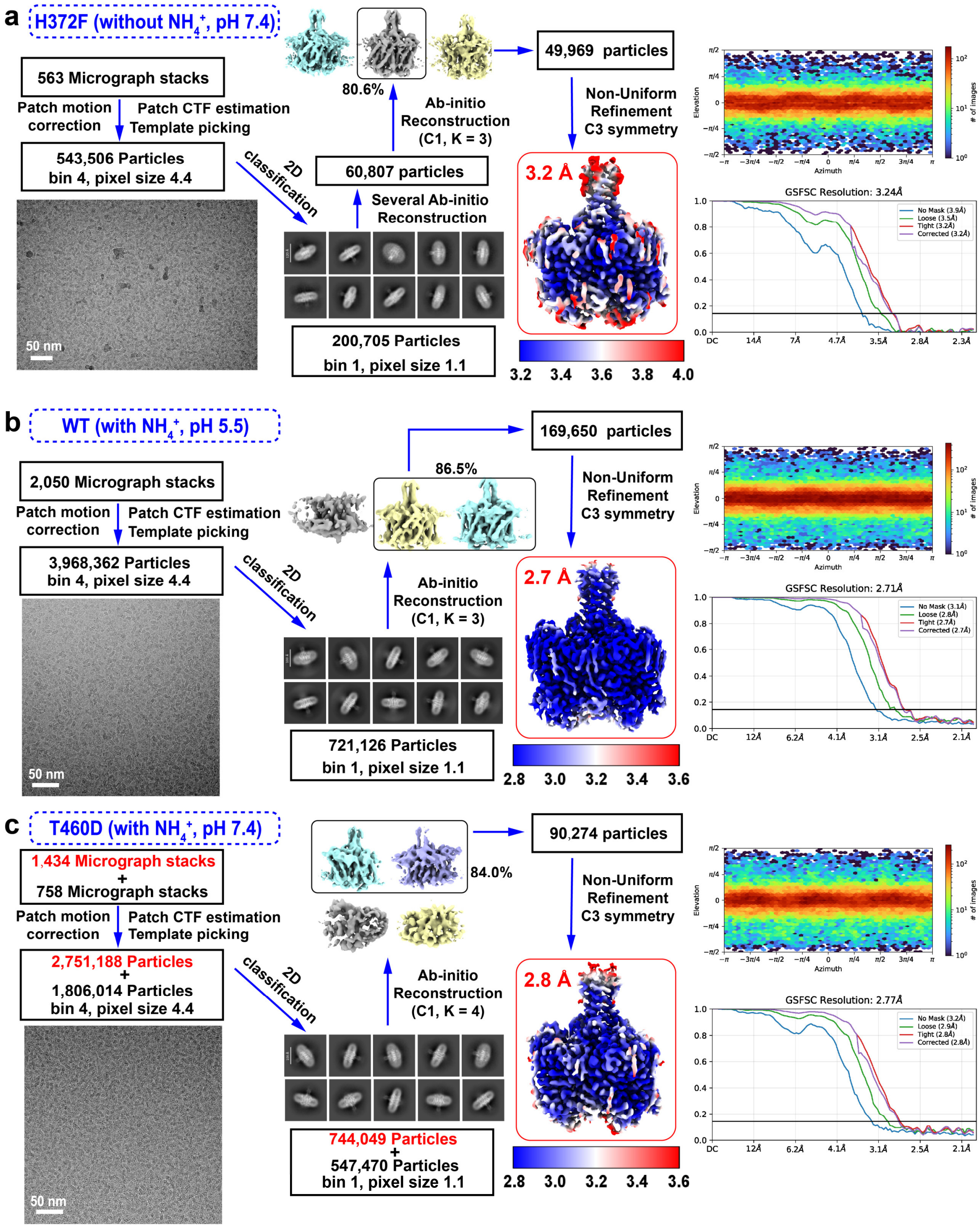

Supplementary Fig. 9 Cryo-EM data-processing of WT and mutant *At*AMT1;1.

a-c, Cryo-EM data-processing workflows for apo *At*AMT1;1$_{H372F}$ at pH 7.4 **(a)**, NH_4^+-supplemented *At*AMT1;1$_{WT}$ at pH 5.5 **(b)** and NH_4^+-supplemented *At*AMT1;1$_{T460D}$ at pH 7.4 **(c)**. Representative micrographs, 2D class averages, local-resolution maps, particle angular distributions and GSFSC curves are shown. The final reconstructions were obtained at overall resolutions of 3.2 Å, 2.7 Å and 2.8 Å, respectively, using the FSC = 0.143 criterion.

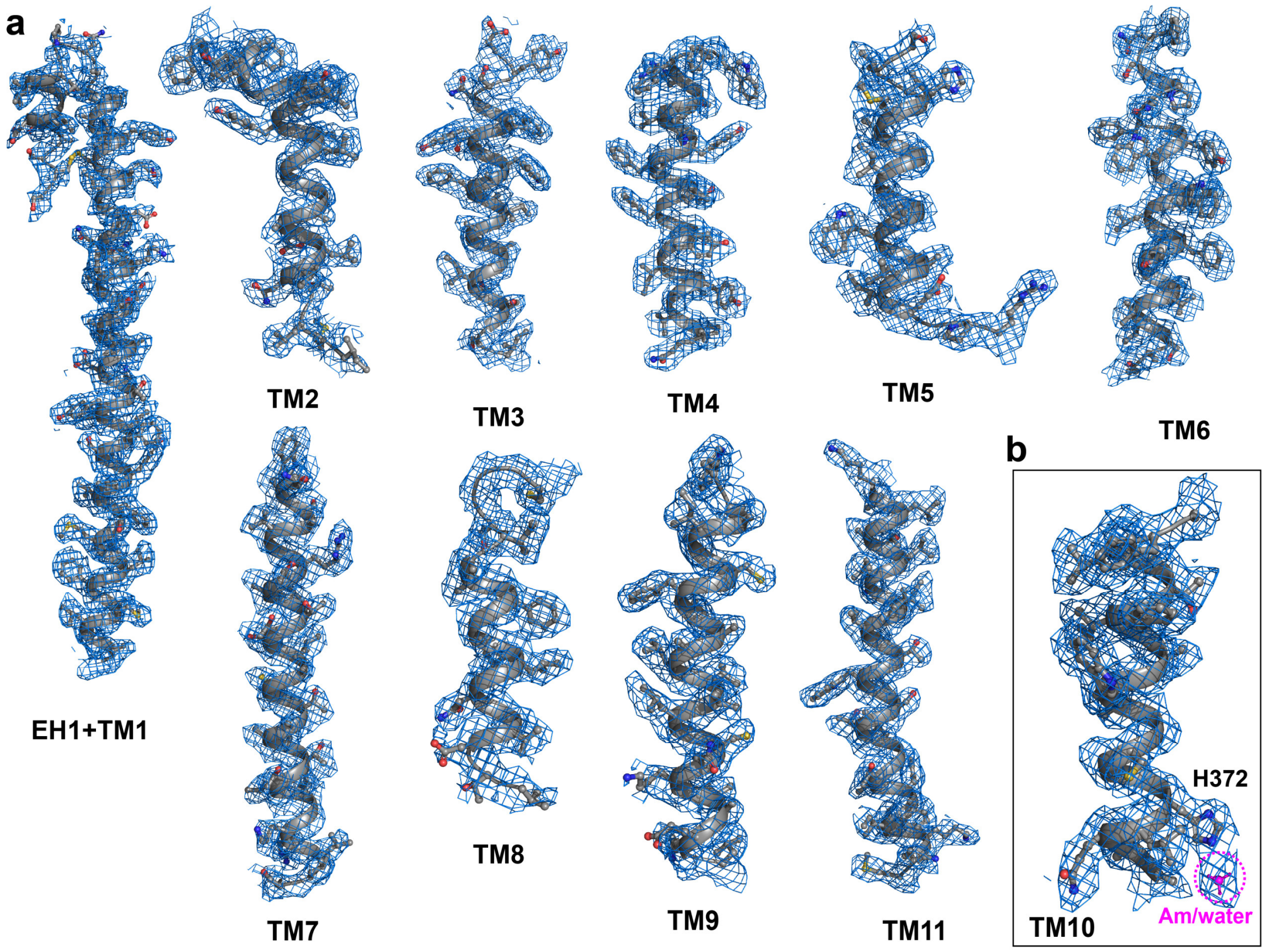

Supplementary Fig. 10 Cryo-EM densities of the transmembrane region of *At*AMT1;1$_{\mathrm{WT}}$ at pH 5.5.

a, Cryo-EM densities of all transmembrane helices except TM10.

b, Cryo-EM density of TM10. Selected side chains are labelled. The unassigned density adjacent to H372 is indicated by the magenta dashed circle and may represent Am or an ordered water molecule.

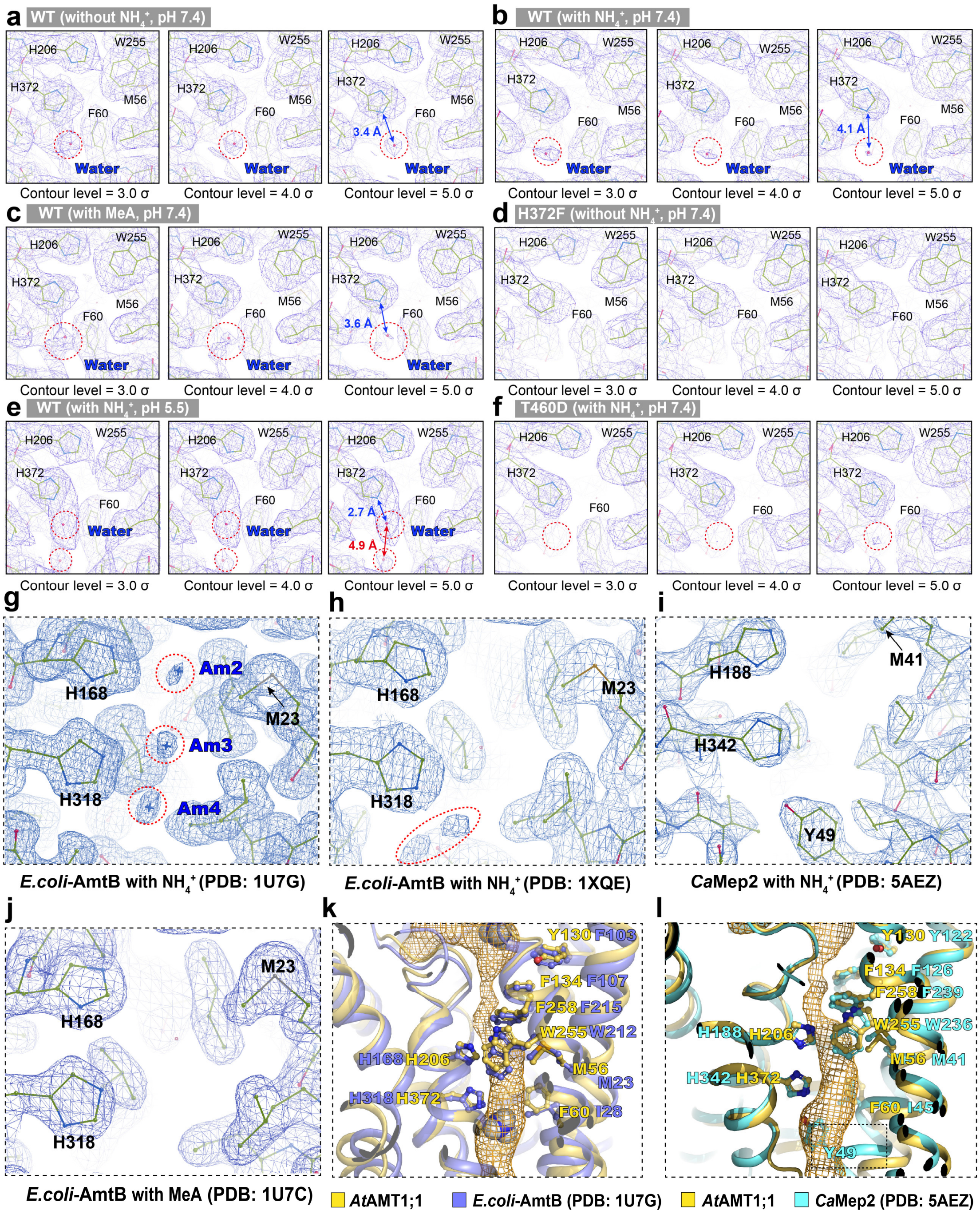

Supplementary Fig. 11 Comparison of non-protein densities near the conserved Twin-His region in AMT-family transporters.

a-f, Cryo-EM densities observed adjacent to H372 in *At*AMT1;1 structures determined under different conditions. Shown are WT (without NH_4^+, pH 7.4) **(a)**, WT (with NH_4^+, pH 7.4) **(b)**, WT (with MeA, pH 7.4) **(c)**, H372F (without NH_4^+, pH 7.4) **(d)**, WT (with NH_4^+, pH 5.5) **(e)**, and T460D (with NH_4^+, pH 7.4) **(f)**. A non-protein density is

consistently observed adjacent to H372 in multiple *At*AMT1;1 structures but is absent in the H372F mutant. Maps are shown at the indicated contour levels.

g–j, Previously reported substrate-associated densities near the conserved Twin-His motif in prokaryotic and fungal ammonium transporters. Shown are *E. coli* AmtB in the presence of NH_4^+ (PDB: 1U7G) **(g)**, *E. coli* AmtB in the presence of NH_4^+ (PDB: 1XQE) **(h)**, *Candida albicans* Mep2 (PDB: 5AEZ) **(i)** and *E. coli* AmtB in the presence of methylammonium (PDB: 1U7C) **(j)**. Non-protein densities previously assigned as substrate molecules are shown as mesh.

k, l, Structural superposition of the pore regions of *At*AMT1;1 with *E. coli* AmtB **(k)** and *C. albicans* Mep2 **(l)**. Conserved residues lining the pore are shown as sticks. In *Ca*Mep2, the residue Y49 (highlighted by a black dashed box) partially occludes the pore adjacent to the conserved histidine pair.

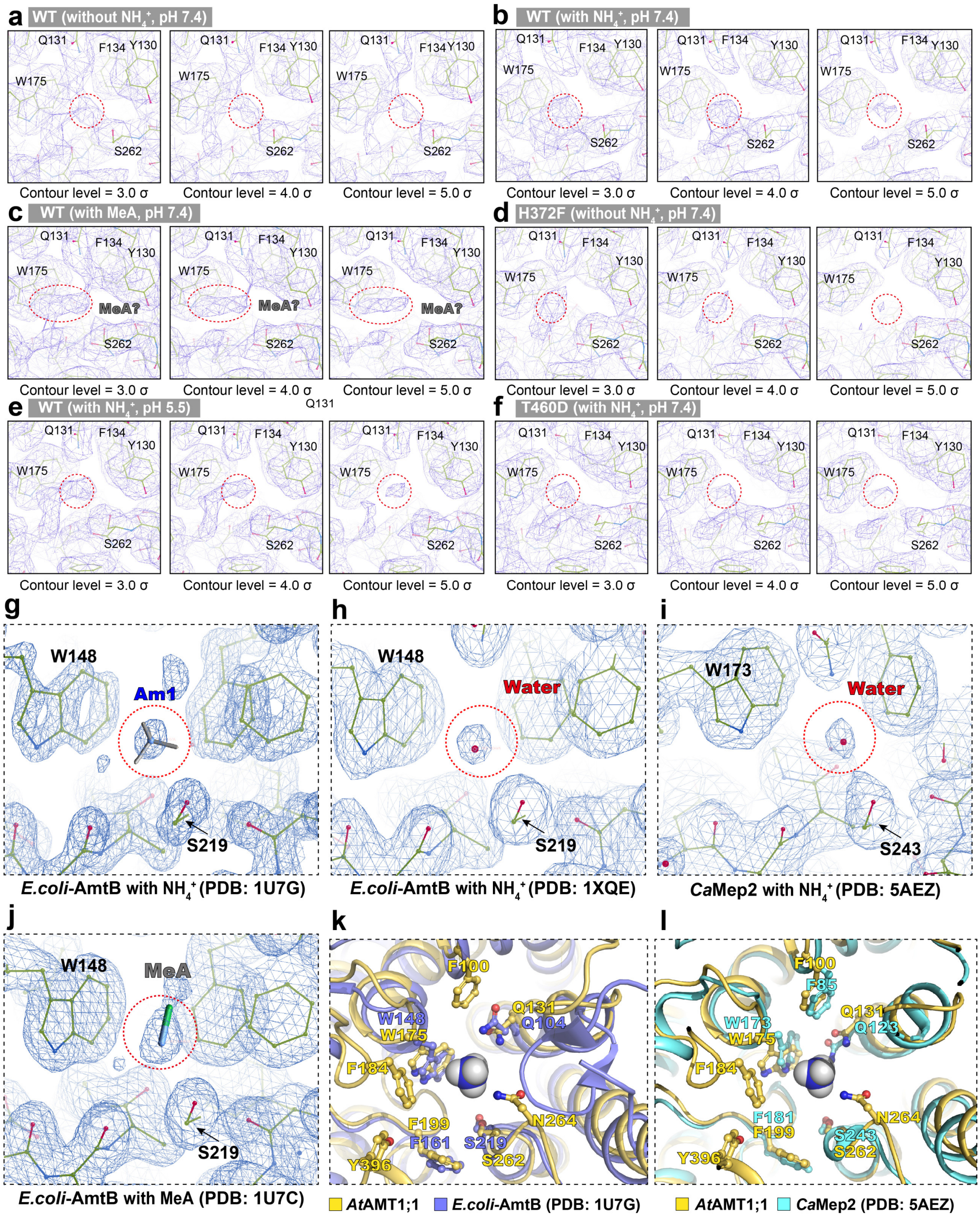

Supplementary Fig. 12 Comparison of non-protein densities at the extracellular recruitment site in AMT-family transporters.

a-f, Cryo-EM densities observed near the extracellular recruitment site surrounding W175 in *At*AMT1;1 structures determined under different conditions. Shown are WT (without NH_4^+, pH 7.4) **(a)**, WT (with NH_4^+, pH 7.4) **(b)**, WT (with MeA, pH 7.4) **(c)**, H372F (without NH_4^+, pH 7.4) **(d)**, WT (with NH_4^+, pH 5.5) **(e)** and T460D (with NH_4^+, pH 7.4) **(f)**. A non-protein density is consistently observed adjacent to W175 and

neighboring residues that form the extracellular vestibule. Maps are shown at the indicated contour levels.

g–j, Previously reported densities observed at the extracellular substrate recruitment region in AMT-family transporters. Shown are *E. coli* AmtB in the presence of NH_4^+ (PDB: 1U7G) **(g)**, *E. coli* AmtB in the presence of NH_4^+ (PDB: 1XQE) **(h)**, *Candida albicans* Mep2 (PDB: 5AEZ) **(i)** and *E. coli* AmtB in the presence of methylammonium (PDB: 1U7C) **(j)**. Similar densities have previously been interpreted as substrate molecules or ordered water molecules associated with the extracellular recruitment site.

k, l, Structural superposition of the extracellular vestibule of *At*AMT1;1 with *E. coli* AmtB **(k)** and *C. albicans* Mep2 **(l)**. Conserved residues surrounding the extracellular recruitment site are shown as sticks.

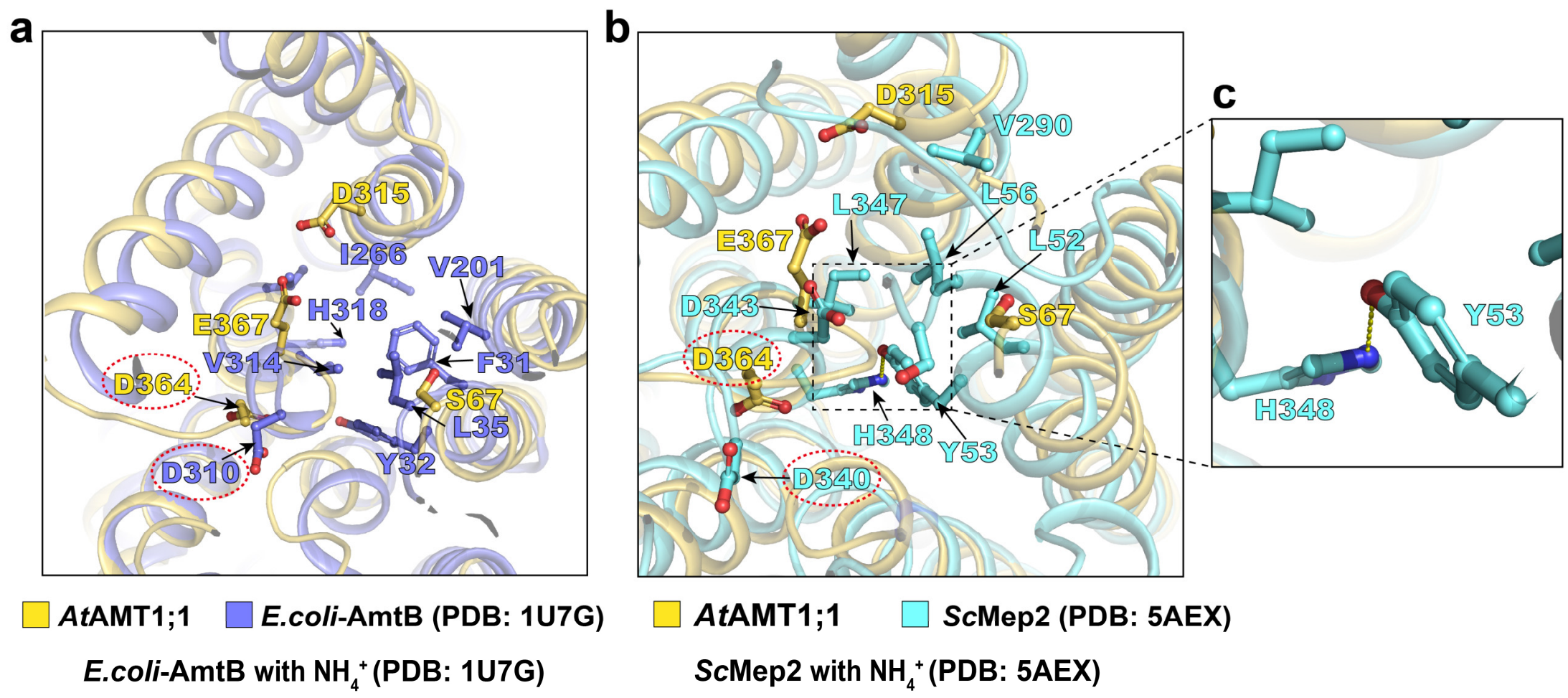

Supplementary Fig. 13 Structural comparison of the cytosolic exit region among AMT-family transporters.

a, Structural superposition of the cytosolic exit region of *At*AMT1;1 and *E. coli* AmtB in the presence of NH_4^+ (PDB: 1U7G).

b, Structural superposition of the cytosolic exit region of *At*AMT1;1 and *Saccharomyces cerevisiae* Mep2 (*Sc*Mep2, PDB: 5AEX). For panels **a** and **b**, residues lining the cytosolic exit pathway are shown as sticks, and conserved glutamate residues along the putative substrate exit route are highlighted by red dashed circles.

c, Enlarged view of the cytosolic exit region in *Sc*Mep2. Residue Y53 forms a hydrogen-bond interaction with the conserved Twin-His residue H348, partially occluding the pore and narrowing the cytosolic exit pathway.

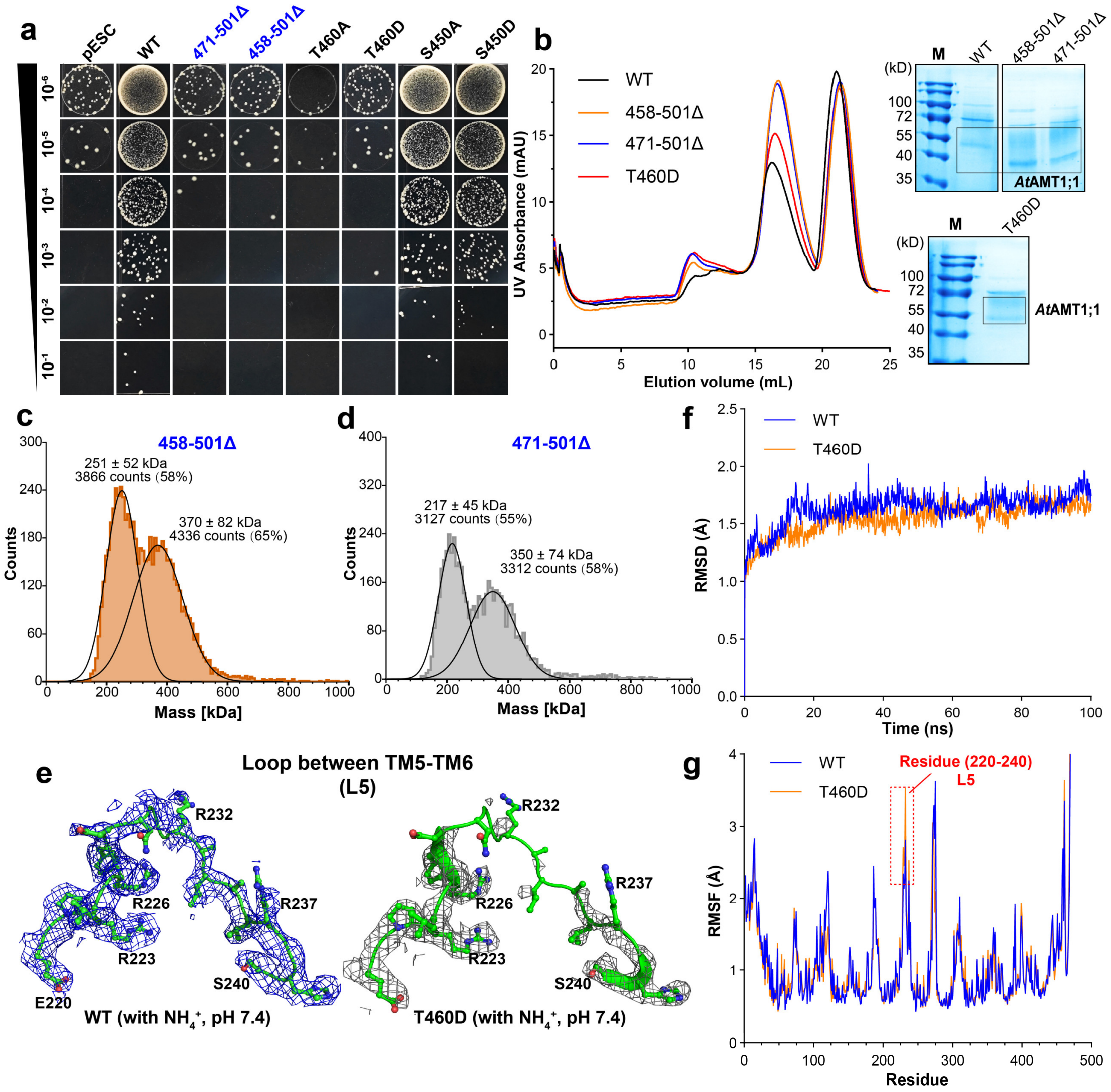

Supplementary Fig. 14 Structural and functional analysis of the C-terminal regulatory domain of *At*AMT1;1.

a, Growth of the ammonium transporter-deficient yeast strain 31019b expressing the indicated constructs was examined on YNB-N medium supplemented with 0.5 mM $(NH_4)_2SO_4$ at pH 5.5 and incubated at 30 °C for 3 days.

b, Representative SEC profiles and Coomassie blue-stained SDS–PAGE analyses of purified WT *At*AMT1;1, the phosphomimetic mutant T460D, and the C-terminal truncation variants (458–501Δ and 471–501Δ).

c, d, Mass photometry analysis of the 458–501Δ **(c)** and 471–501Δ **(d)** truncation variants. Molecular mass distributions were determined under identical detergent conditions to assess the effects of C-terminal truncation on oligomeric stability and sample homogeneity.

e, Cryo-EM densities corresponding to intracellular loop L5 in the WT and T460D structures.

f, g, RMSD (**f**) and root mean square fluctuation (RMSF) (**g**) profiles of WT and T460D *At*AMT1;1. The curves are means from three independent 100-ns simulations. The region corresponding to residues 220–240 in loop L5 is highlighted in **g**.

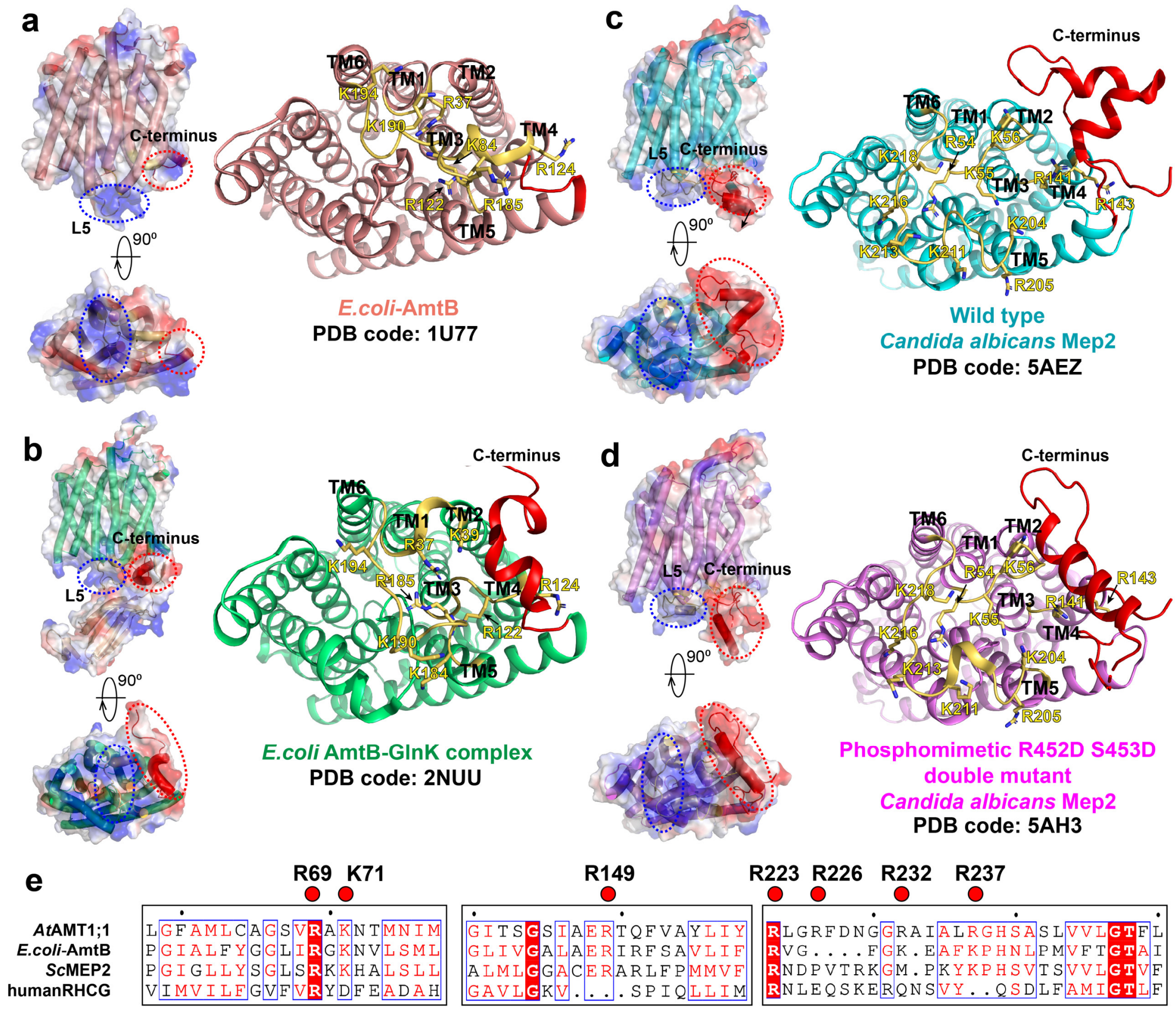

Supplementary Fig. 15 Structural features of intracellular loops and C-terminal regions.

a-d, The structural features of intracellular loops and C-terminal regulatory regions in representative AMT-family transporters. Shown are *E. coli* AmtB (PDB: 1U77) **(a)**, the inhibited *E. coli* AmtB–GlnK complex (PDB: 2NUU) **(b)**, *Ca*Mep2 (PDB: 5AEZ) **(c)** and the phosphomimetic mutant R452D/S453D of *Ca*Mep2 (PDB: 5AH3) **(d)**. In each panel, the left view shows the electrostatic surface representation of the transporter. The intracellular loops and the C-terminal regulatory region are highlighted by blue and red dashed circles, respectively. The right view shows enlarged structural details, with intracellular loops coloured orange and the C-terminal region coloured red.

e, Sequence alignment of intracellular loops L1, L3, and L5 from *At*AMT1;1, *E. coli* AmtB, *S. cerevisiae* Mep2, and human RhCG. Conserved positively charged residues are highlighted by red circles. The enrichment of lysine and arginine residues within

these intracellular loops suggests that positively charged loop elements are broadly conserved across members of the AMT/MEP/Rh superfamily.

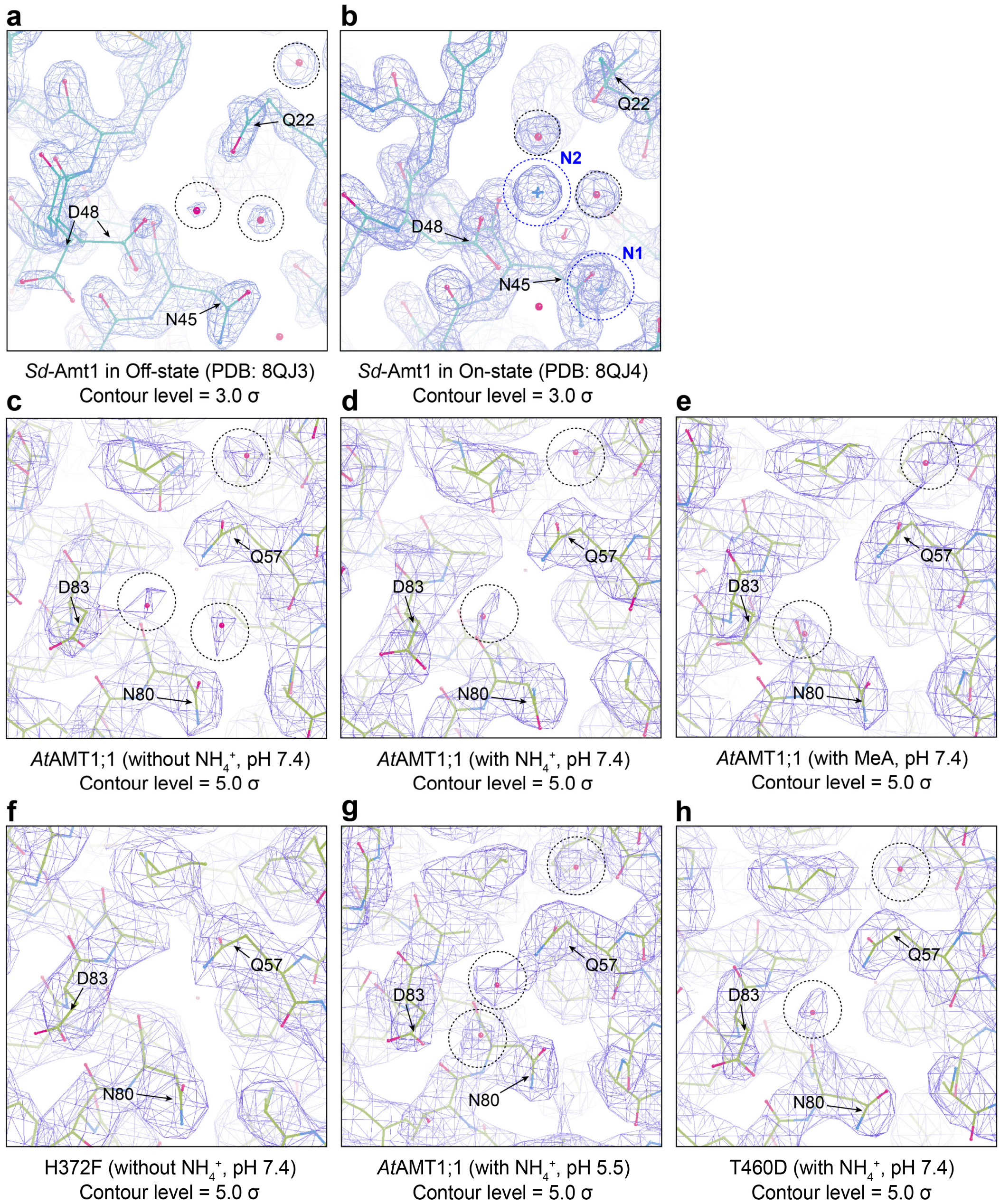

Supplementary Fig. 16 Non-protein densities in the N1/N2-associated region of *Sd*-Amt1 and *At*AMT1;1.

a, b, Previously reported densities around the N1 and N2 sites in the off-state **(a)** and on-state **(b)** structures of *Shewanella denitrificans* Amt1 (*Sd*-Amt1), respectively.

c-h, Cryo-EM density maps of the corresponding N1/N2-equivalent region in six structural states of *At*AMT1;1 determined under different conditions. Shown are WT (without NH_4^+, pH 7.4) **(c)**, WT (with NH_4^+, pH 7.4) **(d)**, WT (with MeA, pH 7.4) **(e)**, H372F (without NH_4^+, pH 7.4) **(f)**, WT (with NH_4^+, pH 5.5) **(g)** and T460D (with NH_4^+, pH 7.4) **(h)**.

Supplementary Table 1 Statistics of cryo-EM data collection, processing, model refinement and validation.

Data Collection	WT (pH 7.4) PDB: 22MG EMD-68466	WT (with NH_4^+, pH 7.4) PDB: 22NK EMD-68512	WT (with MeA, pH 7.4) PDB: 22OY EMD-68580	H372F (pH 7.4) PDB: 22NG EMD-68504	WT (with NH_4^+, pH 5.5) PDB: 27IF EMD-81185	T460D (with NH_4^+, pH 7.4) PDB: 22NN EMD-68515
EM equipment	Titan Krios	Titan Krios	Titan Krios	Titan Krios	Titan Krios	Titan Krios
Voltage (kV)	300	300	300	300	300	300
Detector	BioQuantum K3	Falcon 4i	Falcon 4i	BioQuantum K3	Falcon 4i	Falcon 4i
Pixel size (Å)	1.1	0.971	0.971	1.1	0.971	0.971
Electron dose (e^-/Å^2)	50	50	50	50	50	50
Defocus range (µm)	-1.0 ~ -2.0	-1.0 ~ -2.0	-1.0 ~ -2.0	-1.0 ~ -2.0	-1.0 ~ -2.0	-1.0 ~ -2.0
**3D Reconstruction**						
Software	CryoSPARC v.4.6.2	CryoSPARC v.4.6.2	CryoSPARC v.4.6.2	CryoSPARC v.4.6.2	CryoSPARC v.4.6.2	CryoSPARC v.4.6.2
Number of Particles	65,843	114,149	91,783	49,969	169,650	90,274
Symmetry	C3	C3	C3	C3	C3	C3
Map Resolution at 0.143 FSC cut-off (Å)	2.9	2.7	2.7	3.2	2.7	2.8
**Model Refinement and Validation**						
Map Sharpening *B*-factor (Å^2)	-93.9	-97.8	-101.3	-120.3	-111.4	-104.5
Model Resolution at 0.143 FSC cut-off (Å)	2.9	2.9	2.7	3.2	2.7	2.8
Non-hydrogen atoms	10,485	10,593	10,593	10,587	10,077	10,257
Protein residues	1,392	1,404	1,404	1,404	1,338	1,362
Ligands	0	0	0	0	0	0
CC mask	0.84	0.79	0.79	0.85	0.88	0.80
Bond angles (°)	0.566	0.966	0.929	1.202	1.039	1.509
Bond lengths (Å)	0.004	0.004	0.004	0.004	0.005	0.004
MolProbity Score	2.04	2.08	2.39	1.96	1.87	2.28
All-atom Clashscore	9.67	9.77	16.59	9.00	7.07	14.74
Rotamer outliers (%)	1.44	1.71	2.38	1.14	0.60	2.54
CaBLAM outliers (%)	2.54	2.80	3.02	4.09	3.23	2.91
EMRinger score	3.28	1.94	1.88	2.77	2.32	2.42
**Ramachandran plot**						
Favored (%)	93.80	94.21	94.42	93.35	92.27	95.70
Allowed (%)	6.20	5.79	5.58	6.65	7.73	4.30
Outliers (%)	0.00	0.00	0.00	0.00	0.00	0.00
