# Supplementary material for: Structural basis of ammonium transport and coordinated regulation of AMT1;1 in Arabidopsis thaliana
Source: Nat Commun. 2026 Oct 10;17:11526. doi: 10.1038/s41467-026-78457-1 (PMC13745463; doi:10.1038/s41467-026-78457-1)
Supplement: Supplementary file 2 [file 41467_2026_78457_MOESM2_ESM.pdf]

**Description of Additional Supplementary Files**

File Name: Supplementary Data 1
Description: Primer sequences used for cloning and mutagenesis in this study.
