# Supplementary material for: Structural basis of ammonium transport and coordinated regulation of AMT1;1 in Arabidopsis thaliana
Source: Nat Commun. 2026 Oct 10;17:11526. doi: 10.1038/s41467-026-78457-1 (PMC13745463; doi:10.1038/s41467-026-78457-1)
Supplement: Supplementary file 3 [file 41467_2026_78457_MOESM4_ESM.pdf]

nature portfolio

Corresponding author(s): Wei Ying

Last updated by author(s): Jun 5, 2026

Reporting Summary

Nature Portfolio wishes to improve the reproducibility of the work that we publish. This form provides structure for consistency and transparency in reporting. For further information on Nature Portfolio policies, see our Editorial Policies and the Editorial Policy Checklist.

Statistics

For all statistical analyses, confirm that the following items are present in the figure legend, table legend, main text, or Methods section.

n/a	Confirmed	
☐	☒	The exact sample size (*n*) for each experimental group/condition, given as a discrete number and unit of measurement
☐	☒	A statement on whether measurements were taken from distinct samples or whether the same sample was measured repeatedly
☐	☒	The statistical test(s) used AND whether they are one- or two-sided<br>*Only common tests should be described solely by name; describe more complex techniques in the Methods section.*
☒	☐	A description of all covariates tested
☐	☒	A description of any assumptions or corrections, such as tests of normality and adjustment for multiple comparisons
☐	☒	A full description of the statistical parameters including central tendency (e.g. means) or other basic estimates (e.g. regression coefficient) AND variation (e.g. standard deviation) or associated estimates of uncertainty (e.g. confidence intervals)
☐	☒	For null hypothesis testing, the test statistic (e.g. *F*, *t*, *r*) with confidence intervals, effect sizes, degrees of freedom and *P* value noted<br>*Give P values as exact values whenever suitable.*
☒	☐	For Bayesian analysis, information on the choice of priors and Markov chain Monte Carlo settings
☒	☐	For hierarchical and complex designs, identification of the appropriate level for tests and full reporting of outcomes
☒	☐	Estimates of effect sizes (e.g. Cohen's *d*, Pearson's *r*), indicating how they were calculated

Our web collection on statistics for biologists contains articles on many of the points above.

Software and code

Policy information about availability of computer code

Data collection	cryo-EM data collection: EPU (v 2.12) for the 300-kV Titan Krios microscope (Thermo Fisher Scientific) equipped with K3 Summit direct electron detector (Gatan) or Falcon4 direct electron detector and a GIF Quantum energy filter (Gatan)
Data analysis	cryoSPARC (v4.6.2) for cryo-EM structure reconstruction; WinCoot (v0.9.6) for atomic model building; PHENIX (v1.20.1-4487) for model refinement; GraphPad Prism (v8.0.1) for statistical and data analyses; Refeyn DiscoverMP (2024 R1) for mass photometry data analysis; MEGA11 (v11.0.13) for sequence alignment; and ESPript 3.2 for visualization of sequence alignments.

For manuscripts utilizing custom algorithms or software that are central to the research but not yet described in published literature, software must be made available to editors and reviewers. We strongly encourage code deposition in a community repository (e.g. GitHub). See the Nature Portfolio guidelines for submitting code & software for further information.

Data

Policy information about availability of data

All manuscripts must include a data availability statement. This statement should provide the following information, where applicable:

- Accession codes, unique identifiers, or web links for publicly available datasets
- A description of any restrictions on data availability
- For clinical datasets or third party data, please ensure that the statement adheres to our policy

The 3D cryo-EM maps of the WT (without NH4+, pH 7.4), WT (with NH4+, pH 7.4), WT (with MeA, pH 7.4), H372F (without NH4+, pH 7.4), WT (with NH4+, pH 5.5), and T460D (with NH4+, pH 7.4) of AtAMT1;1 have been deposited in the Electron Microscopy Data Bank (EMDB, https://www.ebi.ac.uk/emdb/) under the accession

number EMD-68466, EMD-68512, EMD-68580, EMD-68504, EMD-81185 and EMD-68515, respectively. The corresponding atomic coordinates have been deposited in the Protein Data Bank (PDB, https://www.rcsb.org/) under the accession code 22MG, 22NK, 22OY, 22NG, 27IF and 22NN, respectively.

Research involving human participants, their data, or biological material

Policy information about studies with human participants or human data. See also policy information about sex, gender (identity/presentation), and sexual orientation and race, ethnicity and racism.

Reporting on sex and gender	No human participants or human data are reported in this study.
Reporting on race, ethnicity, or other socially relevant groupings	No human participants or human data are used in this study, thus no socially constructed or socially relevant categorization variables are used.
Population characteristics	See above.
Recruitment	No human participants are recruited in this study.
Ethics oversight	No human participants or human data are reported in this study.

Note that full information on the approval of the study protocol must also be provided in the manuscript.

Field-specific reporting

Please select the one below that is the best fit for your research. If you are not sure, read the appropriate sections before making your selection.

- [x] Life sciences
- [ ] Behavioural & social sciences
- [ ] Ecological, evolutionary & environmental sciences

For a reference copy of the document with all sections, see nature.com/documents/nr-reporting-summary-flat.pdf

Life sciences study design

All studies must disclose on these points even when the disclosure is negative.

Sample size	All of functional assays were performed with at least three replicates and decribed in the figure legends. The sample size were chosen to ensure the reproducibility of the experiments and to get meaningful results. The sample size were adequate based on distribution of data points and clearly visible effects.
Data exclusions	No data were excluded from the analyses.
Replication	All of functional assays were repeated independently and all attempts at replication were successful. The number of replications are described in the text.
Randomization	For cryo-EM 3D refinement, all particles were randomly split into two groups for independent reconstruction. Samples used for functional assays were randomly selected and prepared under the predefined experimental conditions.
Blinding	Blinding was not used in this study, because it is not technically or practically feasible to do so for either the cryo-EM structure determination or the functional assays.

Reporting for specific materials, systems and methods

We require information from authors about some types of materials, experimental systems and methods used in many studies. Here, indicate whether each material, system or method listed is relevant to your study. If you are not sure if a list item applies to your research, read the appropriate section before selecting a response.

Materials & experimental systems

n/a	Involved in the study	
☐	☒	Antibodies
☐	☒	Eukaryotic cell lines
☒	☐	Palaeontology and archaeology
☒	☐	Animals and other organisms
☒	☐	Clinical data
☒	☐	Dual use research of concern
☒	☐	Plants

Methods

n/a	Involved in the study	
☒	☐	ChIP-seq
☒	☐	Flow cytometry
☒	☐	MRI-based neuroimaging

Antibodies

Antibodies used	The anti-Flag antibody for western blotting was purchased from TransGen Biotech (Catalog number: HT201, Lot number: S20822) and used at a dilution of 1:3000.<br>The anti-β-actin antibody for western blotting was purchased from TransGen Biotech (Catalog number: HC201, Lot number: S3250322) and used at a dilution of 1:3000.<br>The HRP-conjugated goat-anti-mouse IgG was also purchased from from TransGen Biotech (Catalog number: HS201-01, Lot number: S11118) and used at a dilution of 1:5000.
Validation	The anti-Flag antibody, anti-β-actin antibody and HRP-conjugated goat-anti-mouse IgG for western blotting were validated by the commercial vendors:<br>anti-Flag: https://www.transgen.com/antibody_reference/393.html<br>anti-β-actin antibody: https://www.transgen.com/antibody_reference/393.html<br>HRP-conjugated goat-anti-mouse IgG: https://www.transgen.com/antibody_second/403.html

Eukaryotic cell lines

Policy information about cell lines and Sex and Gender in Research

Cell line source(s)	HEK293F (Sino Biological Inc.)
Authentication	No further authentication was performed for commercially available cell lines.
Mycoplasma contamination	The cell line has been tested negative for mycoplasma contamination.
Commonly misidentified lines (See ICLAC register)	No commonly misidentified cell lines were used in this study.

Plants

Seed stocks	None.
Novel plant genotypes	None.
Authentication	None.
