# Supplementary material for: Structural basis of ammonium transport and coordinated regulation of AMT1;1 in Arabidopsis thaliana
Source: Nat Commun. 2026 Oct 10;17:11526. doi: 10.1038/s41467-026-78457-1 (PMC13745463; doi:10.1038/s41467-026-78457-1)
Supplement: Supplementary file 4 [file 41467_2026_78457_MOESM5_ESM.pdf]

nature portfolio

Peer Review File

Structural basis of ammonium transport and coordinated regulation of AMT1;1 in Arabidopsis thaliana

Corresponding Author: Professor Wei Ying

This file contains all reviewer reports in order by version, followed by all author rebuttals in order by version.

Attachments originally included by the reviewers as part of their assessment can be found at the end of this file.

Version 0:

Reviewer comments:

Reviewer #1

(Remarks to the Author)
The manuscript submitted to Nature Communications by Wei Ying and co-worders reports on a broadly researched ammonium transporter from plants that belongs to the AMT1 clade.
The authors solved various cryo-EM structures of the Arabidopsis thaliana AMT1;1, filling the lack of an experimentally determined structure for a plant representative of this broadly distributed and conserved family of Amt/Mep/Rhesus ammonium transporters. They present here the high-resolution structures of as-isolated protein with and without added NH4+, or methylammonium cation (MeA), T460D variant with NH4+ and H372F without NH4+, all at pH 7.4 plus the as-isolated protein with NH4+ at pH 6.0. Besides these, they also produced various mutants for biochemical analysis, namely, delta(1-14), delta(1-32), C24S, S262A, D364A, H206F/H372F, W175A, F134A, F258A, F134A/F258A, single and multiple (?) mutations of R69, K71, R223, R226, R232, R237, K361 to alanine, delta471-501 and delta 458-501.
The biochemical characterization involved complementation assays of a yeast strain lacking its three ammonium transporters (Mep1,2,3), thermal stability, culture uptake of 15N-ammonium and mass photometry analysis.
This is an incredible amount of work. However, it suffers from insufficient awareness of the literature and current status of the art. In various situations, the authors consider hypothesis that have long been discredited in the field which also influence and misguide their results interpretation. On top of this, the authors also tend to overinterpret their data without sufficient back up.
In essence, the work is very interesting and relevant but it will need major restructurings and careful data re-analysis to provide a new step into the understanding of this fantastic family of proteins.

Major issues.

1) Oligomeric state of Amt proteins
a) No Amt protein or Rh protein is a stable monomer. This was never (and likely will never) be seen (check Blakey et al 2002, JBC). The size exclusion chromatogram (Fig Suppl 4a) reveals a dominant peak around 16 mL. Although the molecular weight for this retention volume is not given, considering the column type (methods) and the presence of salt in the buffer, it likely corresponds to a molecular weight of 200 – 250 kDa. This fits very well with a membrane protein of (predicted) 160 kDa in LMNG micelles (aggregation number about 65-75 in 150 mM NaCl; molecular weight ca. 1.07 kDa; micelles with at least 65 kDa), all of which adding to a particle with ca. 225 kDa (160 + 65 kDa).
In the Mass Photometry (MP), one can see systematic peaks for 213-250 kDa species (undoubtedly the trimer) and, in some cases, a second peak with higher molecular weight (370-400 kDa). Could this be a hexamer in this in vitro micellar suspension? If not, what?
Please correct the interpretation of the MP data as clearly there is no monomer here.

b) In the opinion of this review, the thermal stability (TS) assays reveal the different stability of the different trimers. Please correct or justify these points.

2) Role of N-terminus extension

a) Why would intra-monomer cystine bonds, at the tip of the N-terminus, away from the protein body, add to the protein stabilization? If not backed-up by evidence, the authors should make a proposal, not a claim.

b) In the variant without the first 14 amino acids (only one Cys per helical N-term), and under the oxidizing conditions used, why don´t you try to see, or consider the eventuality that the N-term of two monomers can now form a cystine bond? Could such asymmetric disulfide bond (leaving one monomer "loose" in the trimer) be the cause of the thermal instability observed?

c) Just as the bacterial periplasm/cytoplasm are oxidizing/reducing compartments, respectively, the plant apoplast maintains a high redox potential that can influence the folding and stability of proteins like AMT1;1. Why not test the reversibility and consequence of these cystine bonds in the intact protein by simply adding excess DTT or another reducing agent to the native protein to break the bonds (keeping the protein under anaerobic conditions during the assay), then reverting the situation in the presence of oxygen again?

d) When plant cells expand in preparation for division, the apoplast becomes more acidic (pH 4.5 -5.5). Is this N-term a sensor for that? In a recent publication (Pflueger et al, Science Advances, 2024), it is suggested that plants AMT1 can be ammonium transporters and receptors (transceptors) because they appear to shave a signature motif for a binding pocket that binds two ammonium cations as signal. The authors don´t look into this interesting hypothesis, yet they have structures and indeed these show some unassigned density near these residues in some (interestingly not all) cases.
This should clearly be analyzed and discussed as it is a unique opportunity and the first time the structure of AMT1;1 is presented.

3) Transport mechanism of Arabidopsis thaliana AMT1;1.

a) The author´s perception of the mechanistic hypothesis for Amt proteins and the role of conserved pore-lining residues (in Amts) is outdated and/or incorrect.

b) The E coli structure (in ref 18, but not ref 19) is misleading and anyone working in the field (especially with structural knowledge) should see that. The structure described by Khademi et al is not of the native AmtB but of a mutant (near the outside vestibule) that likely altered the W/S vestibule environment – note that the second, and native E coli structure (ref 19) does not see this enlarged density). The functional assay, in the same infamous publication (ref 18), has also been broadly criticized in the community (not reproducible, simplistic interpretations) caused serious damages to the field. In essence: the gas channel hypothesis has been dismissed (see next point).

c) At points (but curiously not always), the authors write that the mechanism of ammonium transport by Arabidopsis thaliana AMT1;1 is unknown. But in ref 17 (and in other publications by the same or by other groups), Mayer and Ludewig show that AMT1;1 acts as an electrogenic, pH-independent uniporter for NH_4^+, in contrast with the (then proposed) gas channel idea (ref 18). Meanwhile, an electrogenic assay was developed and shown to be a reliable tool to effectively monitor prokaryotic Amt transport. In that work (Wacker et al, 2014, PNAS), two Amts from Archaeoglobus fulgidus were shown to be electrogenic and subsequently (Mirandela et al, 2019, The Faseb Journal,) the same assay was applied to E. coli AmtB confirming that this protein is also not a NH3 gas channel.
Given the sequence similarities in pore lining residues between E. coli AmtB, Archaeoglobus fulgidus and many others, with the Arabidopsis thaliana AMT1;1, this reviewer would assume that, most likely, they share the same basic transport principles. Please discuss the data obtained in view of these updates and considering advancements on the role of functional important residues. For example, W/S were shown to delay transport (Wood et al, 2008, JBC), MD simulations on F/F showed that the phenyl groups just have to tilt to allow cations to pass (Lin et al, 2006, JACS) and H/H may act to deprotonate and re-protonate NH4 (Ullmann et al, 2012, J Phys ChemB).

4) Assignment of NH3, NH4+ or Am
If intending to propose a mechanism of ammonium transport, the simplification to Am, is not beneficial. And this assignment is extremely difficult to do (as broadly reported in the literature).

a) Densities below the second H in the pore have been seen in previous structures. If you make an assignment, justify it based on data (not only the structure). If not, leave it as water as many others have done and explain it.

b) Why/how other map features are assigned as NH3 or NH4+ in the structures and in Fig 7?

c) There are basically two theories on where and how internal deprotonation of NH4 to NH3 occurs. At the same time, it seems reasonable that a H+ should not be left outside or be consumed inside in the process (as is suggested on the end of p. 15). Instead, an internal H+ pathway may exist, for example, provided by H/H (see Ullmann, J Phys ChemB, 2012 or others). The hypothesis that positively charged residues in the cytoplasmic face of the protein would give a H+ to NH3 implies that a H+ would be used from the cytoplasm to regenerate their state. This does not seem likely (Andrade et al, Mol Memb Biol, 2007). In view of this, could the effect on transport caused by these mutations be the structural displacement of cytoplasmic regions?

d) It is not clear why so few molecular structural details are shown. Figures in general are very global, simplistic and do not show details. The authors do not really describe with sufficient detail the very interesting structural changes that occur in the intracellular face of AMT1;1 from the various structures shown. Above all, they do not establish a cause-effect.

e) P10/11, l194-198: please clearly show the density for this position and the assigned methylammonium. What is the structural confidence of this assignment after refinement? If the authors choose to cite ref 17 here, please mention that the structure is of a mutant.

5) P.11 l200: where is the high conservation of residues at the pore exit? This exit vestibule is broad and formed by many residues. Therefore, it is not clear in neither of the supplemental figures mentioned, the identity of these residues. Please also clarify why they are considered relevant, in the context of the sentence.

6) When introducing mutations, the rationale behind is not always clear. This happened eg with the H/F single or double mutations (previously shown by several researchers to inhibit transport), or the S262, D364, etc. Give a context to the mutated sites.

7) Twin-His to F single and double mutations, W175, F/F to A single and double mutations, as well as C-term deletions, have been done for AMT1;1 (ref 29; Yuan et al, JBC 2009; ref 28, Ortiz-Ramirez et al, 2011, JBC; etc) or for homolog proteins. Please cite relevant previous work and discuss the results accordingly (are the results the same or not and what that means).

8) P12 l 223 – 227: The conclusion that NH3 passes the F/F site lacks justification, considering the results obtained and the existing electrophysiological data for this protein (ref 17)

9) Please mention the orientation in the membrane of the helices shown in Supplemental Figs 7 and 8. (Why is the cytoplasm always up?)

10) What was the rational for choosing pH 6.0 (above the apoplast pH)? When interpreting data at lower pH, the hypothesis that pH can affect the protonation state of particular residues with structural consequences is not discussed. Couldn´t this simply be the case?

11) P15: When mentioning positively charged residues in cytoplasmic loops, it is not straightforward to understand which mutations were tested in the text. Also, single and double mutants are mentioned but double of which?

12) Start of p13: What are the concrete observations that allow stating that NH4+ release is facilitate by a slight outward deflection of TM7-TM8? This reviewer does not see it.
Maybe an improved figure should show (quantify) how much they move and detail it better. Are there amino acid interactions formed/broken because of this? Does the pore geometry change? From the figure provided, it looks like the connecting loop is what changes most?

13) Fig 5b should depict the orientation of helices with regards to the orientation seen in a). Is the red arrow a double arrow?

14) P 13 l 244: Indeed. But what pH increase are the authors considering? Because with a pKa around 9.2 (at 20 degrees Celcius), at pH 7.0 more than 99% of the entire Am is in the protonated cationic form. At pH 8.0 ca. 94% is NH4+. Does this correlate with the observations?

15) P13 l252-254: Please backup this sentence with the structures of the protein at pH 7.4 and 6.0, with and without ammonium provided, what structural features support this? If none are visible, rationalize reasons for that

16) P14 l268-275: Similar assays have been done previously on various homolog Amt proteins. Please mention, cite that and state that accordingly, for all Amts, the C-term integrity has been shown to be essential for functon(see point 4d)).

17) End of p14: As an important point, and to show that T460D disturbs the cytoplasmic interactions, the region becomes more flexible and the maps are therefore weaker: Supplemental Figure 12b does not show this well. Why not do something equivalent to panel c) (or even better, as it was done in Supplemental Fig 7c)). Also, showing stereo pictures can help.

18) In Fig 6:
a) Panel a): the highlights for the regulatory domain are basically invisible. It is hardly possible to note any differences between both structures.
b) Panel b): legend describes the right side elements as the isolated C-term (ok) and intracellular loop region. However, the circle on the left protein figure is apparently misleading here, since that area is not shown on the right bottom figure. According to the text, the right bottom figure is the cytoplasmic face of the protein without the top (C-term module). What is the circle on that intracellular loop region on the right? The exit vestibule? - not described.
c) Panel d): identify what the sequences in the boxes are showing - segments of the different intracellular loops that interact with the C-term region?

d) P15 l 293: Should D264 be D364 instead (considering the sequence and Fig 6b)? Why is this position highlighted? What is its relevance? It is only mentioned here, as a conserved residue located at the pore exit (see point 6).

19) P 15,16 l299-305: the plasma membrane is surrounded by ions. What is the local pH there? Existing literature describes AMT1;1 as electrogenic (ref 17). As mentioned above (see 4c)), it appears unlikely that H+ are consumed on the lumen side of the membrane as consequence of AMT transport. The authors could make better conclusions, sustained by their observations on these loop residues and C-terminus interactions or delta C-terminus – again conserved residues and again

the same observations across AMTs.

20) P16, 17 last paragraph:
a) make proper citations to previous work(s) showing similar or different results from what is observed here. If (as it seems) conclusions here are backed up by other works, cite them.
b) Nothing in this work indicates that NH4+ is deprotonated at the F/F site.
c) NH4+ or Am will never bind to a transporter, rather interact
d) What data allows predicting that D264 – again likely D364 – is involved in NH3 binding (how?) and translocation (how)?
e) Lines 317-320 (see comments above)
f) This reviewer has serious problems with this mechanistic proposal and the scheme shown in Fig 7. Such problems arise from considerations on data published on other Amt proteins (and the high degree of conservation observed between them and AMT1;1, especially in the pore region), published functional data on this AMT1;1 protein and the over-interpretation of results by the authors.

Minor issues:

p.10, l193, p11 l 199, l 203, l 204 (and others): please change channel for pore.

P11, l 203: histidine is not a hydrophobic residue

P11 l 206 add a reference to the given hydrated radius of NH4+

P15 l289-290: Why not simply name loops and they originate after TM? TM1-TM2 forms L1, and so on, such that the loop shown in Suppl Fig 12c) is L5, described in ref 20 as loop 5 with important connections to the C-terminus of own and neighbor monomers.

P34 l 554: replace key by targeted

P16 l307: only in plants?

P19 l 358: replace deprotonated ammonia by ammonium cation

Along the text: AMT1 and AMT2 are two clades of plant AMT types and these belong to the AMT/MEP/Rh family of ammonium transporters

There seems to be a mistake when refering to a density map with NH4+ at pH 7.4 and pH 6.0. In p 12, lines 235 and l 236 both suggest supplemental figure 8b (which only shows helix 10 at low pH).

Discussion should not repeat sentences from the introduction.

Supplemental Fig13: Which structure of Ecoli was used to observe and compare C-termini? All structures of AmtB have a disordered C-terminus. The only observation of this region in the Ecoli AmtB was in the complex structure with its regulator protein. Alternatively, the structure of Archaeoglobus fulgidus Amt1 alone comprised the entire protein up to the C-terminus.

P20 l391: Which intracellular loop of MEP2?

W255 is not correctly modelled in the structure PDB: 22NK (WT, with NH4+, pH 7.4)

Reviewer #2

(Remarks to the Author)
The manuscript by He et al., reports the structure of AMT1.1 in apo and neutral pH and bound to ammonium under neutral and acidic conditions. In addition, the study reports the structure of the phosphomimetic mutant T460D, revealing the mechanism of inactivation. AMT1:1 play a crucial role in the uptake of environmental ammonium in green leafy plants. Ammonium transport in plants is crucial for acquiring, distributing, and assimilating nitrogen, a vital nutrient for growth, development, and high crop yields. As such, this study reports the structure of an important transporter that will be of interest to a wide range of researchers in structural biology, plant sciences and agricultural research. However, the study is currently too preliminary for me to recommend publication at this stage. I have detailed my thoughts below. In summary, the results presented are an excellent start and certainly hint at the mechanisms drawn from the data. However, many of the experiments are insufficiently detailed to draw the types of mechanistic conclusions being made. While the yeast complementation assays are useful as a general screening tool for activity, they fail to provide the resolution needed to ascribe functions to single amino acid side chains. The inconsistency between the complementation assay and the isotope uptake assay is concerning and may hint at a deeper disconnect between these two assays. The structures, while sufficient to determine the overall structure, are too low resolution to unambiguously identify the ligand or understand the allosteric mechanism behind the T460D mutant. The authors also appear not to have considered the role of the disulphide in oxidative regulation of the system.

My recommendation is to undertake more detailed studies, consider molecular dynamics, and improve the resolution to

determine the structure in C1 at high resolution.

Detailed comments:

Lines 64-65: ‘add-ammonium state’ should be re written.

Lines 98-101: The authors state that the assays shown in Fig. 1 demonstrate that AMT1.1 is a high affinity transporter of ammonium. I would disagree with this statement, as the assays do not provide any quantitative analysis of affinity. The yeast complementation assay in Fig. 1a demonstrates that under low ammonium conditions (50 uM), the overexpression of AMT1.1 is sufficient to allow growth in the ammonium transporter deficient background. However, this does not provide a value for the Km. If the Km for A. thaliana AMT1.1 has been determined previously, the authors can quote this in the paper. However, if the Km has not been previously reported, then I suggest the authors calculate the Km so this can be compared to the bacterial homologues discussed in the introduction. The authors can use the isotope labelled NH4+ substrate and quantification with an LC-MS/MS system and the HEK293 cells.

Extended Data Fig. 4: Is the upper band on the SDS-PAGE Hsp70? This should be mentioned in the figure legend or labelled in the gel.

Extended Data Fig. 9b: The MST data look somewhat strange to me. Can the authors provide the raw data and detail how the Tm values are calculated? The shape of the unfolding curves for 1-32, 1-14 and C24S are different from the WT, indicating a substantial change in behaviour. I need more details about this assay and the authors interpretation to understand what this data tells me about these mutants. The current explanation is not satisfactory. The mass photometry data shown in Extended Data Fig. 9c suggests that the truncations, plus C24S, are monomeric. However, the unfolding curves are different to the WT. Thus, I conclude the assay shown in Extended Data Fig. 9b may simply indicate the trimer breaking apart, rather than unfolding? It is hard to say.

Extended Data Fig. 9c: I am also cautious about the data presented from the mass photometer. In our hands samples in detergent generate inherently noisy traces due to excess micelles. How was this data generated, through rapid dilution from LMNG?

My understanding from this data is that the truncations and C24S potentially result in monomeric protein, which appears to be non-functional. The truncations are expressed at a lower level. The authors need to provide expression data as an additional panel in Extended Data Fig. 9.

It appears that the disulphide bond is critical for trimer assembly, yet this is not discussed.

Also, Extended Data Fig. 9b is called before Extended Data Fig. 9a.

The data shown is Extended Data Fig. 10 and Extended Data Fig. 11 appears to show the binding of ammonia/ammonium and meA to the transporter. I think it is almost impossible at this resolution to be certain this density corresponds to the ligand. I appreciate the density disappears in the H372F mutant, but this is still very speculative. In my opinion, the strongest evidence using the current data is to overlay the higher resolution structures of the bacterial Amt transporters and identify the density using the superposition. Given the high level of sequence conservation between the two transporters, it is highly likely the density corresponds to substrate. However, I don’t think the cryo-EM data alone is sufficient to support this conclusion.

Line 214 & Figure 4d-e: The authors state the W175A mutant displayed the same growth on the yeast complementation assay yet displayed reduced transport in the isotope uptake assay. However, it looks to me as though more yeast colonies are in the 10-6 condition in panel d compared to the WT. A similar case is shown in F258A. However, neither W175A nor F258A show higher uptake (panel e). This is confusing. Can the authors explain this contradiction in the data?

I think the authors need to establish a more sensitive assay or undertake more detailed uptake kinetics to fully understand the role of these side chains in the function of AMT1.1. As it stands, the data shown in Fig. 4 does not really provide much additional insight.

Figure 5: I am also not convinced by the conclusions drawn from the structures of AMT1.1 under neutral and acidic conditions. The structural changes are relatively minor and occur in loop regions. These could easily be attributed to changes in grid preparation or electrostatics. How these relate to any impact of pH under physiological conditions is not clear. This section of the paper is very preliminary in my opinion and probably needs further studies, maybe using molecular dynamics, and biochemical analysis to draw any firm mechanistic conclusions on the role of the pH gradient in AMT1.1 function.

Extended Data Fig. 12: The authors need to show the expression of the mutants shown in panel a. The reduced growth in the yeast could be due to reduced transport or toxicity related to protein folding/UPR.

Lines 255-308: This section of the study reports the structure of the T460D mutant and the possible impact on the cytoplasmic loop between E220 and S240. I found this section to be far too speculative given the current data. The authors offer no credible allosteric mechanism linking the structural impact of the T460D mutant and the cytoplasmic loop. These

changes may be meaningfully correlated or not. I was unconvinced by this data, and the interpretation appears over interpreted. To validate the proposed model the authors, need to design more accurate assays and I suggest, molecular dynamics is likely the best place to start. Alternatively, perhaps solving the structure in nanodiscs may increase the resolution? I also wondered whether the structural models and maps presented in Extended Data Fig. 12 and Figure 6 were from the C3 averaged structures? If so, the authors should probably determine the C1 structures and see if these changes are unique to each monomer?

Reviewer #3

(Remarks to the Author)
Review He et al., Nature Comm.
Structural basis of ammonium transport and phosphorylation-2 mediated regulation of AMT1;1 in Arabidopsis thaliana
Ammonium serves as an important nitrogen form for microbes and plants. AMTs had been shown to be responsible for ammonium acquisition across a wide range of species. There is extensive experimental evidence including transport studies, studies of mutants, yeast and plant mutants and evidence for the role of kinases in regulation via phosphorylation. Also, activity sensors had been developed. Furthermore, evidence had been presented that AMTs may function as transceptors, with dual roles in Am transport and signaling. Moreover, several structures have been published for diverse family members.
Notably extensive mutagenic studies to characterize the role of individual residues for transport function, notably interaction of loop regions with the conserved C-terminus required for activity.
It is thus not clear why the authors emphasize that the function Poorly understood
While the reviewer is not a structural biologist, this is a appears to be a very nice piece of work providing not only cryoEM structures of the Arabidopsis AMT1, but also that of mutant forms, e.g. the important T460C-terminal. It would be recommended to involve structural experts in the review process.
Key results presented here:
- A 1.2 Å structure of AMT1 from Arabidopsis and validation using a variety of mutants, however without properly including all the known mutant and modeling studies (Nygaard, Graff et al., various Loque publications, Yuan et al et.; possibly useful to also look at the supplemental data in these publications). While some of the references are in the bibliography or Discussion, the interpretation and even the experiments here are based or show what was predicted or shown before. For example Phe gate is even a paragraph in reviews from 2010 (www.sciencedirect.com/science/article/pii/S124678201000039X). This is not to criticize the excellent structural work, rather the way the results are presented here as original.
- Structures at two pH conditions show potential differences with TM7–TM8 with an outward deflection, resulting in a wider opening on the intracellular side. The authors conclude that pH plays a role in regulating the activity, and use growth assays to evaluate the difference at different pH. Notably, pH dependency had been shown previously using transport assays, with results not in line with the observation shown here (Ninnemann et al., 1994). Growth assays are not likely a suitable tools for evaluating pH dependencies of specific proteins.
- Demonstration of N-terminal truncations cause defect. This is interesting, yet not unexpected and does not appear to represent a major new insight.
- Disulfide bridge important – It may be useful here to mention literature that analyzed the role of reducing agents, or even observed the cysteines in structures.
- Demonstration that T460 in the conserved C-terminus is important (this is well known and the mechanism is very well studied). The text gives the impression as if this was discovered here.
Major points
Structures of AMT homologs had been obtained previously. Novel here is that we deal with a plant transporter, for which a critical role of the C-terminus had been demonstrated. An advance here is that the C-terminus is includes in the structure of the eukaryotic protein (the C-terminus was also structurally determined in the AMT from A. fulgidus. Major gaps in our knowledge relate to the transport mechanism, that is the conformational rearrangements in a transporter during the transport cycle. This study provides no new insights into the transport cycle.
Notably, the authors refer almost exclusively to their own mutations shown here, but do not discuss the ample number of mutagenic studies which have been published before, not do they consider them here. It appears as if the authors were the first ones to identify the role of T460, or the interaction of the C-terminus and the cytosolic domains (specific residues were known for both).
There are multiple kinases and phosphorylation sites known, which are not discussed here.
Key question not resolved regarding the transport mechanism, ammonia, ammonium or proton symport.
Also not mentioned nor studied is the evidence that AMTs may serve as transceptors, so possibly.
Electrophysiological evidence for transport of charge not considered here.
Minor points:
Aspartate is typically referred to as a mimic of phosphorylated Ser or Thr. However the structures and properties are very different. Thus while one may be able to cautiously state that if we assume that , however the reviewer would suggest not to state that this demonstrates the effect of phosphorylation. The statement: ‘Following T460 phosphorylation, the C-terminus becomes disordered ‘ and others should be cautioned accordingly.
Overall, while this is a very nice and extensive study, and the first to provide a full structure including the soluble C-terminus at high resolution. Even though the structures appear to be very good, the work does not appear to provide many novel insights into the mechanism nor represent a major advance over the previously published work.

Version 1:

Reviewer comments:

Reviewer #1

(Remarks to the Author)
After major modifications, the manuscript is indeed much better. But since so much was reinterpreted and (re-)written, new issues need to be addressed.

The introduction and discussion of results is now much better, including some of the crucial literature and a correct description of the state of the art regarding the mechanistic understandings of ammonium transport in general, as well as particular aspects concerning AtAMT1;1. On page 3 line 44: I would not say that the mechanism is still unclear. I would say that experimentally proving/disproving the best hypothesis is extremely challenging.
Accordingly, the sentence in page 5, lines 80 – 82 should be modified since the structures presented and analyzed do not elucidate the molecular mechanism of ammonium transport. Line 90 should also be altered because “facilitator” is a word best used to describe a passive, down-gradient, channel-like mechanism and this reviewer saw nowhere in the manuscript experiments that could proof this (or any other) mechanism.

While major improvements have been done to the current version, the concept of AtAMT1;1 functioning as a transceptors is now briefly mentioned but remains poorly investigated (see attached file with figures included).
This point should be properly mentioned in the introduction (eventually, after line 77). This will make the description of result and later discussion easier to understand.

Despite all improvements the manuscript remains of questionable quality in the experimental data interpretation, presentation and discussion. In particular, the handling and analysis of structural data (major value of the paper) is surprisingly sloppy.
For example, the monomers in the trimers show a perfect 3-fold symmetry, while the map does not. The map shows variations that have not been taken into consideration. Moreover, all Ramachandran plots show perfect, ideal geometries, indicating that the authors may have refine (if at all) the data with very high restricted geometries. Potentially only one monomer was modelled (although I do not think so given that if I do it, I see changes adapting to the map), but certainly they did not worked on the trimer - unbelievable.

(Please see attached file with much more remarks with added figures for clarity).

Comments from Reviewer #3
The revised manuscript has clearly improved in what concerns the overall description of the state-of-the-art in the field of Amt proteins, the correct citation and inclusion of relevant literature describing work done by others and the perception of relevant or actual hypothesis. This was a major problem in the early submission.

The previously well-studied role of the C-terminus phosphorylation in allosteric transport regulation and the N-term potential regulatory sites are important hallmarks in the field of plant Amts. Their structural description can certainly help researchers advancing our understanding of these proteins.

At the same time, other points remain vague or have not yet been addressed. For example, the role of this protein in sensing. The authors only left a small part of the discussion to the topic. But while they say that the residues required to detect ammonium are present in this Arabidopsis protein, they do not show any picture of the site or describe it any further. Seems like a lost opportunity not to show this important and unique point of a plant Amt protein that has been vastly understood as "only" a transporter and indeed may function as a sensor of ammonium as well.
In my opinion, the relevance of this work to the field is extremely significant - the last homolog in the Amt/Mep/Rh superfamily whose structure was missing - but the authors still perform below what they could do/have.

Reviewer #2

(Remarks to the Author)
The authors have addressed my previous comments and introduced additional clarification and structural analyses.

Version 2:

Reviewer comments:

Reviewer #1

(Remarks to the Author)
The reviewer thanks the authors for the extended work done during revision.

One last remark, easy to verify and to include:

L 82: As far as this reviewer understood from reading the two publications related to the bacterial Amts that have N1/N2 motif, they do not seem to act as transporters, thus they may not be best described as transceptors (in line 82: there is no transport-coupled signaling activities). Instead it is described that ammonium binds at N1/N2.

Change this accordingly throughout the manuscript. However, in line 85 this seems correct since At AMT1;1 has been described in the literature as a transporter. So, if they would also bind ammonium at N1/N2 they would then be transceptors.

Open Access This Peer Review File is licensed under a Creative Commons Attribution 4.0 International License, which permits use, sharing, adaptation, distribution and reproduction in any medium or format, as long as you give appropriate credit to the original author(s) and the source, provide a link to the Creative Commons license, and indicate if changes were made.
In cases where reviewers are anonymous, credit should be given to 'Anonymous Referee' and the source.
The images or other third party material in this Peer Review File are included in the article’s Creative Commons license, unless indicated otherwise in a credit line to the material. If material is not included in the article’s Creative Commons license and your intended use is not permitted by statutory regulation or exceeds the permitted use, you will need to obtain permission directly from the copyright holder.
To view a copy of this license, visit https://creativecommons.org/licenses/by/4.0/

We thank all the reviewers for their time and critical comments, which helped us in improving the quality of the manuscript. The following is a detailed response to the specific suggestions of each reviewer:

Reviewer #1:

The manuscript submitted to Nature Communications by Wei Ying and co-worders reports on a broadly researched ammonium transporter from plants that belongs to the AMT1 clade. The authors solved various cryo-EM structures of the *Arabidopsis thaliana* AMT1;1, filling the lack of an experimentally determined structure for a plant representative of this broadly distributed and conserved family of Amt/Mep/Rhesus ammonium transporters. They present here the high-resolution structures of as-isolated protein with and without added NH_4^+, or methylammonium cation (MeA), T460D variant with NH_4^+ and H372F without NH_4^+, all at pH 7.4 plus the as-isolated protein with NH_4^+ at pH 6.0. Besides these, they also produced various mutants for biochemical analysis, namely, delta (1-14), delta (1-32), C24S, S262A, D364A, H206F/H372F, W175A, F134A, F258A, F134A/F258A, single and multiple (?) mutations of R69, K71, R223, R226, R232, R237, K361 to alanine, delta471-501 and delta458-501.

The biochemical characterization involved complementation assays of a yeast strain lacking its three ammonium transporters (Mep1,2,3), thermal stability, culture uptake of ^{15}N-ammonium and mass photometry analysis.

This is an incredible amount of work. However, it suffers from insufficient awareness of the literature and current status of the art. In various situations, the authors consider hypothesis that have long been discredited in the field which also influence and misguide their results interpretation. On top of this, the authors also tend to overinterpret their data without sufficient back up.

In essence, the work is very interesting and relevant but it will need major restructurings and careful data re-analysis to provide a new step into the understanding of this fantastic family of proteins.

Response: We sincerely thank the reviewer for the careful evaluation of our work and

for the constructive comments. We fully appreciate the reviewer's concerns regarding the interpretation of transport mechanisms and the discussion of previous studies in the AMT/Amt/Rh field. In the revised manuscript, **we have substantially restructured both the Results and Discussion sections to provide a more balanced and rigorous interpretation of our data.**

In particular, we have moderated several overinterpreted conclusions from the previous version and avoided making definitive claims regarding whether the transported species is NH_4^+ or NH_3, as this remains an actively debated question in the field. Instead, the revised manuscript focuses primarily on the structural features of the substrate conduction pathway and the regulatory mechanisms governing transporter activity.

To strengthen the study, we further performed and incorporated additional experiments, including mass photometry analysis, yeast complementation assays, and molecular dynamics simulations. These new data allowed us to better support the roles of the extracellular N-terminal disulfide bond in redox-dependent structural stability and the intracellular C-terminal phosphorylation-dependent regulatory mechanism. We also expanded the comparative analysis with bacterial AmtB and fungal Mep2 transporters and revised the discussion accordingly to better reflect the current understanding of the field.

We believe these substantial revisions have significantly improved the clarity, rigor, and mechanistic relevance of the manuscript.

Major issues:

1) Oligomeric state of Amt proteins

a) No Amt protein or Rh protein is a stable monomer. This was never (and likely will never) be seen (check Blakey et al 2002, JBC). The size exclusion chromatogram (Fig Suppl 4a) reveals a dominant peak around 16 mL. Although the molecular weight for this retention volume is not given, considering the column type (methods) and the presence of salt in the buffer, it likely corresponds to a molecular weight of 200-250 kDa. This fits very well with a membrane protein of (predicted) 160 kDa in LMNG micelles (aggregation number about 65-75 in 150 mM NaCl; molecular weight ca. 1.07 kDa; micelles with at least 65 kDa), all of which adding to a particle with ca. 225 kDa

(160 + 65 kDa).

In the Mass Photometry (MP), one can see systematic peaks for 213-250 kDa species (undoubtedly the trimer) and, in some cases, a second peak with higher molecular weight (370-400 kDa). Could this be a hexamer in this in vitro micellar suspension? If not, what? Please correct the interpretation of the MP data as clearly there is no monomer here.

Response: We thank the reviewer for this important comment and fully agree that our original interpretation of the mass photometry (MP) data was inappropriate. As correctly pointed out by the reviewer, AMT/Amt/Rh family transporters are well established as stable oligomeric assemblies rather than monomers, and our previous assignment of the lower-molecular-weight peak as a monomeric species was not justified.

To address this issue, we performed additional control experiments and re-analyzed the MP data. Specifically, we included detergent-only buffer controls and compared the MP profiles of *At*AMT1;1 purified in both LMNG and DDM detergents. These new analyses indicate that the earlier low-mass peak (~240–300 kDa) primarily originates from detergent micelles/background scattering rather than monomeric protein species **(Extended Data Fig. 7b, c of the revised manuscript, and is also presented below).** In contrast, the higher-molecular-weight peak (~370–400 kDa) most likely corresponds to the stable trimeric assembly of *At*AMT1;1 in detergent micelles. We have therefore revised the interpretation of the MP data throughout the manuscript and removed the previous discussion suggesting the presence of monomeric species.

The revised discussion of the MP data has been substantially updated **(Lines 170–185),** and the new control experiments are presented **(Extended Data Fig. 7).**

Extended Data Fig. 7b, c of the revised manuscript:

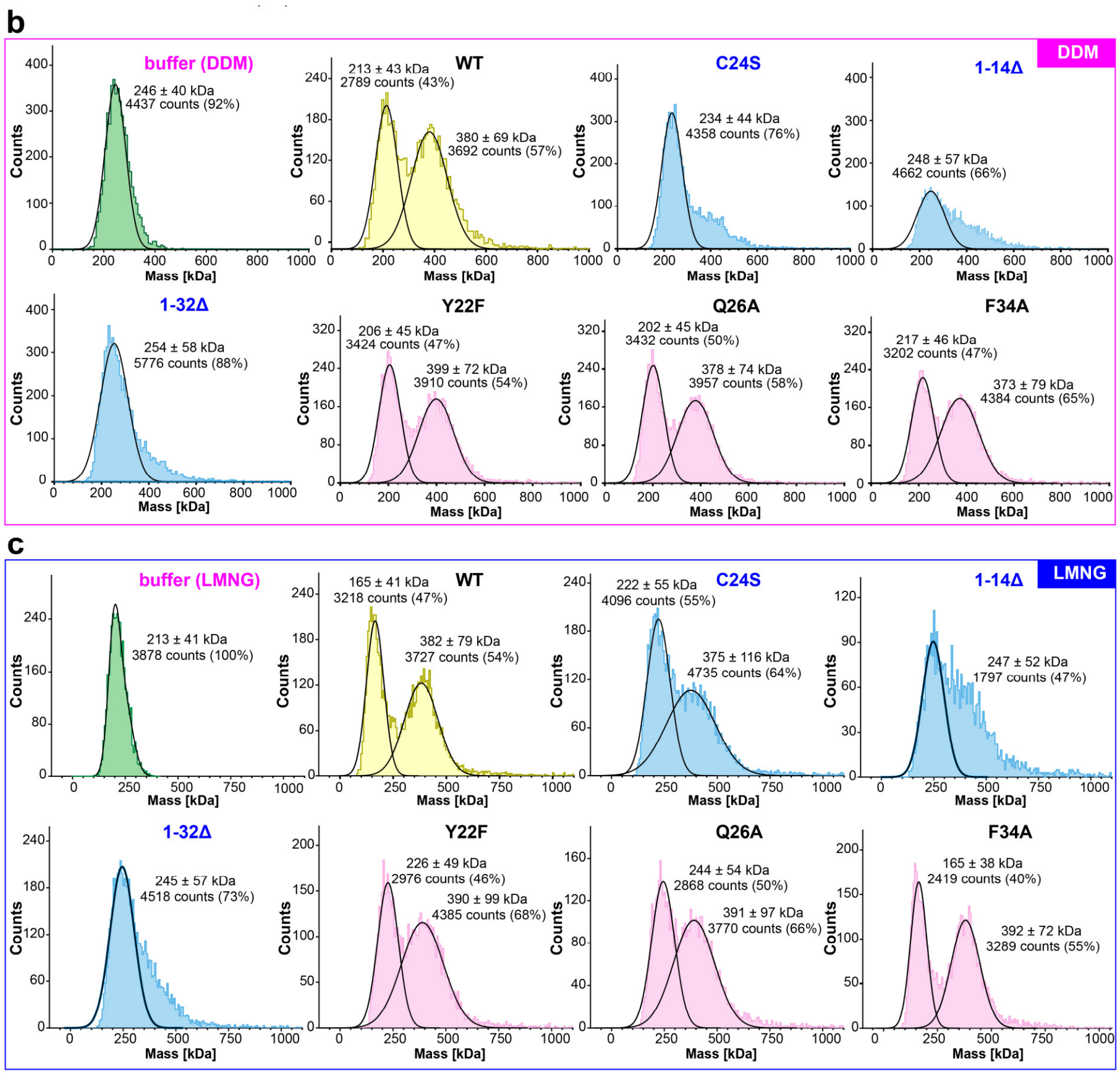

b) In the opinion of this review, the thermal stability (TS) assays reveal the different stability of the different trimers. Please correct or justify these points.

Response: We thank the reviewer for this valuable suggestion. In the revised manuscript, we re-evaluated the thermal stability data more carefully. First, size-exclusion chromatography and SDS–PAGE analyses showed that the expression level and purification behavior of the mutants and truncation variants were comparable to those of the wild-type protein, arguing against major defects in protein production or gross misfolding.

To further quantify thermal stability differences, we performed additional fitting analyses of the thermal unfolding curves and calculated the apparent melting temperatures (Tm) for all variants **(described clearly in revised Method Lines 822–826 and the fitting curves are also presented below)**. These analyses revealed that the C24S mutant and the N-terminal truncation variants (1–14Δ and especially 1–32Δ) exhibited reduced Tm values compared with the wild type and other single-point mutants, indicating reduced thermal stability of the transporter complex.

We have revised the corresponding text accordingly **(Lines 166–170)** and included the

fitted Tm values in the updated Figure 3c.

Determination of melting temperatures (Tm) of *At*AMT1;1 variants:

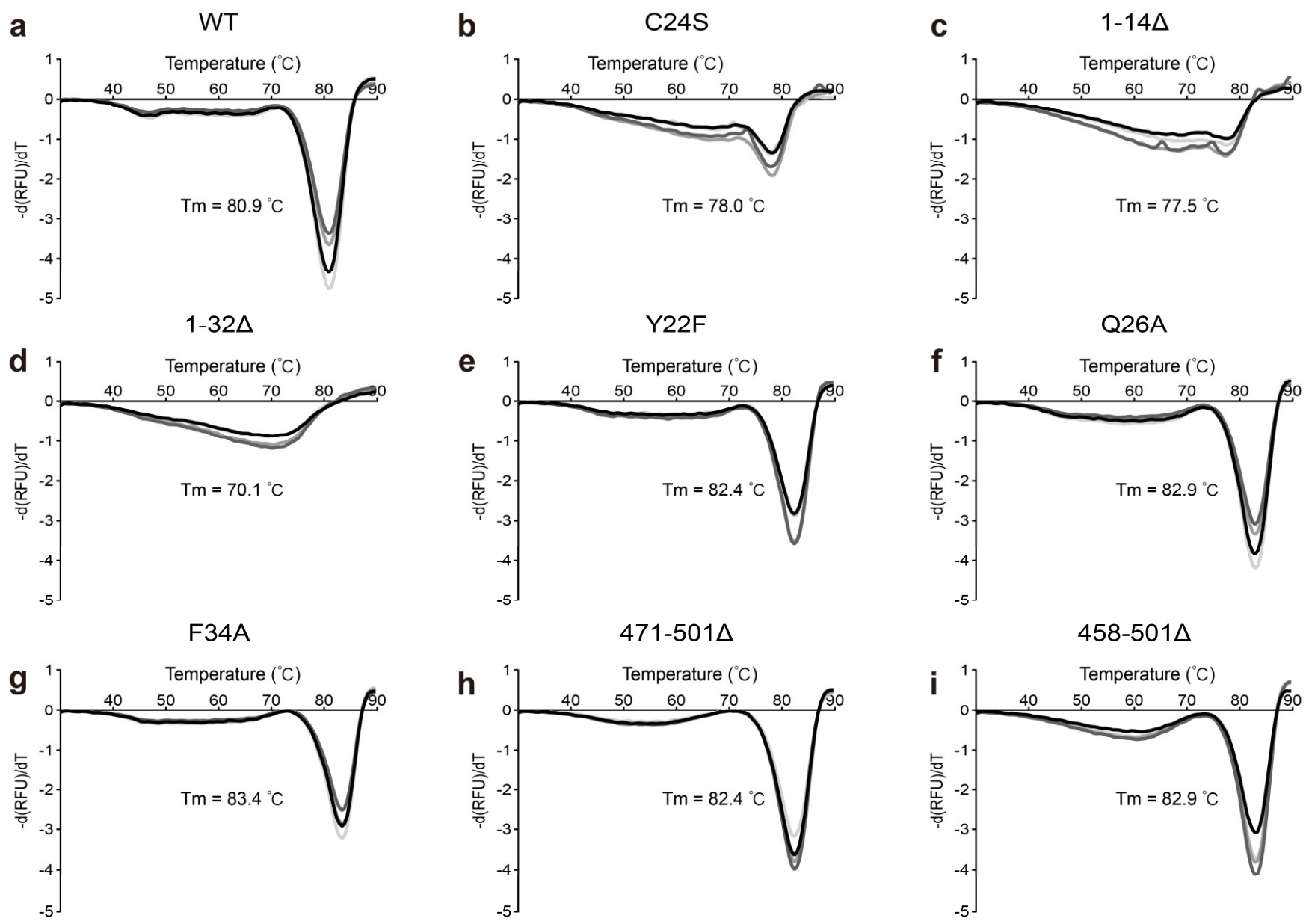

Fig. 3c of the revised manuscript:

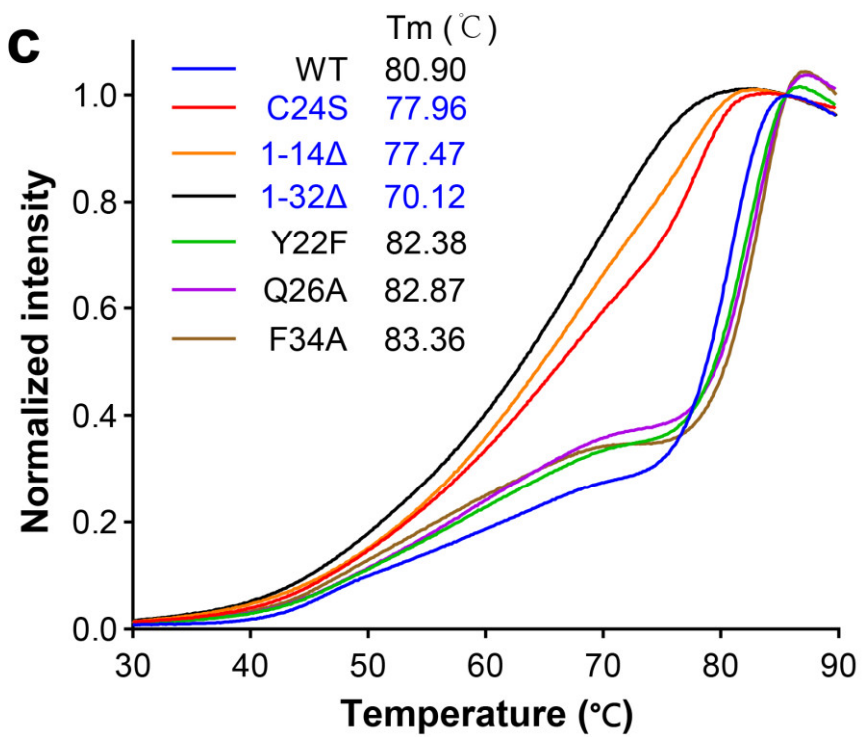

2) Role of N-terminus extension

a) Why would intra-monomer cystine bonds, at the tip of the N-terminus, away from the protein body, add to the protein stabilization? If not backed-up by evidence, the authors should make a proposal, not a claim.

<u>Response:</u> We thank the reviewer for this insightful comment. We agree that our previous wording was overly definitive. In the revised manuscript, we have toned down the conclusion and now describe the stabilizing role of the N-terminal disulfide bond as a proposed mechanism rather than a direct demonstration.

Although the disulfide bond is located at the distal extracellular N-terminus and does not directly contact the transmembrane core, several experimental observations support

its contribution to transporter stability and function. Specifically, the C24S mutant and N-terminal truncation variants exhibited reduced thermal stability, decreased transport activity, and altered behavior in mass photometry analyses. These results suggest that the N-terminus may help maintain a conformation compatible with stable trimer assembly or productive transporter dynamics. At present, however, we cannot exclude the possibility that the disulfide bond affects AMT1;1 indirectly, for example by constraining the flexibility or orientation of the extracellular N-terminal segment, thereby influencing long-range conformational coupling within the trimer. We therefore revised the text throughout the manuscript to avoid overinterpretation and to present this model as a working hypothesis supported by biochemical and structural observations.

We have revised the corresponding text accordingly **(Lines 510-516)** in the Discussion part.

b) In the variant without the first 14 amino acids (only one Cys per helical N-term), and under the oxidizing conditions used, why don't you try to see, or consider the eventuality that the N-term of two monomers can now form a cystine bond? Could such asymmetric disulfide bond (leaving one monomer "loose" in the trimer) be the cause of the thermal instability observed?

Response: We thank the reviewer for this insightful suggestion. In the resolved structure, the C24 residues of individual monomers are spatially distant, making formation of intramolecular disulfide bonds unlikely **(figure is also presented below)**. However, in truncated variants the N-terminal region may undergo rearrangement, bringing the C24 residues into proximity. Accordingly, we agree that, in the 1–14Δ variant, the remaining N-terminal cysteine could potentially form intermolecular disulfide bonds between neighboring protomers under oxidizing conditions. Such asymmetric inter-subunit disulfide linkage could, in principle, generate heterogeneous trimer populations and contribute to the reduced thermal stability observed for this truncation variant.

At present, however, we do not have direct experimental evidence to distinguish between intra- and intermolecular disulfide configurations in the 1–14Δ construct. Importantly, the 1–14Δ variant still exhibited markedly reduced thermal stability

compared with the wild type, whereas the more extensive 1–32Δ truncation showed an even stronger destabilizing effect. These observations suggest that, beyond the disulfide bond itself, the integrity of the N-terminal region is likely important for maintaining proper transporter stability and function. We therefore agree that the possibility of asymmetric inter-subunit disulfide formation in the 1–14Δ variant represents an interesting alternative explanation.

Structural features of the C24 residue:

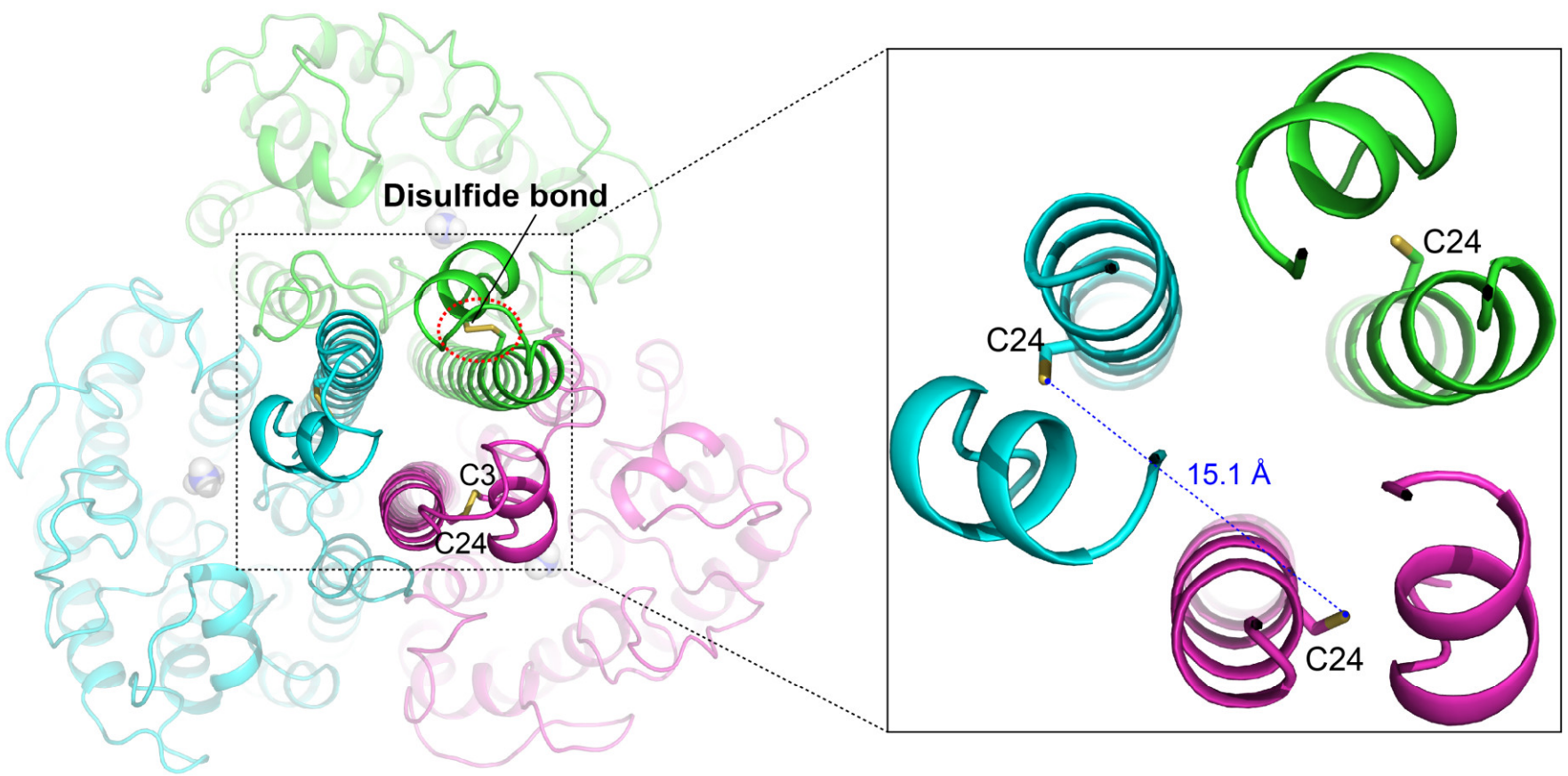

c) Just as the bacterial periplasm/cytoplasm are oxidizing/reducing compartments, respectively, the plant apoplast maintains a high redox potential that can influence the folding and stability of proteins like AMT1;1. Why not test the reversibility and consequence of these cystine bonds in the intact protein by simply adding excess DTT or another reducing agent to the native protein to break the bonds (keeping the protein under anaerobic conditions during the assay), then reverting the situation in the presence of oxygen again?

Response: We thank the reviewer for this valuable suggestion. In the revised manuscript, we performed additional experiments to examine the reversibility and functional consequences of the N-terminal disulfide bond under different redox conditions.

Specifically, treatment of purified wild-type *At*AMT1;1 with high concentrations of DTT caused the predominant oligomeric peak in mass photometry analyses to disappear, whereas subsequent addition of H_2O_2 substantially restored the major peak distribution **(Extended Data Fig. 8 of the revised manuscript, and is also presented below)**. In parallel, yeast complementation assays showed that DTT inhibited the growth of yeast

expressing wild-type *At*AMT1;1 much more strongly than those expressing the C24S mutant **(Fig. 3e of the revised manuscript, and is also presented below)**, further supporting a functional role of the disulfide bond in maintaining transporter activity under reducing conditions.

We attempted to further evaluate the effects of reducing and oxidizing conditions on transporter activity using yeast complementation assays and stable-isotope uptake experiments. However, during these experiments we found that the concentrations of H_2O_2 required to induce measurable oxidative effects caused severe cellular damage. Under these conditions, yeast growth was strongly inhibited and cells used for isotope uptake assays largely lost viability and transport capacity, making it difficult to reliably distinguish direct effects on AMT1;1 activity from nonspecific oxidative toxicity. We also attempted to directly compare the thermal stability of *At*AMT1;1 under oxidizing and reducing conditions. However, we found that the reducing reagents strongly interfered with the fluorescence-based thermal stability assay, resulting in abnormal fluorescence signals and preventing reliable Tm determination. We therefore did not include these data in the revised manuscript.

Together, these additional experiments support the idea that the conserved extracellular disulfide bond is redox sensitive and may reversibly modulate the structural stability and activity of *At*AMT1;1.

Extended Data Fig. 8 of the revised manuscript:

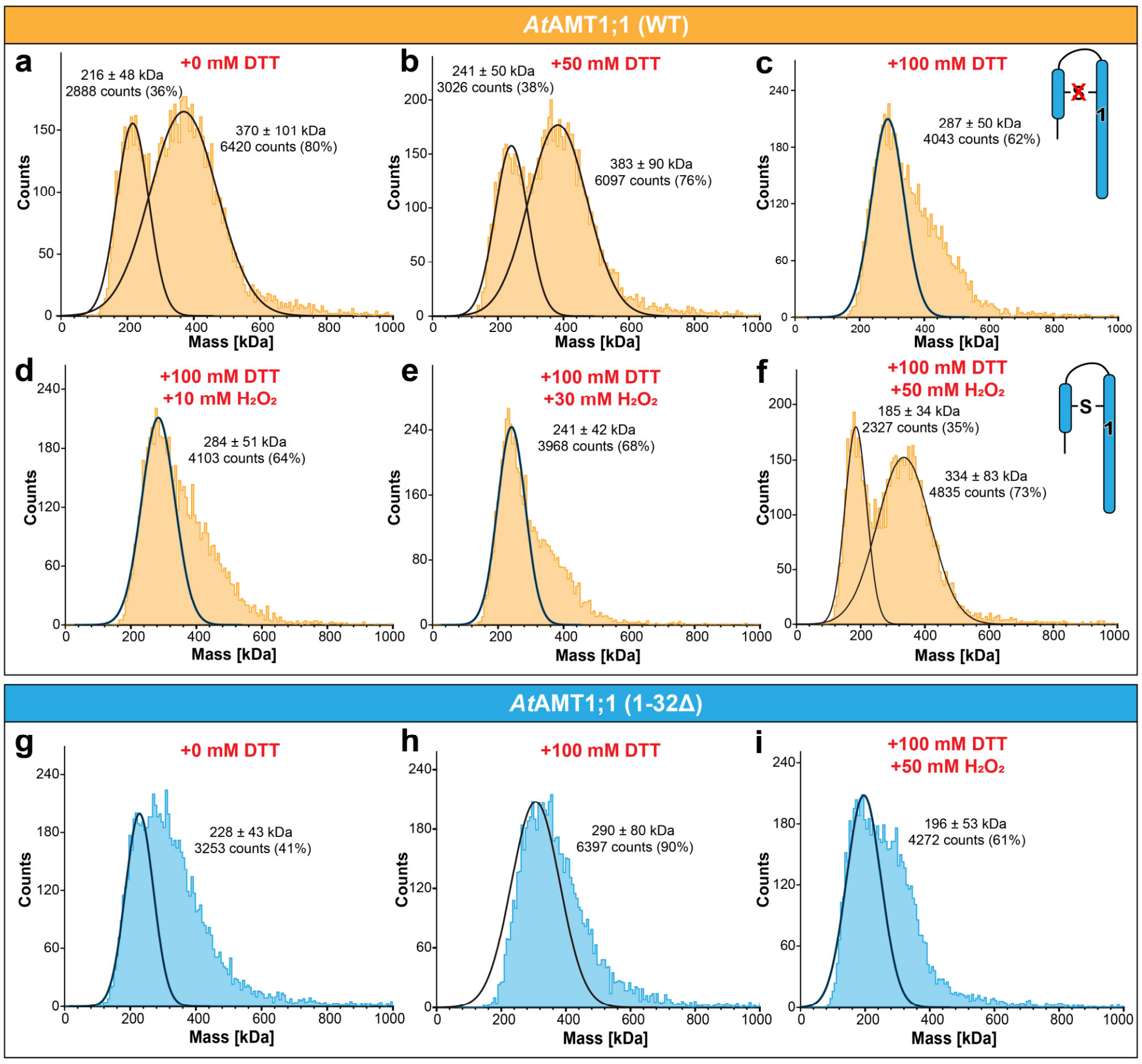

Fig. 3e of the revised manuscript:

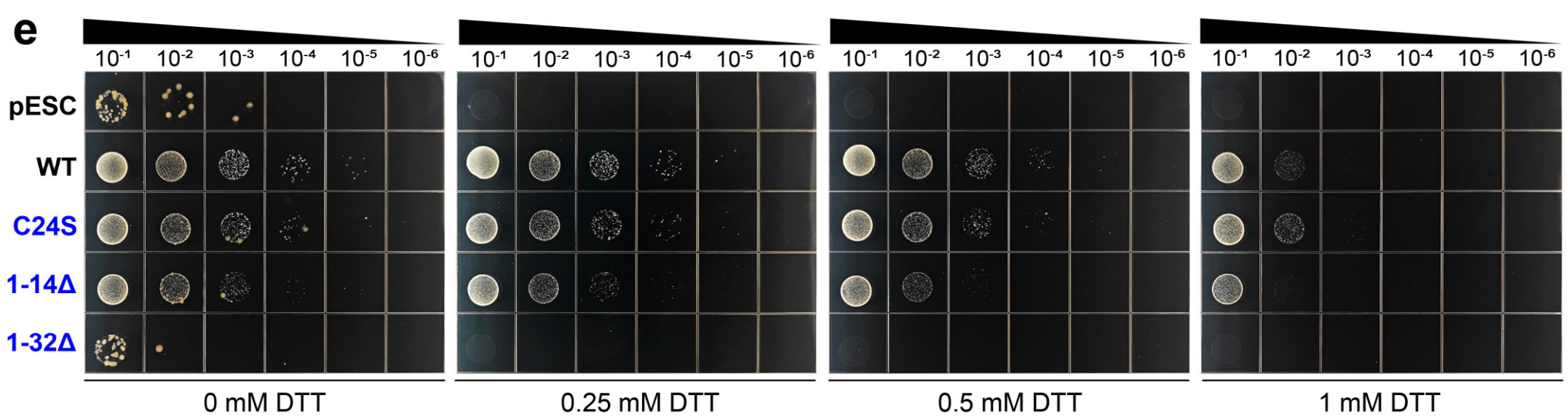

d) When plant cells expand in preparation for division, the apoplast becomes more acidic (pH 4.5 -5.5). Is this N-term a sensor for that? In a recent publication (Pflueger et al, Science Advances, 2024), it is suggested that plants AMT1 can be ammonium transporters and receptors (transceptors) because they appear to shave a signature motif for a binding pocket that binds two ammonium cations as signal. The authors don't look into this interesting hypothesis, yet they have structures and indeed these show some unassigned density near these residues in some (interestingly not all) cases.

This should clearly be analyzed and discussed as it is a unique opportunity and the first time the structure of AMT1;1 is presented.

Response: We thank the reviewer for raising this interesting point. We agree that extracellular pH changes represent an important physiological signal in plants. However, based on our current structural and computational analyses, we do not favor the idea that the N-terminal disulfide-containing region directly functions as a pH sensor. First, comparison of the structures determined under neutral and acidic conditions revealed no obvious conformational differences in the N-terminal region. Second, pKa calculations for the N-terminal residues showed that none of the residues within the first 40 amino acids exhibited predicted pKa values within the physiologically relevant pH range (~5–7), arguing against direct protonation-dependent switching within this region. We therefore interpret the N-terminus primarily as a structural and potentially redox-sensitive regulatory element rather than a direct pH sensor.

We also appreciate the reviewer's suggestion regarding the recent work by Pflueger et al. (Science Advances, 2024). In that study, the authors characterized *Shewanella denitrificans* Amt1 (Sd-Amt1), an unusual Amt-family protein fused to a C-terminal diguanylate cyclase signaling domain, and proposed that it may function as both a transporter and receptor ("transceptor") through ammonium binding at regulatory sites. In the revised manuscript, we have now carefully analyzed and discussed the potential transceptor-like properties of plant AMT1 transporters. Structural comparison revealed that *At*AMT1;1 shares substantial similarity with the bacterial transceptor *Sd*-Amt1, including conservation of several residues surrounding the proposed N1 and N2 ammonium-binding sites **(Extended Data Fig. 16, d-f)**. However, unlike *Sd*-Amt1, no obvious substrate-associated densities were observed at the equivalent N1 and N2 positions in *At*AMT1;1, and residues surrounding these regions did not exhibit clear conformational rearrangements among the *At*AMT1;1 in different states **(Extended Data Fig. 16, g-i)**. Therefore, while our current data do not provide direct evidence that plant AMT1;1 functions as a transceptor, the observed structural conservation suggests that this possibility cannot be excluded. We have now included a dedicated discussion of this hypothesis and its limitations in the revised Discussion section.

Extended Data Fig. 16 of the revised manuscript:

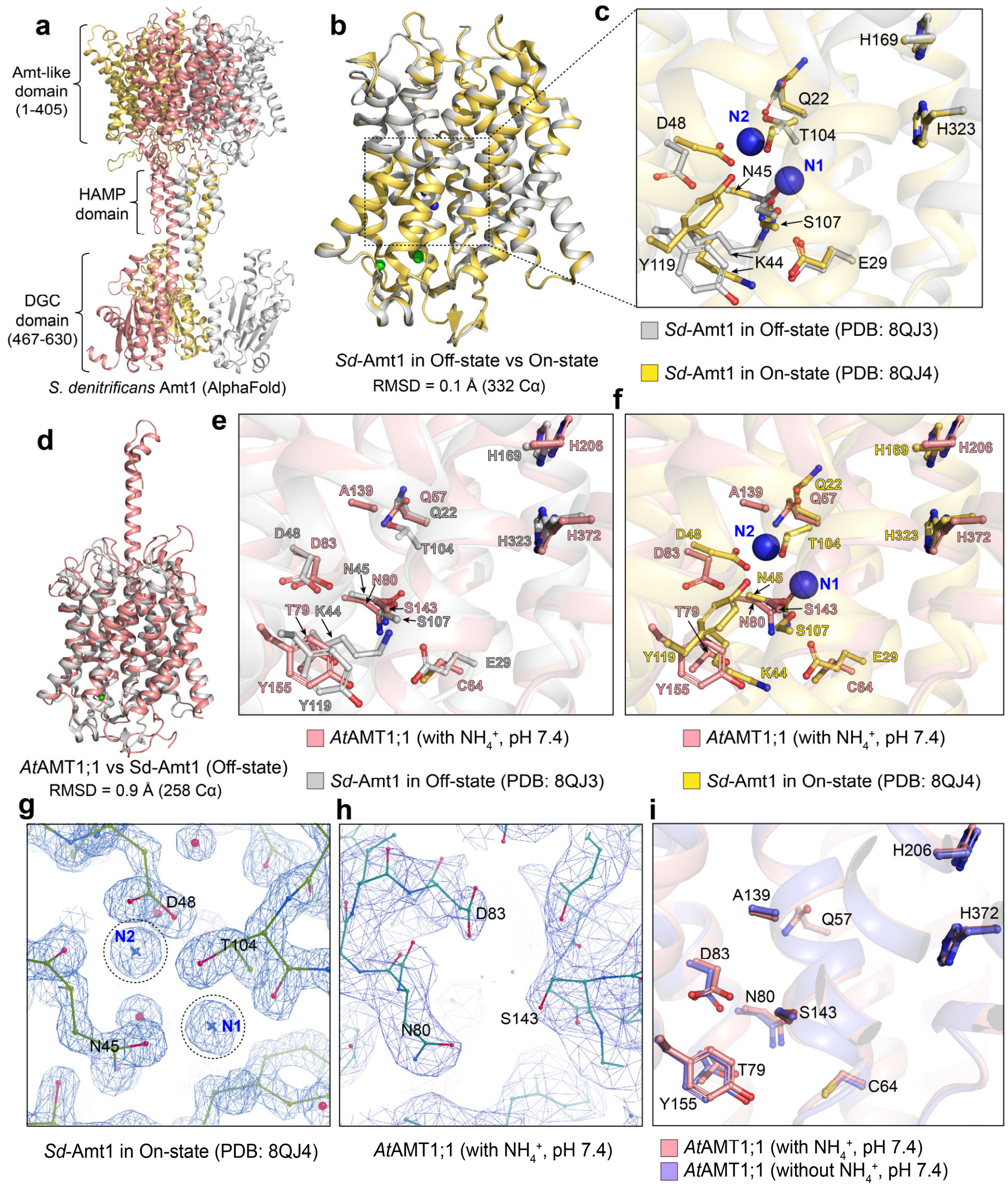

3) Transport mechanism of *Arabidopsis thaliana* AMT1;1.

a) The author's perception of the mechanistic hypothesis for Amt proteins and the role of conserved pore-lining residues (in Amts) is outdated and/or incorrect.

Response: We thank the reviewer for this important comment. In the original version, our discussion of the transport mechanism and the roles of several conserved pore-lining residues was indeed overly simplified and did not sufficiently reflect the current complexity and ongoing debate in the Amt/Mep/Rh field.

In the revised manuscript, we substantially re-analyzed and rewrote the sections related to the substrate conduction pathway **(Lines 273-329)**. We performed more detailed structural comparisons with bacterial AmtB and fungal Mep2 transporters and carefully re-evaluated the potential roles of conserved residues along the transport pathway. Importantly, we no longer make definitive claims regarding whether *At*AMT1;1

transports NH_4^+ or NH_3. Instead, we focus on the structural organization of the conduction pathway and the functional contribution of key conserved residues identified through mutagenesis and transport assays.

We also expanded the discussion of the Twin-His motif, Phe-gate residues, extracellular recruitment region, and cytoplasmic exit architecture in the context of previous mechanistic models proposed for bacterial and fungal homologs. We believe these revisions provide a more balanced and up-to-date interpretation of the data and better position our findings within the current understanding of the Amt/Mep/Rh transporter family.

b) The E coli structure (in ref 18, but not ref 19) is misleading and anyone working in the field (especially with structural knowledge) should see that. The structure described by Khademi et al is not of the native AmtB but of a mutant (near the outside vestibule) that likely altered the W/S vestibule environment-note that the second, and native E coli structure (ref 19) does not see this enlarged density). The functional assay, in the same infamous publication (ref 18), has also been broadly criticized in the community (not reproducible, simplistic interpretations) caused serious damages to the field. In essence: the gas channel hypothesis has been dismissed (see next point).

Response: We thank the reviewer for this important clarification regarding the early E. coli AmtB structural studies and the historical interpretation of the gas-channel hypothesis. We agree that the original Khademi et al. structure has important limitations and that the enlarged vestibular density observed in that study may have been influenced by the mutant background used for crystallization. We also acknowledge that subsequent structural and functional studies, including the later native AmtB structure, did not reproduce several of the original observations.

In the revised manuscript, we substantially revised the Introduction and Discussion to better reflect the current understanding and ongoing debate in the field. In particular, we removed several overly simplified statements related to the classical "NH_3 gas channel" model and no longer interpret our data within a strict NH_3-only transport framework. Instead, we now discuss multiple mechanistic models proposed for Amt/Mep/Rh transporters and focus primarily on the structural features of the substrate

conduction pathway and conserved pore-lining residues.

Importantly, we avoid making definitive conclusions regarding whether *At*AMT1;1 transports NH_4^+, NH_3, or coupled species. Rather, our structural and mutational analyses are now discussed in the context of substrate recruitment, pore coordination, and conformational regulation. We believe these revisions provide a more balanced and up-to-date interpretation of the data.

c) At points (but curiously not always), the authors write that the mechanism of ammonium transport by Arabidopsis thaliana AMT1;1 is unknown. But in ref 17 (and in other publications by the same or by other groups), Mayer and Ludewig show that AMT1;1 acts as an electrogenic, pH-independent uniporter for NH_4^+, in contrast with the (then proposed) gas channel idea (ref 18). Meanwhile, an electrogenic assay was developed and shown to be a reliable tool to effectively monitor prokaryotic Amt transport. In that work (Wacker et al, 2014, PNAS), two Amts from Archaeoglobus fulgidus were shown to be electrogenic and subsequently (Mirandela et al, 2019, The Faseb Journal,) the same assay was applied to E. coli AmtB confirming that this protein is also not a NH3 gas channel.

Response: We thank the reviewer for this important comment and fully agree that previous electrophysiological studies have provided strong evidence that *Arabidopsis* AMT1;1 functions as an electrogenic, pH-independent NH_4^+ transporter. In the original version, our wording regarding the transport mechanism was imprecise and did not sufficiently acknowledge these important studies.

In the revised manuscript, we substantially updated the Introduction and Results to better reflect the current state of the field (Introduction: **Lines 49-64** and Results: **Lines 214-216**). Specifically, we now discuss the electrophysiological work from Mayer, Ludewig and colleagues, as well as the later SSM-based electrophysiological studies on prokaryotic Amt proteins, which collectively argue against a simple NH_3 gas-channel model.

Importantly, in the revised manuscript we no longer present the classical gas-channel model as the favored mechanism for *At*AMT1;1. Instead, we focus primarily on the structural organization of the transport pathway and the roles of conserved residues

involved in substrate coordination and transporter regulation.

Given the sequence similarities in pore lining residues between E. coli AmtB, Archaeoglobus fulgidus and many others, with the Arabidopsis thaliana AMT1;1, this reviewer would assume that, most likely, they share the same basic transport principles. Please discuss the data obtained in view of these updates and considering advancements on the role of functional important residues. For example, W/S were shown to delay transport (Wood et al, 2008, JBC), MD simulations on F/F showed that the phenyl groups just have to tilt to allow cations to pass (Lin et al, 2006, JACS) and H/H may act to deprotonate and re-protonate NH_4^+ (Ullmann et al, 2012, J Phys ChemB).

Response: We thank the reviewer for this important suggestion. We agree that the strong conservation of pore-lining residues among bacterial, archaeal, fungal, and plant Amt/Mep transporters strongly supports the idea that these proteins share common core transport principles despite differences in regulatory mechanisms.

In the revised manuscript, we substantially expanded the discussion of the conserved transport-pathway residues and reinterpreted our mutagenesis results in the context of previous biochemical, electrophysiological, structural, and computational studies. Specifically, we now discuss the possibility that the conserved W/S vestibule primarily modulates substrate recruitment and transport kinetics rather than functioning as a strict selectivity filter. In agreement with this model, mutations of the corresponding residues in *At*AMT1;1 only moderately affected transport activity.

We also revised the discussion of the conserved Phe-gate residues in light of previous molecular dynamics simulations suggesting that transient tilting or rotamer rearrangement of the phenylalanine side chains is sufficient to permit substrate passage without requiring large-scale pore opening (Lin et al, 2006, JACS) (Yang et al, 2007, Biophys J). Consistent with this idea, our data showed that single Phe-gate mutations retained substantial transport activity, whereas simultaneous disruption of both phenylalanines strongly impaired transport, supporting a cooperative gating mechanism.

Finally, we expanded the discussion of the Twin-His motif and now discuss our findings together with previous computational studies proposing that the conserved histidines may participate in substrate coordination and potentially deprotonation/reprotonation

events during transport (Ullmann et al, 2012, J Phys ChemB).

We have revised the corresponding text accordingly **(Lines 287-313)**.

4) Assignment of NH_3, NH_4^+ or Am

If intending to propose a mechanism of ammonium transport, the simplification to Am, is not beneficial. And this assignment is extremely difficult to do (as broadly reported in the literature).

Response: We thank the reviewer for this important comment and fully agree that unambiguous assignment of NH_4^+ or NH_3 within Amt/AMT transport pathways remains extremely challenging and continues to be actively debated in the field.

In the revised manuscript, we therefore avoid making definitive mechanistic claims regarding the exact transported chemical species. Instead, to avoid overinterpretation of the cryo-EM densities and to simplify structural description of the conduction pathway, we now use the general term "Am" when referring to substrate-related densities or transport intermediates in the pore.

a) Densities below the second H in the pore have been seen in previous structures. If you make an assignment, justify it based on data (not only the structure). If not, leave it as water as many others have done and explain it.

Response: We thank the reviewer for this important comment. We agree that assignment of the density below the second histidine within the pore is challenging and should not rely solely on structural interpretation. In the revised manuscript, we therefore substantially toned down our interpretation and now discuss this density more cautiously.

To better evaluate this feature, we re-analyzed the cryo-EM maps at multiple contour levels and systematically compared the corresponding densities with previously reported bacterial and fungal Amt/Mep structures **(As now shown in Extended Data Fig. 11, and is also presented below)**, a similar density near the conserved Twin-His region is clearly present in several bacterial AmtB structures, including the NH_4^+-bound structure reported by Khademi et al. (Science, 2004), where it was interpreted as an ammonium molecule. However, in the other bacterial AmtB structural research (PNAS, 2004), the equivalent density was not explicitly assigned. In contrast, corresponding

densities are absent in fungal Mep2 structures (Nature Communications, 2016), likely due to partial occlusion of the pore near the Twin-His region.

Importantly, in our study the density persisted in multiple *At*AMT1;1 structures, including apo, ammonium-bound, and MeA-bound conditions, but disappeared in the H372F mutant, indicating that the conserved histidine contributes to stabilization of the bound species. Nevertheless, because the current resolution and available biochemical data do not allow unambiguous identification of the molecule, we now avoid definitive assignment as NH_4^+. Instead, we state that the density most likely corresponds to a substrate-related molecule trapped near the Twin-His region, while also acknowledging that it could alternatively represent an ordered water molecule.

We have revised the corresponding text accordingly **(Lines 236-250)**.

Extended Data Fig. 11 of the revised manuscript:

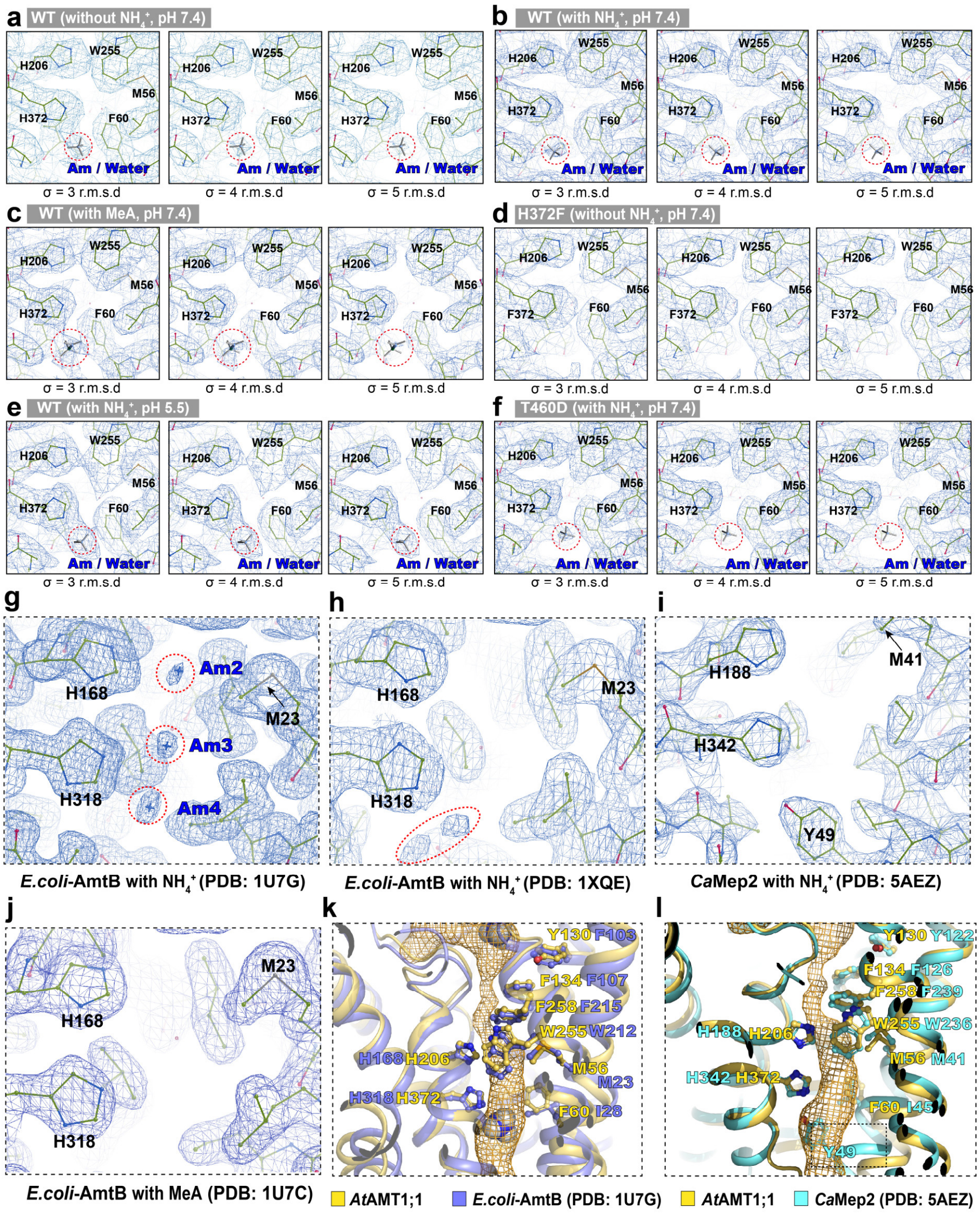

b) Why/how other map features are assigned as NH_3 or NH_4^+ in the structures and in Fig 7?

Response: We thank the reviewer for this important comment. We agree that unambiguous assignment of NH_3 or NH_4^+ based solely on cryo-EM densities is not possible at the current resolution. In the revised manuscript, we therefore carefully revised both the main text and Fig. 7 to avoid overinterpretation of the substrate-related densities.

Specifically, we no longer explicitly assign the observed densities as NH_3 or NH_4^+ in the structural models or mechanistic schematic. Instead, we now use the more general term “Am” or “substrate-related density” when discussing the conduction pathway. The corresponding figure legends and mechanistic descriptions have also been revised

accordingly.

c) There are basically two theories on where and how internal deprotonation of NH_4^+ to NH_3 occurs. At the same time, it seems reasonable that a H^+ should not be left outside or be consumed inside in the process (as is suggested on the end of p. 15). Instead, an internal H^+ pathway may exist, for example, provided by H/H (see Ullmann, J Phys ChemB, 2012 or others). The hypothesis that positively charged residues in the cytoplasmic face of the protein would give a H^+ to NH_3 implies that a H^+ would be used from the cytoplasm to regenerate their state. This does not seem likely (Andrade et al, Mol Memb Biol, 2007). In view of this, could the effect on transport caused by these mutations be the structural displacement of cytoplasmic regions?

Response: We thank the reviewer for this important and insightful comment. We agree that the hypothesis proposing direct proton donation from positively charged cytoplasmic residues to NH_3 is difficult to reconcile with current mechanistic models and may not be energetically favorable. In the revised manuscript, we therefore removed the previous speculative discussion suggesting that these positively charged residues directly participate in proton transfer or reprotonation events during transport. More importantly, we substantially revised our interpretation of the cytoplasmic loop mutants. Rather than proposing a direct role in proton conduction, we now interpret the functional defects primarily in the context of structural and allosteric regulation. Our structural analyses revealed that the intracellular positively charged loops (L1, L3, and L5) form an extensive interaction network with the C-terminal regulatory domain (CRD), particularly surrounding the conserved phosphorylation site T460 **(As now shown in Fig. 6, c-g, and is also presented below)**. Consistently, phosphomimetic mutation weakened the cryo-EM density of the CRD-associated region and increased local flexibility of the L5 loop in MD simulations without inducing major global conformational rearrangements **(As now shown in Extended Data Fig. 14e, g, and is also presented below)**.

Importantly, we further compared these findings with previously reported bacterial AmtB and fungal Mep2 structures **(As now shown in Extended Data Fig. 15, and is also presented below)**. In both systems, intracellular loop regions occupying analogous

positions have been implicated in transporter regulation rather than direct proton transfer. In bacterial AmtB, GlnK binding induces substantial rearrangement of intracellular loops associated with transport inhibition, whereas in fungal Mep2, phosphorylation-dependent regulation has been proposed to involve intracellular loop-mediated autoinhibitory mechanisms. Together, these observations support the idea that the positively charged intracellular loops represent a conserved allosteric regulatory module within the AMT/MEP/Rh superfamily, and that the effects of the mutations observed in our study most likely arise from perturbation of this regulatory interaction network and local loop organization rather than direct participation in proton transfer chemistry.

Fig. 6, c-g of the revised manuscript:

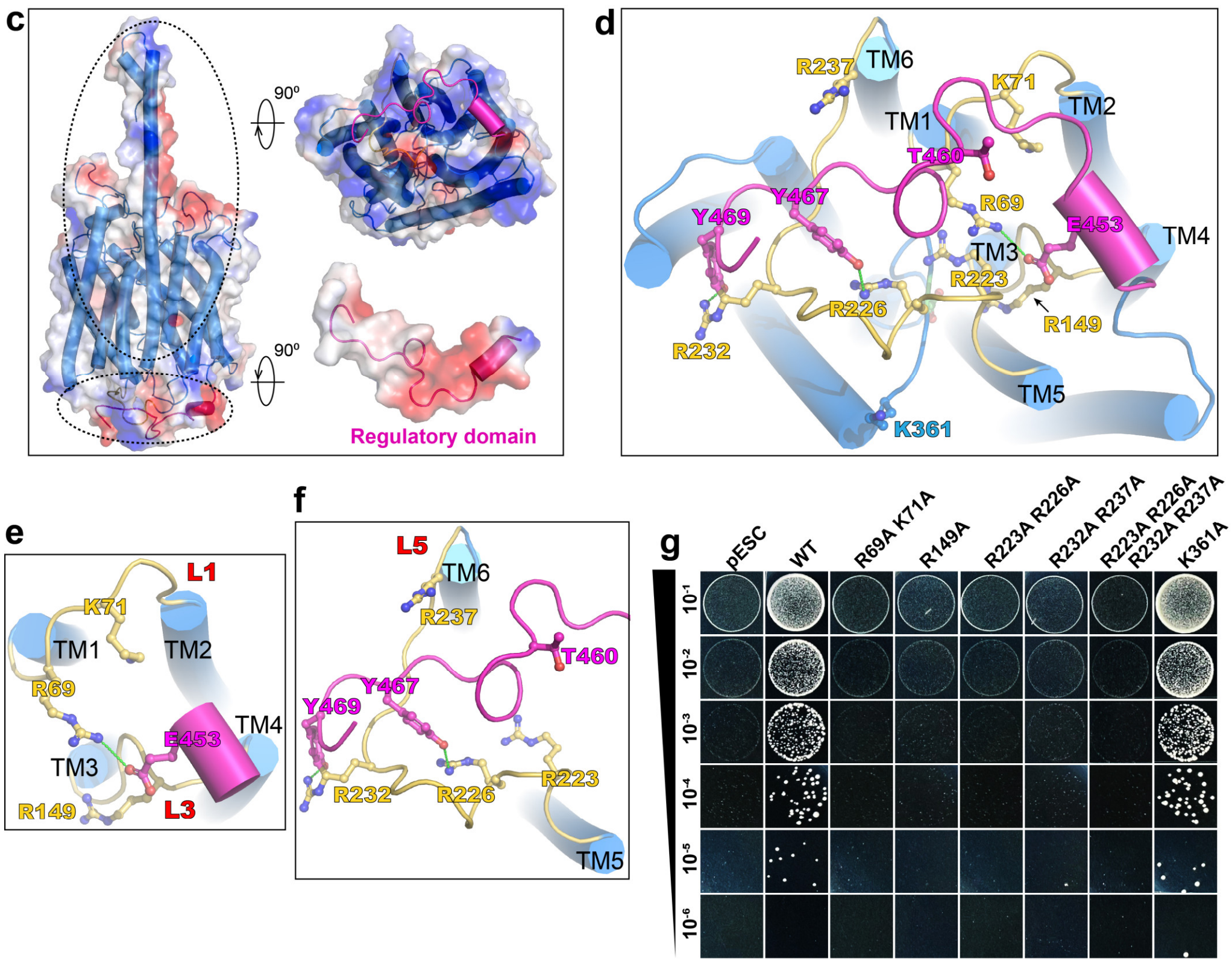

Extended Data Fig. 14e, g of the revised manuscript:

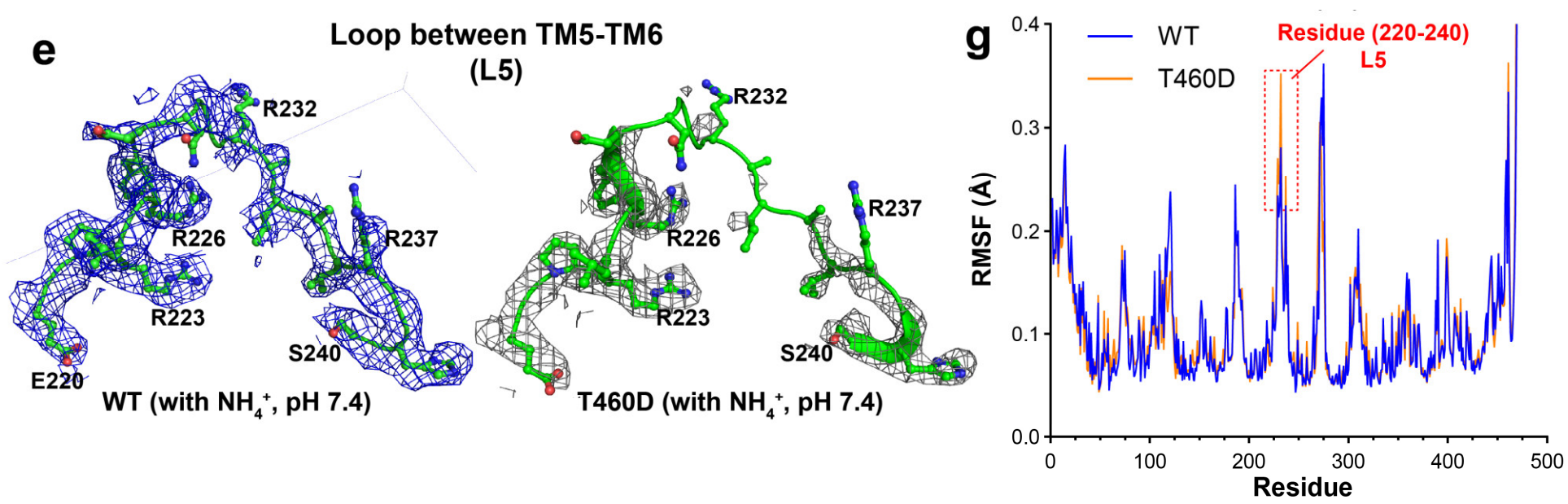

Extended Data Fig. 15 of the revised manuscript:

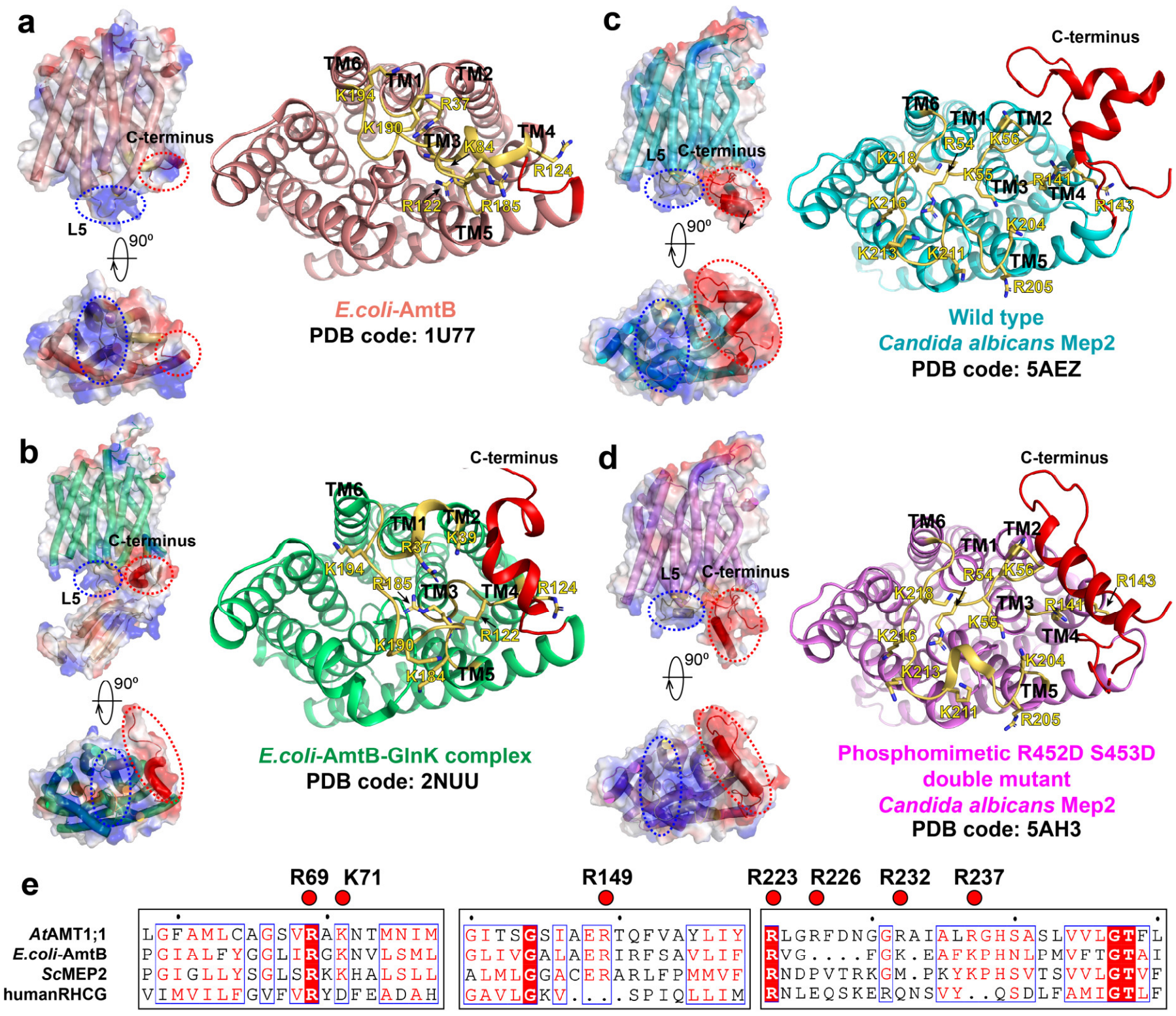

d) It is not clear why so few molecular structural details are shown. Figures in general are very global, simplistic and do not show details. The authors do not really describe with sufficient detail the very interesting structural changes that occur in the intracellular face of AMT1;1 from the various structures shown. Above all, they do not establish a cause-effect.

Response: We thank the reviewer for this important suggestion. We agree that the intracellular structural rearrangements and their functional implications were insufficiently illustrated and discussed in the original version of the manuscript.

In the revised manuscript, we substantially redesigned the structural figures and added more detailed views of the intracellular interaction network, particularly around the C-terminal regulatory domain (CRD) and the positively charged intracellular loops. The

updated figures now more clearly show the electrostatic and hydrogen-bonding interactions between the CRD and the L1, L3, and L5 loops, as well as the structural differences between the wild-type and T460D phosphomimetic mutant **(As now shown in Fig. 6, c-g, and also see above)**.

Importantly, we also expanded the mechanistic discussion to better establish a potential cause–effect relationship between phosphorylation and transporter inhibition. Combined with mutagenesis, cryo-EM density analysis, and molecular dynamics simulations, our data now support a model in which phosphorylation at T460 perturbs the CRD-loop interaction network, increases local loop flexibility—particularly within L5—and thereby allosterically modulates transporter activity without inducing large-scale conformational rearrangements. We believe these revisions provide a clearer and more mechanistically grounded interpretation of the intracellular regulatory architecture of *At*AMT1;1.

e) P10/11, l194-198: please clearly show the density for this position and the assigned methylammonium. What is the structural confidence of this assignment after refinement? If the authors choose to cite ref 17 here, please mention that the structure is of a mutant.

Response: We thank the reviewer for this important comment. We agree that assignment of methylammonium or ammonium at the extracellular recruitment site requires careful interpretation and should not rely solely on the presence of additional cryo-EM density. In the revised manuscript, we therefore re-analyzed the corresponding densities at multiple contour levels and substantially expanded the structural comparison with previously reported bacterial and fungal Amt/Mep structures. **As now shown in Extended Data Fig. 12 and is also presented below**, similar densities adjacent to the conserved W/S recruitment region are observed in both bacterial AmtB and fungal Mep2 structures. In the methylammonium-bound AmtB structure reported by Khademi et al. (Science, 2004; mutant background), the elongated density at this position was interpreted as bound methylammonium, whereas in the ammonium-bound structures the density was assigned to ammonium. In contrast, the other bacterial AmtB structures conservatively modeled this density as ordered water molecules (PNAS, 2004).

Extended Data Fig. 12 of the revised manuscript:

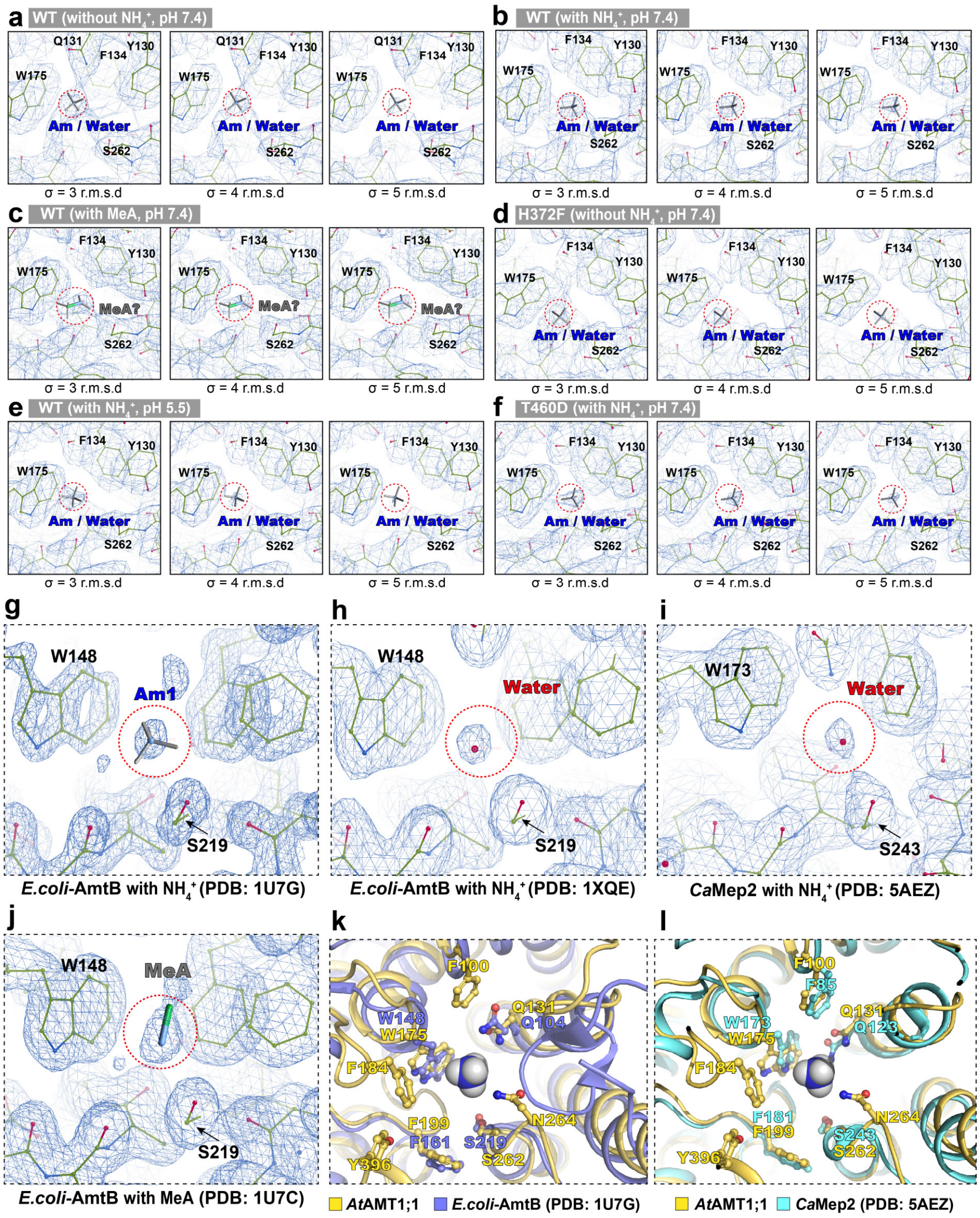

In our structures, the density near W175 and S262 is consistently observed under apo, ammonium-bound, and methylammonium-bound conditions. Notably, the density in the methylammonium-bound structure displays a more elongated shape compared with the ammonium-bound condition, resembling the previously reported methylammonium-associated density in bacterial AmtB. However, because the current resolution does not allow unambiguous discrimination between methylammonium, ammonium, and coordinated water molecules, we now avoid definitive assignment in the revised manuscript. Instead, we describe these features more cautiously as substrate-associated or solvent-associated densities within the conserved extracellular recruitment vestibule. In the revised manuscript, we now explicitly clarify that the methylammonium-associated bacterial AmtB structure reported by Khademi et al.

(Science, 2004) was determined using a mutant background rather than native wild-type AmtB. This clarification has been added to the main text when discussing the similarity between the elongated density observed in our MeA-bound *At*AMT1;1 structure and the previously reported AmtB methylammonium-associated density **(Lines 259-268)**.

5) P.11 l200: where is the high conservation of residues at the pore exit? This exit vestibule is broad and formed by many residues. Therefore, it is not clear in neither of the supplemental figures mentioned, the identity of these residues. Please also clarify why they are considered relevant, in the context of the sentence.

Response: We thank the reviewer for this important comment. After careful re-analysis of the sequence conservation and structural features of the intracellular exit region, we agree that the overall conservation in this region is substantially lower than we originally implied. In the revised manuscript, we therefore revised the wording and expanded the structural description of the pore exit architecture **(As now shown in Fig. 4e, and is also presented below)**.

Fig. 4e of the revised manuscript:

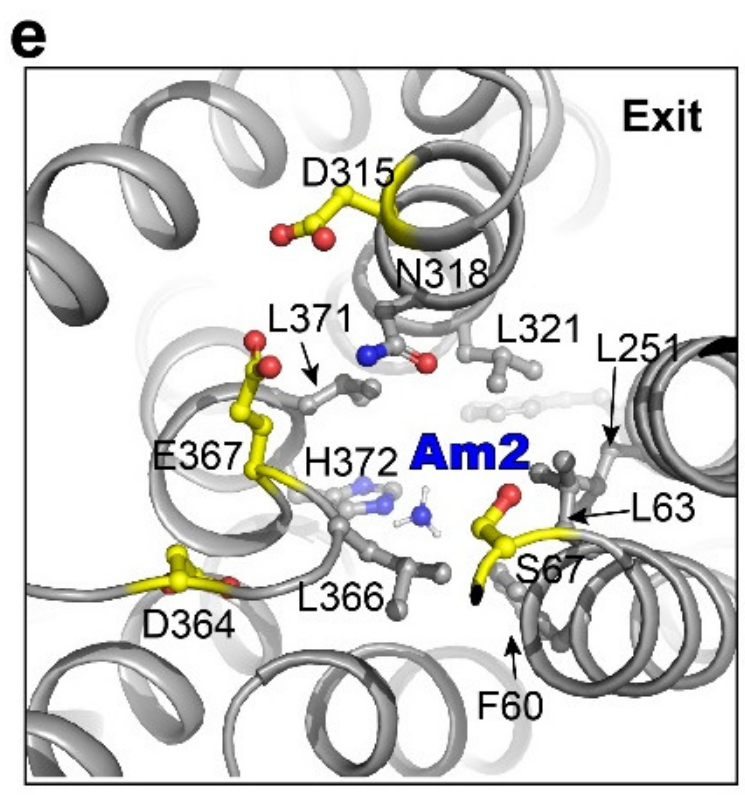

Specifically, we now distinguish between the conserved hydrophobic environment immediately below the Twin-His region and the more variable outer cytoplasmic vestibule. Our structural comparison showed that residues directly beneath H372 form a relatively hydrophobic region similar to those observed in bacterial AmtB and fungal Mep2 **(As now shown in Extended Data Fig. 13 and is also presented below)**. In contrast, the outer exit vestibule of *At*AMT1;1 is composed of several negatively charged and polar residues that are poorly conserved in bacterial AmtB but more closely

resemble the corresponding region in fungal Mep2. We also clarified why these residues were discussed. Their relevance does not stem from strict sequence conservation, but rather from their distinct electrostatic and structural characteristics at the cytoplasmic exit region. In particular, comparison with fungal Mep2 suggested that this region may contribute to modulation of exit-pathway geometry and transporter regulation, as the corresponding region in Mep2 adopts a partially occluded conformation associated with an inhibited state. These points are now more carefully discussed in the revised manuscript and corresponding supplementary figures.

Extended Data Fig. 13 of the revised manuscript:

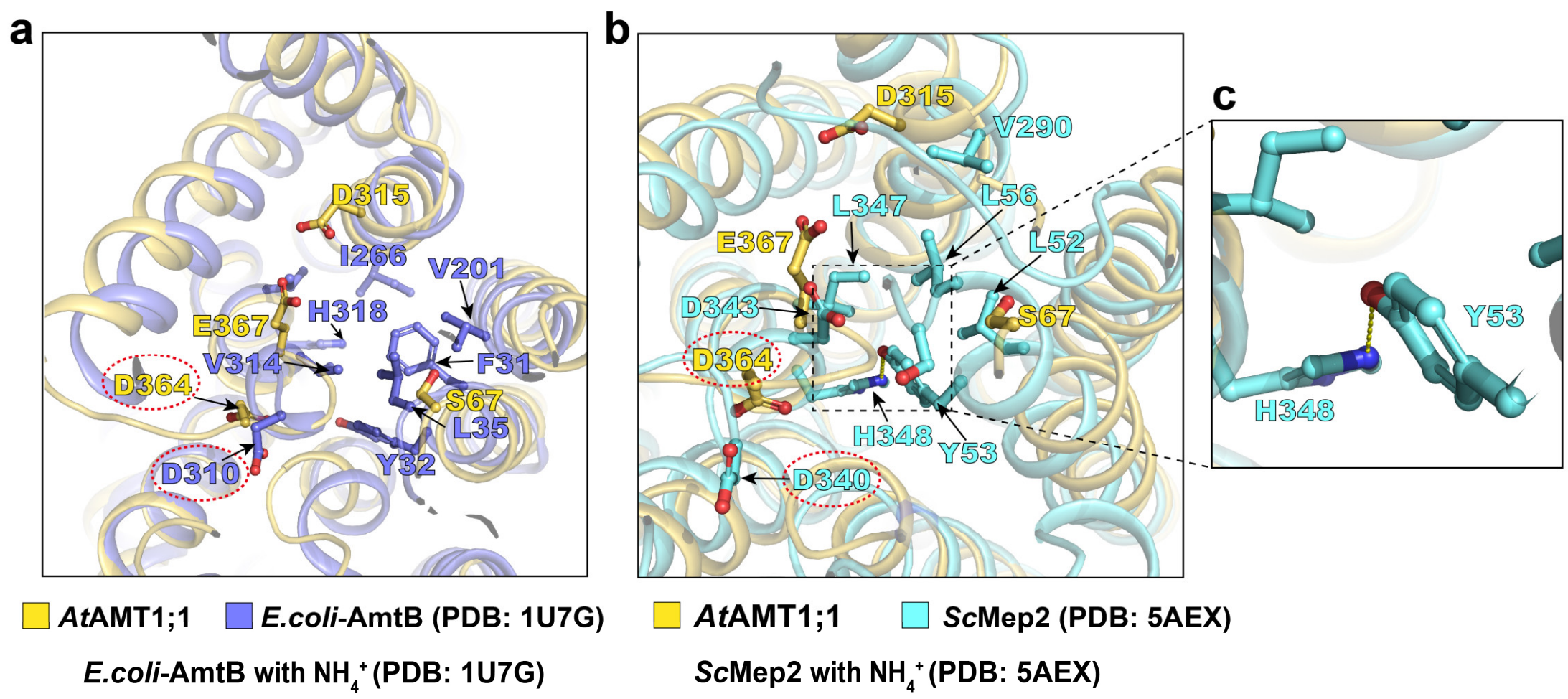

6) When introducing mutations, the rationale behind is not always clear. This happened eg with the H/F single or double mutations (previously shown by several researchers to inhibit transport), or the S262, D364, etc. Give a context to the mutated sites.

Response: We thank the reviewer for this helpful suggestion. In the original manuscript, the rationale underlying several mutational designs was indeed insufficiently explained. In the revised version, we clarified the basis for residue selection and mutation strategy throughout the Results section. Briefly, most residues were selected based on their structural positions within the substrate conduction pathway or regulatory interaction network. Conserved residues lining the pore entrance, Twin-His region, Phe-gate, and intracellular regulatory interfaces were systematically analyzed by mutagenesis to evaluate their contributions to transport activity and regulation. In most cases, residues were mutated to alanine to minimize side-chain interactions while preserving overall backbone structure. For the conserved Twin-His motif, we introduced histidine-to-

phenylalanine substitutions rather than alanine substitutions because phenylalanine preserves the aromatic character and steric environment of the pore while eliminating the protonatable imidazole group. This strategy has been widely used in previous functional studies of Amt/Mep transporters to specifically probe the mechanistic role of the Twin-His motif in substrate coordination and transport.

We have now added these explanations to the revised manuscript to provide clearer context for the mutational analyses.

7) Twin-His to F single and double mutations, W175, F/F to A single and double mutations, as well as C-term deletions, have been done for AMT1;1 (ref 29; Yuan et al, JBC 2009; ref 28, Ortiz-Ramirez et al, 2011, JBC; etc) or for homolog proteins. Please cite relevant previous work and discuss the results accordingly (are the results the same or not and what that means).

Response: We thank the reviewer for pointing this out. In the revised manuscript, we carefully incorporated and discussed the relevant previous studies involving Twin-His, W175, Phe-gate, and C-terminal truncation mutants in *At*AMT1;1 and related Amt/Mep homologs. These revisions provide clearer historical context and better position our structural and functional findings within the broader framework of prior AMT/MEP transporter studies.

8) P12 l 223-227: The conclusion that NH_3 passes the F/F site lacks justification, considering the results obtained and the existing electrophysiological data for this protein (ref 17)

Response: We thank the reviewer for this important comment and agree that our original wording overinterpreted the structural observations. Based on the currently available structural and electrophysiological data, our results do not allow us to conclude that NH_3 specifically passes through the Phe-gate region. In the revised manuscript, we therefore substantially toned down this statement. We now discuss the conserved Phe-gate more cautiously as a dynamic aromatic constriction within the central conduction pathway rather than proposing a specific NH_3 permeation mechanism. We also incorporated the electrophysiological evidence from previous studies on *At*AMT1;1, which supports electrogenic transport properties inconsistent with a simple NH_3 gas-

channel model.

9) Please mention the orientation in the membrane of the helices shown in Supplemental Figs 7 and 8. (Why is the cytoplasm always up?)

Response: We thank the reviewer for pointing this out. In the original figures, the membrane orientation was not consistently indicated, which could indeed cause confusion regarding the cytoplasmic and extracellular sides of the transporter. In the revised manuscript, we adjusted the orientation of the transmembrane regions in the relevant figures so that the extracellular side is consistently shown at the top and the cytoplasmic side at the bottom throughout the manuscript.

10) What was the rational for choosing pH 6.0 (above the apoplast pH)? When interpreting data at lower pH, the hypothesis that pH can affect the protonation state of particular residues with structural consequences is not discussed. Couldn't this simply be the case?

Response: We thank the reviewer for this important suggestion. We agree that pH 6.0 is still higher than the typical acidic conditions of the plant apoplast. To better address this concern, we subsequently determined an additional structure under pH 5.5 conditions, which more closely approximates the physiological extracellular environment. Importantly, the structure (pH 5.5) was highly similar to the previously determined at pH 6.0 **(A comparison of the EM densities is presented below)**, and therefore, in the revised manuscript, we replaced the pH 6.0 dataset with the pH 5.5 structure throughout the study. In addition, we expanded the discussion regarding possible pH-dependent effects on *At*AMT1;1 structure and dynamics **(Lines 346-347 and 364-366)**. Specifically, we now discuss the possibility that acidic conditions may alter the protonation states of particular residues, thereby influencing local conformational flexibility or interaction networks rather than inducing large-scale global structural rearrangements. This interpretation is also consistent with our MD simulations, which revealed increased flexibility of several intracellular loop regions under acidic conditions despite the overall structural similarity between different pH states.

The comparison of EM densities between different acidic conditions (pH 6.0 vs pH 5.5):

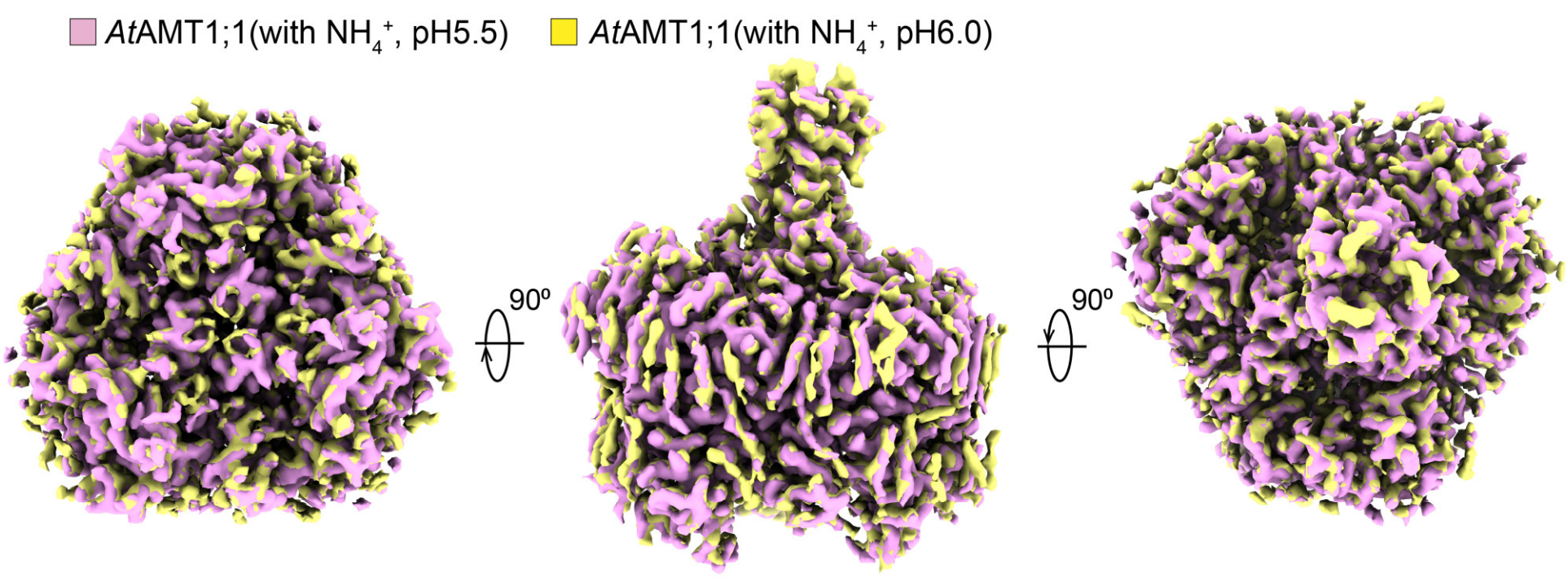

11) P15: When mentioning positively charged residues in cytoplasmic loops, it is not straightforward to understand which mutations were tested in the text. Also, single and double mutants are mentioned but double of which?

Response: We thank the reviewer for pointing this out. We agree that the organization and mutational design of the positively charged residues in the intracellular loops were insufficiently explained in the original manuscript.

In the revised version, we substantially improved the structural figures to more clearly display the positions of these positively charged residues within the intracellular L1, L3, and L5 loops, as well as their interactions with the C-terminal regulatory domain (CRD) **(As now shown in Fig. 6, d-f, and is also presented above)**. Specifically, residues including R69, K71, R149, R223, R226, R232, and R237 were selected because they cluster around the CRD-mediated interaction network. To evaluate their functional contribution, residues were mutated to alanine. In addition, for several spatially adjacent residues, we designed double or multiple alanine-substitution mutants to minimize potential functional redundancy and better assess the collective contribution of the positively charged interaction surface to transporter regulation. These clarifications have now been incorporated into the revised manuscript and corresponding figures.

12) Start of p13: What are the concrete observations that allow stating that NH_4^+ release is facilitate by a slight outward deflection of TM7-TM8? This reviewer does not see it. Maybe an improved figure should show (quantify) how much they move and detail it better. Are there amino acid interactions formed/broken because of this? Does the pore geometry change? From the figure provided, it looks like the connecting loop is what changes most?

Response: We thank the reviewer for this important comment. We agree that our original interpretation regarding substrate release facilitated by TM7–TM8 movement was overly speculative. In the revised manuscript, we therefore substantially toned down this conclusion and expanded the structural analysis. Specifically, we generated additional figures showing detailed pore-radius comparisons under different pH conditions and quantitatively indicated the displacement distances of TM7 and TM8 **(As now shown in Fig. 5, a-e, and is also presented below)**.

Fig. 5, a-e of the revised manuscript:

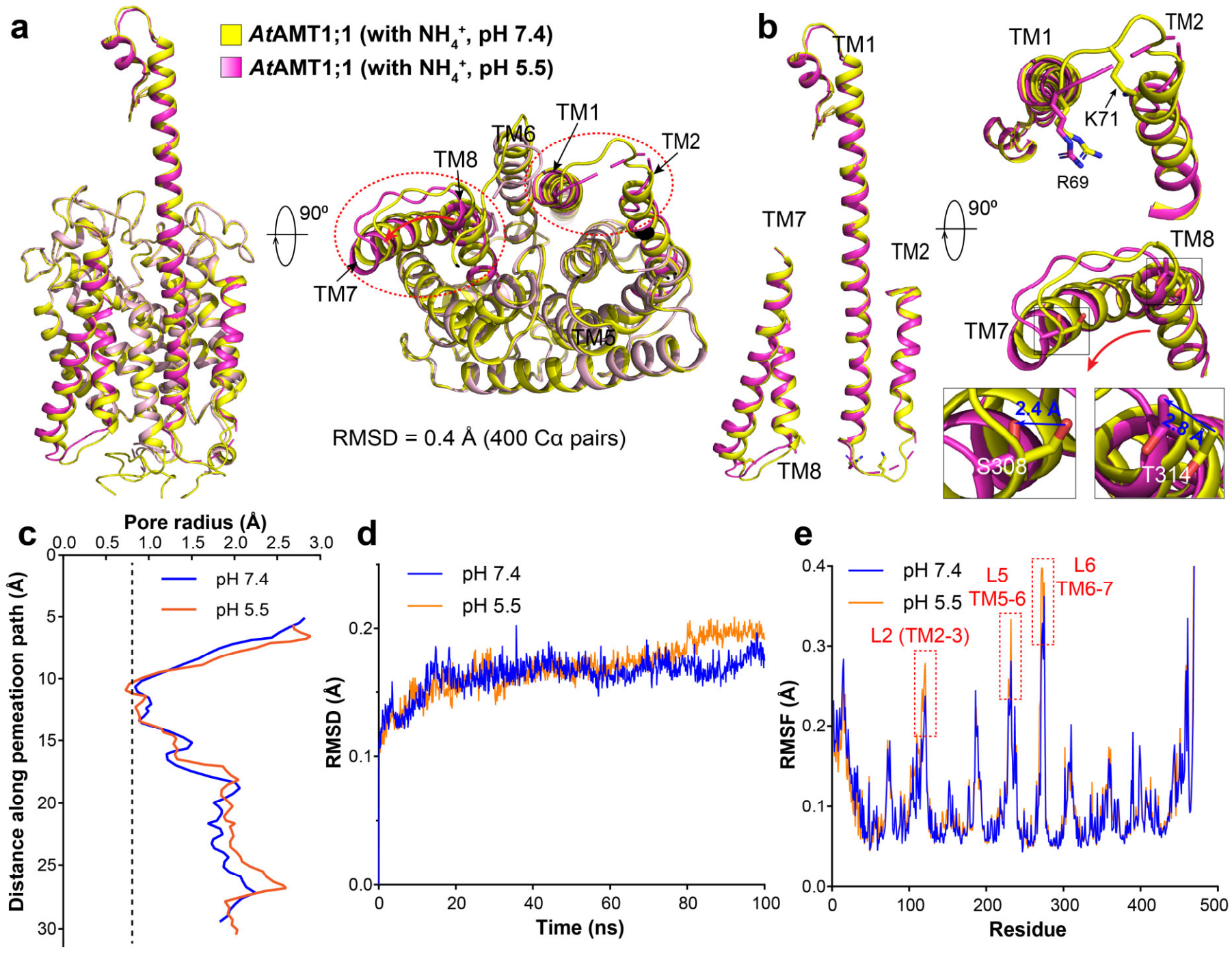

Structural alignment revealed that TM7 and TM8 undergo modest outward displacements of approximately 2.4 Å and 2.8 Å, respectively, accompanied primarily by increased flexibility of the adjacent loop regions. Pore-radius analysis showed that although the cytoplasmic exit region became slightly wider at low pH, the narrow constriction at the Phe-gate remained largely preserved. Therefore, our current data do not provide sufficient evidence to conclude that substrate release is directly facilitated by these subtle conformational changes. Instead, in the revised manuscript we now interpret these observations more cautiously and propose that extracellular pH mainly affects local conformational dynamics and loop flexibility rather than inducing a major opening transition of the transport pathway.

13) Fig 5b should depict the orientation of helices with regards to the orientation seen in a). Is the red arrow a double arrow?

Response: We thank the reviewer for pointing this out and apologize for the lack of clarity in the original figure. The arrow in Fig. 5b was intended to indicate a single-direction displacement rather than a bidirectional movement, but the presentation may indeed have caused confusion. In the revised figure, we redesigned the panel to more clearly and improved the visualization of the displacement direction. The arrow has been replaced with a clearer single-direction indicator.

14) P 13 l 244: Indeed. But what pH increase are the authors considering? Because with a pKa around 9.2 (at 20 degrees Celcius), at pH 7.0 more than 99% of the entire Am is in the protonated cationic form. At pH 8.0 ca. 94% is NH_4^+. Does this correlate with the observations?

Response: We thank the reviewer for this important and technically accurate comment. We agree that, given the pKa of the NH_4^+/NH_3 equilibrium (~9.2), the vast majority of ammonium remains in the protonated NH_4^+ form under our experimental conditions, including at pH 8.0. Therefore, our previous speculation that increased isotope accumulation in the empty-vector control at higher pH might result from passive NH_3 diffusion was not sufficiently justified.

In the revised manuscript, we have removed this interpretation and now describe the observations more cautiously. Specifically, we only report that empty-vector control cells exhibited increased $^{15}NH_4^+$ accumulation and improved growth under more alkaline conditions, whereas *At*AMT1;1-expressing cells consistently showed higher uptake at lower pH. At present, the mechanistic basis underlying the improved growth and increased isotope accumulation in control cells at elevated pH remains unclear. We therefore avoid further speculation and instead focus our interpretation on the observation that acidic conditions enhance AMT1;1-mediated ammonium uptake.

15) P13 l252-254: Please backup this sentence with the structures of the protein at pH 7.4 and 6.0, with and without ammonium provided, what structural features support this? If none are visible, rationalize reasons for that

Response: We thank the reviewer for this important comment. We agree that the original

statement was insufficiently supported by structural evidence and was overly speculative. In the revised manuscript, we carefully re-evaluated the structures determined at pH 7.4 and pH 5.5 in the presence and absence of ammonium. Overall structural alignment revealed that AtAMT1;1 adopts highly similar architectures under these conditions. Importantly, these structural changes were modest and did not provide direct evidence for a distinct substrate release or gating transition. Therefore, in the revised manuscript we substantially toned down our interpretation and now propose that extracellular pH may primarily influence local conformational dynamics and residue protonation states rather than inducing large-scale structural transitions.

16) P14 l268-275: Similar assays have been done previously on various homolog Amt proteins. Please mention, cite that and state that accordingly, for all Amts, the C-term integrity has been shown to be essential for function (see point 4d)).

Response: We thank the reviewer for this helpful suggestion. In the revised manuscript, we incorporated and discussed the relevant previous studies on C-terminal regulation in AMT transporters. We also expanded the discussion to place our C-terminal truncation and phosphorylation-related results in the broader context of conserved AMT autoregulatory mechanisms.

17) End of p14: As an important point, and to show that T460D disturbs the cytoplasmic interactions, the region becomes more flexible and the maps are therefore weaker: Supplemental Figure 12b does not show this well. Why not do something equivalent to panel c) (or even better, as it was done in Supplemental Fig 7c)). Also, showing stereo pictures can help.

Response: We thank the reviewer for this valuable suggestion. We agree that the original Extended Data Fig. 12b did not sufficiently illustrate the weakened density and increased flexibility of the cytoplasmic interaction region in the T460D mutant. In the revised manuscript, we completely redesigned this panel **(Fig. 6b, and is also presented below)** to more clearly visualize the structural differences between wild-type *At*AMT1;1 and the T460D phosphomimetic mutant.

Fig. 6a, b of the revised manuscript:

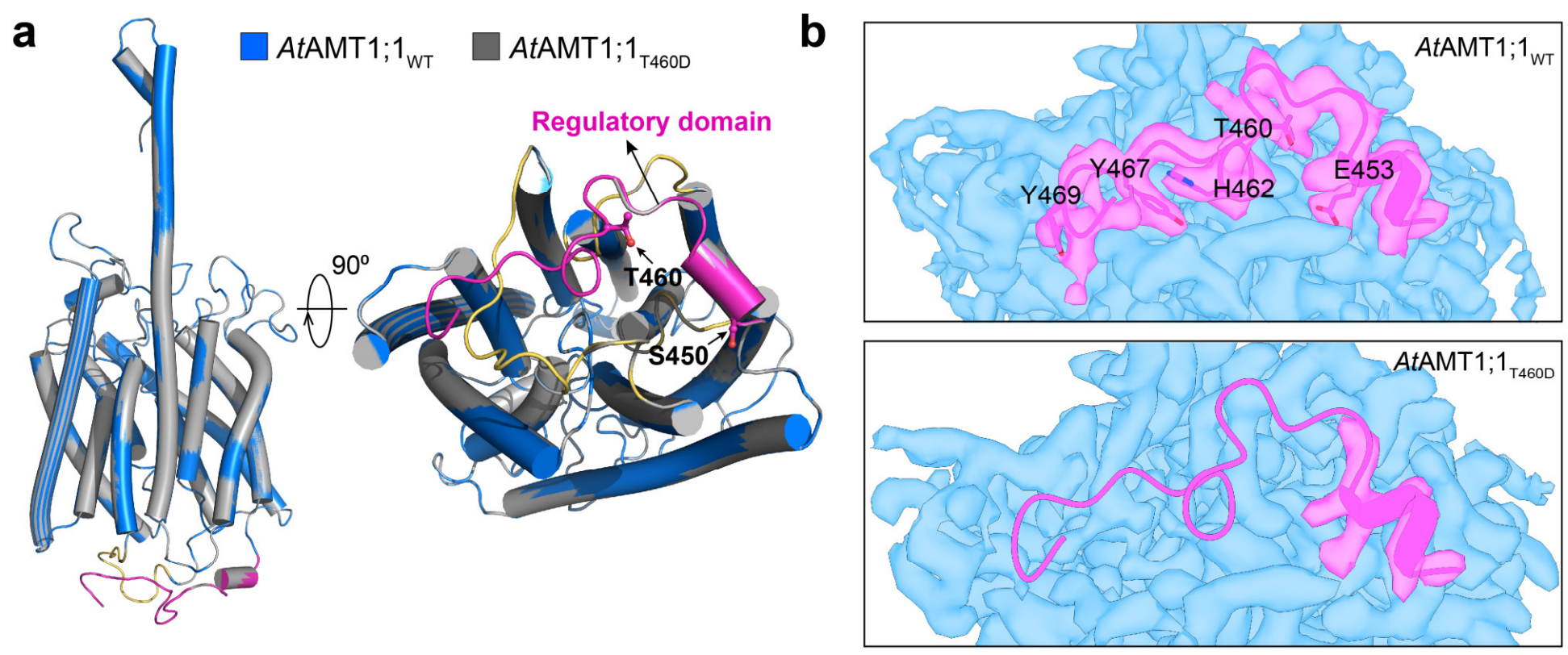

18) In Fig 6:

a) Panel a): the highlights for the regulatory domain are basically invisible. It is hardly possible to note any differences between both structures.

Response: We thank the reviewer for this helpful comment. We agree that the original version of Fig. 6a did not clearly visualize the regulatory domain or the structural differences between wild-type *At*AMT1;1 and the T460D mutant. In the revised figure, we redesigned the structural representation to improve clarity. Specifically, the intracellular loops L1, L3, and L5 of the wild-type structure are now highlighted in yellow, while the C-terminal regulatory domain (CRD) is shown in magenta. This revised color scheme allows the relative positions and interactions of these regulatory elements to be visualized more clearly. Importantly, the overall structures of wild-type and T460D remain highly similar and do not exhibit obvious large-scale conformational rearrangements. Instead, the primary difference is that the CRD and adjacent L5 region in the T460D mutant display substantially weaker cryo-EM density and could not be modeled reliably, suggesting increased local flexibility or conformational heterogeneity in this region relative to the wild type. We believe the revised figure now better illustrates this key observation.

b) Panel b): legend describes the right side elements as the isolated C-term (ok) and intracellular loop region. However, the circle on the left protein figure is apparently misleading here, since that area is not shown on the right bottom figure. According to the text, the right bottom figure is the cytoplasmic face of the protein without the top (C-term module). What is the circle on that intracellular loop region on the right? The exit vestibule? - not described.

Response: We thank the reviewer for pointing this out and agree that the original presentation and figure legend were unclear and potentially misleading. In the revised version, we removed the circle annotation from the right-side panel to avoid confusion **(now Fig. 6c)**. We also substantially revised the figure legend to more clearly describe each structural view and the specific regions being displayed.

c) Panel d): identify what the sequences in the boxes are showing - segments of the different intracellular loops that interact with the C-term region?

Response: We thank the reviewer for this helpful comment. We agree that the original Fig. 6c did not sufficiently clarify how the intracellular loops interact with the C-terminal regulatory domain (CRD). In the revised version, we substantially redesigned this panel to provide a clearer presentation of the intracellular interaction network. We now first show an overall view of the interactions between the CRD and the intracellular L1, L3, and L5 loops **(Fig. 6c)**. Because these loop regions are spatially clustered and difficult to distinguish in a single view, we additionally separated the individual loop regions into enlarged panels to more clearly display the detailed electrostatic and hydrogen-bonding interactions formed with the CRD **(Fig. 6e, f)**. We believe the revised figure now provides a much clearer structural interpretation of the regulatory interaction network.

d) P15 l 293: Should D264 be D364 instead (considering the sequence and Fig 6b)? Why is this position highlighted? What is its relevance? It is only mentioned here, as a conserved residue located at the pore exit (see point 6).

Response: We thank the reviewer for carefully noticing this issue. The residue was indeed incorrectly labeled as D264 in the previous version, whereas the correct residue is D364. This has now been corrected throughout the revised manuscript and figures.

D364 was highlighted because it is located within the intracellular exit region of the conduction pathway and represents one of the few conserved charged residues positioned near the pore exit. Similar acidic residues have also been noted in previous structural studies of bacterial AmtB and fungal Mep2, although their functional roles have not been experimentally investigated in detail. In our study, we systematically mutated several negatively charged and polar residues located in the exit vestibule

region **(Fig. 4e)**. Interestingly, among all tested residues, only mutation of D364 markedly impaired transport activity, whereas mutations of neighboring residues showed little or no effect **(Fig. 4f)**. At present, our structural data do not allow us to assign a precise mechanistic role to D364. However, based on its conserved position and functional importance, we speculate that D364 may play a particularly important role in maintaining the local electrostatic environment or structural organization of the intracellular exit pathway. We have now discussed these possibilities more cautiously in the revised manuscript without overinterpreting the current data **(Lines 322-326)**.

19) P 15,16 l299-305: the plasma membrane is surrounded by ions. What is the local pH there? Existing literature describes AMT1;1 as electrogenic (ref 17). As mentioned above (see 4c)), it appears unlikely that H^+ are consumed on the lumen side of the membrane as consequence of AMT transport. The authors could make better conclusions, sustained by their observations on these loop residues and C-terminus interactions or delta C-terminus - again conserved residues and again the same observations across AMTs.

Response: We thank the reviewer for this important comment and agree that our previous discussion regarding possible proton donation by positively charged intracellular residues was overly speculative and not sufficiently supported by existing electrophysiological evidence (Loqué et al, 2009, J Biol Chem).

Accordingly, in the revised manuscript we removed the previous speculation regarding proton transfer mediated by the intracellular positively charged residues. Instead, we now focus on the structural and regulatory roles of the conserved intracellular loop network and C-terminal interactions. Similar regulatory architectures and functional dependencies on the C-terminal region have also been reported in bacterial AmtB and fungal Mep2 homologs **(As now shown in Extended Data Fig. 15 and is also presented above)**. We therefore interpret these conserved intracellular loops primarily as regulatory elements that modulate transporter dynamics and activity through structural coupling with the C-terminal domain, rather than as direct participants in proton transfer during substrate translocation.

20) P16, 17 last paragraph:

a) make proper citations to previous work(s) showing similar or different results from what is observed here. If (as it seems) conclusions here are backed up by other works, cite them.

Response: We thank the reviewer for this important suggestion. Following the reviewer's comments and our further analysis of the structural and functional data, we realized that several mechanistic conclusions in the previous version were overly speculative, particularly regarding the precise substrate translocation mechanism and the chemical identity of the transported species during conduction. Accordingly, we substantially revised the final mechanistic model and redrew **Fig. 7** to focus primarily on the regulatory mechanisms supported directly by our experimental observations. In the revised manuscript, we also incorporated additional citations to previous studies.

Fig. 7 of the revised manuscript:

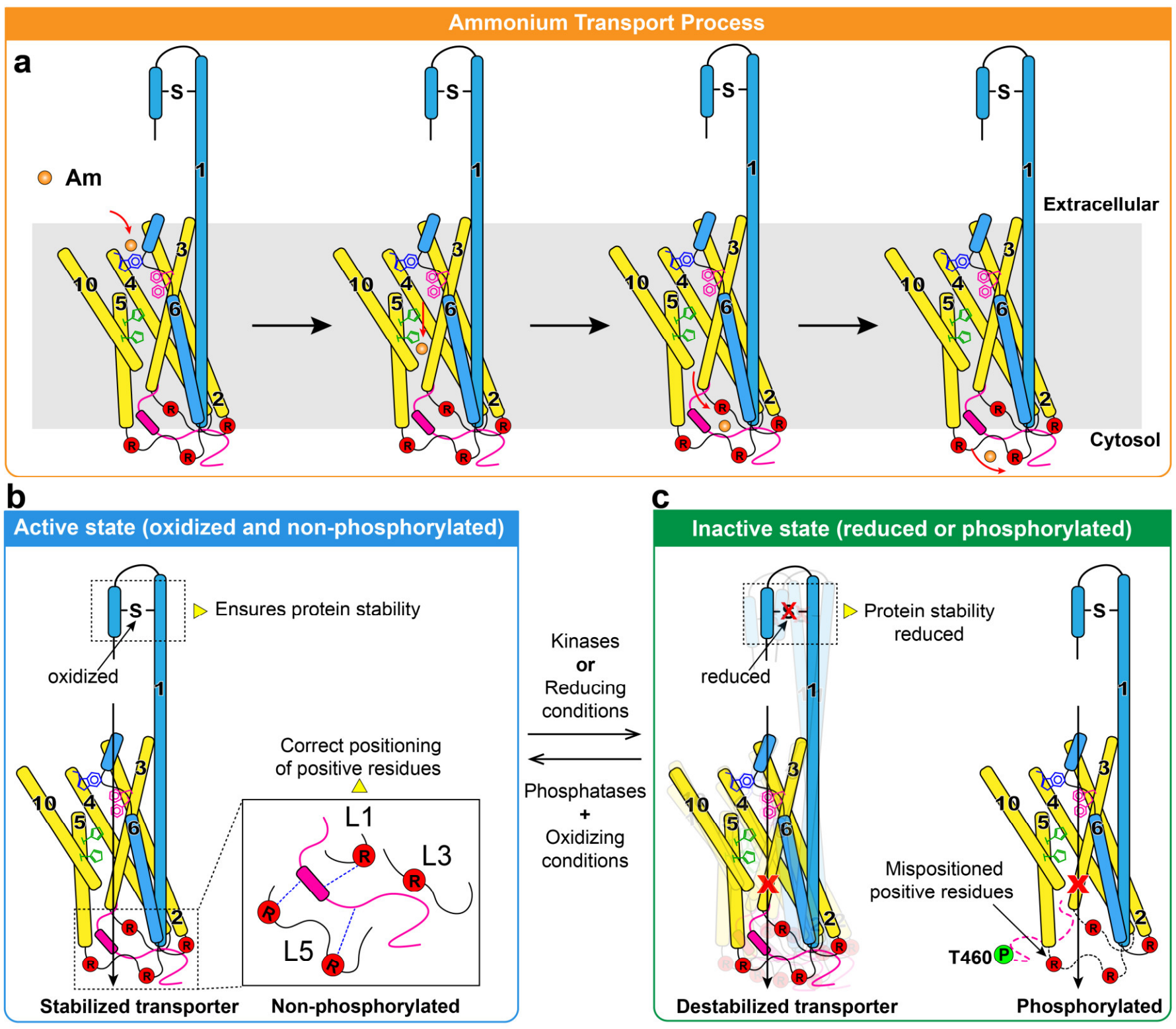

b) Nothing in this work indicates that NH_4^+ is deprotonated at the F/F site.

Response: We thank the reviewer for pointing this out. We did not intend to propose that NH_4^+ is deprotonated at the F/F constriction site, either in the original or revised manuscript. We apologize that the previous mechanistic illustration may have unintentionally created this impression. To avoid ambiguity, we have completely

redesigned the mechanistic model in the **revised Fig. 7**. In particular, the substrate along the proposed transport pathway is now uniformly labeled as "Am" without distinguishing between NH_4^+ and NH_3.

c) NH_4^+ or Am will never bind to a transporter, rather interact

Response: We thank the reviewer for this important comment. We agree that the terms "bind" or "binding" may imply formation of a stable, well-defined substrate-bound state, which is difficult to conclude from the current structural data. Accordingly, in the revised manuscript we replaced such wording with more neutral descriptions, including "interact", "density adjacent to", or "substrate-associated density", depending on the context.

d) What data allows predicting that D264-again likely D364 is involved in NH_3 binding (how?) and translocation (how)?

Response: We thank the reviewer for this important comment and agree that our previous wording regarding D364 was overly speculative. Our current data do not provide direct evidence that D364 participates in NH_3 binding.

In the revised manuscript, we therefore removed statements suggesting that D364 directly binds NH_3 or functions as a specific substrate coordination site. Instead, we now describe D364 more cautiously as a functionally important residue located within the intracellular exit vestibule. Our conclusion is based primarily on mutational analysis showing that both D364A and D364N markedly impair transporter activity, whereas mutations of neighboring polar or charged residues have comparatively minor effects. Structurally, D364 is positioned near the intracellular exit pathway, suggesting that it may contribute indirectly to transport by maintaining the local electrostatic environment, hydration network, or structural organization of the exit vestibule rather than directly interacting with a specific transported species. Accordingly, we revised the text to avoid mechanistic overinterpretation regarding the role of D364 in substrate translocation.

e) This reviewer has serious problems with this mechanistic proposal and the scheme shown in Fig 7. Such problems arise from considerations on data published on other Amt proteins (and the high degree of conservation observed between them and AMT1;1,

especially in the pore region), published functional data on this AMT1;1 protein and the over-interpretation of results by the authors.

Response: We thank the reviewer for this important and thoughtful comment. Following the reviewer's suggestions and our further re-analysis of both the structural and functional data, we recognized that the previous mechanistic model in Fig. 7 contained interpretations that were not sufficiently supported by direct experimental evidence **(The new Fig. 7 is presented above)**. The updated model now focuses primarily on the experimentally supported regulatory mechanisms of *At*AMT1;1, including the extracellular N-terminal disulfide bond, the intracellular C-terminal regulatory domain, and the conserved positively charged intracellular loop network.

Minor issues:

p.10, l193, p11 l 199, l 203, l 204 (and others): please change channel for pore.

Response: We thank the reviewer for this helpful suggestion. We agree that the term "channel" may imply a passive diffusion mechanism and is not sufficiently precise in the context of the current study. Accordingly, in the revised manuscript we replaced "channel" with "pore" throughout the text when describing the internal conduction pathway of *At*AMT1;1.

P11, l 203: histidine is not a hydrophobic residue

Response: We thank the reviewer for pointing this out. We agree that histidine should not be classified as a hydrophobic residue. In the revised manuscript, we corrected the description and replaced "hydrophobic residue" with "aromatic residue" in the corresponding section.

P11 l 206 add a reference to the given hydrated radius of NH_4^+

Response: We thank the reviewer for this helpful suggestion. We realized that the previous description regarding the hydrated radius of ammonium was not sufficiently precise. In the revised manuscript, we clarified that ammonium does not possess a fixed hydrated radius but instead forms a flexible hydration shell in aqueous solution. We also added an appropriate reference to support this statement **(Lines 270-273)**.

P15 l289-290: Why not simply name loops and they originate after TM? TM1-TM2 forms L1, and so on, such that the loop shown in Suppl Fig 12c) is L5, described in ref

20 as loop 5 with important connections to the C-terminus of own and neighbor monomers.

Response: We thank the reviewer for this helpful suggestion. In the revised manuscript, we adopted a consistent loop nomenclature based on the transmembrane helices, such that the loop connecting TM1–TM2 is designated L1, TM2–TM3 as L2, and so forth **(Lines 341-342)**. We also added this definition explicitly in the main text to facilitate structural interpretation throughout the manuscript. Accordingly, the intracellular loop highlighted in the previous Supplementary Fig. 12c is now consistently referred to as L5, in agreement with the nomenclature used in previous studies.

P34 l554: replace key by targeted

Response: We thank the reviewer for pointing this out. The term "key" has been replaced with "targeted" in the revised manuscript.

P16 l307: only in plants?

Response: We thank the reviewer for this important comment. We agree that the original wording could be misleading. In the revised manuscript, we clarified that the specific positively charged residues identified in *At*AMT1;1 are highly conserved among plant AMT1 transporters **(Fig. 6h)**. However, enrichment of positively charged intracellular loop regions, particularly within L1, L3, and L5, is a more broadly conserved structural feature observed across bacterial, fungal, and plant members of the AMT/MEP/Rh superfamily **(As now shown in Extended Data Fig. 15, and is also presented above)**. We therefore revised the text to distinguish between conservation of the overall positively charged intracellular loop architecture across kingdoms and conservation of the individual residues specifically within plant AMT1 proteins.

P19 l358: replace deprotonated ammonia by ammonium cation

Response: We thank the reviewer for pointing this out. We agree that "deprotonated ammonia" was inaccurate and potentially confusing in this context. In the revised manuscript, we replaced it with "ammonium cation" accordingly.

Along the text: AMT1 and AMT2 are two clades of plant AMT types and these belong to the AMT/MEP/Rh family of ammonium transporters

Response: We thank the reviewer for this helpful suggestion. In the revised Introduction,

we explicitly clarified that AMT1 and AMT2 represent the two major clades of plant AMT transporters and that both belong to the evolutionarily conserved AMT/MEP/Rh superfamily of ammonium transporters **(Lines 38-39)**.

There seems to be a mistake when refering to a density map with NH_4^+ at pH 7.4 and pH 6.0. In p 12, lines 235 and l 236 both suggest supplemental figure 8b (which only shows helix 10 at low pH).

Response: We thank the reviewer for carefully noticing this issue. The figure citations related to the density maps at pH 7.4 and low pH conditions have now been carefully corrected throughout the revised version.

Discussion should not repeat sentences from the introduction.

Response: We thank the reviewer for this suggestion. We carefully re-examined the Discussion section and revised the text to avoid repetition of statements already presented in the Introduction. In the revised manuscript, the Discussion has been substantially rewritten to focus more specifically on interpretation of the structural and functional findings, comparison with previous studies, and the broader regulatory implications of our results.

Supplemental Fig13: Which structure of Ecoli was used to observe and compare C-termini? All structures of AmtB have a disordered C-terminus. The only observation of this region in the Ecoli AmtB was in the complex structure with its regulator protein. Alternatively, the structure of Archaeoglobus fulgidus Amt1 alone comprised the entire protein up to the C-terminus.

Response: We thank the reviewer for this important comment. In our revised analysis, the bacterial comparison was primarily based on the E. coli AmtB mutant structure reported by Khademi et al. (Science, 2004), because this structure contains the most well-resolved intracellular L5 region among the available apo-like AmtB structures, thereby allowing more informative comparison of intracellular loop organization. We agree that the C-terminal region of isolated AmtB structures is generally poorly resolved and that clearer stabilization of the C-terminus is mainly observed in the AmtB–GlnK complex structures. Accordingly, in the revised manuscript we further compared the conformational differences between apo AmtB and the AmtB–GlnK

complex. This comparison revealed noticeable rearrangements of the intracellular loop regions, particularly L5, upon GlnK association, together with increased stabilization of the cytoplasmic region **(As now shown in Extended Data Fig. 15a, b and is also presented above)**. We have now clarified these distinctions and limitations more carefully in the revised manuscript and revised the discussion accordingly **(Lines 432-446)**.

P20 l391: Which intracellular loop of MEP2?

Response: We thank the reviewer for pointing this out. We apologize that the intracellular loop identity was not sufficiently clear in the previous version. In the revised manuscript and figures, the loop nomenclature has now been explicitly defined, and the relevant intracellular loop in fungal Mep2 is clearly identified as the L5 loop.

In the revised version, we now more clearly illustrate the structural differences between the wild-type and phosphomimetic Mep2 structures, highlighting the relative positioning of the L5 loop and the C-terminal region **(As now shown in Extended Data Fig. 15c, d and is also presented above)**.

W255 is not correctly modelled in the structure PDB: 22NK (WT, with NH_4^+, pH 7.4)

Response: We thank the reviewer for carefully noticing this issue. We re-examined the side-chain fitting and local cryo-EM density around W255 in the PDB 22NK model (WT, with NH_4^+, pH 7.4) and identified an inconsistency in the previous modelling. The residue has now been carefully rebuilt and refined against the corresponding density map in the revised structure.

Reviewer #2:

The manuscript by He et al., reports the structure of AMT1.1 in apo and neutral pH and bound to ammonium under neutral and acidic conditions. In addition, the study reports the structure of the phosphomimetic mutant T460D, revealing the mechanism of inactivation. AMT1:1 play a crucial role in the uptake of environmental ammonium in green leafy plants. Ammonium transport in plants is crucial for acquiring, distributing, and assimilating nitrogen, a vital nutrient for growth, development, and high crop yields. As such, this study reports the structure of an important transporter that will be of interest to a wide range of researchers in structural biology, plant sciences and agricultural research. However, the study is currently too preliminary for me to recommend publication at this stage. I have detailed my thoughts below. In summary, the results presented are an excellent start and certainly hint at the mechanisms drawn from the data. However, many of the experiments are insufficiently detailed to draw the types of mechanistic conclusions being made. While the yeast complementation assays are useful as a general screening tool for activity, they fail to provide the resolution needed to ascribe functions to single amino acid side chains. The inconsistency between the complementation assay and the isotope uptake assay is concerning and may hint at a deeper disconnect between these two assays. The structures, while sufficient to determine the overall structure, are too low resolution to unambiguously identify the ligand or understand the allosteric mechanism behind the T460D mutant. The authors also appear not to have considered the role of the disulphide in oxidative regulation of the system.

My recommendation is to undertake more detailed studies, consider molecular dynamics, and improve the resolution to determine the structure in C1 at high resolution.

Response: We thank the reviewer for the constructive comments and positive evaluation of our work. We agree that the previous version of the manuscript contained several mechanistic interpretations that were not sufficiently supported by the available structural and functional data. In the revised manuscript, we therefore substantially expanded the experimental analyses, improved the structural datasets, and revised the mechanistic discussion to provide a more cautious and data-supported interpretation.

Specifically, we performed additional single-molecule mass photometry analyses, including measurements in different detergents and under different redox conditions, to better characterize the oligomeric stability of *At*AMT1;1. We further carried out thermal stability assays with quantitative Tm fitting, additional yeast complementation assays for multiple pore-lining and regulatory mutants, and more detailed concentration-gradient growth analyses for mutants displaying subtle phenotypes. To further investigate transporter dynamics and phosphorylation-dependent regulation, we also performed molecular dynamics simulations comparing wild-type and T460D proteins under different conditions.

In parallel, we collected additional cryo-EM datasets for both wild-type *At*AMT1;1 and the phosphomimetic T460D mutant, which improved the overall resolution and particularly enhanced the quality of the C1 reconstructions. These improvements allowed clearer visualization of the intracellular regulatory regions and local substrate-associated densities.

Importantly, following the reviewer's concerns, we substantially revised the mechanistic interpretation throughout the manuscript. In the revised version, we avoid overinterpretation regarding the precise NH_3/NH_4^+ transport mechanism and instead focus more conservatively on the experimentally supported transport pathway features and regulatory mechanisms. We also expanded the discussion of oxidative regulation mediated by the conserved N-terminal disulfide bond and incorporated additional experimental evidence supporting its role in transporter stability and redox responsiveness.

Overall, we believe that the revised manuscript now provides a more rigorous and balanced analysis of the structural basis and regulatory mechanisms of this important plant ammonium transporter.

Detailed comments:

Lines 64-65: “add-ammonium state” should be re written.

Response: We thank the reviewer for this suggestion. In the revised manuscript, we carefully replaced the wording "add-ammonium state" throughout both the main text and figures. In figure labels, this term has been replaced with "with NH_4^+", while in the

main text it has been revised to "structure determined in the presence of ammonium" to provide clearer and more accurate descriptions **(Lines 81-82, 226-228 and so on)**.

Lines 98-101: The authors state that the assays shown in Fig. 1 demonstrate that AMT1.1 is a high affinity transporter of ammonium. I would disagree with this statement, as the assays do not provide any quantitative analysis of affinity. The yeast complementation assay in Fig. 1a demonstrates that under low ammonium conditions (50 uM), the overexpression of AMT1.1 is sufficient to allow growth in the ammonium transporter deficient background. However, this does not provide a value for the Km. If the Km for A. thaliana AMT1.1 has been determined previously, the authors can quote this in the paper. However, if the Km has not been previously reported, then I suggest the authors calculate the Km so this can be compared to the bacterial homologues discussed in the introduction. The authors can use the isotope labelled $NH4^+$ substrate and quantification with an LC-MS/MS system and the HEK293 cells.

Response: We thank the reviewer for this important comment. We agree that the yeast complementation assay alone cannot be used to rigorously define *At*AMT1;1 as a high-affinity transporter, since it does not provide quantitative affinity measurements.

Accordingly, in the revised manuscript we moderated the interpretation of the yeast growth assay and additionally determined the apparent transport affinity of *At*AMT1;1 using our isotope uptake system in mammalian cells. Specifically, cellular ammonium accumulation rates were measured over a range of substrate concentrations, and the net uptake activity of *At*AMT1;1-expressing cells relative to empty-vector controls was fitted to derive a transport Km value of 49.53 ± 9.47 μM **(Fig. 1d and is also presented below)**. This finding indicates that *At*AMT1;1 functions as a high-affinity transporter, which is consistent with previous measurements observed in plants and yeast.

Importantly, the obtained Km value is also in good agreement with previously reported transport measurements for plant AMT1 transporters, which generally within the 10–100 μM range. We believe these additional quantitative analyses now provide stronger support for classifying *At*AMT1;1 as a high-affinity ammonium transporter **(Lines 109-114)**.

Fig. 1d of the revised manuscript:

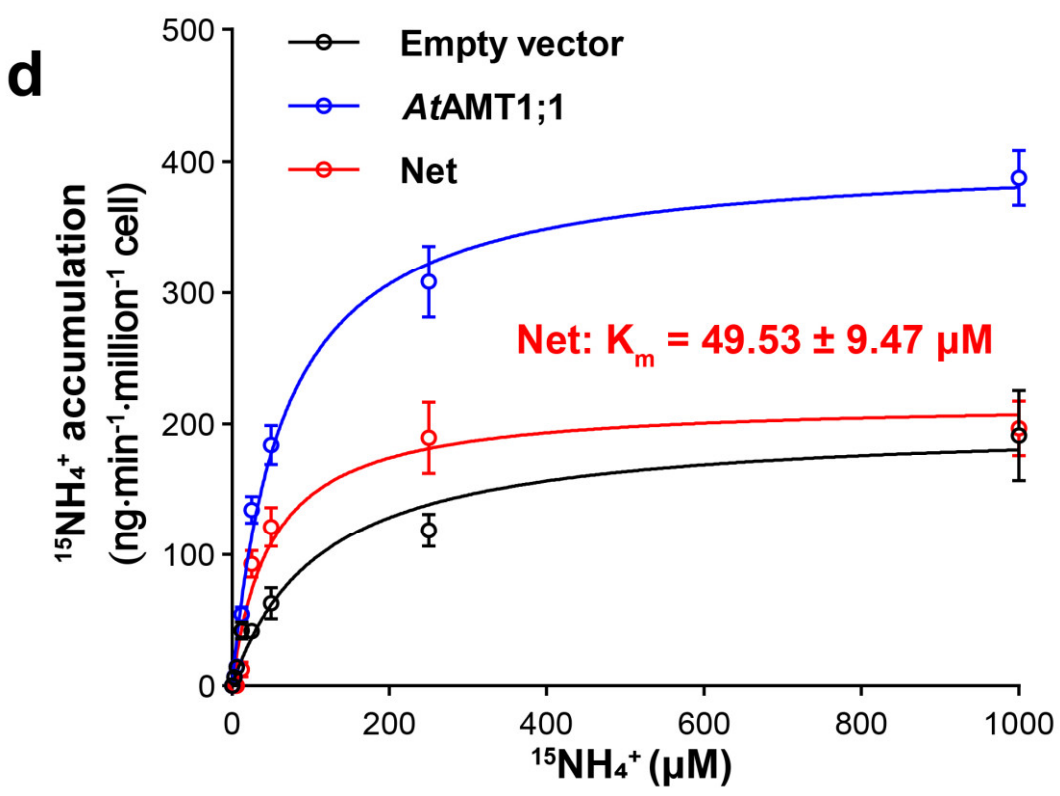

Extended Data Fig. 4: Is the upper band on the SDS-PAGE Hsp70? This should be mentioned in the figure legend or labelled in the gel.

Response: We thank the reviewer for pointing this out. The upper band in the SDS–PAGE indeed corresponds to Hsp70 contamination. In the revised manuscript, we have updated that figure by explicitly labeling the Hsp70 band in the gel image and clarifying this in the figure legend **(now Extended Data Fig. 1a and is also presented below)**.

Extended Data Fig. 1a of the revised manuscript:

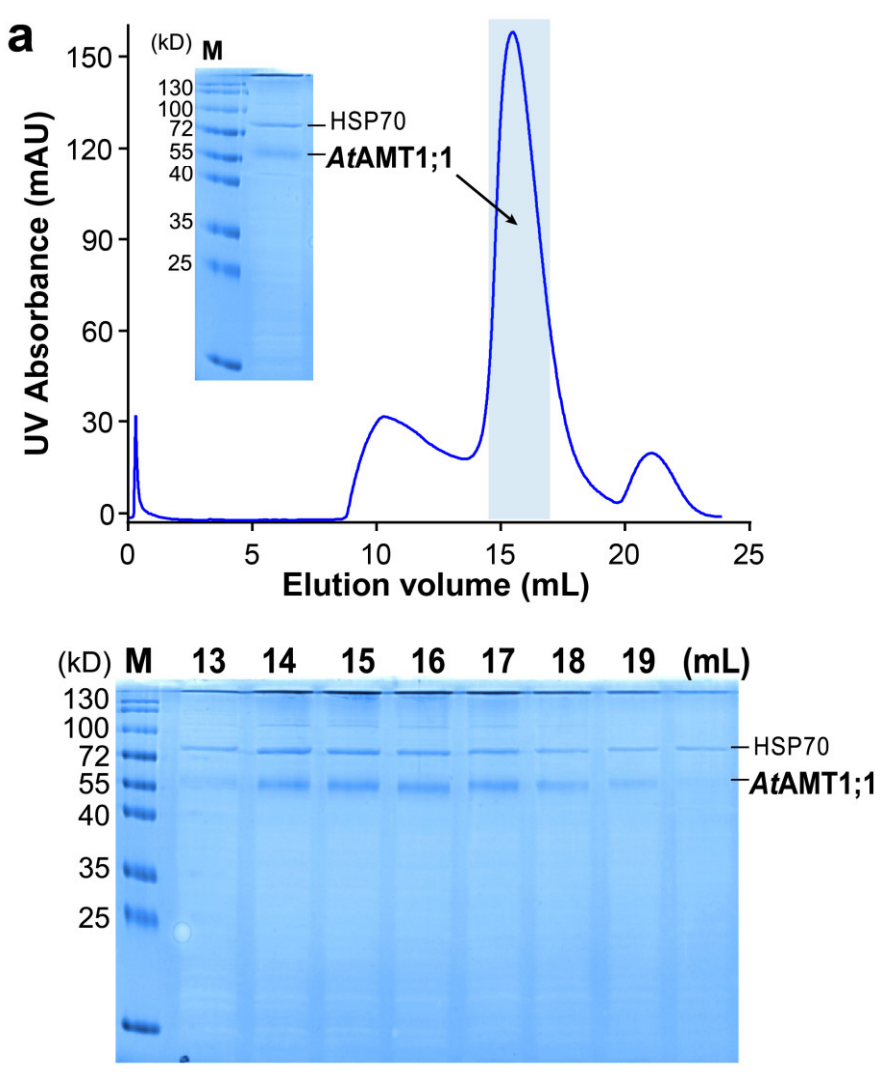

Extended Data Fig. 9b: The MST data look somewhat strange to me. Can the authors provide the raw data and detail how the Tm values are calculated? The shape of the unfolding curves for 1-32, 1-14 and C24S are different from the WT, indicating a substantial change in behaviour. I need more details about this assay and the authors interpretation to understand what this data tells me about these mutants. The current explanation is not satisfactory. The mass photometry data shown in Extended Data Fig. 9c suggests that the truncations, plus C24S, are monomeric. However, the unfolding

curves are different to the WT. Thus, I conclude the assay shown in Extended Data Fig. 9b may simply indicate the trimer breaking apart, rather than unfolding? It is hard to say.

Response: We thank the reviewer for this careful and insightful analysis. We apologize that the previous description of the CPM thermal stability assay and mass photometry data was overly simplified and may have led to confusion regarding the interpretation of the results.

In the revised manuscript, we substantially expanded both the experimental description and the analysis of these datasets. First, we added a detailed description of the CPM thermal unfolding assay and Tm fitting procedure in the Methods section, including the dye preparation, data acquisition, derivative analysis, and fitting strategy used to determine apparent Tm values **(Lines 810-826)**. We also now provide quantitative Tm fitting results for the WT and mutant proteins **(The fitting curve is presented blow)**.

Determination of melting temperatures (Tm) of *At*AMT1;1 variants:

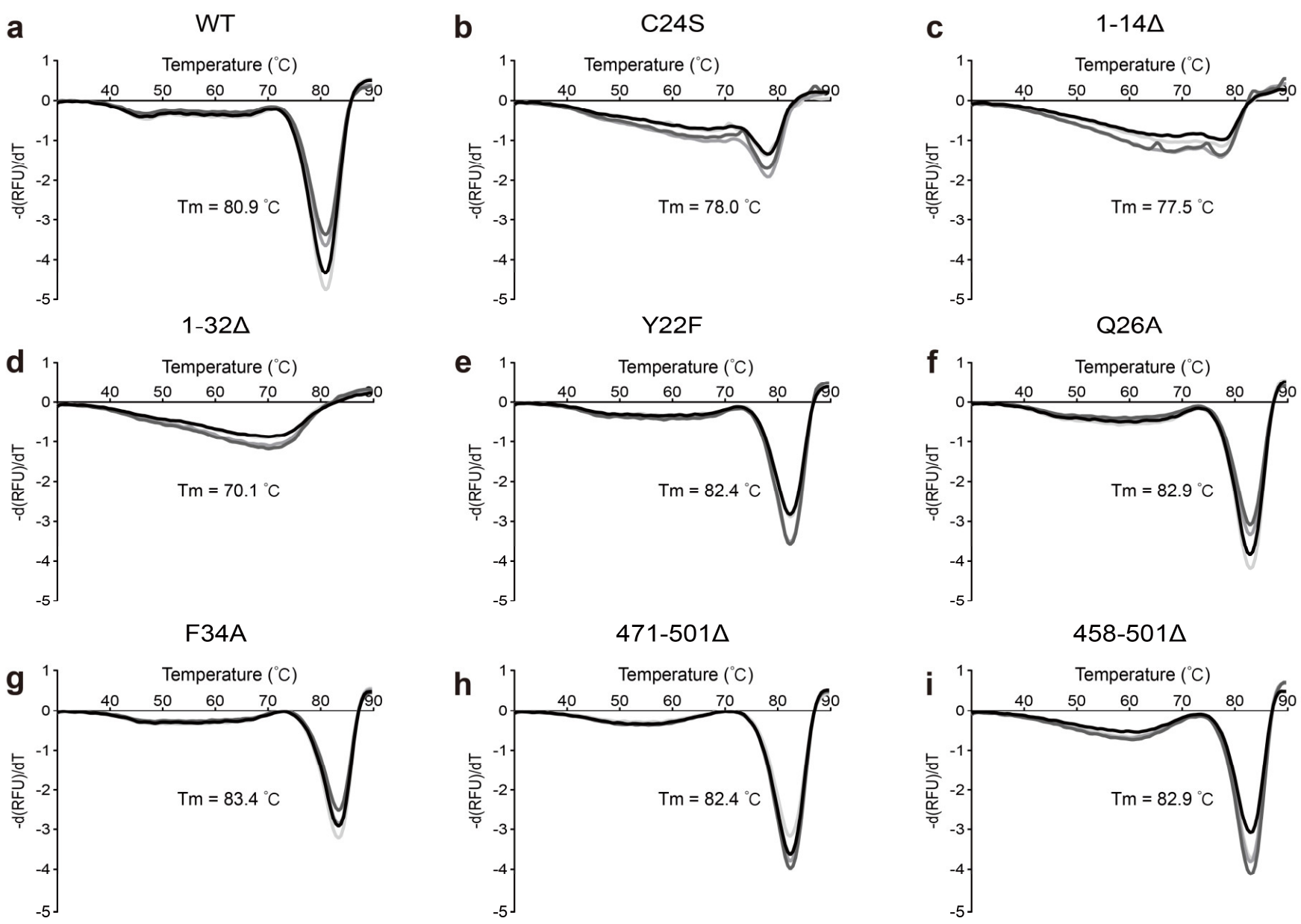

The revised analysis showed that the N-terminal truncation variants (1–14Δ and 1–32Δ) and the C24S mutant display reduced Tm values compared with WT *At*AMT1;1, supporting reduced structural stability **(Fig3c and is presented below)**. Importantly, we agree with the reviewer that the altered unfolding curve shapes suggest that these variants undergo substantially different behaviours relative to WT, consistent with

destabilization of the oligomeric assembly and/or altered unfolding pathways.

Fig. 3c of the revised manuscript:

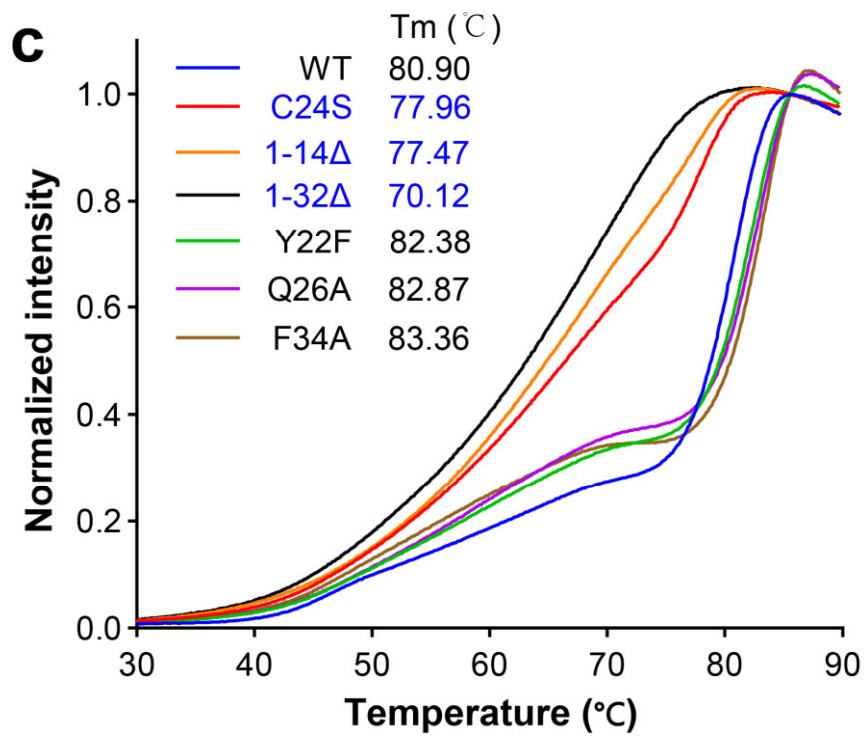

In parallel, we carefully re-examined the mass photometry data and realized that our previous interpretation was incorrect. By including detergent-only controls and comparing proteins purified in different detergents (DDM and LMNG), we found that the earlier low-mass peaks primarily correspond to detergent micelle signals rather than monomeric protein species. The later peaks represent the major stable oligomeric protein population. In the revised manuscript, we therefore completely reinterpreted the MP data accordingly. Importantly, unlike WT *At*AMT1;1, the C24S mutant and N-terminal truncation variants failed to display a clear stable oligomeric protein peak and instead showed highly dispersed mass distributions, indicating reduced oligomeric stability **(as now Extended Date Fig. 7b, c and is also presented below)**.

In the revised manuscript, we have substantially moderated our interpretation and now discuss the CPM data more cautiously as an indicator of altered oligomeric and conformational stability rather than simply protein unfolding. We believe that the revised analyses, together with the expanded methodological details and corrected MP interpretation, now provide a much clearer and more rigorous explanation of the behaviour of these mutants.

Extended Date Fig. 7b, c of the revised manuscript:

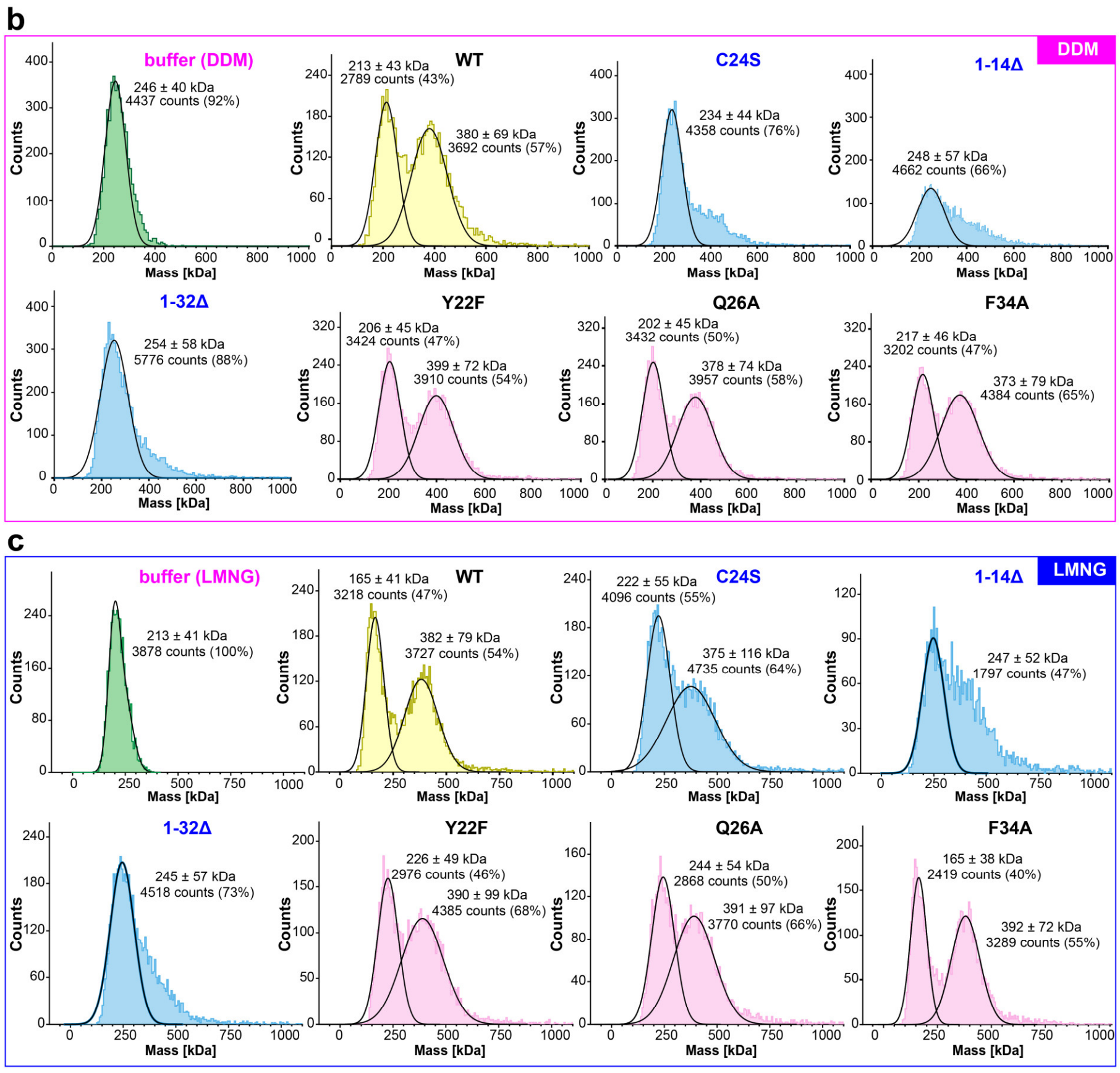

Extended Data Fig. 9c: I am also cautious about the data presented from the mass photometer. In our hands samples in detergent generate inherently noisy traces due to excess micelles. How was this data generated, through rapid dilution from LMNG?

Response: We thank the reviewer for this important comment. We agree that detergent micelles can generate substantial background signals and increase noise in mass photometry measurements of membrane proteins.

To address this issue, in the revised experiments we included detergent-only buffer controls under identical conditions. These measurements indeed revealed clear signal peaks originating from detergent micelles alone, confirming that detergent contributes substantially to the background scattering signal. Accordingly, we carefully reinterpreted the mass photometry data in the revised manuscript **(Results: Lines 166-185)**.

For all measurements, protein samples were diluted using buffer containing the identical detergent concentration as the corresponding control samples, thereby maintaining equivalent detergent micelle backgrounds between experimental and control conditions. Both LMNG and DDM conditions were tested independently.

Comparison of the detergent-only controls with the protein-containing samples allowed us to distinguish the detergent-associated peaks from the larger stable protein-associated peaks more reliably **(Method： Lines 890-907)**.

These additional controls and analyses are now described in the revised manuscript, and the interpretation of the MP data has been substantially revised accordingly.

My understanding from this data is that the truncations and C24S potentially result in monomeric protein, which appears to be non-functional. The truncations are expressed at a lower level. The authors need to provide expression data as an additional panel in Extended Data Fig. 9.

<u>Response:</u> We thank the reviewer for this important comment. After carefully re-evaluating the mass photometry data, we realized that our previous interpretation suggesting monomer formation was incorrect. In the revised manuscript, we therefore substantially revised both the experimental analysis and the interpretation of these data. To address the reviewer's concern regarding protein expression and sample quality, we additionally included SDS–PAGE and size-exclusion chromatography analyses **(as now Extended Data Fig. 7a and is also presented below)**. These results indicate that the WT protein, truncation variants, and C24S mutant all display broadly comparable expression and purification behaviour, without obvious differences in overall protein abundance.

Extended Date Fig. 7a of the revised manuscript:

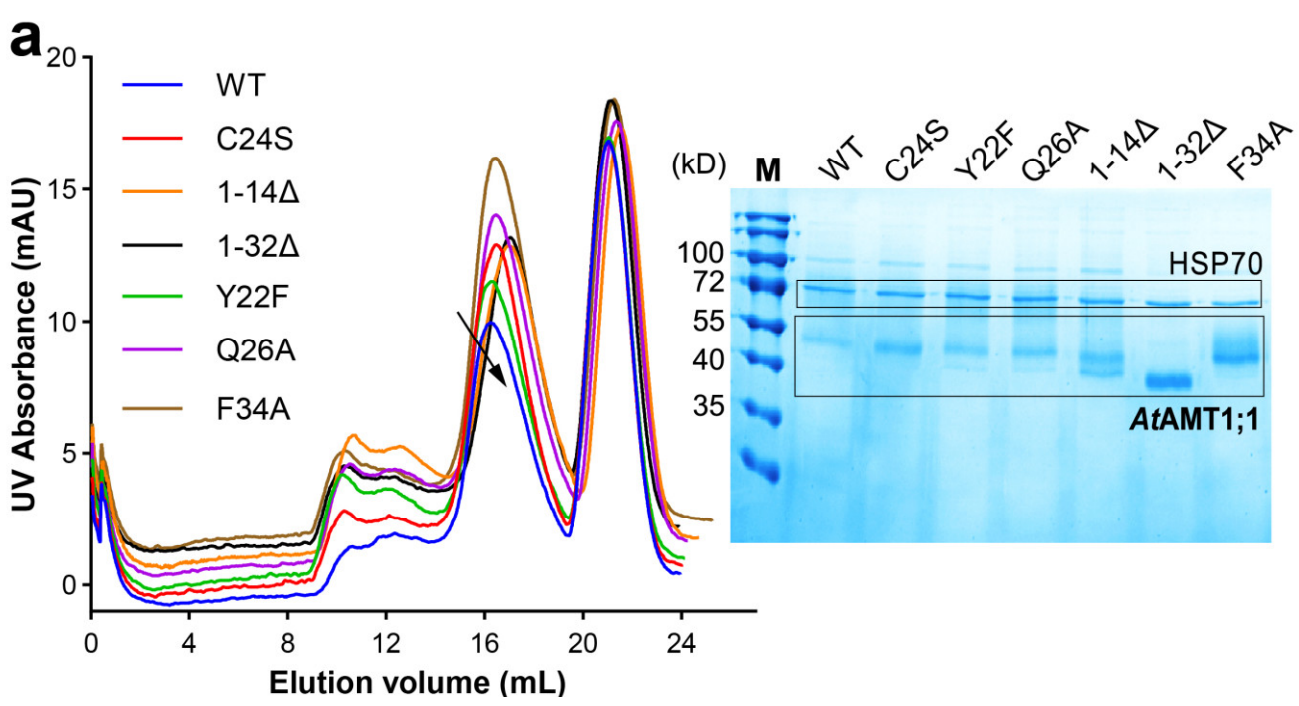

Thus, the impaired activity and altered MP behaviour are unlikely to arise simply from reduced expression levels. Instead, the combined SEC, thermal stability, MP, yeast complementation, and isotope uptake data collectively support the interpretation that disruption of the conserved N-terminal disulfide bond destabilizes the oligomeric

assembly and/or conformational integrity of *At*AMT1;1, thereby impairing transporter function.

It appears that the disulphide bond is critical for trimer assembly, yet this is not discussed.

Response: We thank the reviewer for this insightful comment. We agree that the potential role of the conserved disulfide bond in oligomer stability and regulation was insufficiently discussed in the original version.

In the revised manuscript, we substantially expanded both the experimental analyses and the discussion regarding the functional role of the N-terminal disulfide bond. Specifically, we now include additional yeast growth assays under different DTT concentrations, as well as single-molecule mass photometry experiments performed under reducing and re-oxidizing conditions. Our results showed that the reducing agent DTT inhibited yeast expressing wild-type *At*AMT1;1 more strongly than yeast expressing the C24S mutant **(Fig. 3e and is also see below)**. Furthermore, mass photometry analysis revealed that treatment with high concentrations of DTT caused the predominant oligomeric peak of wild-type *At*AMT1;1 to disappear, whereas subsequent addition of H_2O_2 largely restored the peak **(Extended Data Fig. 8 and is also see below)**. Importantly, the N-terminal truncation variant (1–32Δ) was insensitive to either DTT or H_2O_2 treatment. Together, these observations support the idea that the conserved extracellular disulfide bond contributes to oligomeric stability and may participate in redox-dependent regulation.

We attempted to further evaluate the effects of reducing and oxidizing conditions on transporter activity using yeast complementation assays and stable-isotope uptake experiments. However, during these experiments we found that the concentrations of H_2O_2 required to induce measurable oxidative effects caused severe cellular damage. Under these conditions, yeast growth was strongly inhibited and cells used for isotope uptake assays largely lost viability and transport capacity, making it difficult to reliably distinguish direct effects on AMT1;1 activity from nonspecific oxidative toxicity. We also attempted to directly compare the thermal stability of *At*AMT1;1 under oxidizing and reducing conditions. However, we found that the reducing reagents strongly

interfered with the fluorescence-based thermal stability assay, resulting in abnormal fluorescence signals and preventing reliable Tm determination. We therefore did not include these data in the revised manuscript.

We revised the manuscript to discuss the possibility that plant AMT1 proteins may function as redox-responsive transporters. We also now explicitly discuss the potential structural role of the disulfide bond in stabilizing inter-subunit coupling within the trimeric assembly **(Lines 212-215, 527-538)**. At the same time, we moderated our interpretation and clearly state that the precise molecular mechanism by which this extracellular intramolecular disulfide bond influences transporter stability and activity remains to be further investigated. We believe these additional experiments and expanded discussion now address the reviewer's concern and substantially strengthen the mechanistic interpretation of the disulfide bond function.

Fig. 3e of the revised manuscript:

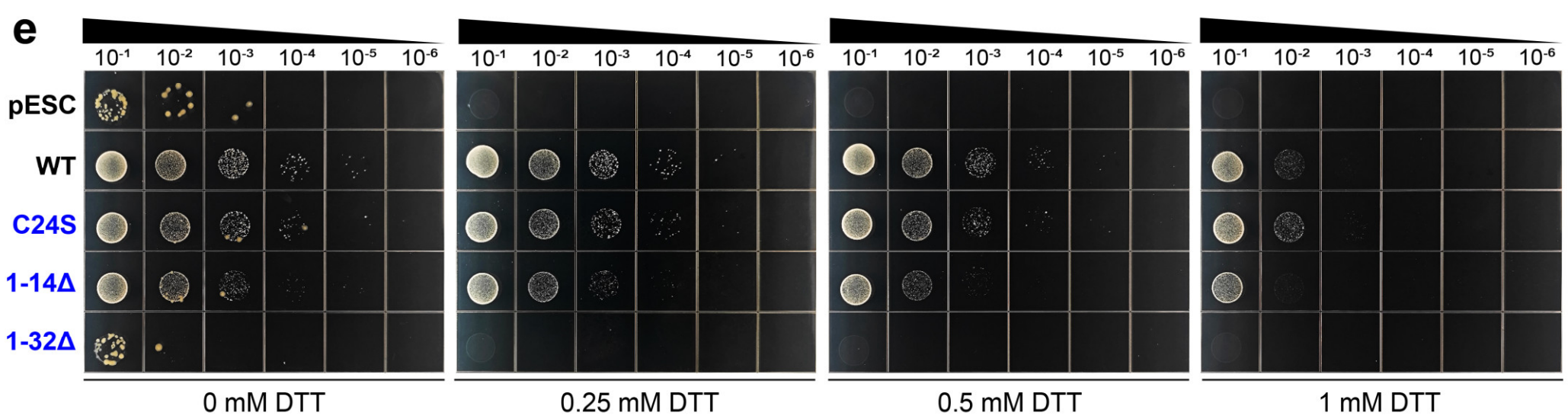

Extended Date Fig. 8 of the revised manuscript:

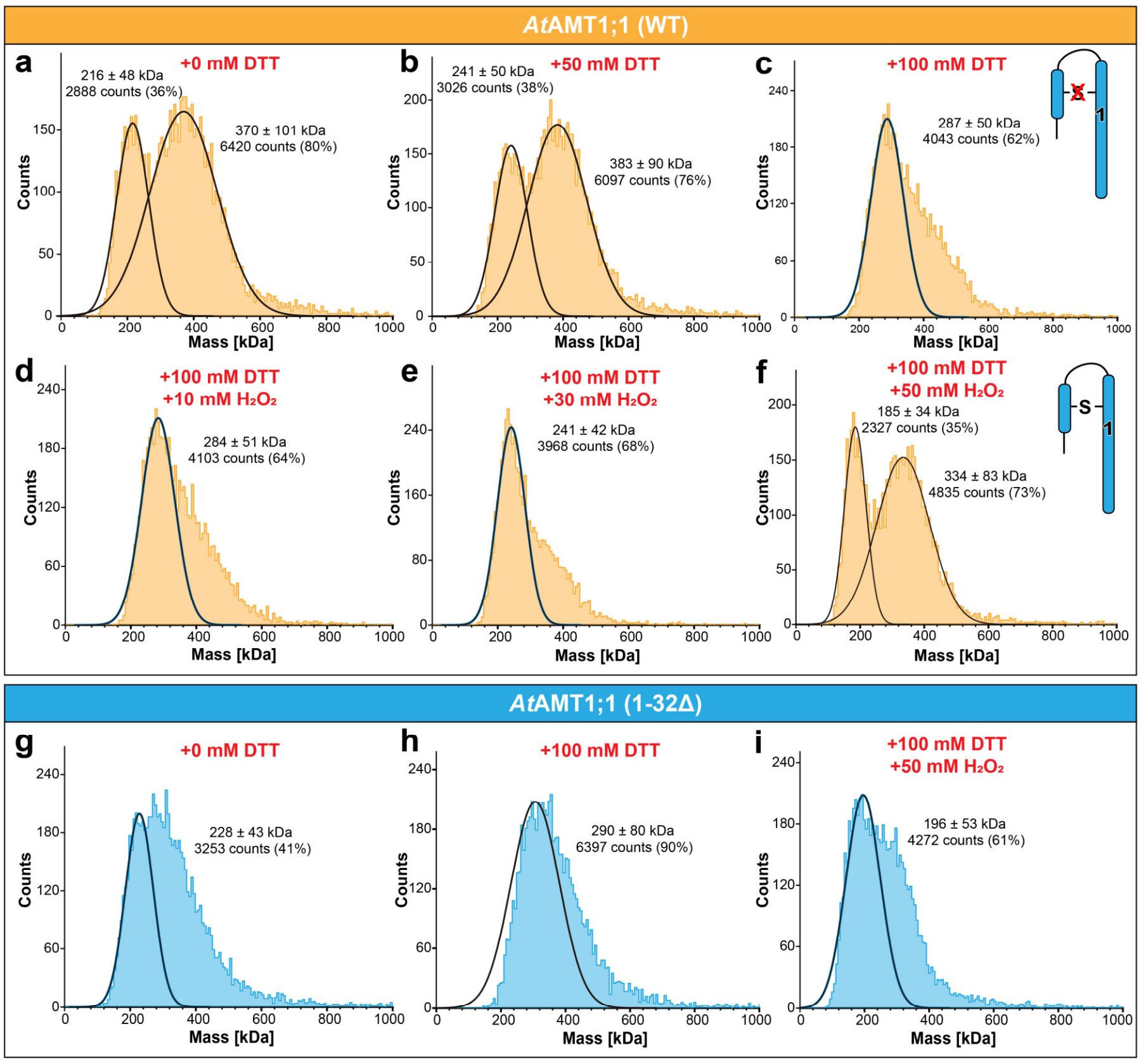

Also, Extended Data Fig. 9b is called before Extended Data Fig. 9a.

Response: We thank the reviewer for carefully noticing this issue. We re-checked the figure citation order throughout the revised manuscript and corrected the corresponding references.

The data shown is Extended Data Fig. 10 and Extended Data Fig. 11 appears to show the binding of ammonia/ammonium and meA to the transporter. I think it is almost impossible at this resolution to be certain this density corresponds to the ligand. I appreciate the density disappears in the H372F mutant, but this is still very speculative. In my opinion, the strongest evidence using the current data is to overlay the higher resolution structures of the bacterial Amt transporters and identify the density using the superposition. Given the high level of sequence conservation between the two transporters, it is highly likely the density corresponds to substrate. However, I don't think the cryo-EM data alone is sufficient to support this conclusion.

Response: We thank the reviewer for this careful and constructive comment. We agree that, at the current cryo-EM resolution, the densities alone are insufficient to unambiguously assign the bound species as NH_4^+, NH_3, or methylammonium. In the

revised manuscript, we therefore substantially moderated our interpretation and avoided overstatement regarding ligand identity.

To strengthen the analysis, we now present the densities at multiple contour levels and systematically compare them with previously reported bacterial AmtB and fungal Mep2 structures **(Extended Data Fig. 11, a-j, Extended Data Fig. 12, a-j, and is also see below)**. In particular, we emphasize that the observed densities occupy positions highly similar to those previously proposed as substrate-associated sites in bacterial AmtB structures. We also explicitly note that the methylammonium-associated density observed near the extracellular recruitment region resembles that reported in the bacterial AmtB mutant structure described by Khademi et al.

Importantly, we now avoid definitive assignment of these densities as substrate molecules. Instead, throughout the revised manuscript we state that these densities may correspond either to substrate-related molecules or ordered water molecules **(Lines 233-268)**. For the Twin-His-associated density, we further note that disappearance of the density in the H372F mutant suggests that H372 contributes to stabilization of the bound species, but does not by itself definitively establish substrate identity.

We fully agree with the reviewer that the strongest support for the substrate-associated interpretation comes from the structural superposition with previously reported higher-resolution bacterial Amt transporter structures and the strong conservation of the pore architecture. Accordingly, the revised manuscript now presents this comparison more carefully and frames our conclusions in a more conservative manner.

Extended Date Fig. 11 of the revised manuscript:

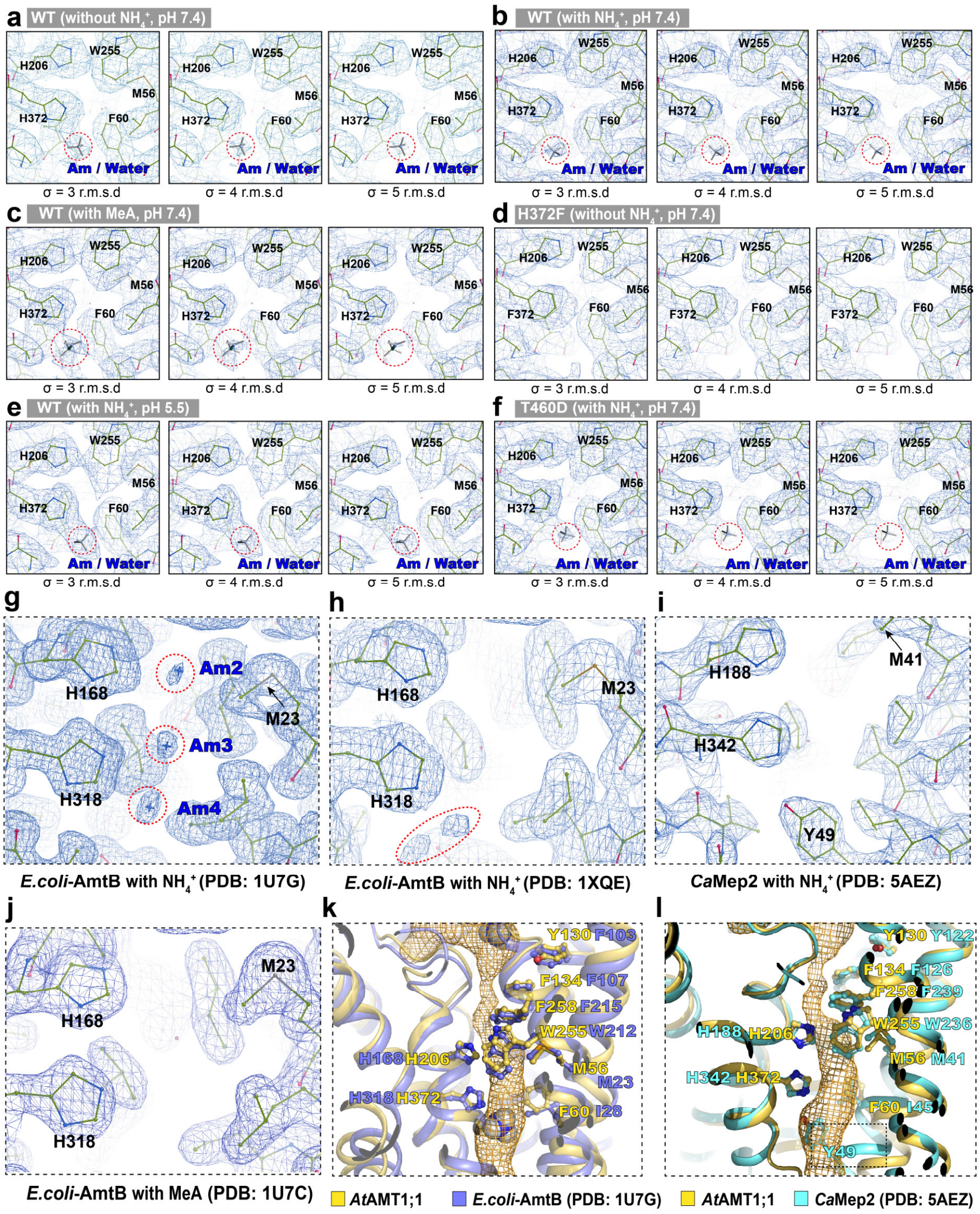
a WT (without NH_4^+, pH 7.4)
b WT (with NH_4^+, pH 7.4)
c WT (with MeA, pH 7.4)
d H372F (without NH_4^+, pH 7.4)
e WT (with NH_4^+, pH 5.5)
f T460D (with NH_4^+, pH 7.4)
H206
W255
M56
H372
F372
F60
Am / Water
σ = 3 r.m.s.d
σ = 4 r.m.s.d
σ = 5 r.m.s.d
g
h
i
H168
H318
M23
Am2
Am3
Am4
H188
H342
M41
Y49
E.coli-AmtB with NH_4^+ (PDB: 1U7G)
E.coli-AmtB with NH_4^+ (PDB: 1XQE)
*Ca*Mep2 with NH_4^+ (PDB: 5AEZ)
j
k
l
E.coli-AmtB with MeA (PDB: 1U7C)
Y130 F103
F134 F107
F258 F215
W255 W212
H168 H206
M56 M23
H318 H372
F60 I28
Y130 Y122
F134 F126
F258 F239
H188 H206
W255 W236
M56 M41
H342 H372
F60 I45
Y49
*At*AMT1;1
E.coli-AmtB (PDB: 1U7G)
*At*AMT1;1
*Ca*Mep2 (PDB: 5AEZ)

Extended Date Fig. 12 of the revised manuscript:

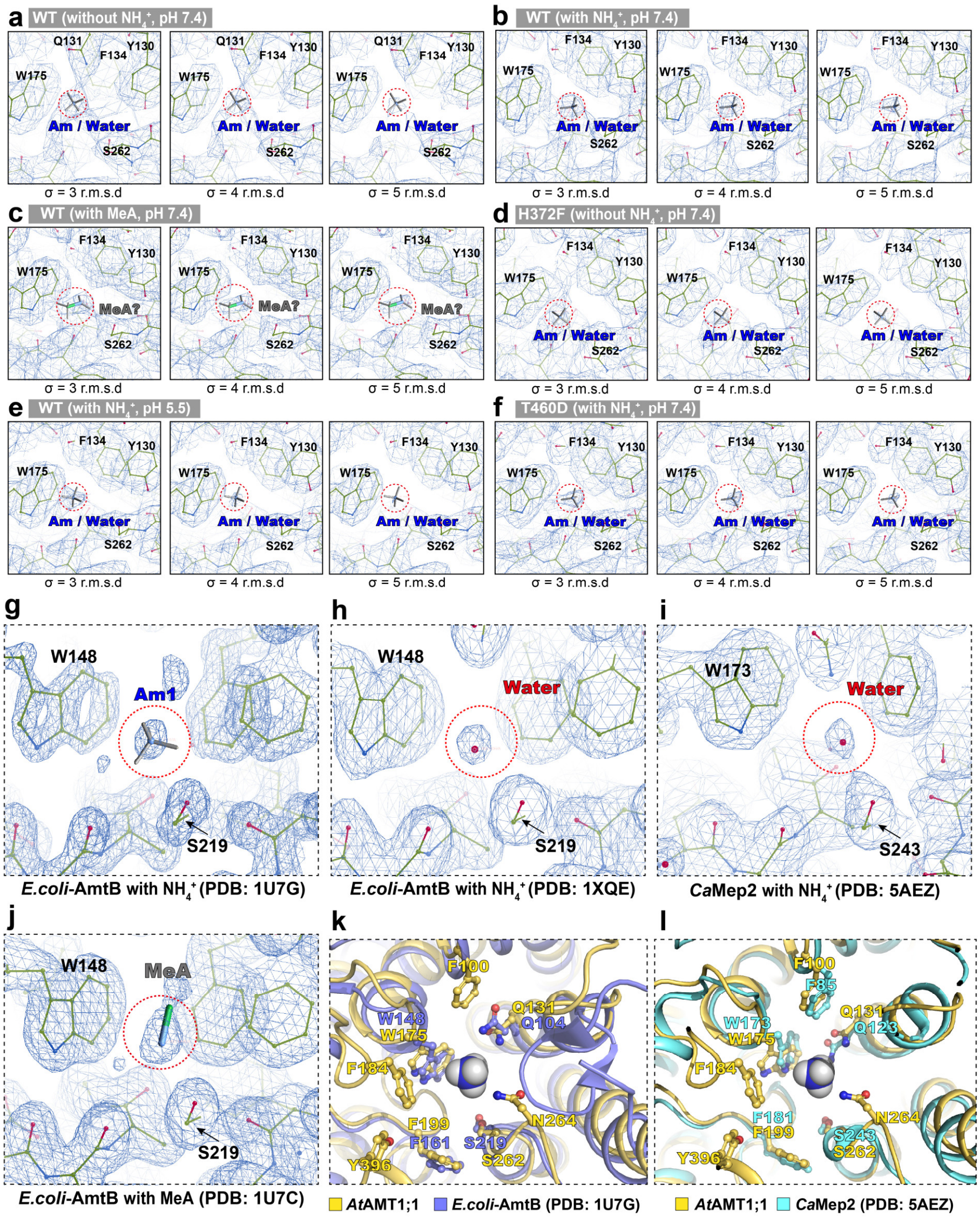

Line 214 & Figure 4d-e: The authors state the W175A mutant displayed the same growth on the yeast complementation assay yet displayed reduced transport in the isotope uptake assay. However, it looks to me as though more yeast colonies are in the 10^{-6} condition in panel d compared to the WT. A similar case is shown in F258A. However, neither W175A nor F258A show higher uptake (panel e). This is confusing. Can the authors explain this contradiction in the data?

Response: We sincerely apologize for this inconsistency and thank the reviewer for carefully noticing it. After re-examining the original experimental procedures and discussing the issue with the student who performed the assays, we identified an operational error during the yeast serial dilution experiment. Specifically, pipette tips

were not changed promptly during dilution steps in that particular experiment, which likely caused cross-contamination between samples and resulted in the misleading colony growth pattern.

To address this issue, we independently repeated the yeast complementation experiments multiple times. In the repeated experiments, the previously observed discrepancy was no longer detected. We have therefore replaced the original complementation data in the revised manuscript with the newly repeated experimental results **(as now Fig. 4f and is also see below)**. We apologize again for this error and appreciate the reviewer's careful evaluation, which helped us identify and correct the issue.

Fig. 4f of the revised manuscript:

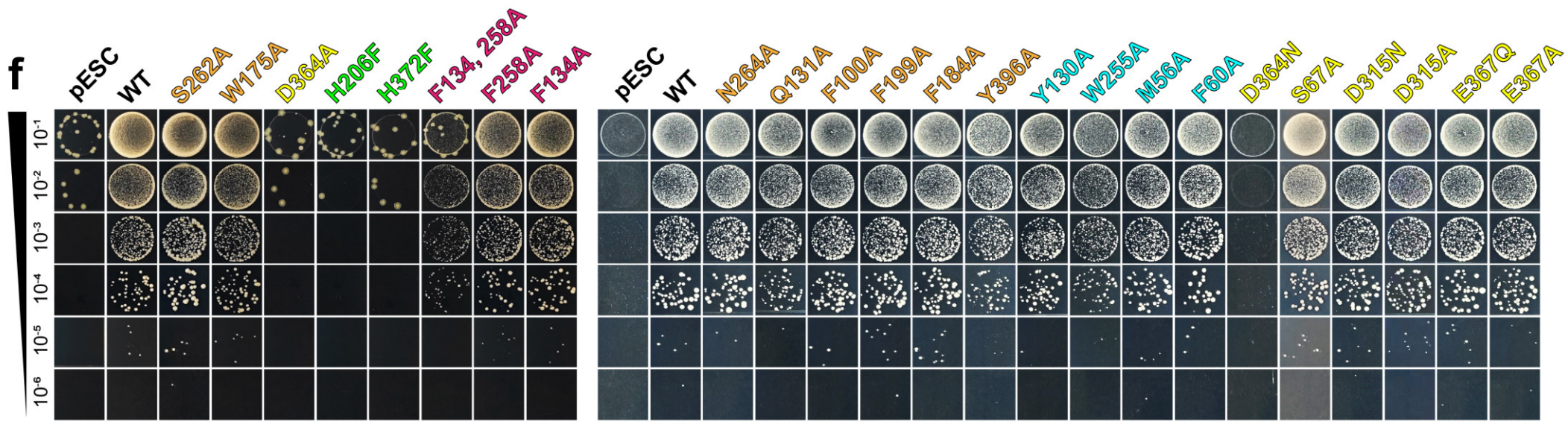

I think the authors need to establish a more sensitive assay or undertake more detailed uptake kinetics to fully understand the role of these side chains in the function of AMT1.1. As it stands, the data shown in Fig. 4 does not really provide much additional insight.

Response: We thank the reviewer for this important suggestion and agree that the yeast complementation assay has relatively limited resolution for dissecting the precise mechanistic contributions of individual side chains. As the reviewer noted, this assay is more suitable as a general functional screening approach than for detailed kinetic characterization.

We apologize that, due to limitations in time, experimental conditions, and available resources, we were unable to perform more quantitative electrophysiological measurements or comprehensive transport kinetic analyses for all mutants in the current revision. Nevertheless, we made several efforts in the revised manuscript to strengthen the functional interpretation.

First, we substantially expanded the mutational analysis of the transport pathway and included additional validation experiments **(as now Fig. 4 and is also see below)**. Second, we extensively incorporated and discussed previous functional studies of homologous AMT/Amt proteins and previously reported mutants of *At*AMT1;1. The revised discussion now compares our observations with earlier biochemical, electrophysiological, and mutational studies, helping place the present results into a broader mechanistic context **(Lines 287-329)**.

Fig. 4f of the revised manuscript:

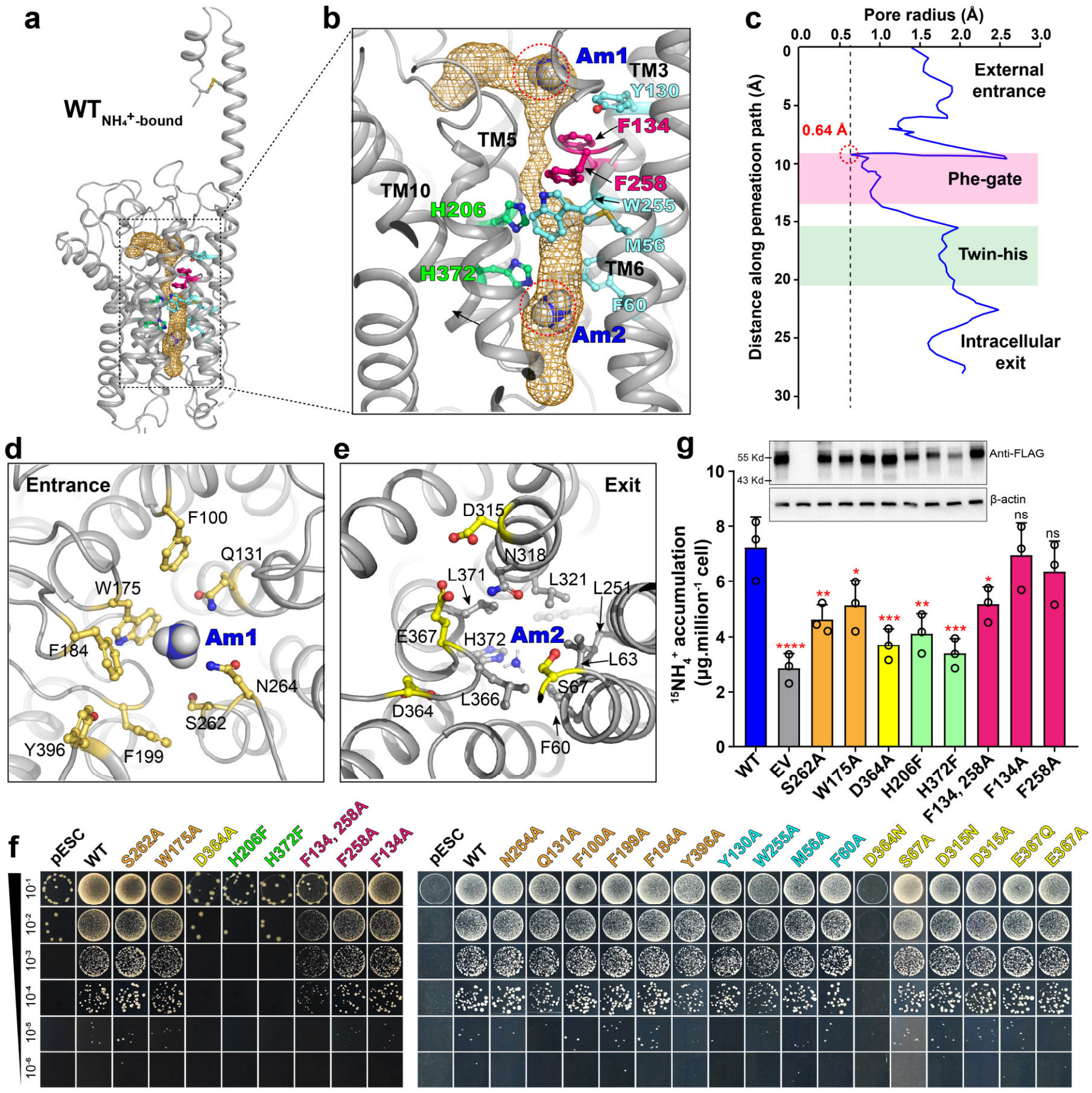

Finally, in response to the reviewer's concerns, we also moderated several mechanistic interpretations throughout the manuscript. In the revised version, the main focus is no longer the assignment of highly specific roles to individual pore residues. Instead, we emphasize the overall transport pathway architecture together with the regulatory mechanisms mediated by the N-terminal disulfide bond and the C-terminal regulatory domain, which are more strongly supported by the current structural and functional data. We hope these revisions improve the rigor and balance of the manuscript.

Figure 5: I am also not convinced by the conclusions drawn from the structures of AMT1.1 under neutral and acidic conditions. The structural changes are relatively minor and occur in loop regions. These could easily be attributed to changes in grid preparation or electrostatics. How these relate to any impact of pH under physiological conditions is not clear. This section of the paper is very preliminary in my opinion and probably needs further studies, maybe using molecular dynamics, and biochemical analysis to draw any firm mechanistic conclusions on the role of the pH gradient in AMT1.1 function.

Response: We thank the reviewer for this thoughtful comment and agree that the pH-dependent structural differences observed in *At*AMT1;1 are relatively subtle and do not support a model involving large-scale conformational rearrangements. In the revised manuscript, we therefore substantially moderated our interpretation and avoided strong mechanistic conclusions regarding pH-dependent transport regulation.

As the reviewer pointed out, the overall structures determined under different pH conditions are highly similar. Pore-radius analysis further revealed that acidic conditions slightly narrow the Phe-gate while modestly widening the exit vestibule **(as now Extended Date Fig. 5b, c and is also see below)**.

To further assess whether these observations reflect meaningful dynamic differences rather than noise arising from sample preparation or electrostatic effects, we performed molecular dynamics simulations under different pH conditions **(as now Extended Date Fig. 5d, e and is also see below)**. The simulations showed comparable overall structural stability, consistent with the cryo-EM structures, but revealed increased local fluctuations in the L2, L5, and L6 loop regions under acidic conditions. These results support the idea that extracellular pH may primarily influence local conformational dynamics rather than inducing major structural rearrangements.

Accordingly, we have revised the text to emphasize that our current data support a correlation between extracellular pH, local structural dynamics, and transport efficiency, but do not yet establish a definitive mechanistic model for pH coupling in *At*AMT1;1. We agree that further biochemical, electrophysiological, and computational studies will be required to fully resolve the molecular basis of pH-dependent regulation.

Fig. 5, a-e of the revised manuscript:

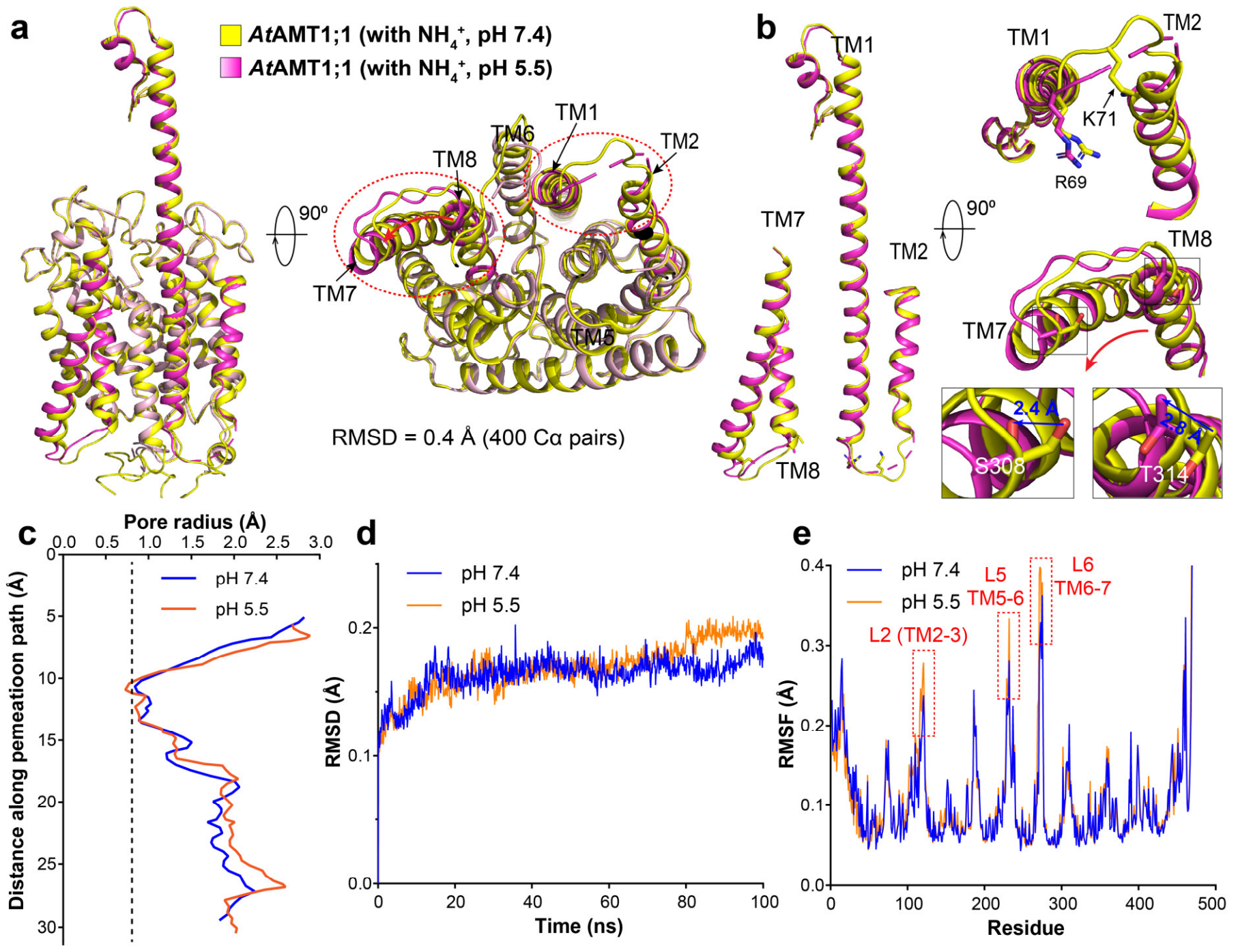

Extended Data Fig. 12: The authors need to show the expression of the mutants shown in panel a. The reduced growth in the yeast could be due to reduced transport or toxicity related to protein folding/UPR.

Response: We thank the reviewer for this important concern. We agree that reduced yeast growth could potentially result either from impaired transport activity or from indirect effects such as protein misfolding, instability, or cellular stress responses.

Although we did not perform western blot analysis in yeast cells, we carefully assessed the biochemical properties of the corresponding mutants expressed in HEK293 cells. In the revised manuscript, we now include additional size-exclusion chromatography and SDS–PAGE analyses showing that the mutants (including T460D and S450D) as well as the C-terminal truncation variants exhibit robust expression and favourable purification behaviour comparable to wild-type *At*AMT1;1. Notably, several C-terminal truncation variants even showed higher expression levels than the wild-type protein.

Furthermore, the purified proteins did not exhibit obvious aggregation or degradation during purification, suggesting that these mutations do not cause severe folding defects or major protein instability. In addition, mass photometry analysis showed that the C-

terminal truncations retained stable oligomeric assembly properties comparable to the wild-type transporter **(as now Extended Date Fig. 14b and is also see below)**.

Together, these observations suggest that the reduced yeast growth phenotypes observed for the T460D mutant and the C-terminal truncation variants are more likely associated with altered transport regulation rather than general defects in protein expression or folding. Nevertheless, because we did not directly assess protein expression levels in yeast cells, we cannot completely exclude possible contributions from altered membrane trafficking or yeast-specific quality-control pathways.

Extended Date Fig. 14b of the revised manuscript:

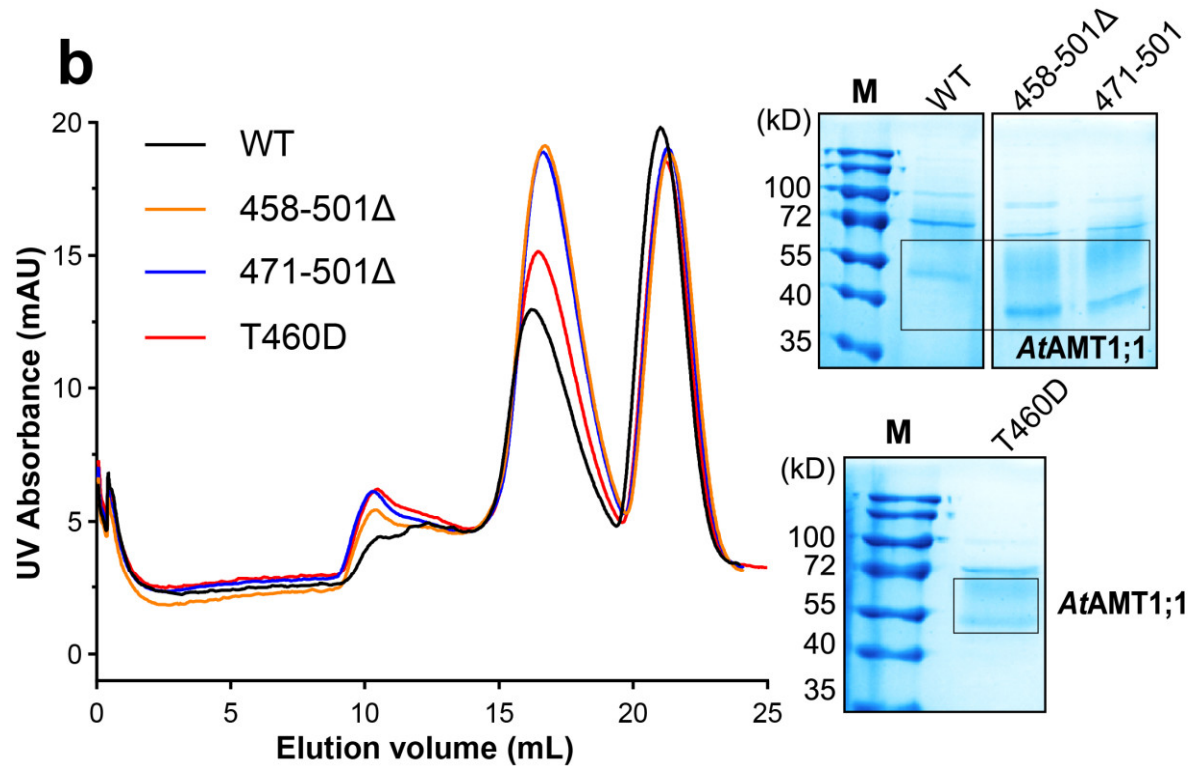

Lines 255-308: This section of the study reports the structure of the T460D mutant and the possible impact on the cytoplasmic loop between E220 and S240. I found this section to be far too speculative given the current data. The authors offer no credible allosteric mechanism linking the structural impact of the T460D mutant and the cytoplasmic loop. These changes may be meaningfully correlated or not. I was unconvinced by this data, and the interpretation appears over interpreted. To validate the proposed model the authors, need to design more accurate assays and I suggest, molecular dynamics is likely the best place to start. Alternatively, perhaps solving the structure in nanodiscs may increase the resolution? I also wondered whether the structural models and maps presented in Extended Data Fig. 12 and Figure 6 were from the C3 averaged structures? If so, the authors should probably determine the C1 structures and see if these changes are unique to each monomer?

Response: We thank the reviewer for this thoughtful and constructive comment. We agree that the original version overstated the mechanistic conclusions that could be

drawn solely from the cryo-EM densities. In the revised manuscript, we therefore substantially moderated our interpretation and added additional analyses to better support the proposed regulatory model.

First, following the reviewer's suggestion, we performed molecular dynamics simulations comparing wild-type *At*AMT1;1 and the phosphomimetic T460D mutant **(as now Extended Date Fig. 14f and is also see below)**. The simulations showed that the overall structural stability of the T460D mutant remained highly similar to that of the wild type, consistent with the cryo-EM structures. However, RMSF analysis revealed a clear increase in local flexibility within the L5 region (residues 220–240) in the T460D mutant **(as now Extended Date Fig. 14g and is also see below)**. These results are consistent with our structural observation that the C-terminal regulatory domain and the adjacent L5 loop become less well resolved in the T460D structure, suggesting increased conformational dynamics rather than large-scale structural rearrangement.

Extended Date Fig. 14f, g of the revised manuscript:

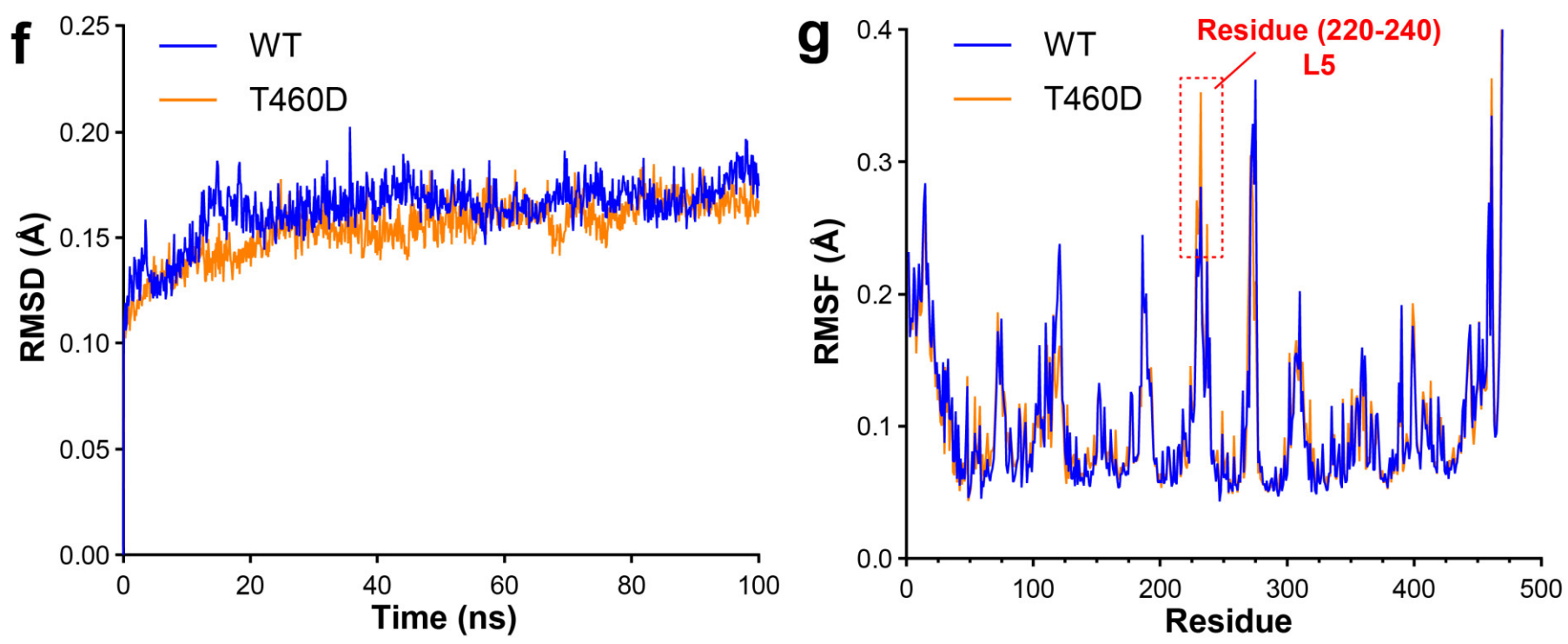

Importantly, in the revised manuscript we no longer claim a definitive allosteric mechanism linking T460 phosphorylation to transporter inhibition. Instead, we now describe the data more cautiously and emphasize that phosphorylation at T460 appears to perturb the interaction network between the C-terminal regulatory domain and intracellular loops, thereby altering local dynamics.

We also agree with the reviewer that higher-resolution structures would strengthen the analysis. We attempted to reconstitute *At*AMT1;1 into nanodiscs for cryo-EM analysis; however, the reconstitution quality was insufficient for high-resolution reconstruction. As an alternative, we collected substantially more cryo-EM data for both WT and

T460D samples and further improved the reconstructions **(as now Extended Date Fig. 5a, b and Extended Date Fig. 9c)**. In the revised version, both WT and T460D structures refined with C1 symmetry reach resolutions better than 3 Å, allowing clearer visualization of local structural features. Overall, the C1 and C3 maps were highly similar, and no obvious asymmetric conformational differences among individual protomers were observed **(Extended Date Fig. 5b, and is also presented in panel a below)**. In the WT structure, the C-terminal regulatory domain (CRD) density could be resolved under both C1 and C3 symmetry, although the density was substantially clearer in the C3 reconstruction **(Presented in panel b below)**. In contrast, the CRD density in the T460D mutant remained relatively weak under both C1 and C3 symmetry, suggesting increased flexibility of this region rather than symmetry-imposed averaging artifacts **(Presented in panel c below)**. Because the C3 reconstructions provided substantially improved local density quality for the CRD region while remaining fully consistent with the C1 maps, we therefore used the C3 maps for subsequent structural analysis and model interpretation.

The EM densities analysis of WT and T460D：

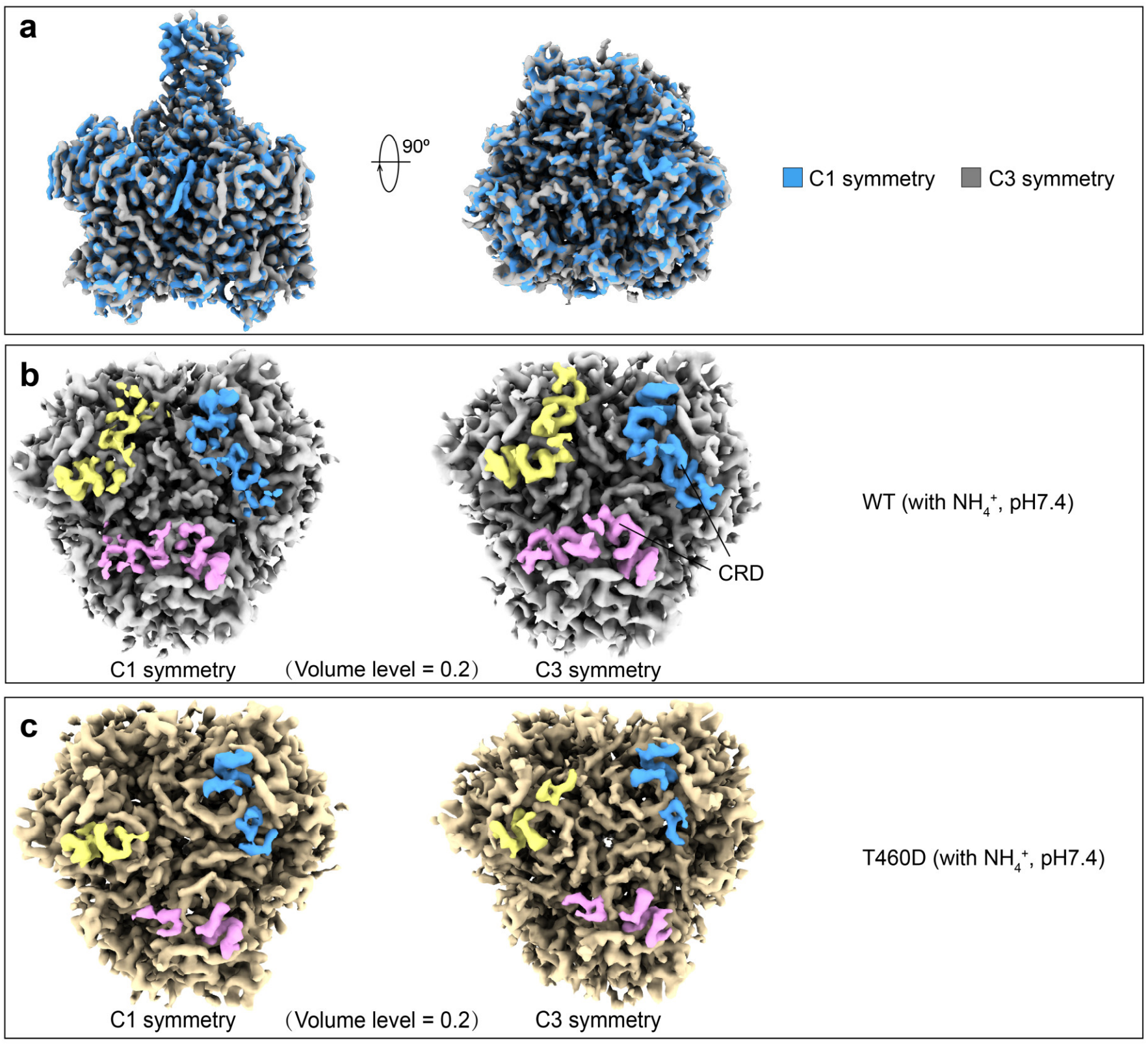

Reviewer #3:

Review He et al., Nature Comm.

Structural basis of ammonium transport and phosphorylation-mediated regulation of AMT1;1 in *Arabidopsis thaliana* Ammonium serves as an important nitrogen form for microbes and plants. AMTs had been shown to be responsible for ammonium acquisition across a wide range of species. There is extensive experimental evidence including transport studies, studies of mutants, yeast and plant mutants and evidence for the role of kinases in regulation via phosphorylation. Also, activity sensors had been developed. Furthermore, evidence had been presented that AMTs may function as transceptors, with dual roles in Am transport and signaling. Moreover, several structures have been published for diverse family members. Notably extensive mutagenic studies to characterize the role of individual residues for transport function, notably interaction of loop regions with the conserved C-terminus required for activity. It is thus not clear why the authors emphasize that the function Poorly understood. While the reviewer is not a structural biologist, this is a appears to be a very nice piece of work providing not only cryoEM structures of the Arabidopsis AMT1, but also that of mutant forms, e.g. the important T460D-terminal. It would be recommended to involve structural experts in the review process.

Key results presented here:

- A 1.2 Å structure of AMT1 from Arabidopsis and validation using a variety of mutants, however without properly including all the known mutant and modeling studies (Nygaard, Graff et al., various Loque publications, Yuan et al et.; possibly useful to also look at the supplemental data in these publications). While some of the references are in the bibliography or Discussion, the interpretation and even the experiments here are based or show what was predicted or shown before. For example Phe gate is even a paragraph in reviews from 2010 (www.sciencedirect.com/science/article/pii/S124678201000039X). This is not to criticize the excellent structural work, rather the way the results are presented here as original.

- Structures at two pH conditions show potential differences with TM7 – TM8 with an outward deflection, resulting in a wider opening on the intracellular side. The authors conclude that pH plays a role in regulating the activity, and use growth assays to evaluate the difference at different pH. Notably, pH dependency had been shown previously using transport assays, with results not in line with the observation shown here (Ninnemann et al., 1994). Growth assays are not likely a suitable tools for evaluating pH dependencies of specific proteins.

- Demonstration of N-terminal truncations cause defect. This is interesting, yet not unexpected and does not appear to represent a major new insight.

- Disulfide bridge important – It may be useful here to mention literature that analyzed the role of reducing agents, or even observed the cysteines in structures.

- Demonstration that T460 in the conserved C-terminus is important (this is well known and the mechanism is very well studied). The text gives the impression as if this was discovered here.

Response: We thank the reviewer for the thoughtful and balanced evaluation of our work, as well as for recognizing the value of the structural analysis presented in this study. We fully agree that the AMT/MEP/Rh family has been extensively investigated over the past decades through genetics, transport assays, mutagenesis, physiological studies, and structural biology. In the original version, some of our wording may indeed have overstated the novelty of certain functional concepts or insufficiently acknowledged previous work. We have carefully revised the manuscript to better place our findings into the context of the extensive existing literature.

First, we have substantially revised the Introduction and Discussion to more comprehensively acknowledge previous mechanistic and mutagenesis studies on AMT transporters. We now explicitly discuss prior studies on the Phe-gate, Twin-His motif, intracellular loops, C-terminal regulation, and phosphorylation-dependent inhibition. Throughout the revised manuscript, we have carefully distinguished between previously established concepts and the new structural observations provided in this work.

We also agree that several functional features described here, including the importance

of the conserved C-terminal phosphorylation site T460, were already well established in previous studies. In the revised version, we no longer present these findings as novel discoveries. Instead, we emphasize that our study provides structural insight into how phosphorylation at T460 may remodel the interaction network between the C-terminal regulatory domain and intracellular loop regions. Similarly, we now describe the N-terminal disulfide bond as a conserved structural and potentially regulatory element whose functional importance is consistent with earlier observations on redox sensitivity and cysteine conservation in AMT proteins.

Regarding the pH-dependent structural analysis, we appreciate the reviewer's caution. In the revised manuscript, we substantially moderated our interpretation and no longer propose strong mechanistic conclusions regarding pH coupling. We now explicitly acknowledge that the observed conformational differences are relatively subtle and may partially reflect local electrostatic or protonation-state effects. To strengthen this section, we additionally performed molecular dynamics simulations and pore-radius analyses, which support the idea that extracellular pH influences local structural dynamics rather than inducing major conformational rearrangements. We also acknowledge the limitations of yeast growth assays for quantitatively evaluating pH dependence and now discuss these data more cautiously in the context of previous transport studies.

Importantly, our revised manuscript shifts its emphasis away from proposing a definitive transport mechanism and instead focuses more strongly on the structural basis of regulation. In particular, we believe the structures of the phosphomimetic T460D mutant, combined with mutagenesis, biochemical analysis, and molecular dynamics simulations, provide useful structural insight into the conserved regulatory role of intracellular loop regions and the C-terminal regulatory domain in plant AMT transporters.

Overall, we greatly appreciate the reviewer's comments, which helped us substantially improve the accuracy, balance, and presentation of the manuscript.

Major points:

Structures of AMT homologs had been obtained previously. Novel here is that we deal with a plant transporter, for which a critical role of the C-terminus had been

demonstrated. An advance here is that the C-terminus is includes in the structure of the eukaryotic protein (the C-terminus was also structurally determined in the AMT from A. fulgidus. Major gaps in our knowledge relate to the transport mechanism, that is the conformational rearrangements in a transporter during the transport cycle. This study provides no new insights into the transport cycle.

Response: We thank the reviewer for this important clarification. We fully agree that structures of AMT/MEP/Rh family transporters have been reported previously, including the *Archaeoglobus fulgidus* Amt1 structure in which the C-terminal region was also resolved. Although the C-terminal region was structurally resolved in the archaeal *Af*Amt1 transporter, its regulatory mechanism appears fundamentally different from that of plant AMT1 proteins. In plant AMT1 transporters, the conserved C-terminal regulatory domain functions as a phosphorylation-dependent module. By contrast, such a phosphorylation-mediated regulatory mechanism has not been established for *Af*Amt1. In the revised manuscript we emphasize that the main advance of our study is the structural characterization of a plant AMT1 transporter together with its phosphorylation-associated regulatory state, including the phosphomimetic T460D mutant, which provides structural insight into how the conserved C-terminal regulatory domain interacts with intracellular loop regions **(as now Fig. 6, c-f and is also see below)**.

Fig. 6, c-f of the revised manuscript:

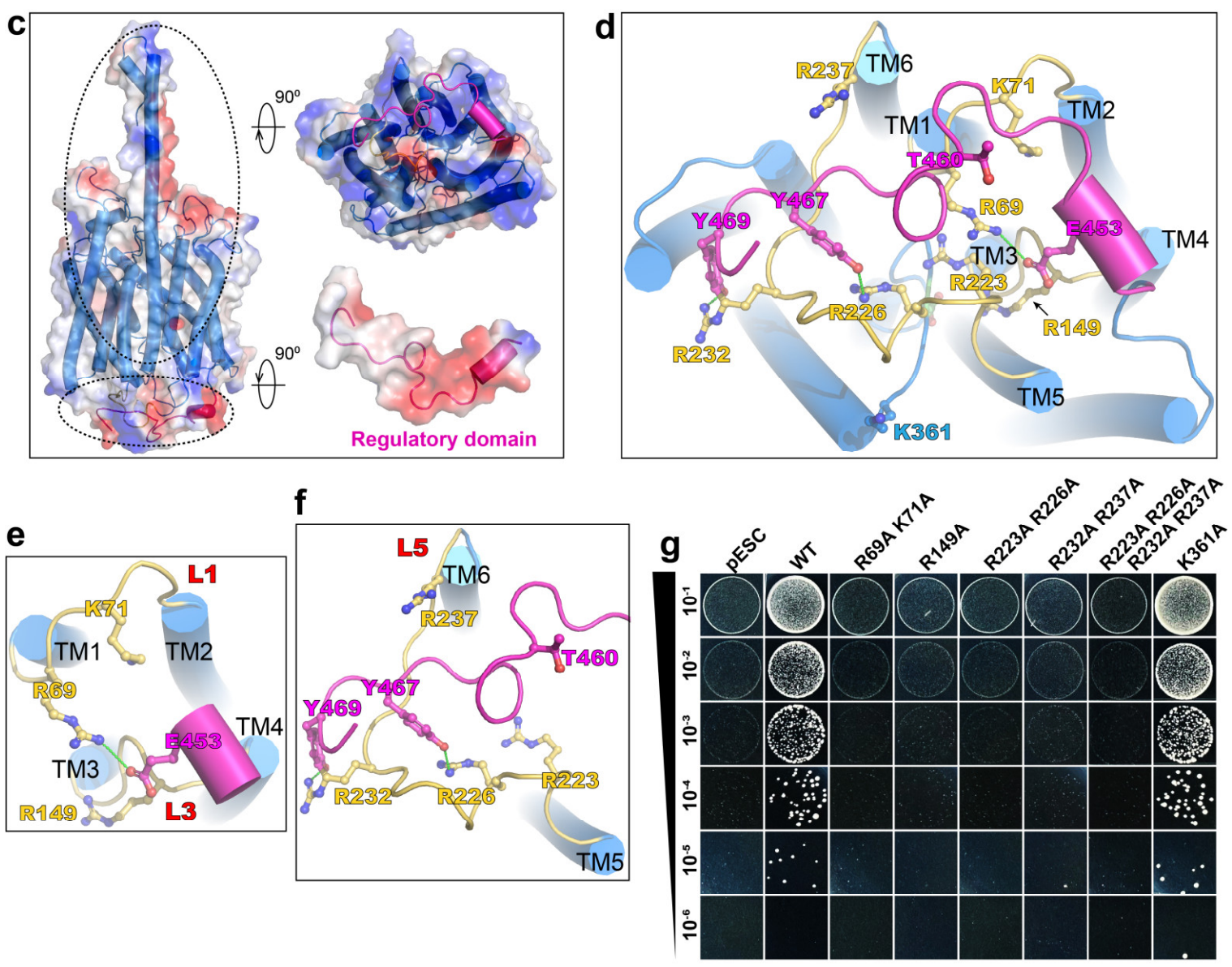

We agree that one of the major unresolved questions in the AMT/MEP/Rh field concerns the transport mechanism itself, particularly whether transport involves large-scale conformational rearrangements and how ammonium/ammonia is translocated through the pore during the transport cycle.

Indeed, based on the currently available structures from bacterial, archaeal, fungal, and the structures reported here, there is so far little evidence for major global conformational changes associated with transport. At the same time, the precise molecular mechanism of ammonium transport remains highly controversial and technically challenging to resolve. Early structural and functional studies of bacterial AmtB proposed that the transporter mediates conduction of neutral NH_3 after deprotonation of NH_4^+ within the pore. However, later electrophysiological studies demonstrated electrogenic transport activity, implying movement of net charge across the membrane and challenging the simple NH_3-diffusion model. In plant AMT1 transporters, the situation appears even more complex, as previous electrophysiological and functional studies have suggested diverse transport behaviors and possible mechanistic differences among AMT proteins.

Given these unresolved controversies, we substantially revised the manuscript to avoid overinterpretation of the current structural data. In the revised version, we no longer attempt to define a complete transport-cycle mechanism or explicitly assign substrate protonation states during transport. Instead, we focus more conservatively on describing the overall substrate-conduction pathway and identifying structurally and functionally important residues along this pathway through combined cryo-EM analysis, mutagenesis, isotope uptake assays, and comparison with previous studies.

Although some of these residues had been investigated previously, we believe the present study provides one of the more structurally comprehensive analyses of the putative transport pathway in a plant AMT transporter. Importantly, our structures provide a framework for integrating earlier mutagenesis and physiological observations and for guiding future mechanistic studies aimed at resolving how ammonium transport actually occurs in AMT-family proteins.

We fully agree that our study does not resolve the complete transport mechanism.

However, we hope that the structural framework, comparative analysis, and regulatory insights presented here will provide a useful foundation for future biochemical, electrophysiological, computational, and plant physiological investigations of AMT-family transporters.

Notably, the authors refer almost exclusively to their own mutations shown here, but do not discuss the ample number of mutagenic studies which have been published before, not do they consider them here. It appears as if the authors were the first ones to identify the role of T460, or the interaction of the C-terminus and the cytosolic domains (specific residues were known for both).

Response: We thank the reviewer for this important comment. We fully agree that the original manuscript insufficiently discussed the extensive previous mutagenesis and regulatory studies on AMT-family transporters, particularly regarding the conserved phosphorylation site T460 and the functional interplay between the C-terminal region and intracellular loop domains.

In the revised manuscript, we have substantially expanded the discussion of previous work and carefully revised the wording throughout the text to avoid giving the impression that these regulatory features were first identified in this study. We now explicitly acknowledge that phosphorylation of the conserved T460 site and the essential role of the C-terminal regulatory region in AMT1 activity had already been convincingly demonstrated in earlier studies. We also now discuss previous studies on bacterial AmtB and fungal Mep2 transporters, in which intracellular loop regions were proposed to participate in transporter regulation. In bacterial AmtB, interaction with the regulatory protein GlnK was shown to alter intracellular loop conformations and inhibit transport activity, whereas in fungal Mep2, intracellular loops and the C-terminal region were implicated in phosphorylation-dependent autoinhibitory regulation **(as now Extended Date Fig. 15, a-d and is also see below)**.

Importantly, while these previous studies established the functional importance of these regulatory regions, the underlying structural basis and potential common regulatory principles across kingdoms remained less clear. In the revised manuscript, we therefore position our contribution more carefully: through comparative structural analysis of

plant *At*AMT1;1 together with bacterial and fungal homologs, combined with mutagenesis and molecular dynamics simulations, we propose that intracellular positively charged loops may function as conserved regulatory elements within the AMT/MEP/Rh superfamily. In plants and fungi, phosphorylation-dependent modulation of the C-terminal region may alter loop dynamics, whereas in bacteria, interaction with GlnK may similarly regulate transporter activity by affecting intracellular loop conformations **(as now Extended Date Fig. 15, a-d and is also see below)**.

Thus, rather than presenting the identification of T460 or C-terminal regulation as novel discoveries, the revised manuscript now focuses on providing structural insight into how these previously established regulatory modules may function at the molecular level.

Extended Date Fig. 15 of the revised manuscript:

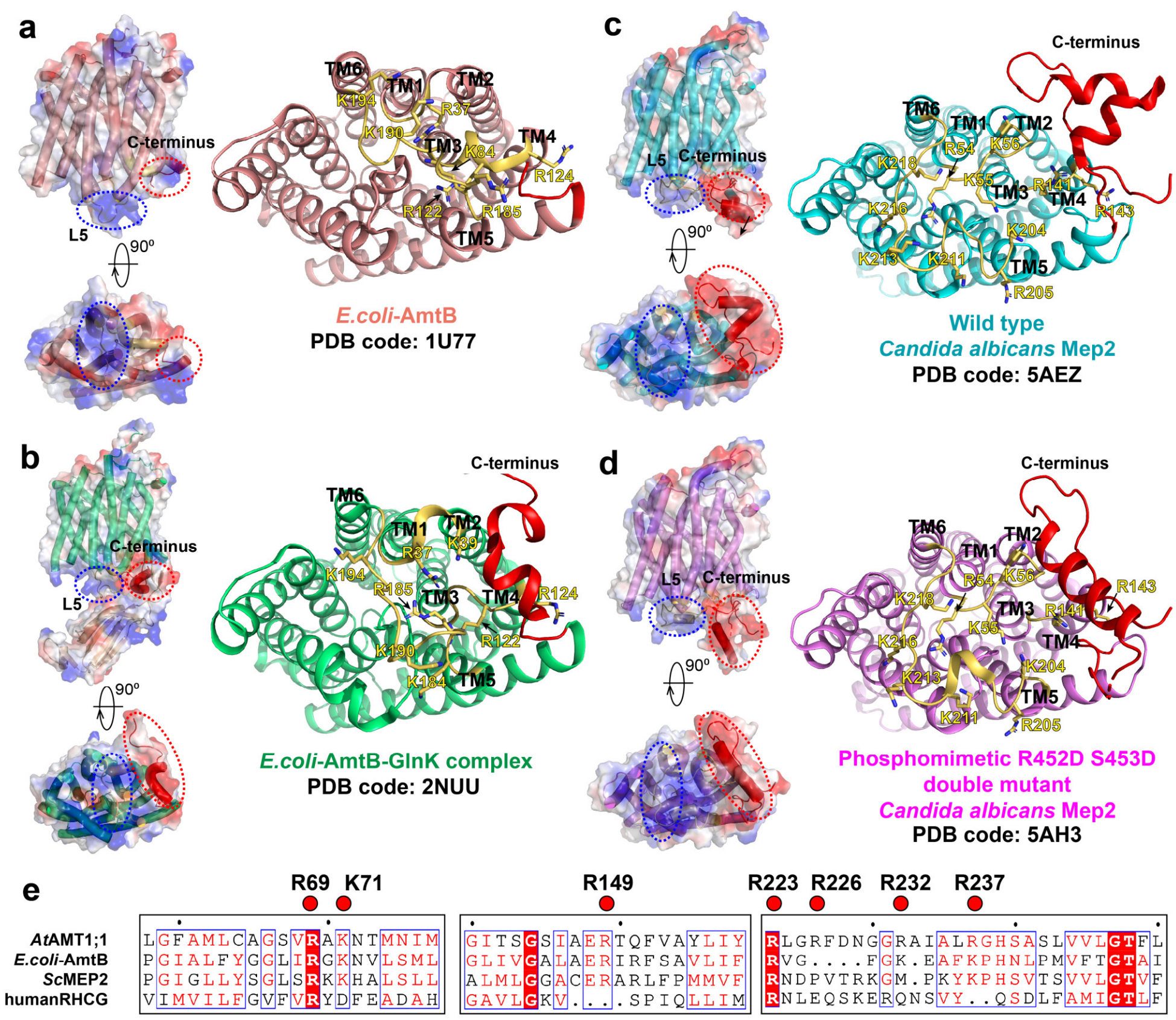

There are multiple kinases and phosphorylation sites known, which are not discussed here.

Response: We thank the reviewer for this important comment. We agree that multiple

phosphorylation sites and kinases involved in AMT1 regulation have been reported previously, and the original manuscript did not sufficiently discuss this broader regulatory context. In the revised manuscript, we now clarify that several phosphorylation sites have been identified in plant AMT1 transporters. However, among these sites, the functional characterization of S450 and T460 has been the most extensive and reproducible across previous studies. Earlier work demonstrated that phosphorylation at S450 is associated with enhanced transport activity, whereas phosphorylation at the conserved T460 site triggers strong inhibition or trans-inactivation of AMT1 transporters.

Importantly, despite these previous physiological and biochemical studies, the structural basis underlying how phosphorylation modulates AMT1 activity has remained largely unknown. Therefore, rather than attempting to comprehensively address all reported phosphorylation events, our study focuses specifically on the structurally and functionally important T460-centered regulatory region and its associated intracellular interaction network.

Through structural analysis of the phosphomimetic T460D mutant, mutagenesis, and molecular dynamics simulations, we provide preliminary mechanistic insight into how phosphorylation-associated remodeling of the C-terminal regulatory domain may alter intracellular loop dynamics and thereby modulate transporter activity. We believe that this structural framework may also provide useful reference information for future studies investigating additional phosphorylation sites and kinase-dependent regulatory pathways in plant AMT transporters.

To better reflect the current state of knowledge, we have revised the text throughout the manuscript to explicitly acknowledge previous phosphorylation studies and to avoid implying that phosphorylation-dependent regulation was first identified here.

Key question not resolved regarding the transport mechanism, ammonia, ammonium or proton symport.

Response: We fully agree with the reviewer that one of the central unresolved questions in the AMT/MEP/Rh field concerns the precise transport mechanism, including whether these transporters conduct NH_4^+, NH_3, or involve proton-coupled transport

processes. Despite extensive structural, electrophysiological, and functional studies over the past two decades, this issue remains highly controversial and technically challenging to resolve, particularly in plant AMT1 transporters.

Indeed, previous studies have produced partially conflicting models. Early bacterial AmtB studies proposed deprotonation of NH_4^+ and transport of neutral NH_3, whereas subsequent electrophysiological analyses demonstrated electrogenic transport activity, implying net charge movement across the membrane. In plants, the situation appears even more complex, as different AMT1 transporters may exhibit distinct transport behaviors depending on experimental systems and physiological conditions. Given these unresolved complexities, we substantially revised the manuscript to avoid overinterpretation of our structural data. In the revised version, we no longer attempt to define whether the transported species is NH_4^+, NH_3, or a proton-coupled intermediate. Instead, we use the more neutral term “Am” or “substrate” when discussing the transport pathway.

Accordingly, the revised study focuses more conservatively on defining the overall conduction pathway and identifying structurally and functionally important residues along this pathway through combined cryo-EM analysis, mutagenesis, molecular dynamics simulations, and transport assays. Although this work does not resolve the complete transport mechanism, we believe that establishing a structural framework for the transport pathway and regulatory architecture of plant AMT1;1 is still important for future mechanistic studies, plant functional analyses, and potentially crop-improvement research aimed at optimizing ammonium uptake and nitrogen-use efficiency.

Also not mentioned nor studied is the evidence that AMTs may serve as transceptors, so possibly.

<u>Response:</u> We appreciate the reviewer’s suggestion regarding the recent work by Pflueger et al. (Science Advances, 2024). In that study, the authors characterized *Shewanella denitrificans* Amt1 (Sd-Amt1), an unusual Amt-family protein fused to a C-terminal diguanylate cyclase signaling domain, and proposed that it may function as both a transporter and receptor (“transceptor”) through ammonium binding at regulatory sites. In the revised manuscript, we have now carefully analyzed and

discussed the potential transceptor-like properties of plant AMT1 transporters. Structural comparison revealed that *At*AMT1;1 shares substantial similarity with the bacterial transceptor *Sd*-Amt1, including conservation of several residues surrounding the proposed N1 and N2 ammonium-binding sites **(Extended Data Fig. 16, d-f)**. However, unlike *Sd*-Amt1, no obvious substrate-associated densities were observed at the equivalent N1 and N2 positions in *At*AMT1;1, and residues surrounding these regions did not exhibit clear conformational rearrangements among the *At*AMT1;1 in different states **(Extended Data Fig. 16, g-i)**. Therefore, while our current data do not provide direct evidence that plant AMT1;1 functions as a transceptor, the observed structural conservation suggests that this possibility cannot be excluded. We have now included a dedicated discussion of this hypothesis and its limitations in the revised Discussion section **(Lines 525-545)**.

Extended Data Fig. 16 of the revised manuscript:

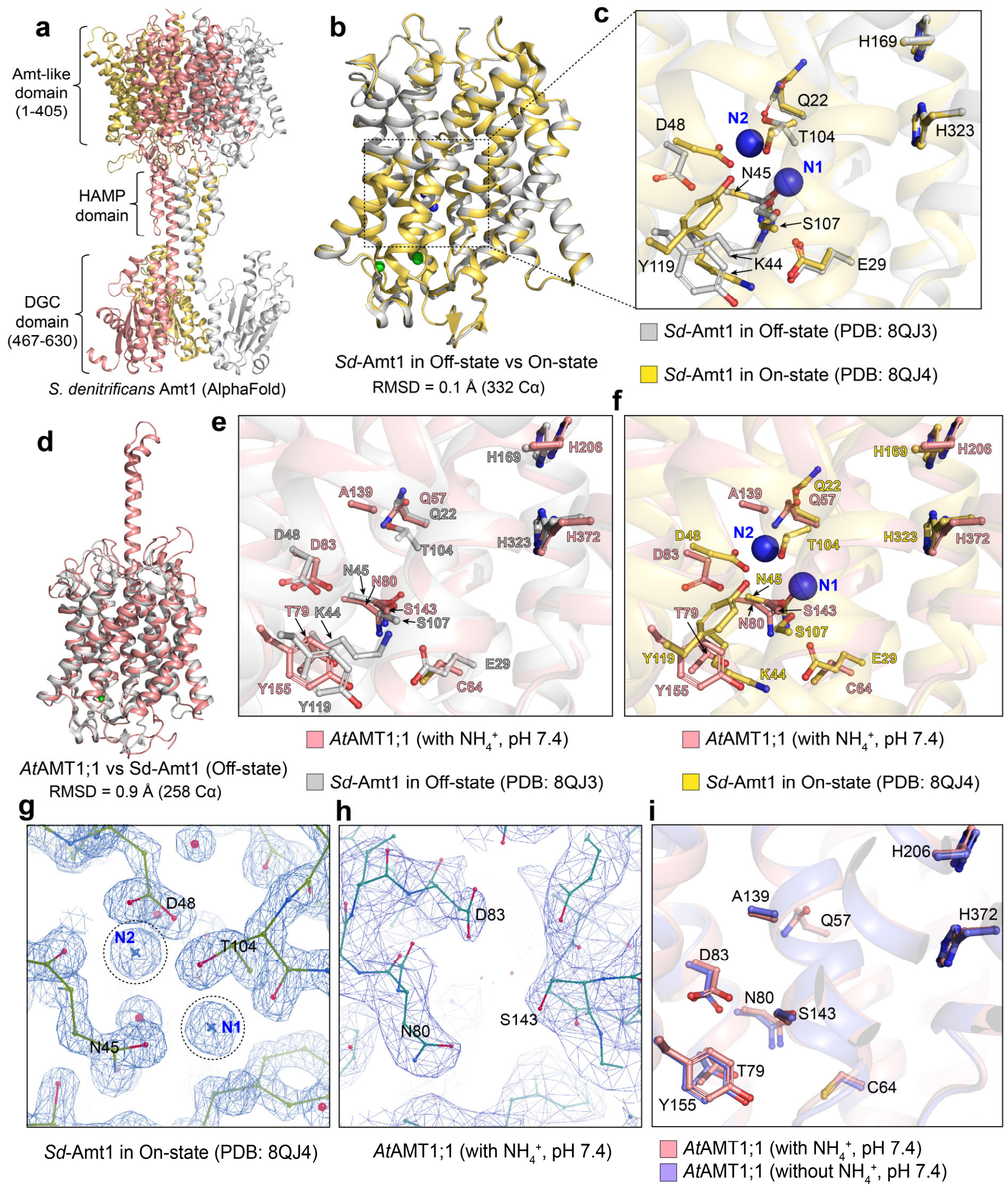

Electrophysiological evidence for transport of charge not considered here.

Response: We thank the reviewer for this important comment. We agree that electrophysiological evidence supporting electrogenic transport behavior in AMT transporters is highly important and should be properly discussed in the context of our structural analysis. In the revised manuscript, we have substantially expanded both the Introduction **(Lines 49-64)** and Results **(Lines 212-216)** sections to better summarize and discuss previous electrophysiological studies.

Specifically, we now explicitly discuss that several plant AMT1 transporters, including *At*AMT1;1, *Le*AMT1;1, and *Os*AMT1;1, exhibit strong electrogenic ammonium transport currents consistent with NH_4^+-associated charge movement. We also discuss that different AMT family members have been proposed to operate through distinct transport modes, including NH_4^+ uniport, NH_3/H^+ symport, electroneutral NH_3

transport, and NH_4^+/H^+ cotransport. These observations highlight the mechanistic diversity and complexity of plant ammonium transport systems. In addition, the revised Results section now explicitly acknowledges that early bacterial AmtB studies proposed an NH_3 gas-channel model, whereas subsequent electrophysiological analyses demonstrated electrogenic and pH-dependent transport properties, arguing for a more complex mechanism than simple NH_3 diffusion.

We appreciate the reviewer's comment, which helped us better integrate the structural observations presented here with the extensive electrophysiological literature on AMT-family transporters.

Minor points:

Aspartate is typically referred to as a mimic of phosphorylated Ser or Thr. However the structures and properties are very different. Thus while one may be able to cautiously state that if we assume that however the reviewer would suggest not to state that this demonstrates the effect of phosphorylation. The statement: "Following T460 phosphorylation, the C-terminus becomes disordered" and others should be cautioned accordingly.

Response: We thank the reviewer for this important and careful comment. We fully agree that aspartate substitution cannot fully reproduce the chemical and structural properties of a phosphorylated threonine residue. Although Asp mutations are widely used as phosphomimetic substitutions, they only partially mimic the negative charge introduced by phosphorylation and do not recapitulate the steric, electrostatic, or hydrogen-bonding properties of a true phosphate group.

Accordingly, we have substantially revised the wording throughout the manuscript to avoid overinterpretation of the T460D structure. In the revised version, we no longer state that the T460D mutant directly demonstrates the structural consequences of physiological phosphorylation. For example, statements such as "Following T460 phosphorylation, the C-terminus becomes disordered" have been revised to more conservative wording such as "In the phosphomimetic T460D mutant, the C-terminal region." Similar revisions were made throughout the Results, Discussion, and figure legends.

We appreciate the reviewer's comment, which helped us substantially improve the precision and rigor of our interpretation.

Overall, while this is a very nice and extensive study, and the first to provide a full structure including the soluble C-terminus at high resolution. Even though the structures appear to be very good, the work does not appear to provide many novel insights into the mechanism nor represent a major advance over the previously published work.

Response: We thank the reviewer for the thoughtful evaluation of our work and fully appreciate the concern regarding the degree of mechanistic advance relative to previous studies in the AMT field. We agree that our study does not resolve the complete transport mechanism of AMT-family transporters. As discussed above, the molecular basis of ammonium transport — including whether transport occurs as NH_4^+, NH_3, or through proton-coupled mechanisms — remains highly controversial and technically challenging to address.

Nevertheless, we believe the present study provides several advances that are important for the plant AMT field and for understanding regulation within the AMT/MEP/Rh superfamily.

First, to our knowledge, this work presents the first high-resolution structural analysis of a plant AMT1 transporter containing a well-resolved C-terminal regulatory domain. Although the archaeal *Af*Amt1 structure also included a resolved C-terminus, the regulatory architecture of plant AMT1 proteins is fundamentally different because plant AMT1 transporters undergo phosphorylation-dependent trans-inactivation through the conserved C-terminal regulatory region.

Second, our study identifies and structurally characterizes a conserved extracellular N-terminal disulfide bond that is required for protein stability and transport activity. Combined with redox perturbation experiments, these findings raise the possibility that plant AMT1 proteins may function as redox-responsive nutrient transporters. To our knowledge, this aspect has not previously been structurally analyzed in plant AMTs.

Finally, although previous studies investigated individual pore residues, our revised manuscript now focuses more broadly on systematically analyzing the overall

substrate-conduction pathway of a plant AMT1 transporter through combined structural analysis, mutagenesis, isotope uptake assays, and comparison with bacterial and fungal homologs. While some individual residues were previously studied, we believe the present work provides one of the most structurally integrated analyses of the putative transport pathway in a plant AMT transporter.

Accordingly, in the revised manuscript we substantially moderated our mechanistic claims and shifted the focus away from proposing a definitive transport mechanism toward providing a structural and regulatory framework for future mechanistic, physiological, and crop-improvement studies.

We sincerely thank the reviewers for their careful assessment of our manuscript and for their constructive comments and suggestions. We appreciate the opportunity to revise our work and have carefully considered all concerns raised during the review process. The comments have greatly helped us improve the clarity, rigor, and interpretation of our study. We have revised the manuscript accordingly and provide a detailed point-by-point response below:

Reviewer #1:

After major modifications, the manuscript is indeed much better. But since so much was reinterpreted and (re-)written, new issues need to be addressed.

Response: We sincerely thank the reviewer for recognizing the substantial improvements made in the revised manuscript. We appreciate the reviewer's careful re-evaluation and agree that the extensive reinterpretation and rewriting of several sections require careful consideration to ensure that all statements are appropriately supported by the data. We have carefully addressed the newly raised concerns, further refined our interpretations, moderated statements where necessary, and provided additional analyses or clarifications to improve the accuracy and rigor of the manuscript. Detailed responses to each comment are provided below.

The introduction and discussion of results is now much better, including some of the crucial literature and a correct description of the state of the art regarding the mechanistic understandings of ammonium transport in general, as well as particular aspects concerning *At*AMT1;1. On page 3 line 44: I would not say that the mechanism is still unclear. I would say that experimentally proving/disproving the best hypothesis is extremely challenging.

Accordingly, the sentence in page 5, lines 80 – 82 should be modified since the structures presented and analyzed do not elucidate the molecular mechanism of ammonium transport. Line 90 should also be altered because "facilitator" is a word best used to describe a passive, down-gradient, channel-like mechanism and this reviewer saw nowhere in the manuscript experiments that could proof this (or any other)

mechanism.

Response: We thank the reviewer for this insightful comment. We agree that the phrase "the mechanism remains unclear" was too broad and did not accurately reflect the current state of the field. Several transport models have been proposed based on previous electrophysiological and biochemical studies. However, experimentally distinguishing among these models and determining the precise chemical events underlying ammonium translocation remain highly challenging. We have therefore revised the Introduction accordingly **(Lines 44-46)**.

In addition, we agree that our structural analyses primarily provide a framework for understanding the substrate conduction pathway and regulatory mechanisms rather than directly resolving the complete molecular transport mechanism. We have modified the corresponding statements to avoid overinterpretation **(Lines 92-93)**.

We agree that the term "facilitates" may imply a passive, channel-like transport mechanism and could lead to ambiguity regarding the transport mode. We have therefore replaced "facilitates" with "mediates" throughout the manuscript to more accurately describe the functional role of *At*AMT1;1 without implying a specific transport mechanism **(Line 102)**.

While major improvements have been done to the current version, the concept of *At*AMT1;1 functioning as a transceptors is now briefly mentioned but remains poorly investigated (see attached file with figures included).

This point should be properly mentioned in the introduction (eventually, after line 77). This will make the description of result and later discussion easier to understand.

Response: We thank the reviewer for this important suggestion. We agree that the potential transceptor function of AMT proteins provides an important context for interpreting the structural features of *At*AMT1;1. In the previous revision, we mainly discussed this possibility in the Discussion section, but we agree that introducing this concept earlier would improve the overall logic of the manuscript. We have therefore added a paragraph in the Introduction describing the emerging evidence for AMT proteins as potential transceptors and highlighting that whether plant AMT1 proteins possess similar signaling functions remains unresolved **(Lines 80-88)**.

Despite all improvements the manuscript remains of questionable quality in the experimental data interpretation, presentation and discussion. In particular, the handling and analysis of structural data (major value of the paper) is surprisingly sloppy.

For example, the monomers in the trimers show a perfect 3-fold symmetry, while the map does not. The map shows variations that have not been taken into consideration. Moreover, all Ramachandran plots show perfect, ideal geometries, indicating that the authors may have refine (if at all) the data with very high restricted geometries. Potentially only one monomer was modelled (although I do not think so given that if I do it, I see changes adapting to the map), but certainly they did not work on the trimer - unbelievable.

Response: We thank the reviewer for raising this important concern regarding the symmetry treatment and model refinement of the *At*AMT1;1 structures. We would like to clarify that the initial atomic model was independently built and refined against the C1-symmetry reconstructed map, rather than generated by imposing three-fold symmetry on a single protomer.

To further evaluate whether the apparent three-fold symmetry resulted from over-restrained refinement, we performed additional structural analyses using the C1-reconstructed map. **As shown below in the Response Fig. 1**, the three protomers within the C1-derived trimer were individually compared. All three protomers exhibited highly similar overall conformations, with RMSD values ranging from 0.19 to 0.27 Å, indicating that the transmembrane core of *At*AMT1;1 is intrinsically highly symmetric. The observed differences were mainly localized to flexible regions, such as the intracellular L5 loop and C-terminal regulatory domain, where local density quality varied among individual protomers and allowed different extents of model building. We further compared the C1-derived model with the model refined against the C3-symmetry reconstructed map. The two models showed nearly identical transmembrane architectures (RMSD = 0.25 Å), with differences again restricted primarily to flexible peripheral regions such as the C-terminal regulatory domain. These analyses indicate that application of C3 symmetry did not introduce artificial conformational features or obscure biologically relevant asymmetry, but instead improved the map quality for

regions with limited local resolution. Therefore, for subsequent structural interpretation and visualization, we used the C3-refined model because it provides more reliable density interpretation, particularly for the conserved transmembrane transport core.

Regarding the Ramachandran statistics, all structures were refined using standard cryo-EM refinement procedures in Phenix/real-space refinement, and no excessive geometric restraints beyond standard refinement parameters were applied. The high-quality geometry reflects the good resolution and well-defined density of the structures rather than artificial over-restraining.

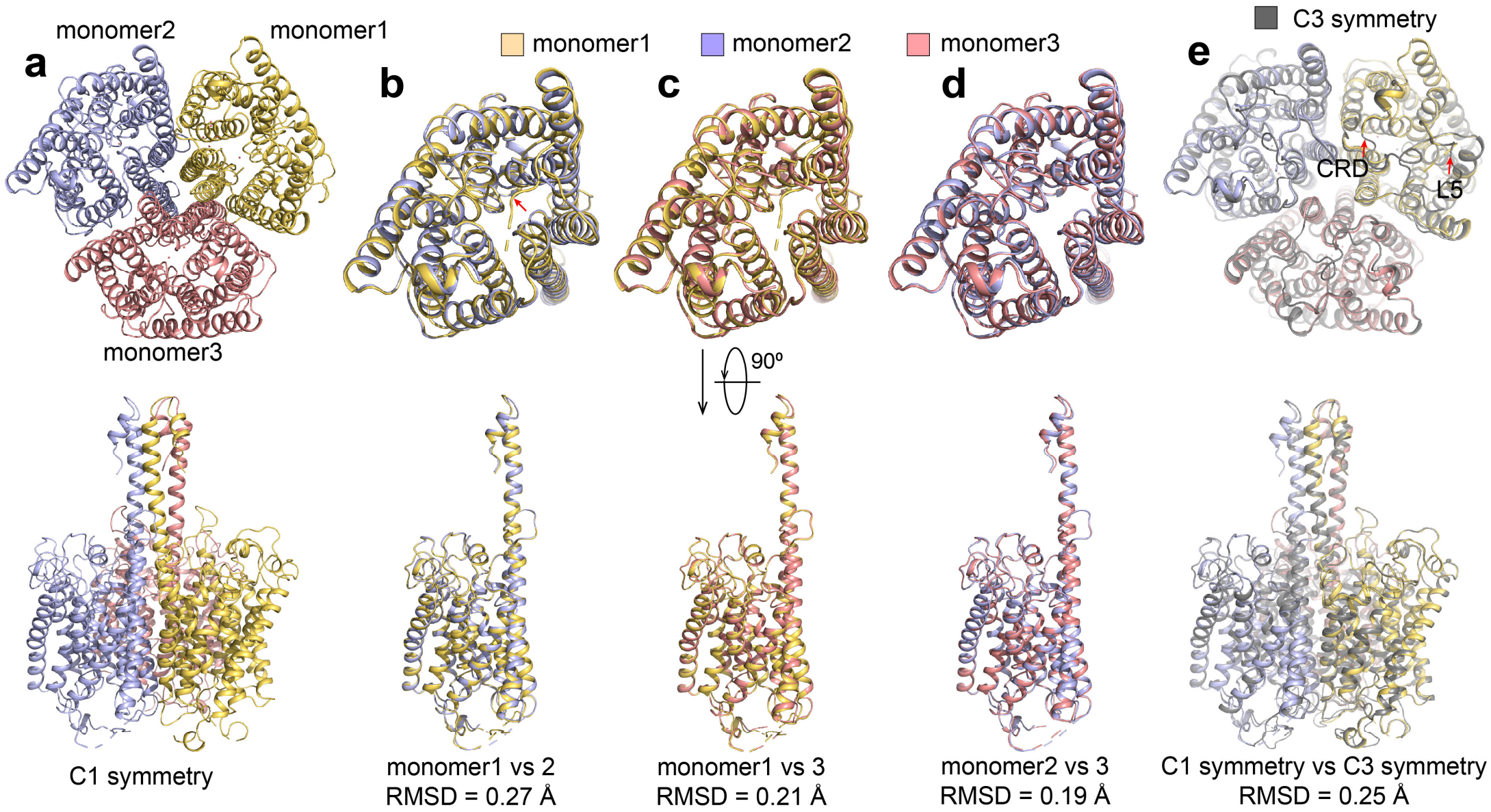

Response Fig. 1 Structural comparison of *At*AMT1;1 protomers derived from C1- and C3-symmetry reconstructions.

a, Atomic model of the *At*AMT1;1 trimer built and refined against the C1-symmetry reconstructed map. The three protomers are individually colored.

b–d, Structural comparison between individual protomers within the C1-derived *At*AMT1;1 trimer. Pairwise superposition of protomer1 versus protomer2 **(b)**, protomer 1 versus protomer3 **(c)**, and protomer2 versus protomer3 **(d)** shows high structural similarity, with differences mainly localized to flexible regions including L5 and the C-terminal regulatory domain.

e, Comparison between the C1-derived model and the C3-symmetry refined model. The two structures are highly similar, with differences primarily observed in flexible peripheral regions, whereas the transmembrane transport core remains nearly identical.

A-STRUCTURAL CONCERNS:

On His372 and the density located below:

If it is NH_4^+ (as the authors propose in line 237), it would make sense to turn the His ring around and let the NE2 atom point to the NH_4^+ volume. In that situation, the NE2 atom would be at ca 3.37 Angstrom (A) from the volume which is NOT modelled by the authors in the 22MG-apo-pH7p4 data and 4.19 A from the modelled "NH_3" (albeit without proper volume, see figure below). In the 22NK- NH_4^+-pH7p4 data, the "NH_3" modelled volume would be at 3.98 A from the NE2 atom of His372 (if turned). Interestingly, this distance is 2.81 A in the 22MV-NH_4^+-pH6 data. This is not adequately described or discussed.

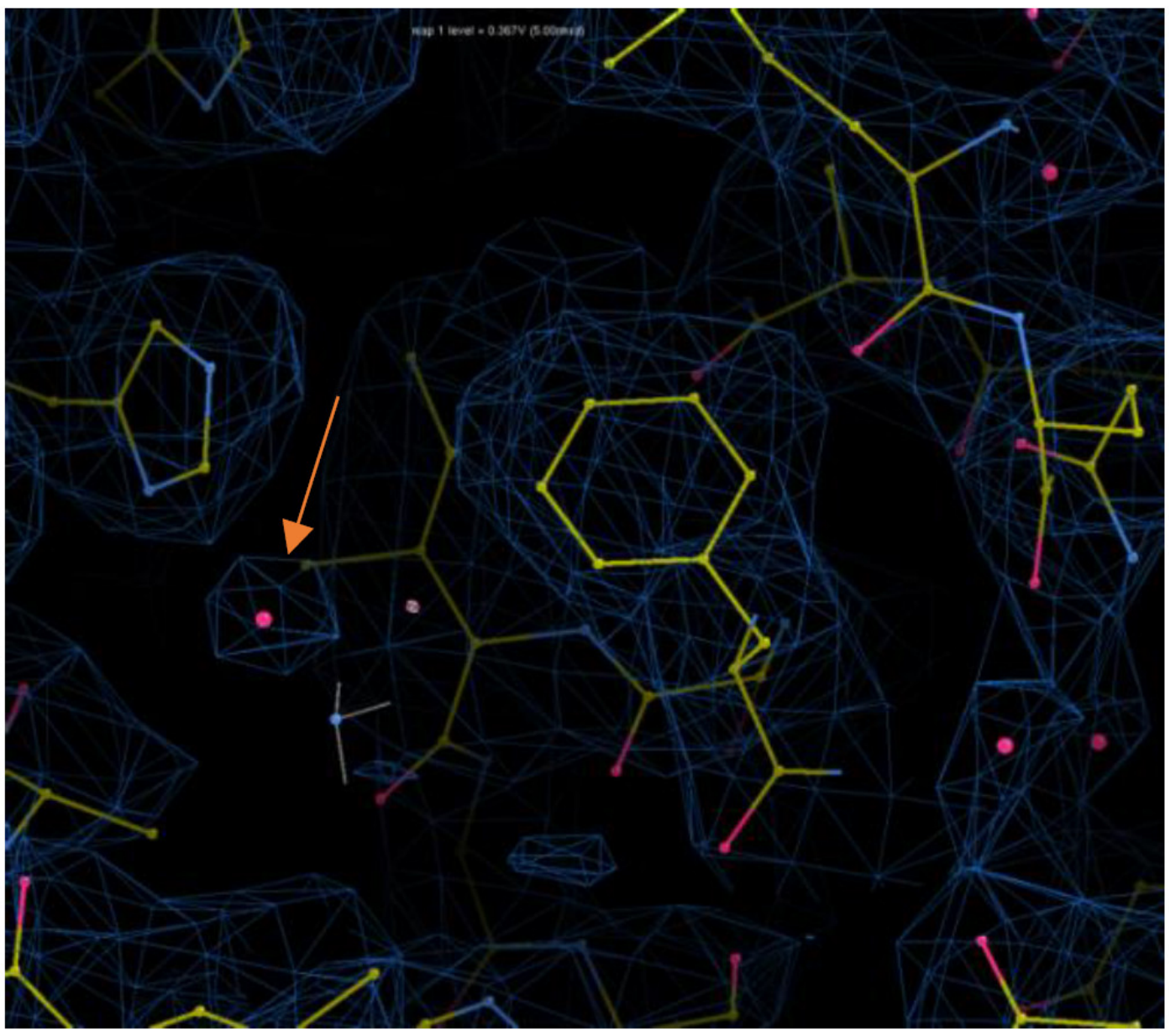

PDB: 22MG EMD-68466

At 5.0 sigma: the modelling of an NH_3 molecule (blue) by the authors has no volume at this sigma contour level, while other volumes - much better defined - were left unmodelled by the authors (here, bellow the second His on the His pair motif a "water" (red) was modelled by the reviewer). To the right, two more volumes (non-modelled by the authors) were modelled by the reviewer, again as "water". These are at the N1/N2 site see below for a better view of the site.

The assignment of MeA in the PDB: 22OY is, in the opinion of this reviewer, not adequate and potential damaging: a) the volume is not very good for the assignment (see figure below) and b) there is no alternative experiment backing up the identification

of MeA in this position:

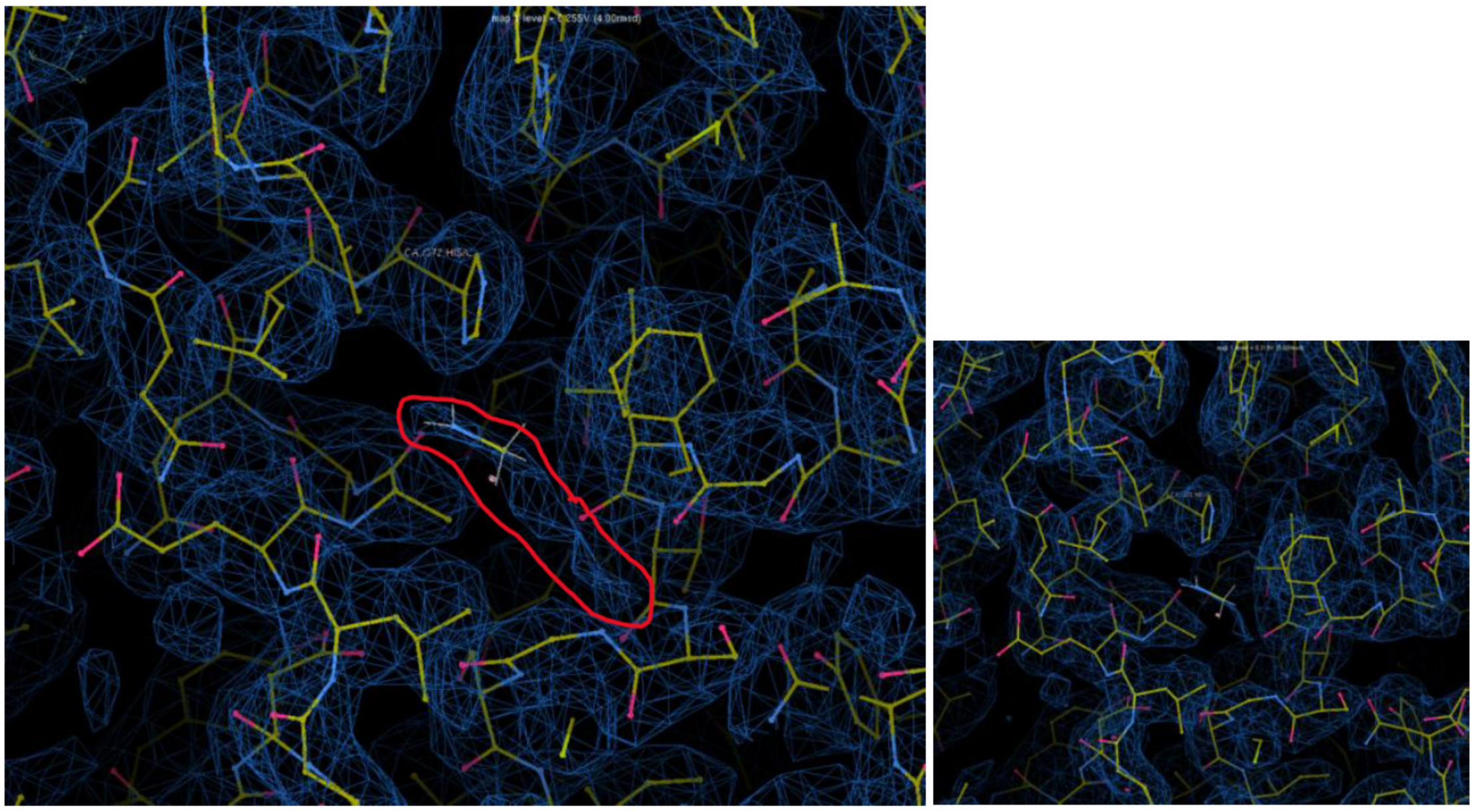

Above, left: MeA was modelled (arrow). Figure is shown at 4 sigma contour. Not only there is more volume highlighted in red, but the volume density is weak and thin, potentially reflecting a weakly associated detergent molecule in this exit vestibule region, as it has been seen in other Amt structures). Above, rigth: same location now at 5.o sigma confirming the very weak and thin volume.

Response: We thank the reviewer for the careful examination of the non-protein densities observed near H372 and W175. We agree that the assignment of these densities requires caution, particularly given the limited resolution and the absence of direct chemical identification.

For the H372 region, we carefully re-examined all cryo-EM maps under different conditions, including apo, With NH_4^+, With MeA, With NH_4^+ at low-pH, H372F, and T460D states. In all structures, the NE2 atom of H372 is oriented toward the observed non-protein density, consistent with a possible interaction between the twin-His motif and a small molecule or solvent molecule. However, considering the reviewer's comment and the limitations of density interpretation at this resolution, we have revised the structural interpretation. Specifically, the previously modeled NH_3/NH_4^+ molecules have been replaced by water molecules, and the corresponding distances between the H372 NE2 atom and these densities are now indicated in the revised **Extended Data Fig. 11**. We have also modified the main text to avoid definitive assignment of this

density as ammonium/ammonia and instead describe it as a substrate-associated density near the twin-His motif **(Lines 254-266 and 273-276)**.

Extended Data Fig. 11, a-f:

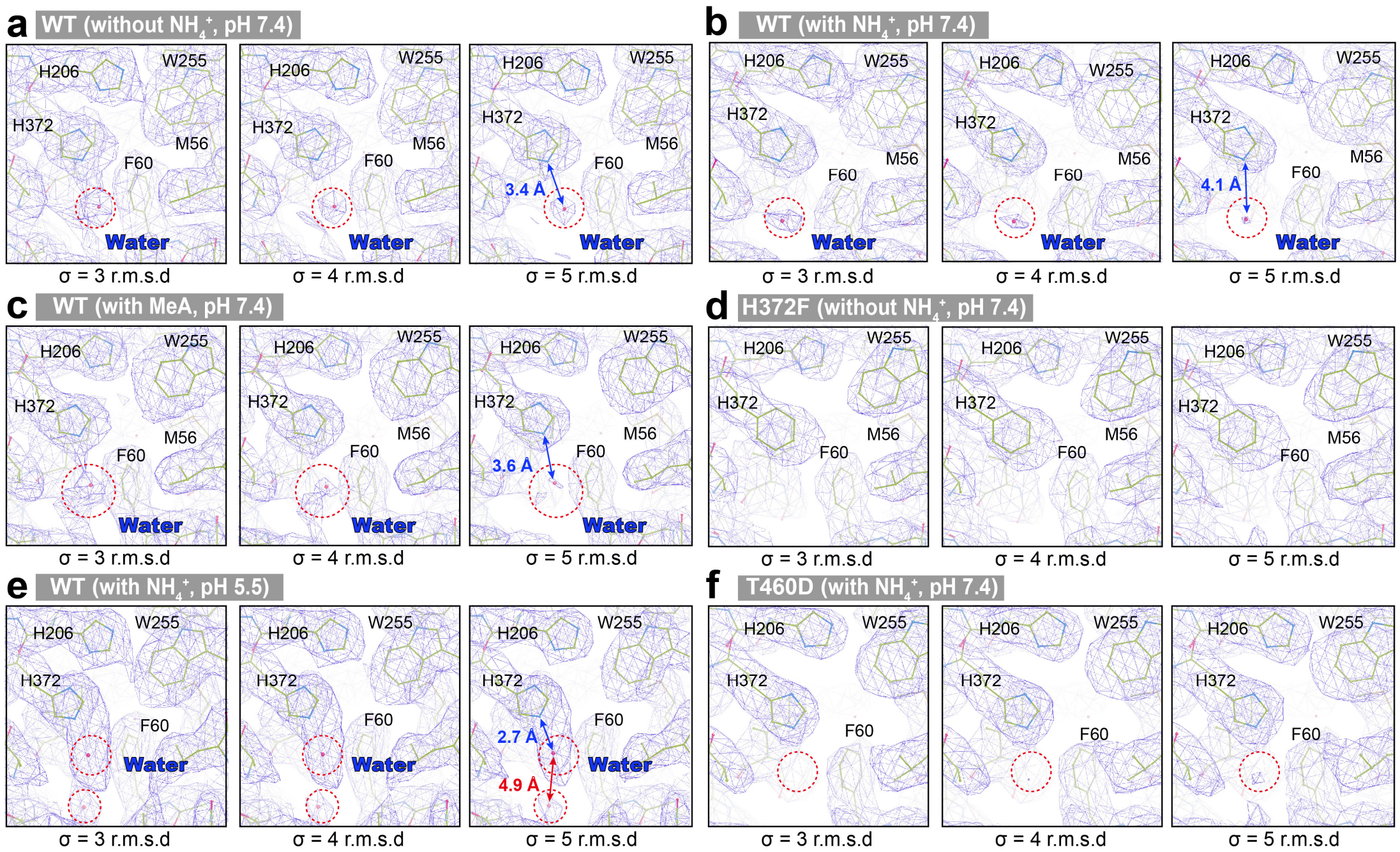

For the W175 region, we agree that assigning the observed density as ammonium or methylammonium was insufficiently supported. Although this density is located at the extracellular vestibule, direct identification of its chemical identity is not possible from the current cryo-EM data alone. Therefore, we have removed the assignment of NH_4^+ and MeA from this region, revised **Extended Data Fig. 12** to show these densities without chemical interpretation, and modified the text accordingly **(Lines 285-295)**. We now describe this region as a potential substrate-recruitment vestibule rather than a directly visualized ammonium-binding site.

These revisions have been incorporated throughout the manuscript, including the corresponding figure panels **(Fig. 4, Extended Data Fig. 11 and Extended Data Fig. 12)**, legends, and text descriptions. We believe these changes provide a more accurate and conservative interpretation of the cryo-EM densities while preserving the structural insights into the substrate conduction pathway.

Extended Data Fig. 12, a-f:

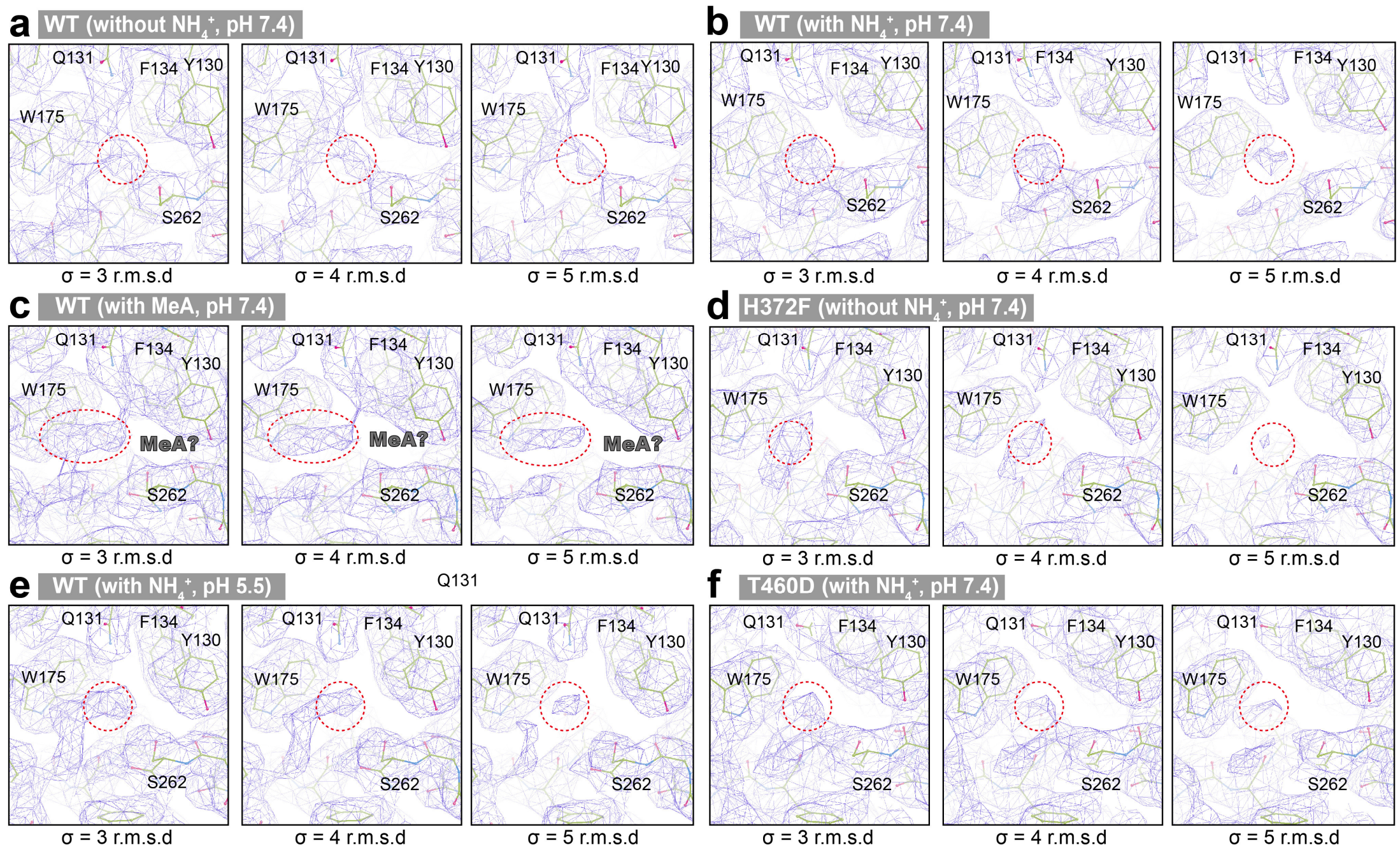

<u>On N1/N2 site and unassigned volumes there:</u>

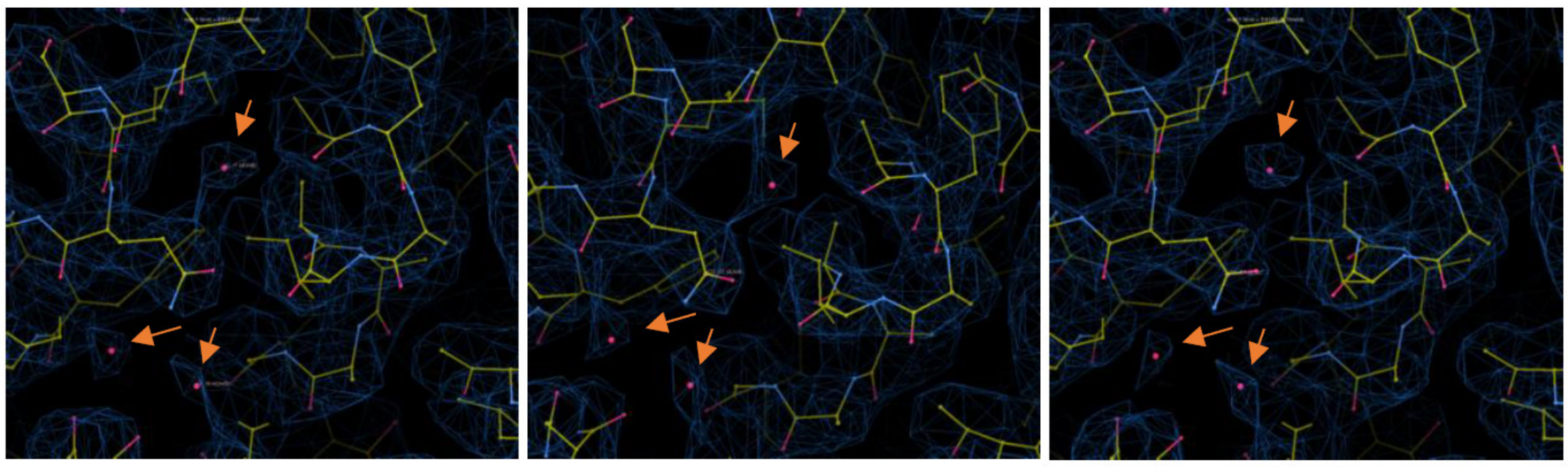

Chain A chain B chain C

In all these 3 pictures above, the N1/N2 site has three unmodelled densities. Arrows show where the reviewer modelled “water” (orange arrow). The sigma contour is 5.7 for all.

The authors have made no effort to model any non-protein volume, except at the recruitment site (an ammonium although this is pure imagination given the experimental volumes there) and below the second twin His (an ammonia – improperly modelled as seen above).

PDB: 22NK
EMD-68512

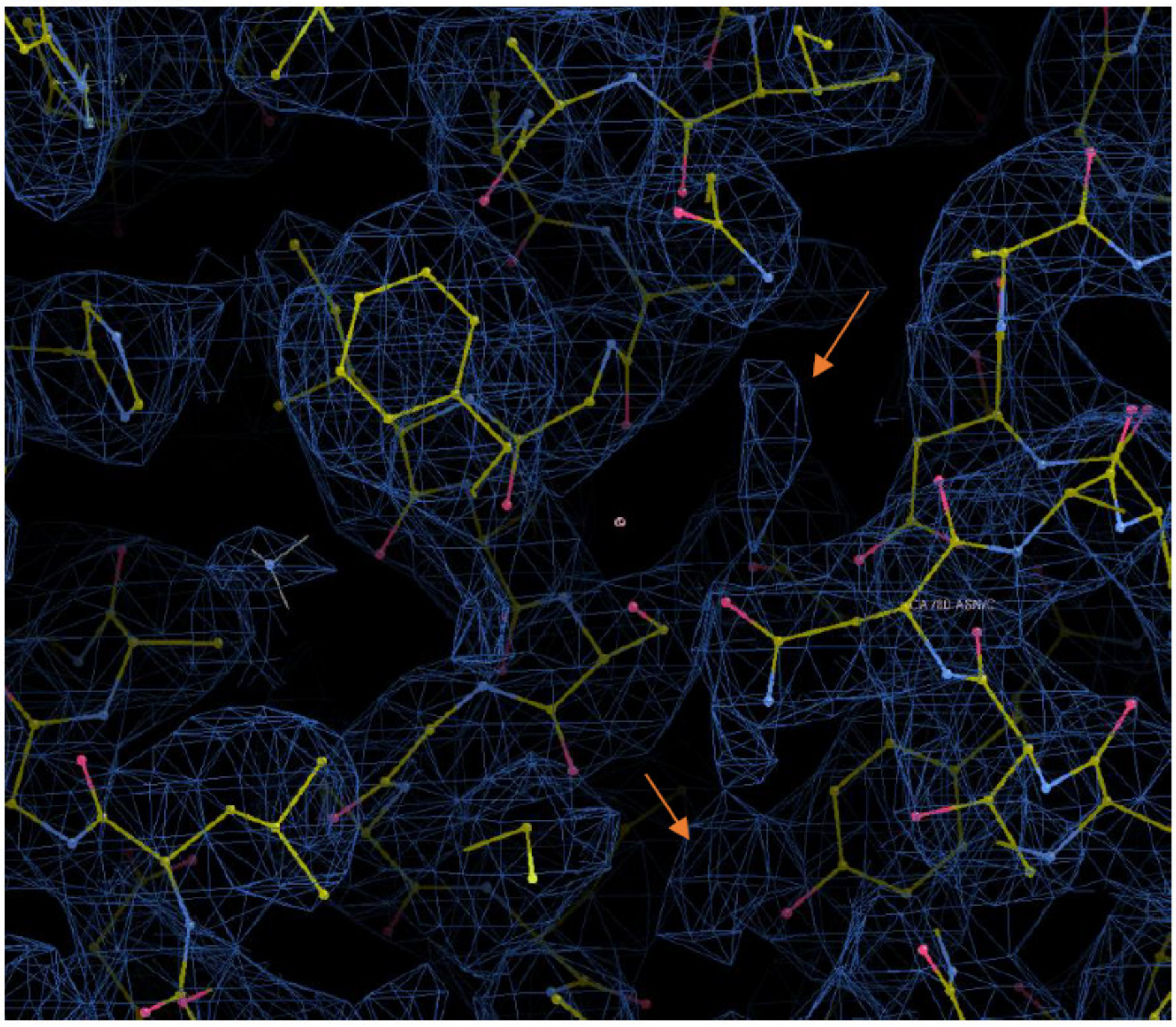

At 3.9 sigma, some unassigned volumes are shown by orange arrows.

Comparison of PDB 22NK (above) to the PDB 22MG (below, in identical position): green arrows show the “waters” modelled by the reviewer below in unassigned volumes:

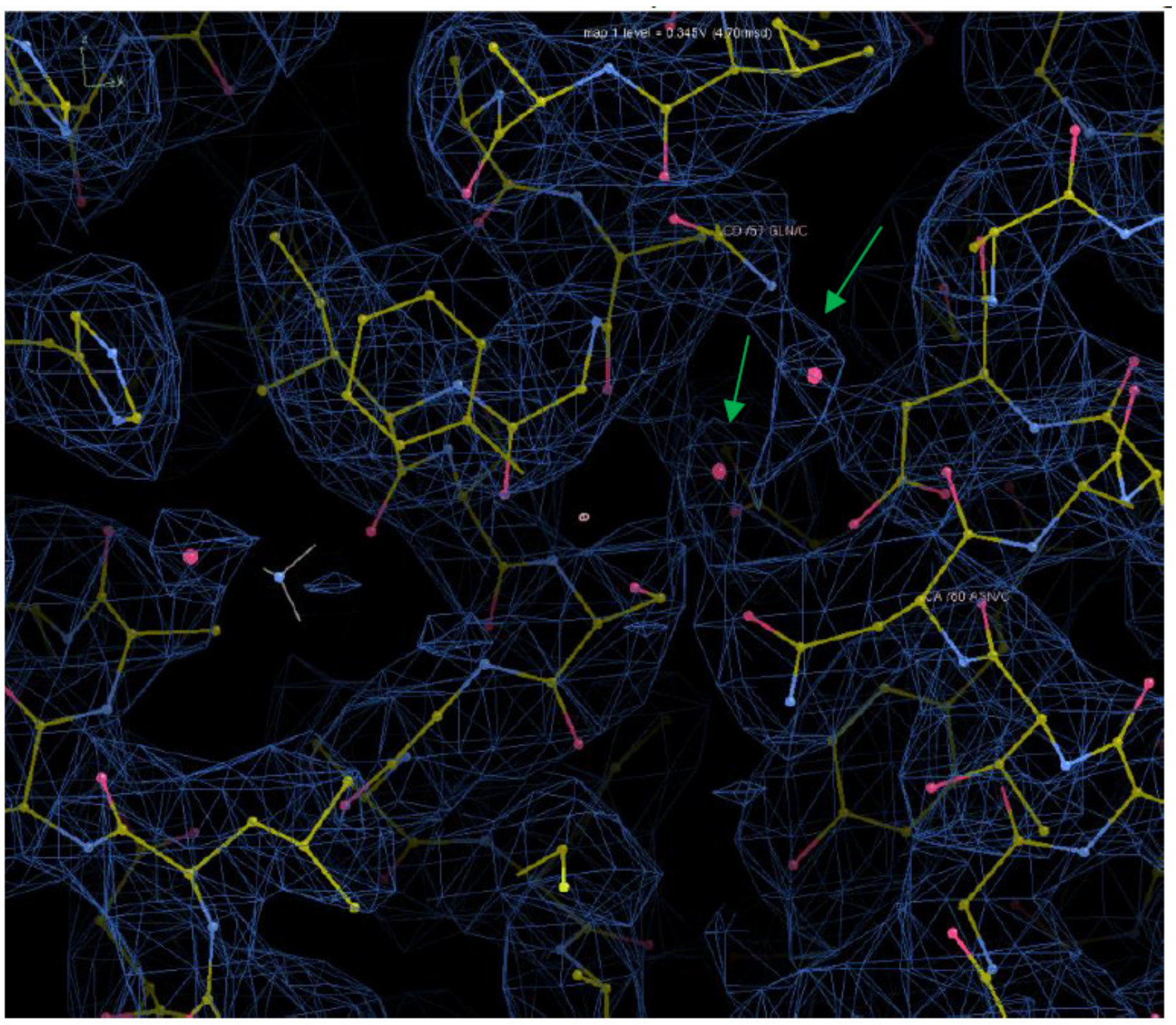

Clearly interesting to compare situations, conformations of again, unassigned volumes

near this region of a potential ammonium binding site.

Another example of some densities left unassigned between PDB 22MG (top), 22NK (middle) and 22MV (bottom), around Gln57 (near N1/N2 site) with potential functional significance. Quality of the maps vary but at least two significant volumes can be seen (arrow):

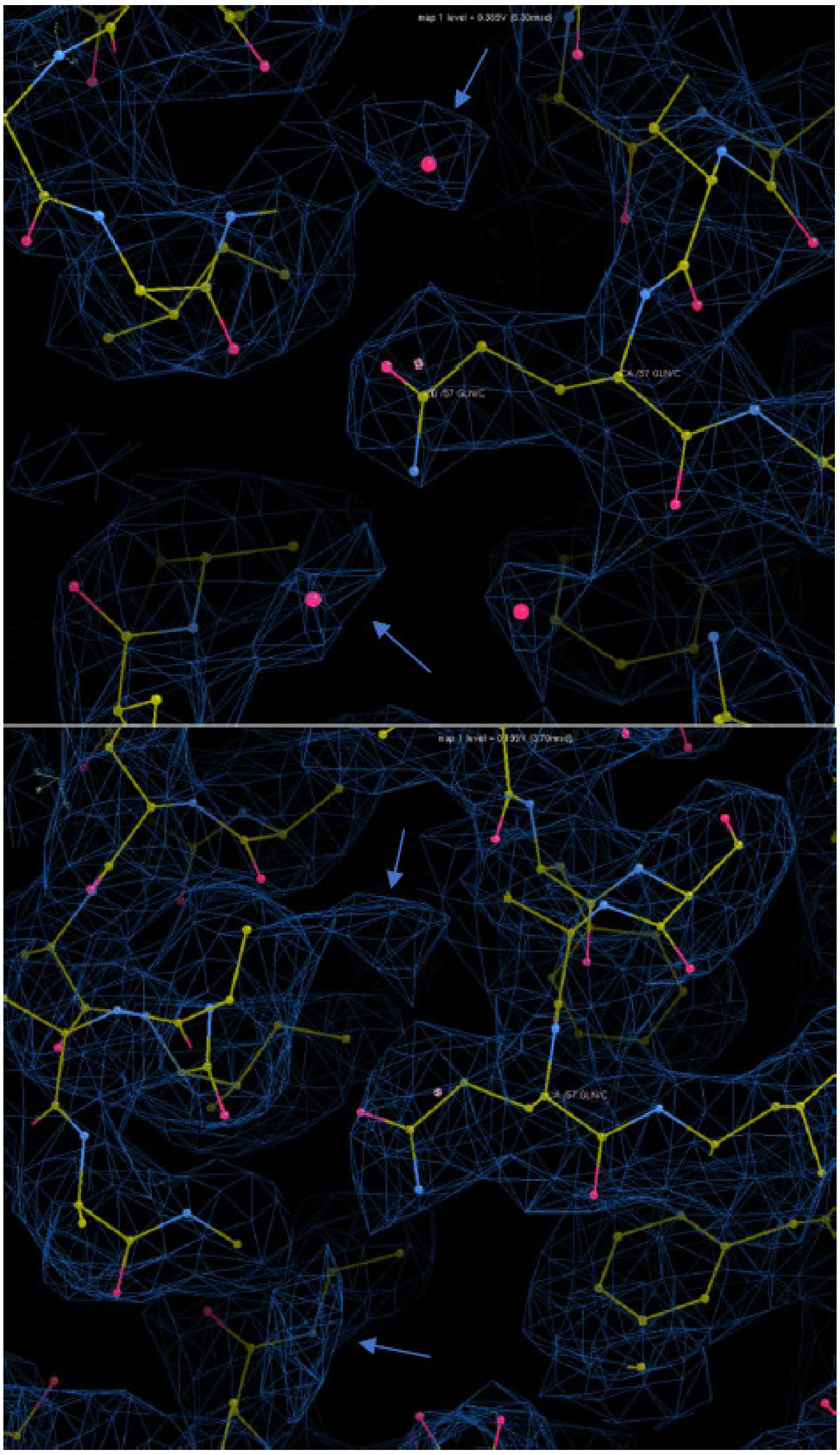

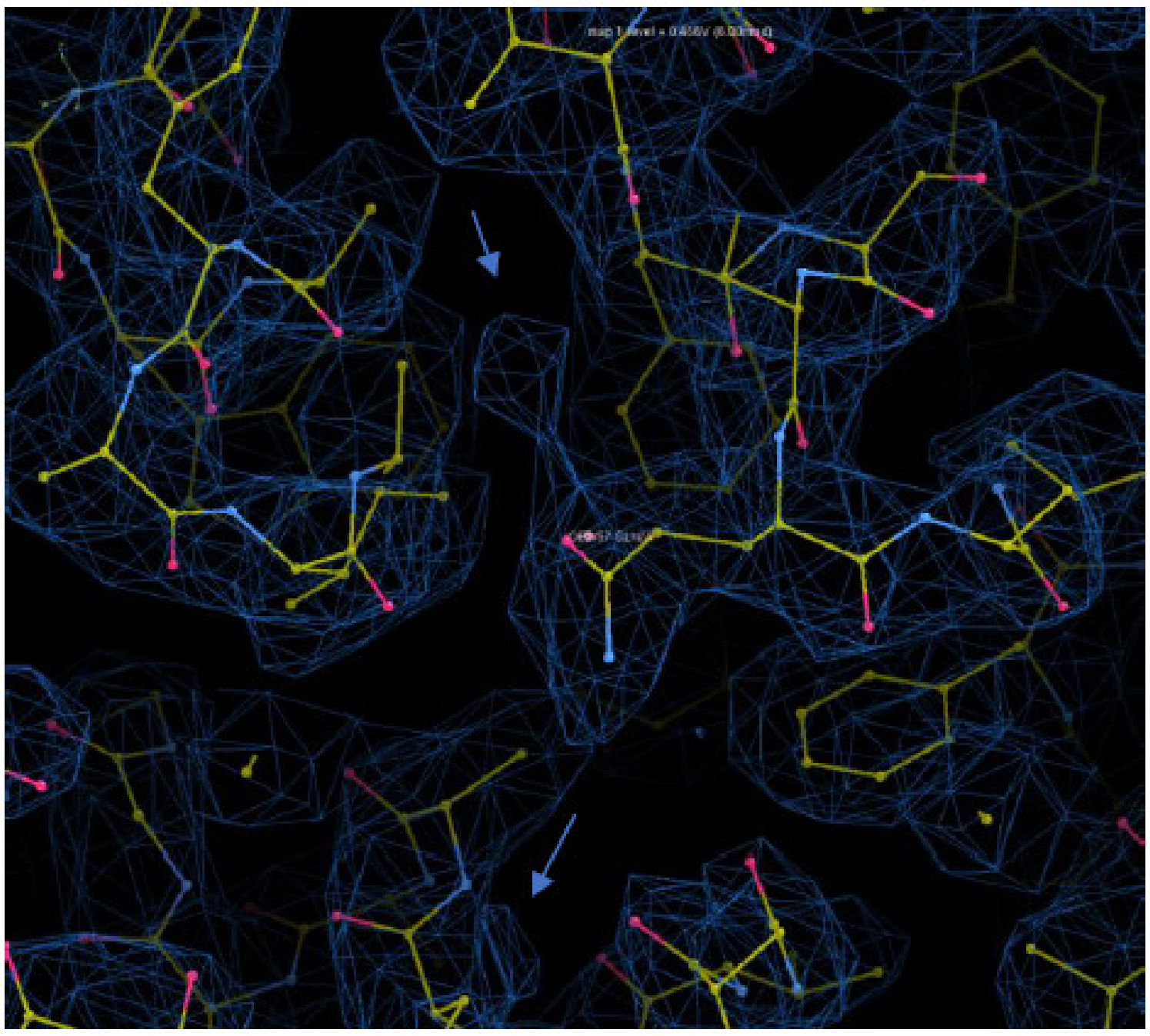

Only one chain is shown, but similar volumes are seen in all chains.

To this reviewer, *At*Amt1;1 residues: Q57, C64, N80, D83, A84, G1540 and S143 should be thoroughly analyzed as very likely N1/N2 ammonium ligands based on recent literature dealing with sequence analysis. Here, the structures show clear volumes in positions that have previously been assigned to water or ammonium in ammonium receptors. They are however, left unassigned and unanalyzed by the authors, which stands as a lost opportunity. The authors claim that they do not show conformational changes, but this is not the decisive point to disclaim this important point. The authors should analyze and describe the site properly. Further investigations may address the transceptors question further, but structurally, the data description is essentially absent and this is not acceptable.

Response: In response to this comment, we re-examined the cryo-EM density maps of the N1/N2-equivalent region across all structures determined under different ligand and regulatory conditions. We now explicitly show the corresponding density maps in **Extended Data Fig. 16 (Also shown below)**. Recurring non-protein densities are indeed observed around Q57, N80 and D83 in multiple structural states. Where the density was sufficiently resolved, we conservatively modeled these features as ordered water molecules. We also examined the corresponding region in the H372F structure,

in which no comparable additional density was apparent. Importantly, however, the occurrence of these densities does not show a consistent correlation with ammonium or methylammonium binding. The corresponding features are present in both ligand-bound and ligand-free structures, and we do not observe an obvious rearrangement of these densities between the different structural states. This behavior differs from the distinct ligand-associated densities and conformational changes reported for the N1 and N2 sites of *Sd*-Amt1. We therefore agree that these densities warrant description, but we consider their assignment as ammonium or methylammonium to be premature based on the current density maps alone.

We further tested the functional relevance of the conserved residues surrounding this region by mutagenesis and yeast complementation assays. Substitution of Q57, N80, or D83 substantially impaired *At*AMT1;1-mediated ammonium transport, whereas C64, S143 and Y155 substitutions had little or no apparent effect under the same conditions **(Now Fig. 8g and also shown below)**. These results provide functional evidence that several residues within the N1/N2-equivalent region contribute to ammonium transport, while also indicating that not all conserved residues in this region make equivalent contributions. We have therefore revised the manuscript to provide a more complete structural and functional analysis of this region **(Lines 506-543)**. Specifically, we now show the relevant cryo-EM densities across the different structural states **(Extended Data Fig. 16)**, model ordered waters where justified by the map quality **(Extended Data Fig. 16)**, and include mutational analysis of conserved residues surrounding the N1/N2-equivalent region **(Fig. 8g)**. We have also revised the text to avoid leaving these densities undescribed or assigning them to ammonium without sufficient structural evidence.

These additional analyses support the presence of a conserved and functionally important region corresponding to the N1/N2 sites of *Sd*-Amt1. However, because the observed densities do not exhibit a consistent ligand-dependent behavior, the present structures do not by themselves establish that these features represent ammonium-binding sites equivalent to the N1/N2 sites of *Sd*-Amt1 or that they mediate transceptor signaling.

Revised Fig. 8:

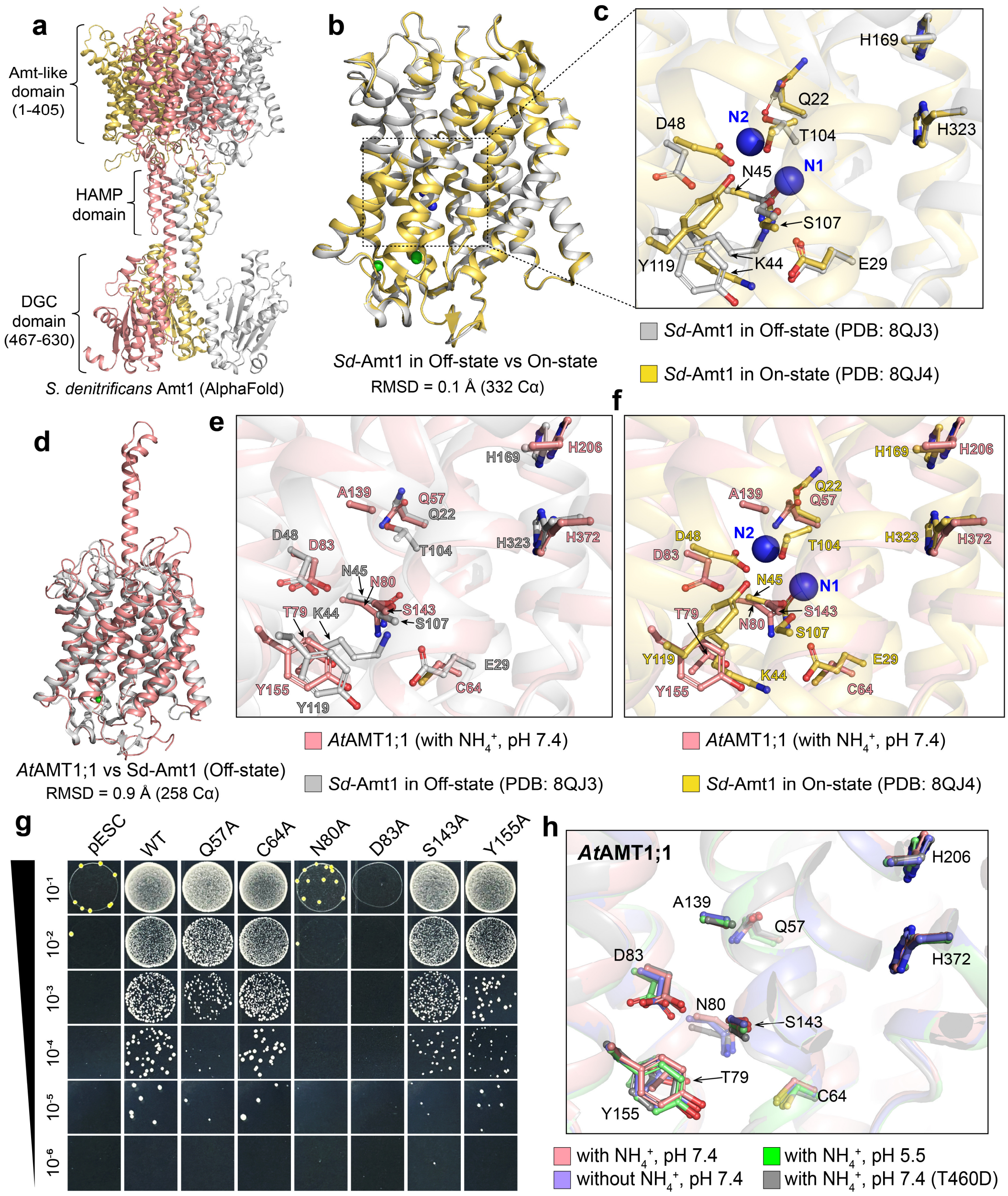

Revised Extended Data Fig. 16:

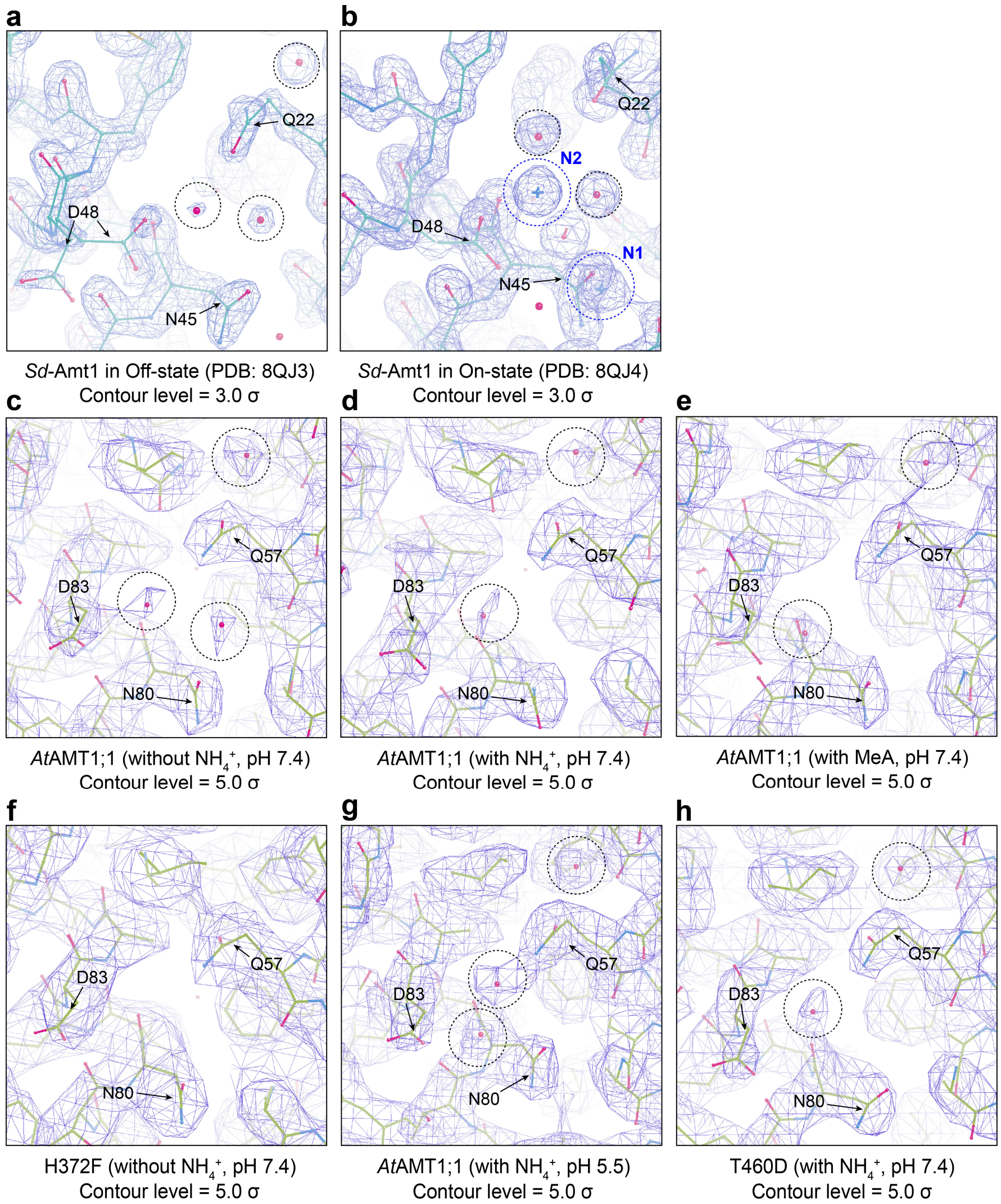

General structural issues:

This reviewer notes a worrisome feature in all structures; Besides the fact that there was absolutely no attempt to model any volume resembling water as water, all monomers in the trimeric models are rigid, appearing in the same exact copied position in the trimer, despite visible changes occur in the volumes for the different monomers in the structure. Were the monomers copied and not further modelled? The authors describe using Phoenix in the materials and methods, but it seems that refinement was not done? How can the observations of static monomers be explained?

Response: We thank the reviewer for raising this important concern regarding the model building and refinement procedure. We agree that careful evaluation of individual protomers is essential for accurately interpreting cryo-EM structures of oligomeric membrane proteins.

We have carefully re-examined all cryo-EM maps and atomic models and have revised the structural analyses accordingly. In particular, we inspected the densities corresponding to non-protein regions throughout the structures and incorporated ordered water molecules into several well-defined densities, especially those located near substrate conduction and recognition regions, where the local density and hydrogen-bonding environment supported reliable assignment. Other ambiguous densities were left unassigned to avoid overinterpretation.

Regarding the apparent similarity among the three protomers, we would like to clarify that the atomic models were not generated by simply copying one protomer to form the trimer. The initial atomic model was independently built into the C1-symmetry reconstructed map and refined using Phenix real-space refinement. Each protomer was individually inspected and adjusted according to its corresponding density. We have now added the following clarification to the Methods section:

"The initial atomic model was built into the C1-symmetry reconstructed map and refined using Phenix real-space refinement. Each protomer was individually inspected and adjusted according to the corresponding density. The final structural analyses were performed using the C3-symmetry refined model because of its improved map quality in the core transporter region."

Our additional analysis of the C1-refined model showed that the three protomers exhibit highly similar overall architectures, with structural variations mainly localized to flexible regions, including the intracellular L5 loop and C-terminal regulatory domain, where local density quality differs among protomers. These observations indicate that the apparent three-fold symmetry reflects the intrinsic structural organization of the *At*AMT1;1 trimer rather than an artifact caused by model duplication **(Shown as below Response Fig. 1)**. Furthermore, comparison between the C1- and C3-derived models demonstrates that the transmembrane transport core is nearly identical, whereas differences are primarily restricted to flexible peripheral regions. Therefore, we used the C3-refined model for subsequent structural analyses because the improved density quality provides more reliable interpretation of the conserved transport core.

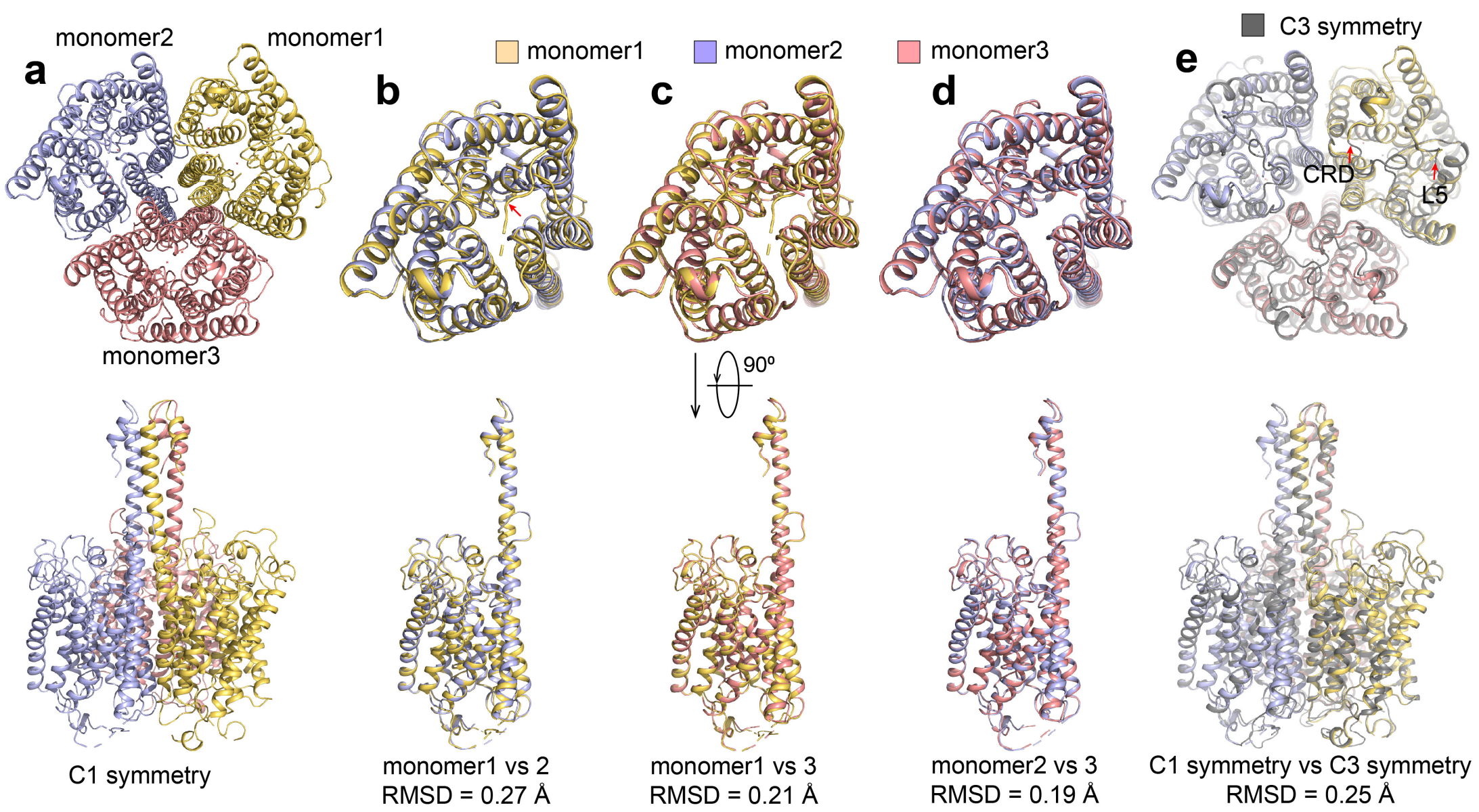

Why is the **PBD: 22OY** (MeA, pH 7.4) a dimer? Why was the trimer not modelled? There is volume for it – as expected since all Amts are obligatory trimers.

The structural work is sloppy, not well done and needs improvements. Please also take care of chain annotations in the PDB files.

Response: We thank the reviewer for pointing out this issue. We agree that AMT proteins form stable trimers, and the *At*AMT1;1 structures presented in this study were determined and analyzed as trimeric assemblies. The dimeric representation of the PDB entry 22OY was not intended and does not reflect the biological assembly of the methylammonium-bound *At*AMT1;1 structure. We suspect that this issue may have resulted from an error during preparation or deposition of the structure files, including the assignment of biological assembly information or chain annotations. We have carefully re-examined all deposited structural files and will correct the PDB entries accordingly to ensure that the complete trimeric assembly is properly represented. We will also carefully check the chain IDs, residue annotations, and biological assembly information for all structures before final submission. We appreciate the reviewer for identifying this issue, which has helped us improve the accuracy and presentation of the structural data.

No PDB or EMD files of the new lower pH dataset were provided for consideration.

Response: We thank the reviewer for pointing this out. The atomic model and EM density map for the low-pH (pH 5.5) dataset were included in the uploaded structural

files during the revision process. However, we apologize if these files were not accessible or properly linked for the reviewer's evaluation.

We have now carefully checked the submission files and will ensure that the PDB coordinate file and corresponding EMDB map entry for the pH 5.5 dataset are correctly provided and clearly indicated in the revised submission. We will also verify that all newly determined structures, including the low-pH dataset, contain the correct metadata, chain annotations, and associated EM density information.

On the recruitment site volumes:

The figure showing NH_4^+/H_2O or MeA at the recruitment vestibule is misleading (Ext-Fig11). The volume is more complex and does not fit such molecules. The sigma level of the maps is not shown in the figures or legends but (see below), even at 6 sigma, the volumes (surrounded in red) do not match the model.

The figures below show the PDB: 22MG (top) and PDB:22OY (bottom), at different sigma contour levels (written on top of the figure as sigma 4 on the left, 5 in the middle and 6 on the right for the top case of 22MG):

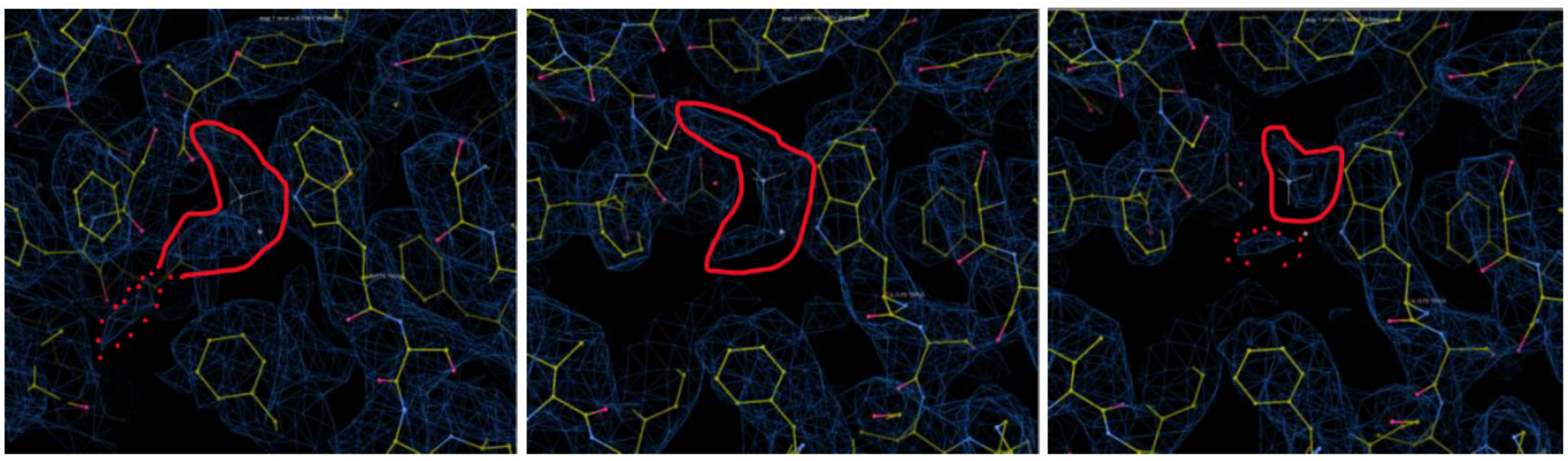

In both (top and down) cases, the view is from outside looking into the recruitment side with W157 located on the right side of the red marks.

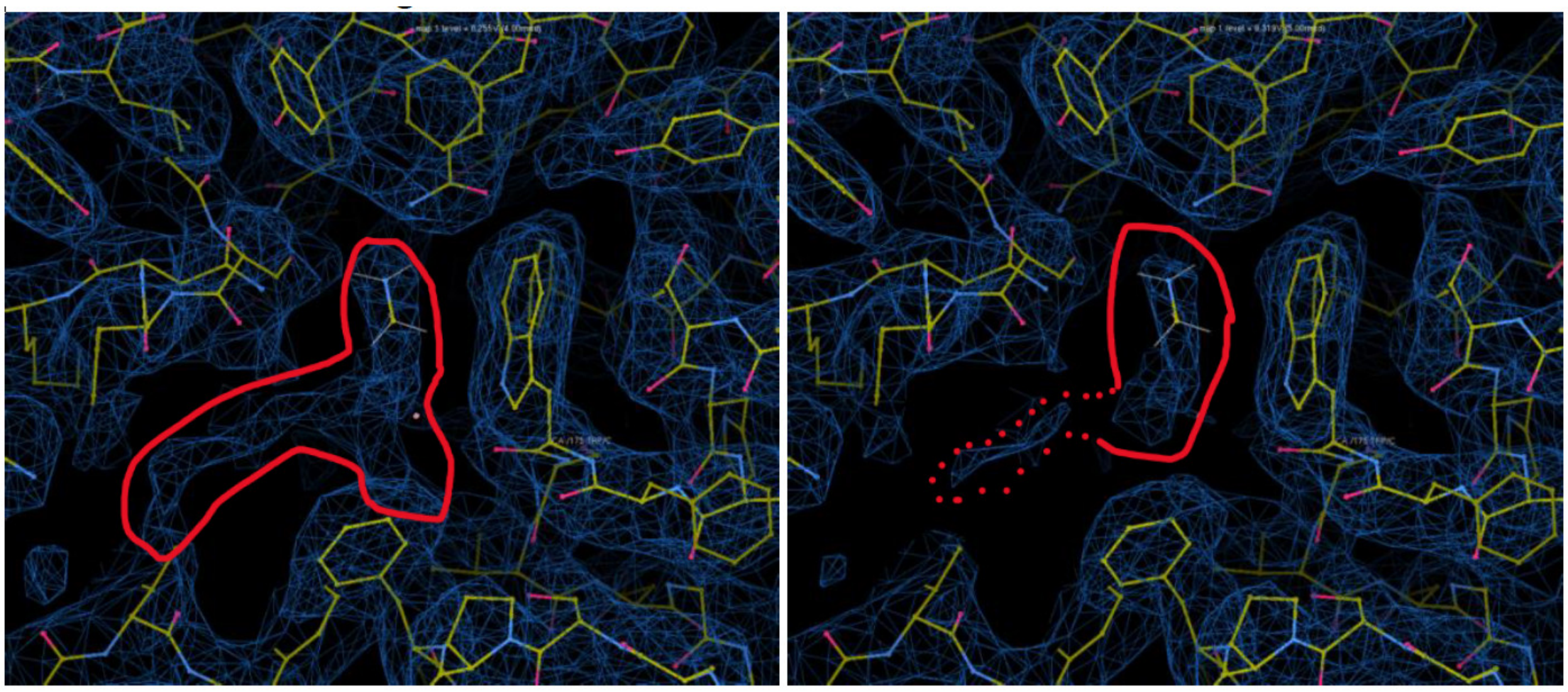

Clearly, the volumes do not correspond neither to MeA nor something as simple and round as H_2O, NH_3 or NH_4^+ and this cannot be written in the text.

Response: We thank the reviewer for carefully examining the non-protein densities observed at the extracellular recruitment vestibule. We agree that the previous assignment of these densities as NH_4^+, H_2O, or methylammonium was not sufficiently supported by the current cryo-EM data, particularly given the complex shape of the densities and the limitation of chemical identification at the achieved resolution.

Following the reviewer's suggestion, we have revised **Extended Data Fig. 12** and removed the specific assignment of these densities. The corresponding densities are now presented without chemical interpretation, and the contour levels of the cryo-EM maps have been explicitly indicated in the figure and legend. In the revised manuscript, we now describe these features as non-protein densities located within the extracellular aromatic vestibule rather than as directly visualized substrate molecules.

We have also revised the main text accordingly **(Lines 285-295)**. The updated interpretation focuses on the role of the conserved aromatic vestibule, including W175 and surrounding residues, as a potential substrate recruitment environment, while acknowledging that the precise identity of the associated densities cannot be determined from the current structural data alone.

The new low pH dataset:

Given that the two Phe have their aromatic rings staked, it is not straightforwardly understandable what the authors mean by " the Phe-gate was slightly narrowed"? What does the site look like if the low pH structure is superimposed with any other? Such

situation has not been described before and a detailed view showing that on Fig 5 would be very adequate.

Response: We thank the reviewer for this insightful comment. We agree that the previous description of a “slightly narrowed Phe-gate” based solely on the pore-radius analysis was insufficient to explain the underlying structural basis. Therefore, we have revised **Fig. 5c** to include a direct structural comparison between the neutral pH and low-pH structures.

Structural superposition revealed that the overall architecture of the ammonium conduction pathway remains highly conserved between the two pH conditions. However, a subtle local rearrangement occurs at the Phe-gate region under acidic conditions. Specifically, the distance between F258 within the Phe-gate and the conserved H206 residue of the adjacent twin-his motif is reduced from 4.7 Å at pH 7.4 to 4.4 Å at pH 5.5, indicating a slight local constriction of the gate region. Importantly, the aromatic rings of the two Phe residues remain stacked, and the observed change does not represent a large rearrangement of the Phe-gate but rather a subtle repositioning of surrounding residues that alters the local pore environment.

We have revised the corresponding description in the main text **(Lines 375-378)** and now state that low pH induces a subtle local rearrangement, leading to a modest reduction in the distance between F258 and H206, accompanied by widening of the intracellular exit region **(Fig. 5c and also shown below)**.

Revised Fig. 5c:

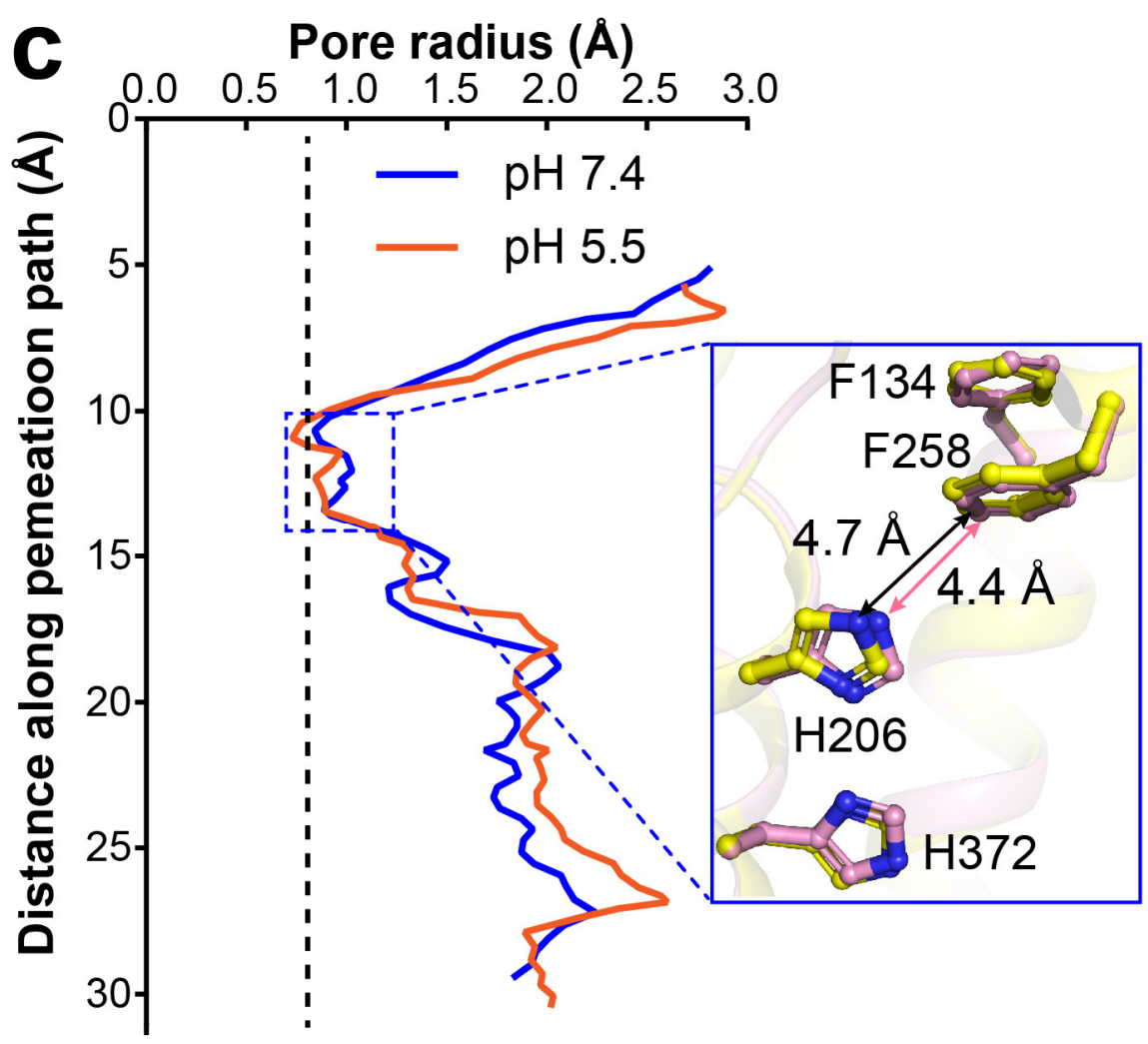

B - MASS PHOTOMETRY DATA:

Line 175 and ext-Fig 7b, c: the MP interpretation data remains debatable: This reviewer finds it unclear why the authors assign the large MW peak to a trimer instead of a hexamer – this MP data should be taken together with size exclusion chromatography results of pure protein in detergent micelles). Moreover, micelles (from detergent-only or from protein in detergent suspensions) will reveal background contributions that depend on the concentration of detergent used and will produce symmetric profiles in the negative x axis scale, while the same is not true for protein contributions. Can the authors verify this?

Response: We thank the reviewer for this insightful comment regarding the interpretation of our mass photometry (MP) data. We agree that the oligomeric state assignment from MP measurements should not be interpreted independently and should be considered together with complementary biochemical and structural analyses. Therefore, we have revised the manuscript to clarify that MP provides evidence for a stable oligomeric population of *At*AMT1;1, whereas the trimeric architecture is established by SEC analysis and cryo-EM reconstruction (Shown below in **Response Fig. 2**).

To further address the potential contribution of detergent micelles, we performed additional analysis of the complete positive and negative mass photometry signals. Mass photometry detects individual particles based on changes in interferometric scattering signals upon their interaction with the glass surface. Particle landing events generate positive signals, whereas dissociation events generate negative signals. Because detergent micelles undergo rapid and reversible association/dissociation with the surface, they typically produce symmetric positive and negative peaks. In contrast, stable protein particles generally exhibit predominantly positive signals due to their longer-lived surface association.

Importantly, the final LMNG concentration used for purified *At*AMT1;1 samples was identical to that used in detergent-only controls. As shown in the **revised Extended Data Fig. 7**, LMNG detergent alone generated a symmetric peak distribution with comparable positive and negative signals (−213 ± 41 kDa and 213 ± 41 kDa). Similarly,

the first peak observed in WT *At*AMT1;1 samples displayed a symmetric distribution (−157 ± 32 kDa and 165 ± 41 kDa), closely matching the detergent micelle signal, indicating that this population represents detergent-associated background.

In contrast, the second major population in WT *At*AMT1;1 samples (~382 ± 79 kDa) lacked a corresponding negative peak, suggesting that this signal originates from a stable protein-associated particle rather than detergent micelles. The molecular mass of this population is consistent with a detergent-stabilized *At*AMT1;1 trimer, which is independently supported by the trimeric architecture resolved by cryo-EM and the oligomeric behavior observed by SEC.

Furthermore, the C24S mutant and N-terminal truncation mutants showed reduced or heterogeneous protein-associated populations, supporting the role of the N-terminal disulfide-linked region in maintaining oligomer stability. We have revised the corresponding text and modified our interpretation of the MP results accordingly **(Lines 186-202)**.

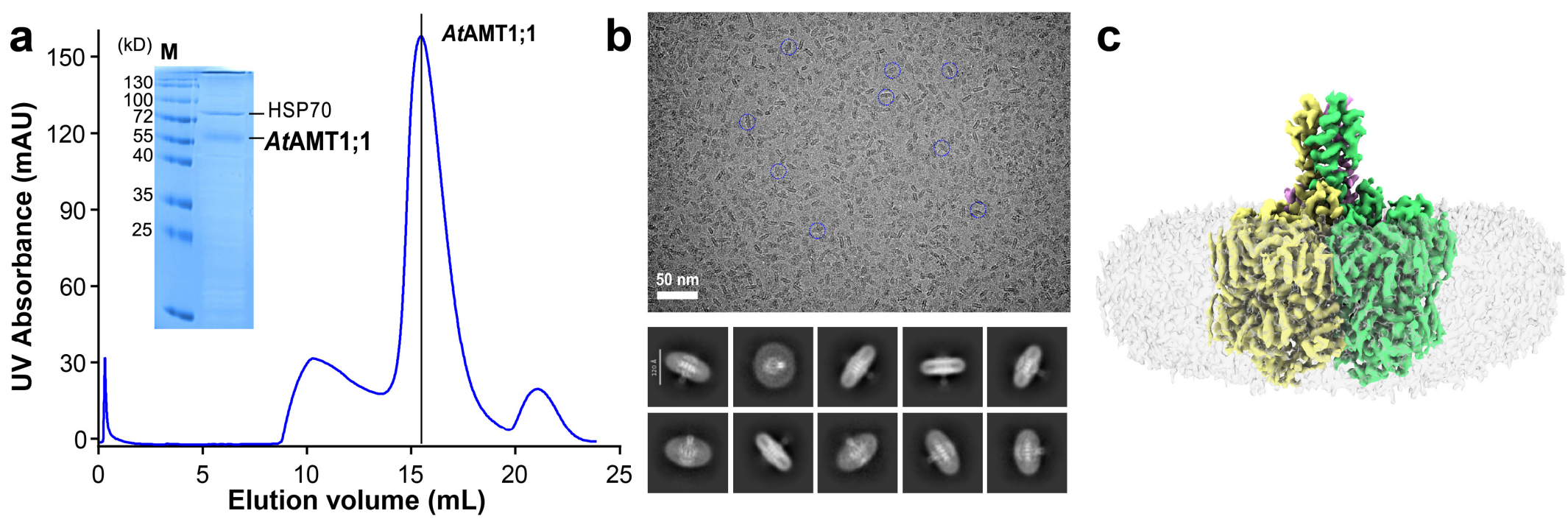

Response Fig. 2 Analysis of detergent micelle contribution and oligomeric state determination by mass photometry.

a, Purification profile and SDS-PAGE analysis of *At*AMT1;1. SEC analysis of purified *At*AMT1;1 in detergent micelles showed a predominant oligomeric peak.

b, Representative cryo-EM micrograph and 2D class averages of *At*AMT1;1.

c, Cryo-EM reconstruction showing the trimeric architecture of *At*AMT1;1.

Revised Extended Data Fig. 7b:

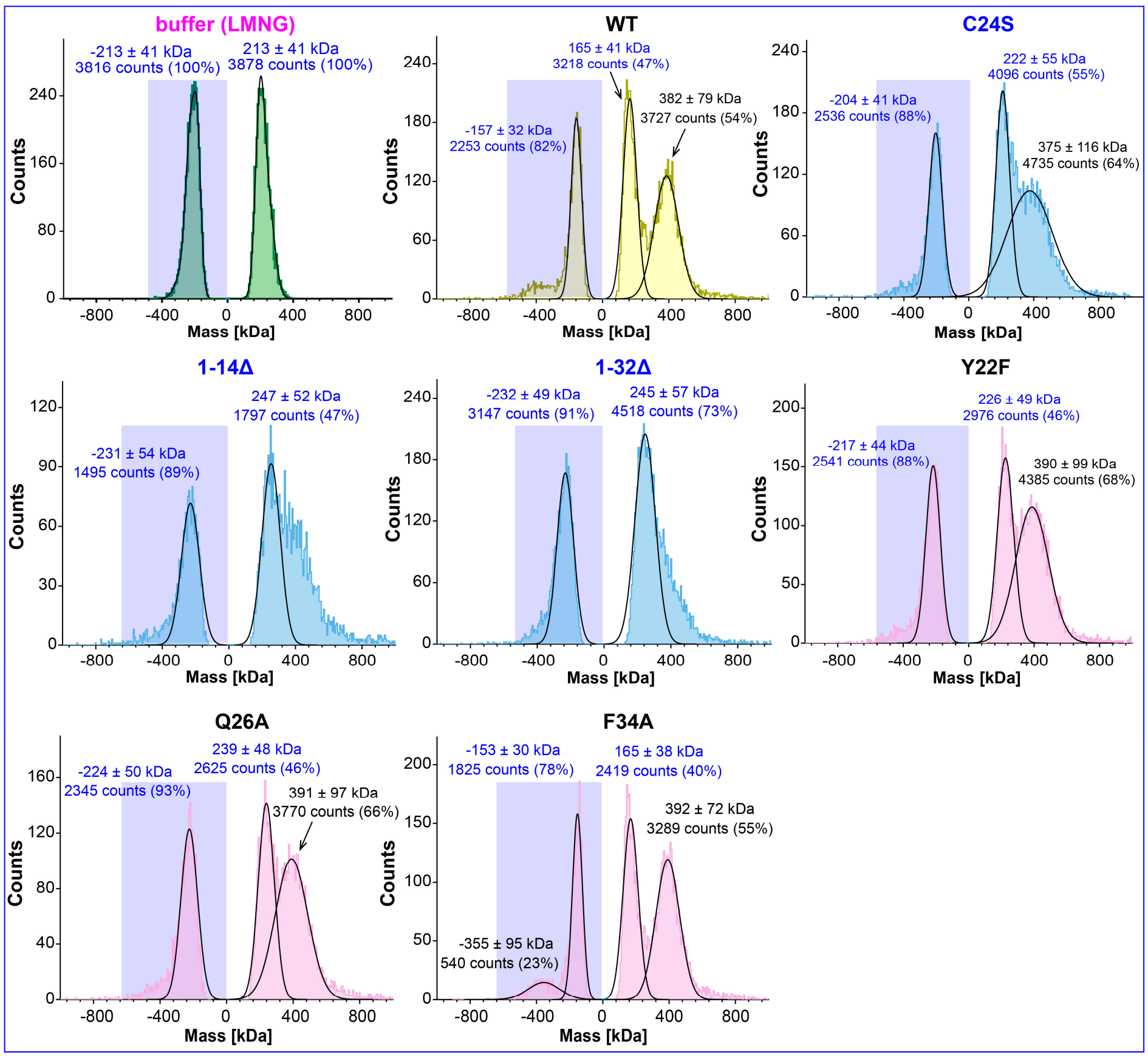

<u>C – N-TERM CYS:</u>

In the experiments done using (i) DDT / (ii) H_2O_2 to (i) reduce the disulfide bond and break the cystine and (ii) re-oxidize the thiols and regenerate the cystine, H_2O_2 is a very poor choice as it is too strong oxidizing. DTT (not 50 or 100 mM but less, 5 – 20 mM can be used at neutral pH) but DTT needs to be removed (desalting or dialysis) before adding (not 30 mM, but 0.1 -1 mM) H_2O_2. If it is not removed, DTT will mostly react with the added H_2O_2 and rapidly deactivate it. Also, H_2O_2 near lipids and aminoacids is well known to be extremely destructive. Therefore, instead of this assay which can produce irreproducible and non-linear results, a glutathione redox buffer system or the use of diamide instead of H_2O_2 would have been a much better choice.

<u>Response: </u>In response to this comment, we redesigned the redox experiments using milder and better-controlled conditions. Specifically, we reduced the concentration of DTT used for disulfide reduction and introduced a desalting step to remove residual DTT before the subsequent oxidation treatment. We also substantially reduced the

concentration of the oxidizing reagent and optimized the treatment conditions to minimize nonspecific oxidation and potential damage to the protein or surrounding lipids.

Using the revised experimental procedure, we repeated the analysis of the effects of disulfide reduction and re-oxidation on *At*AMT1;1. The new results are consistent with our original observation that the redox state of the N-terminal cysteine pair affects the oligomeric behavior of the protein, whereas the 1-32Δ mutant is less sensitive to the redox treatment. These results provide additional support for the involvement of the N-terminal Cys3–Cys24 disulfide bond in maintaining the oligomeric state of *At*AMT1;1. We have replaced the original redox experiments and revised the corresponding Results text **(Lines 211-223)** and figure accordingly **(As now Extended Data Fig. 8 and also shown below)**. We have also removed the previous experimental conditions and clarified the revised procedure in the Methods section. Importantly, we have moderated our interpretation to avoid implying that the experiments directly establish a physiological redox-sensing mechanism. Rather, the revised data support a role for the conserved N-terminal disulfide bond in maintaining the structural and oligomeric properties of *At*AMT1;1.

Revised Extended Data Fig. 8:

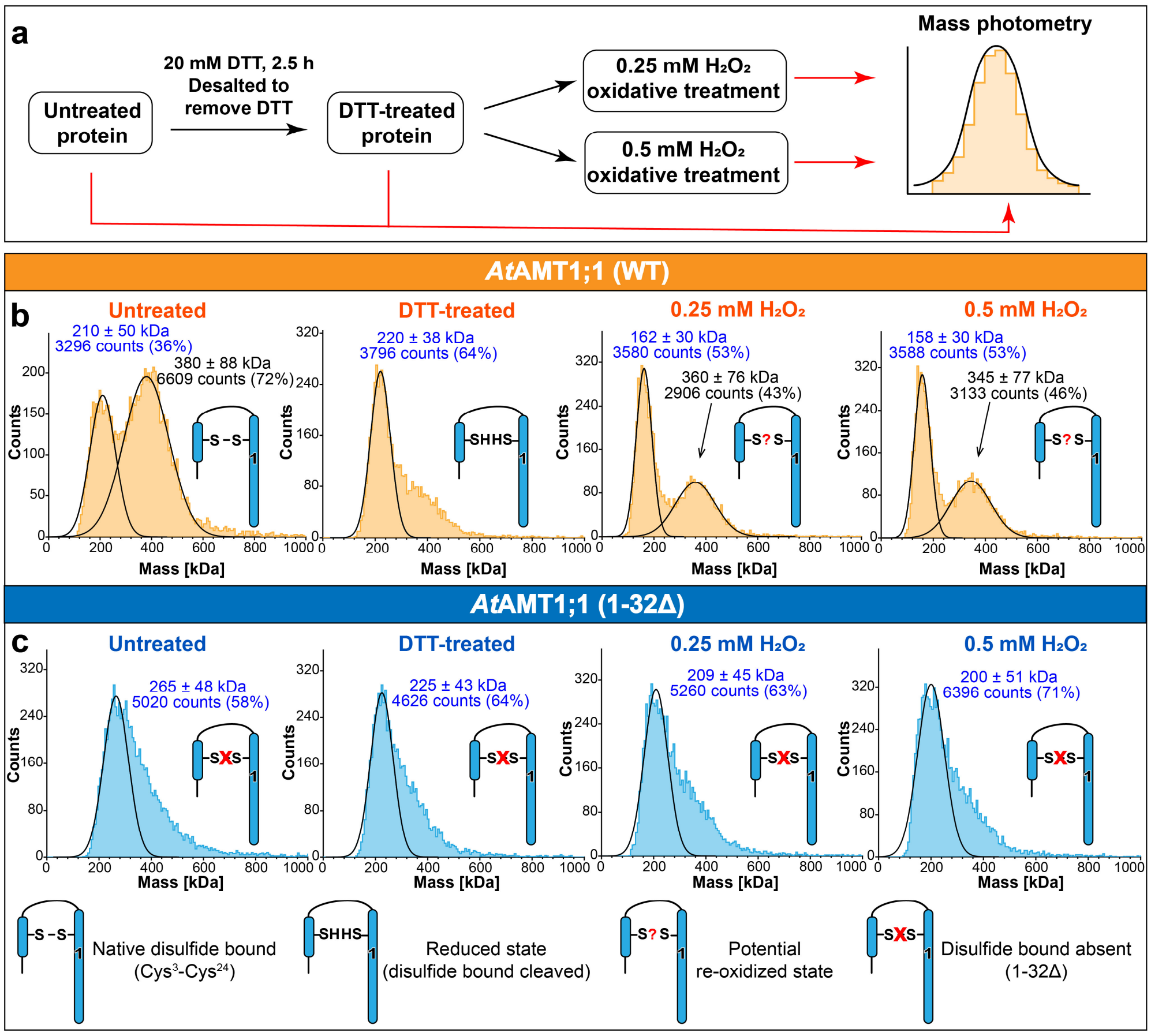

D - OTHER ISSUES:

Please replace "substrate" with "cargo" as this is the correct description for the molecule/ion that a transporter recognizes and mediates transport of, in contrast to a substrate molecule that is catalyzed into a product by an enzyme.

Response: We appreciate the reviewer's suggestion. Although "substrate" has been frequently used in previous ammonium transporter studies to describe transported molecules, we agree that "cargo" more accurately reflects the function of AMT1;1 as a transporter rather than an enzyme. We agree that the term "substrate" may be misleading in the context of membrane transporters, as the transported molecule is recognized and translocated without undergoing enzymatic conversion. Therefore, we have replaced "substrate" with "cargo" throughout the revised manuscript to improve terminological accuracy and consistency.

Line 302: When mentioning computation data on the hypothesis of protonation/deprotonation of the twin-His motif during transport, it is a must to cite Ullmann et al, J Phys Chem B, 2012, 116:9690 or similar publications, which focused

precisely on this issue.

Response: We thank the reviewer for pointing this out. We agree that previous computational studies investigating the protonation/deprotonation behavior of the conserved twin-His motif are highly relevant to the discussion of ammonium transport mechanisms. We have carefully checked the citation and confirmed that the work by Ullmann et al. (J. Phys. Chem. B, 2012, 116:9690) **(as now ref. 23)** has already been included in the revised manuscript. We have further revised the corresponding discussion to better acknowledge previous computational studies on the protonation dynamics of the twin-His motif and to place our structural observations in the context of these proposed transport mechanisms.

Line 394: replace “is” by “are”

Response: We thank the reviewer for pointing this out. We have corrected this grammatical error by replacing “is” with “are” in the revised manuscript **(Line 425)**. We appreciate the reviewer’s careful reading and helpful suggestion.

Line 455: why plural? Which W do the authors refer to here with the plural?

Response: We thank the reviewer for pointing this out. We agree that the use of the plural form “tryptophan residues” was imprecise, as our structural and functional analyses mainly refer to the conserved W175 residue located at the extracellular entrance vestibule of *At*AMT1;1. We have revised this sentence accordingly and replaced “tryptophan residues” with “the conserved tryptophan residue W175” to avoid ambiguity **(Lines 484-487)**.

Line 456: replace “gate” by “vestibule”.

Response: We thank the reviewer for pointing this out. We agree that “vestibule” is more appropriate for describing the extracellular aromatic recruitment region, whereas “gate” should be reserved for specific constriction sites such as the Phe-gate. We have replaced “gate” with “vestibule” in the revised manuscript.

459 and 496: “continuous .. conduction pathway”? The two Phe interrupt the pore opening, it is not exactly continuous – or explain better.

Response: We thank the reviewer for this important comment. We agree that the term “continuous conduction pathway” may be misleading, as the Phe-gate represents a

conserved constriction point within the transport pathway and does not correspond to an uninterrupted open pore.

We have therefore revised the text by replacing "continuous conduction pathway" with "conserved transport pathway" throughout the manuscript to more accurately describe the architecture of the *At*AMT1;1 transport route **(Lines 490-491)**.

Line 484: Wasn´t there a reference from 2011 (ref 48) showing that N-terminal Cys affect oligomer stability in LeAMT1;1? Rephrase accordingly this paragraph.

Response: We thank the reviewer for pointing out this important previous study. We agree that the role of the conserved N-terminal cysteine residues in AMT1 stability and oligomerization was previously suggested in *Le*AMT1;1 **(as now ref. 52)**. Our intention was not to imply that the contribution of the N-terminal cysteine residues to transporter stability was newly discovered in this study. Rather, our structural and biochemical analyses provide direct evidence for the presence of the conserved extracellular disulfide bond and further reveal its potential role in coupling extracellular redox conditions to AMT1 transporter stability and activity.

We have therefore revised this paragraph to better acknowledge the previous findings and clarify the advance provided by our study.

Fig 2, a and b are redundant.

Response: We thank the reviewer for this suggestion. We agree that Fig. 2a and Fig. 2b both provide an overview of the *At*AMT1;1 structure; however, they serve different purposes. Fig. 2a shows the cryo-EM density map of the *At*AMT1;1 trimer, highlighting the overall architecture and the quality of the reconstructed density, whereas Fig. 2b presents the corresponding atomic model to illustrate the structural organization, including the trimeric assembly, transmembrane topology, and the extracellular N-terminal disulfide bond.

We believe that retaining both panels is important because the density map provides direct evidence for the structural determination, while the atomic model allows detailed interpretation of the molecular architecture. To avoid potential confusion, we have revised the figure legend to better clarify the distinct purposes of these two panels **(Lines 680-684)**.

Reviewer #2:

The authors have addressed my previous comments and introduced additional clarification and structural analyses.

Response: We sincerely thank the reviewer for the positive evaluation of our revised manuscript. We appreciate the constructive suggestions provided during the review process, which have greatly helped us improve the clarity and rigor of our study. The additional analyses and clarifications have further strengthened our conclusions.

Reviewer #3:

The revised manuscript has clearly improved in what concerns the overall description of the state-of-the-art in the field of Amt proteins, the correct citation and inclusion of relevant literature describing work done by others and the perception of relevant or actual hypothesis. This was a major problem in the early submission.

The previously well-studied role of the C-terminus phosphorylation in allosteric transport regulation and the N-term potential regulatory sites are important hallmarks in the field of plant Amts. Their structural description can certainly help researchers advancing our understanding of these proteins.

Response: We sincerely thank the reviewer for the positive evaluation of our revised manuscript. We appreciate the reviewer's recognition that the revised version provides a more accurate description of the current understanding of Amt proteins and appropriately incorporates previous studies and relevant hypotheses. We agree that C-terminal phosphorylation and N-terminal regulatory elements are important features of plant AMT regulation. We believe that our structural and functional analyses provide new insights into how these regulatory regions contribute to AMT1;1 regulation. We greatly appreciate the reviewer's constructive comments, which have helped us further improve the quality and clarity of our manuscript.

At the same time, other points remain vague or have not yet been addressed. For example, the role of this protein in sensing. The authors only left a small part of the discussion to the topic. But while they say that the residues required to detect ammonium are present in this Arabidopsis protein, they do not show any picture of the site or describe it any further. Seems like a lost opportunity not to show this important and unique point of a plant Amt protein that has been vastly understood as "only" a transporter and indeed may function as a sensor of ammonium as well.

In my opinion, the relevance of this work to the field is extremely significant - the last homolog in the Amt/Mep/Rh superfamily whose structure was missing - but the authors still perform below what they could do/have.

Response: We thank the reviewer for this important suggestion. We agree that the potential sensing function of plant AMT proteins deserved a more thorough analysis in

the original manuscript. In the revised manuscript, we have substantially expanded this part of the study by combining structural analysis of the conserved ammonium-recognition region with additional functional experiments.

First, we re-examined the cryo-EM density maps around the N1/N2-equivalent region across all available *At*AMT1;1 structures. The revised analysis now explicitly shows the corresponding non-protein densities in the different structural states **(Extended Data Fig. 16 and also shown below)**. Where the map quality allowed confident interpretation, these densities were modeled as ordered water molecules. We also compared their occurrence and positions across apo, NH_4^+-bound, methylammonium-bound, low-pH and phosphorylation-mimetic states. This analysis revealed a recurring structural feature in the N1/N2-equivalent region, but did not identify a clear ligand-dependent rearrangement analogous to the Off-to-On transition reported for the bacterial transceptor *Sd*-Amt1.

Second, we experimentally examined the functional contribution of conserved residues surrounding this region. Mutagenesis followed by yeast complementation showed that Q57, N80 and D83 are important for *At*AMT1;1-mediated ammonium transport, whereas C64, S143 and Y155 had little or no apparent effect under the same conditions **(As now Fig. 8g and also shown below)**. These experiments provide direct functional support for the importance of the conserved ammonium-recognition region in the plant protein and complement the structural analysis.

Third, following the reviewer's suggestion, we have substantially expanded the presentation of this topic in the main text (**Lines 21-24 in the Abstract section, Lines 80-88 in the Introduction section, Lines 506-543 in the Results section and Lines 594-611 in the Discussion section)**. Rather than mentioning the potential transceptor-related significance only briefly in the Discussion, we now present the structural comparison with the Off- and On-state *Sd*-Amt1 structures, the conserved N1/N2-equivalent region, the corresponding cryo-EM densities, and the mutational analysis together in a dedicated Results section entitled **"Conserved ammonium-recognition residues are required for *At*AMT1;1 transport activity" (Fig. 8 and Extended Data Fig. 16)**. We have also expanded the Discussion to place these findings in the context

of the bacterial transceptor mechanism.

Importantly, we have kept the interpretation conservative. Although *At*AMT1;1 retains a conserved ammonium-recognition architecture and several residues within this region are functionally important, our current data do not demonstrate a transceptor or ammonium-sensing function analogous to that of *Sd*-Amt1. In particular, *At*AMT1;1 lacks the HAMP–DGC signaling module and we do not observe a clear ligand-dependent conformational transition corresponding to the Off-to-On switching of *Sd*-Amt1. We therefore believe that the revised manuscript provides a substantially more complete structural and functional characterization of this conserved region while avoiding an assignment of a sensing mechanism that is not yet directly supported by the available data.

We appreciate the reviewer's suggestion, which has helped us better highlight the significance of this aspect of the work and clarify both what is conserved and what remains to be established regarding potential sensing or regulatory functions of plant AMT proteins.

Revised Fig. 8:

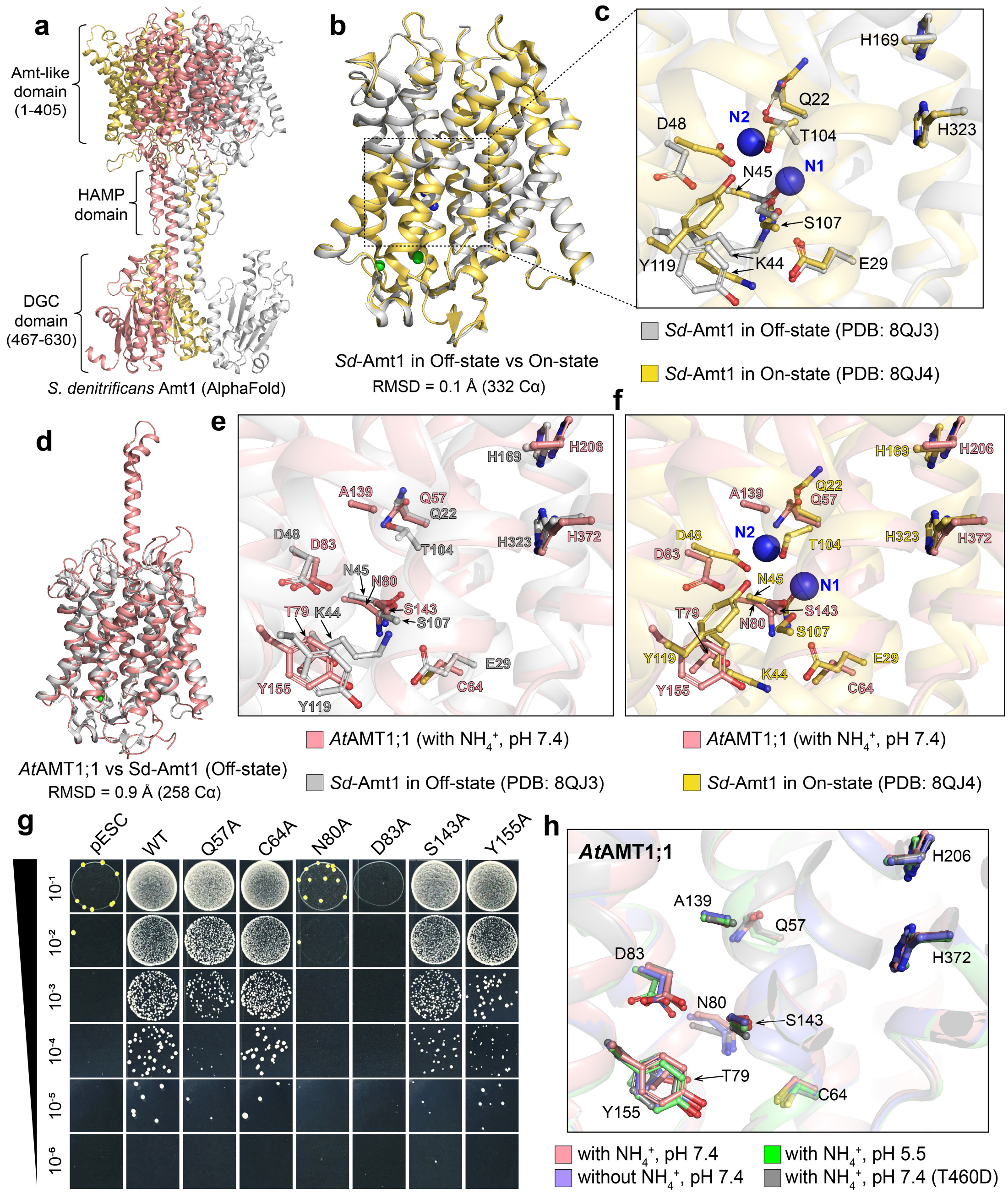
a
Amt-like domain (1-405)
HAMP domain
DGC domain (467-630)
S. denitrificans Amt1 (AlphaFold)
b
Sd-Amt1 in Off-state vs On-state
RMSD = 0.1 Å (332 Cα)
c
H169
Q22
N2
D48
T104
H323
N1
N45
S107
Y119
K44
E29
Sd-Amt1 in Off-state (PDB: 8QJ3)
Sd-Amt1 in On-state (PDB: 8QJ4)
d
AtAMT1;1 vs Sd-Amt1 (Off-state)
RMSD = 0.9 Å (258 Cα)
e
H169
H206
A139
Q57
Q22
D48
D83
T104
H323
H372
N45
N80
T79
K44
S143
S107
E29
Y155
C64
Y119
AtAMT1;1 (with NH4+, pH 7.4)
Sd-Amt1 in Off-state (PDB: 8QJ3)
f
H169
H206
A139
Q22
Q57
N2
D48
D83
T104
H323
H372
N45
N1
S143
T79
N80
S107
Y119
E29
K44
Y155
C64
AtAMT1;1 (with NH4+, pH 7.4)
Sd-Amt1 in On-state (PDB: 8QJ4)
g
pESC
WT
Q57A
C64A
N80A
D83A
S143A
Y155A
10-1
10-2
10-3
10-4
10-5
10-6
h
AtAMT1;1
H206
A139
Q57
H372
D83
N80
S143
T79
Y155
C64
with NH4+, pH 7.4
with NH4+, pH 5.5
without NH4+, pH 7.4
with NH4+, pH 7.4 (T460D)

Revised Extended Data Fig. 16:

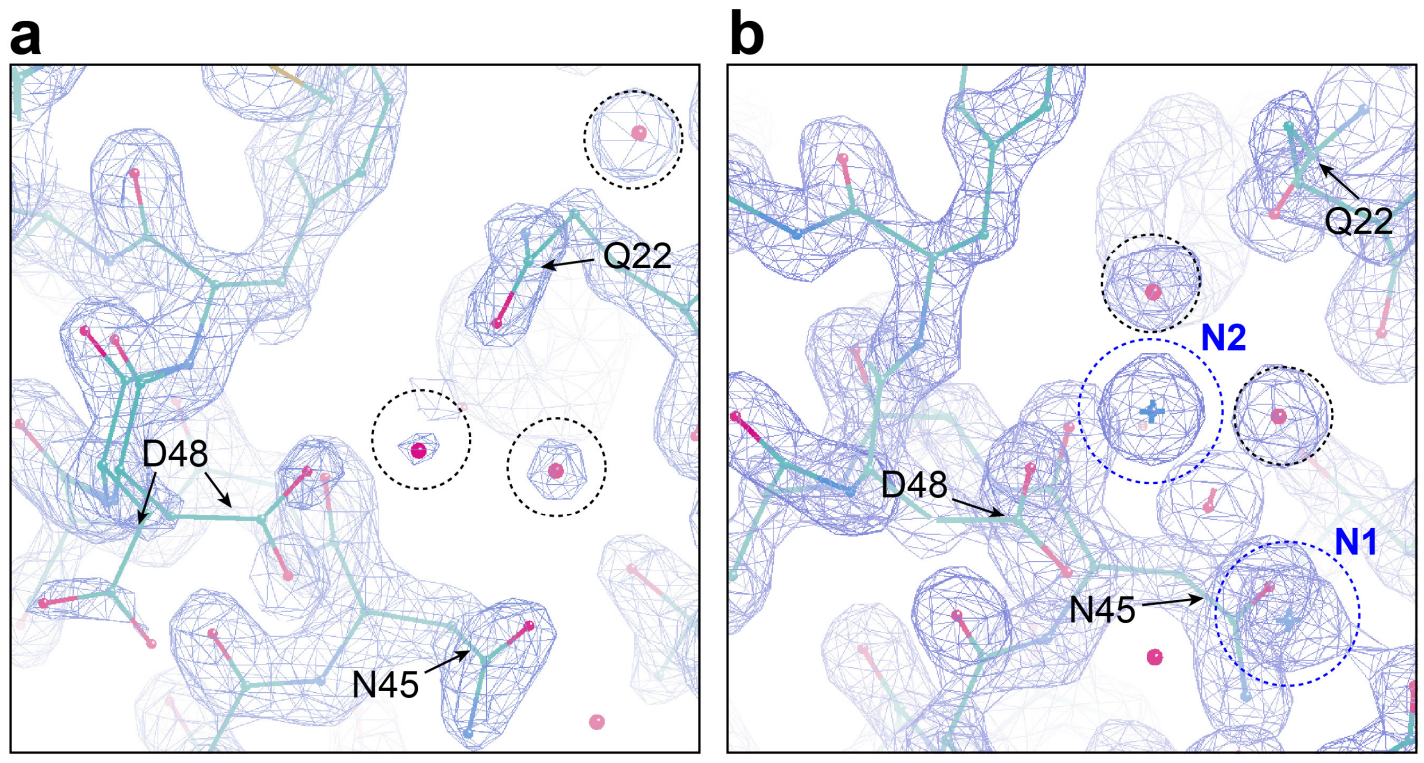

Sd-Amt1 in Off-state (PDB: 8QJ3)
Contour level = 3.0 σ

Sd-Amt1 in On-state (PDB: 8QJ4)
Contour level = 3.0 σ

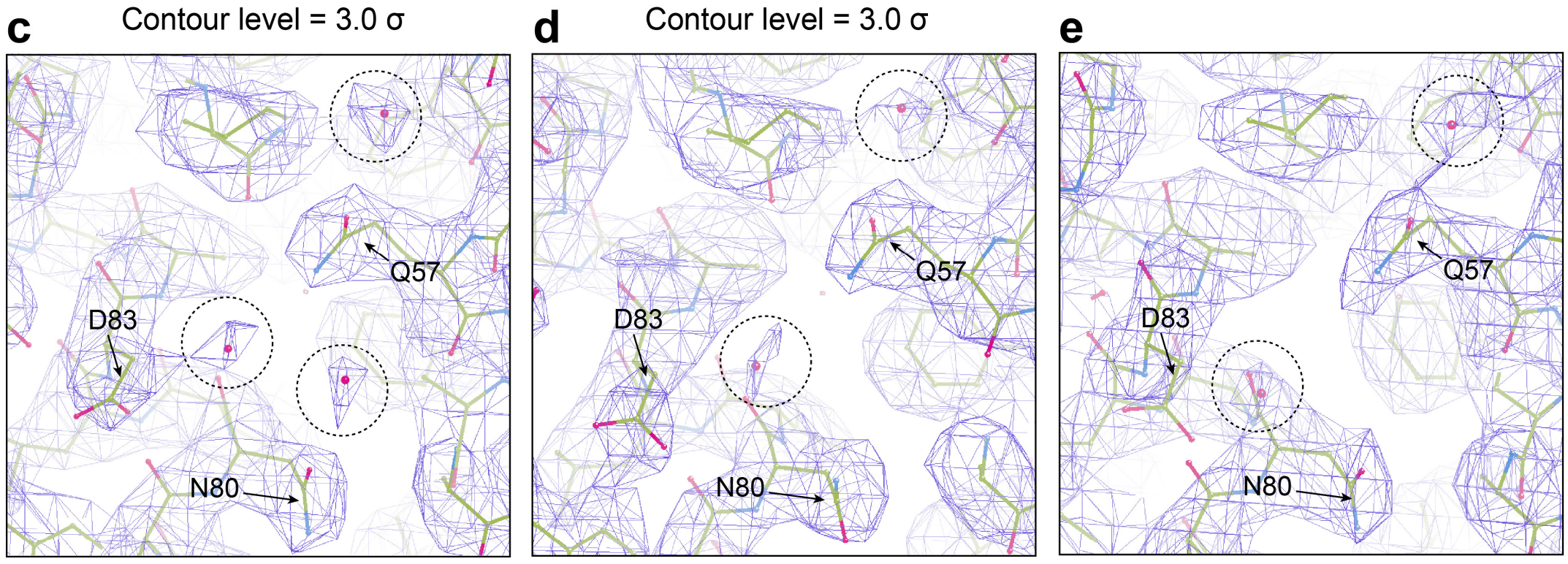

*At*AMT1;1 (without NH_4^+, pH 7.4)
Contour level = 5.0 σ

*At*AMT1;1 (with NH_4^+, pH 7.4)
Contour level = 5.0 σ

*At*AMT1;1 (with MeA, pH 7.4)
Contour level = 5.0 σ

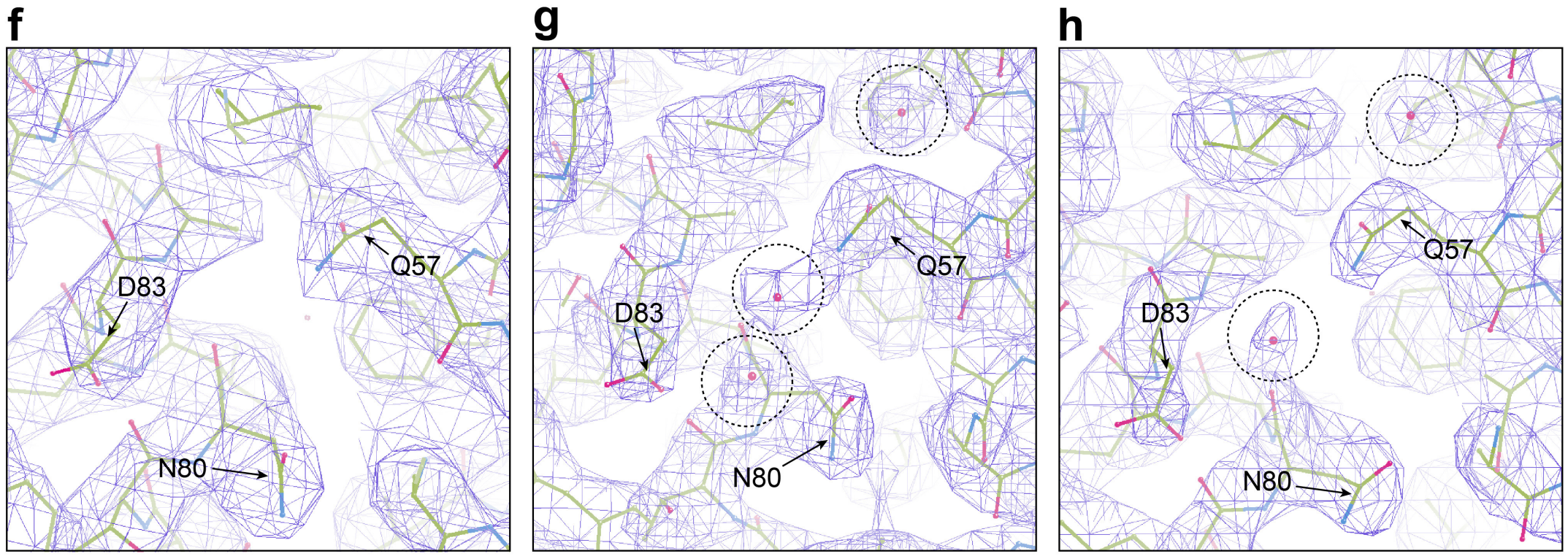

H372F (without NH_4^+, pH 7.4)
Contour level = 5.0 σ

*At*AMT1;1 (with NH_4^+, pH 5.5)
Contour level = 5.0 σ

T460D (with NH_4^+, pH 7.4)
Contour level = 5.0 σ

Reviewer #1 (Remarks to the Author):

The reviewer thanks the authors for the extended work done during revision.

One last remark, easy to verify and to include:

L82: As far as this reviewer understood from reading the two publications related to the bacterial Amts that have N1/N2 motif, they do not seem to act as transporters, thus they may not be best described as transceptors (in line 82: there is no transport-coupled signaling activities). Instead, it is described that ammonium binds at N1/N2.

Change this accordingly throughout the manuscript. However, in line 85 this seems correct since AtAMT1;1 has been described in the literature as a transporter. So, if they would also bind ammonium at N1/N2 they would then be transceptors.

Response: We thank the reviewer for this important clarification. We agree that the bacterial Amt proteins containing the N1/N2 ammonium-binding sites should not be described as transceptors in the absence of demonstrated ammonium transport activity. We have therefore revised the manuscript throughout to remove references to these bacterial proteins as transceptors or as exhibiting transport-coupled signaling. Instead, we now describe them more precisely as ammonium-sensing Amt proteins in which ammonium binding at the N1/N2 sites is associated with conformational changes and downstream signaling.

Accordingly, we also revised related wording in the Abstract, Introduction, Results and Discussion, including replacing terms such as “bacterial transceptor,” “transceptor-associated ammonium-recognition architecture,” and “transport-coupled signaling.” For *At*AMT1;1, which is an established ammonium transporter, we retain the discussion of a potential transceptor function, while clearly stating that our current data do not demonstrate such a function and that additional biochemical and cellular evidence will

be required. We thank the reviewer again for helping us improve the conceptual precision of the manuscript.

ROUND 1 REVIEWER 1 ATTACHMENT:

Reviewer 1:

After major modifications, the manuscript is indeed much better. But since so much was reinterpreted and (re-)written, new issues need to be addressed.

The introduction and discussion of results is now much better, including some of the crucial literature and a correct description of the state of the art regarding the mechanistic understandings of ammonium transport in general, as well as particular aspects concerning AtAMT1;1. On page 3 line 44: I would not say that the mechanism is still unclear. I would say that experimentally proving/disproving the best hypothesis is extremely challenging. Accordingly, the sentence in page 5, lines 80 – 82 should be modified since the structures presented and analyzed do not elucidate the molecular mechanism of ammonium transport. Line 90 should also be altered because "facilitator" is a word best used to describe a passive, down-gradient, channel-like mechanism and this reviewer saw nowhere in the manuscript experiments that could proof this (or any other) mechanism.

While major improvements have been done to the current version, the concept of AtAMT1;1 functioning as a transceptors is now briefly mentioned but remains poorly investigated (see below).
This point should be properly mentioned in the introduction (eventually, after line 77). This will make the description of result and later discussion easier to understand.

Despite improvements the manuscript remains of questionable quality in the experimental data interpretation, presentation and discussion. In particular, the handling and analysis of structural data (major value of the paper) is surprisingly sloppy (see below).

A- STRUCTURAL CONCERNS:

<u>On His372 and the density located below:</u>
If it is NH4+ (as the authors propose in line 237), it would make sense to turn the His ring around and let the NE2 atom point to the NH4+ volume. In that situation, the NE2 atom would be at ca 3.37 Angstrom (A) from the volume which is NOT modelled by the authors in the 22MG-apo-pH7p4 data and 4.19 A from the modelled "NH3" (albeit without proper volume, see figure below). In the 22NK-NH4-pH7p4 data, the "NH3" modelled volume would be at 3.98 A from the NE2 atom of His372 (if turned). Interestingly, this distance is 2.81 A in the 22MV-NH4-pH6 data. This is not adequately described or discussed.

PDB: 22MG
EMD-68466

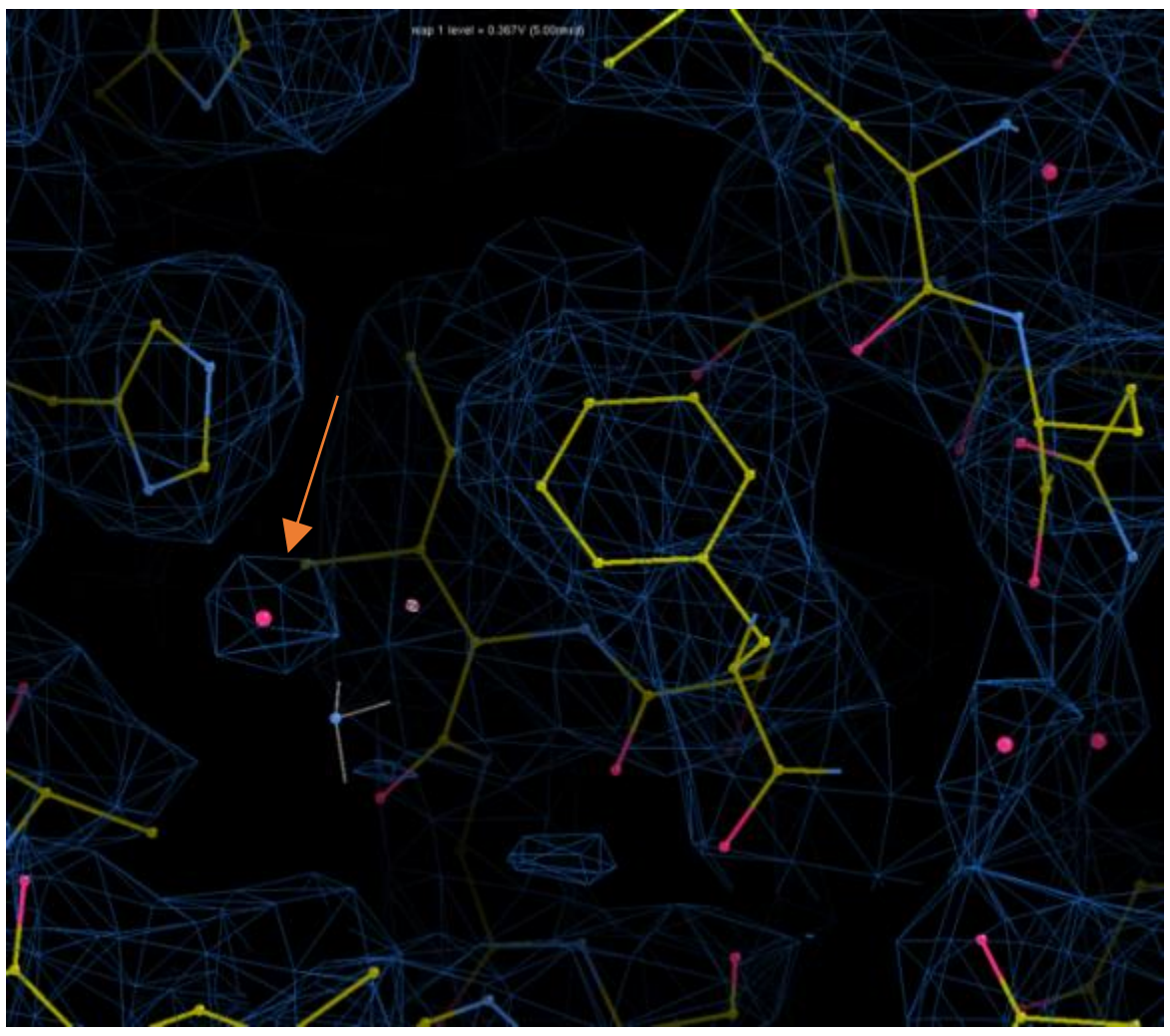

At 5.0 sigma: the modelling of an NH3 molecule (blue) by the authors has no volume at this sigma contour level, while other volumes – much better defined – were left unmodelled by the authors (here, bellow the second His on the His pair motif a “water” (red) was modelled by the reviewer). To the right, two more volumes (non-modelled by the authors) were modelled by the reviewer, again as “water”. These are at the N1/N2 site – see below for a better view of the site.

The assignment of MeA in the PDB: 22OY is, in the opinion of this reviewer, not adequate and potential damaging: a) the volume is not very good for the assignment (see figure below) and b) there is no alternative experiment backing up the identification of MeA in this position:

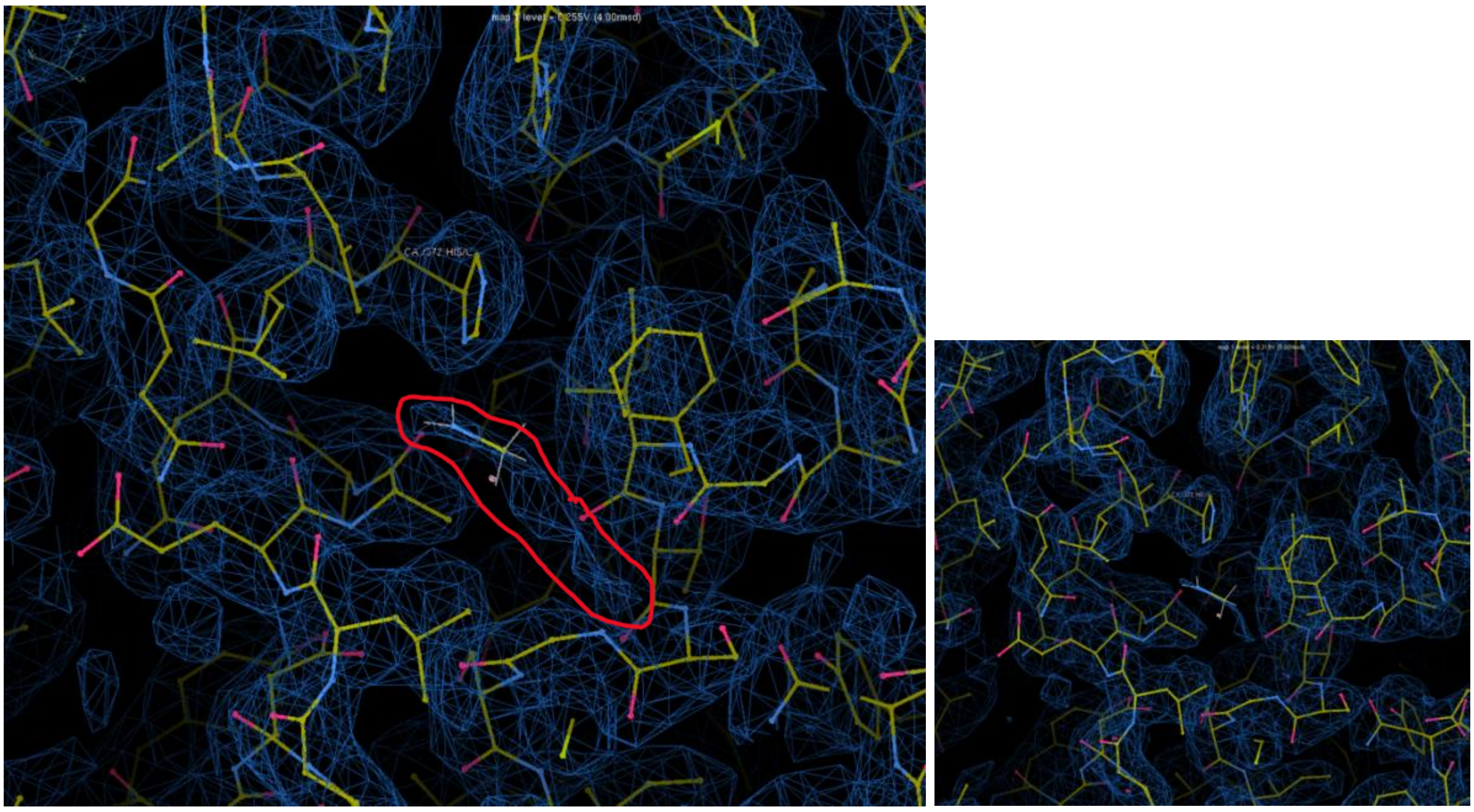

Above, left: MeA was modelled (arrow). Figure is shown at 4 sigma contour. Not only there is more volume highlighted in red, but the volume density is weak and thin, potentially reflecting a weakly associated detergent molecule in this exit vestibule region, as it has been seen in other Amt structures). Above, rigth: same location now at 5.o sigma confirming the very weak and thin volume.

On N1/N2 site and unassigned volumes there:

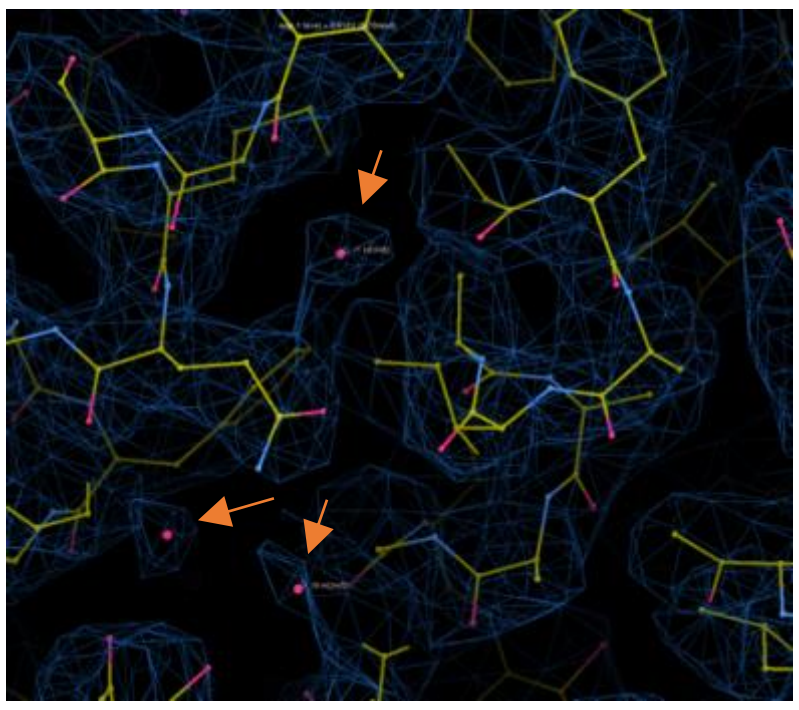

Chain A

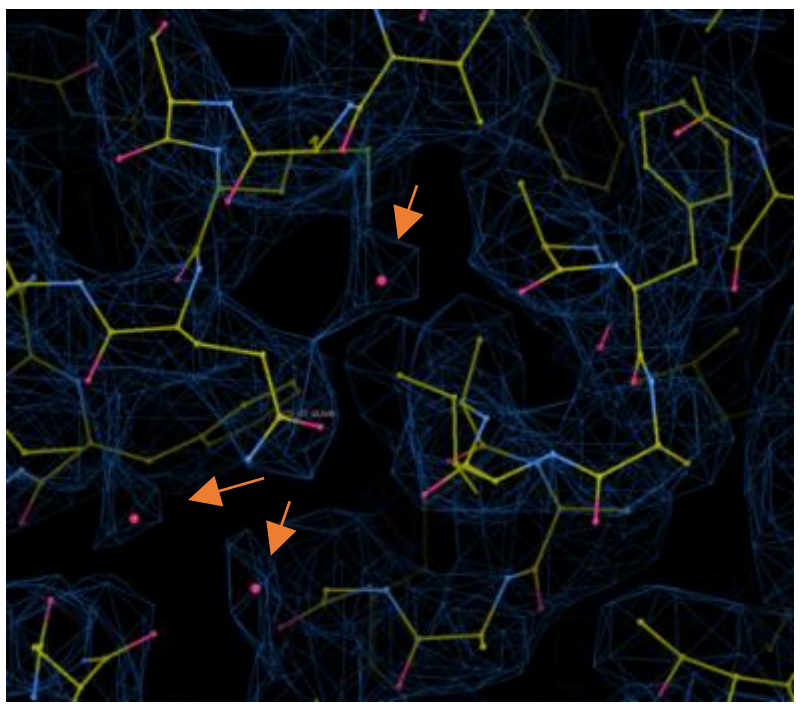

chain B

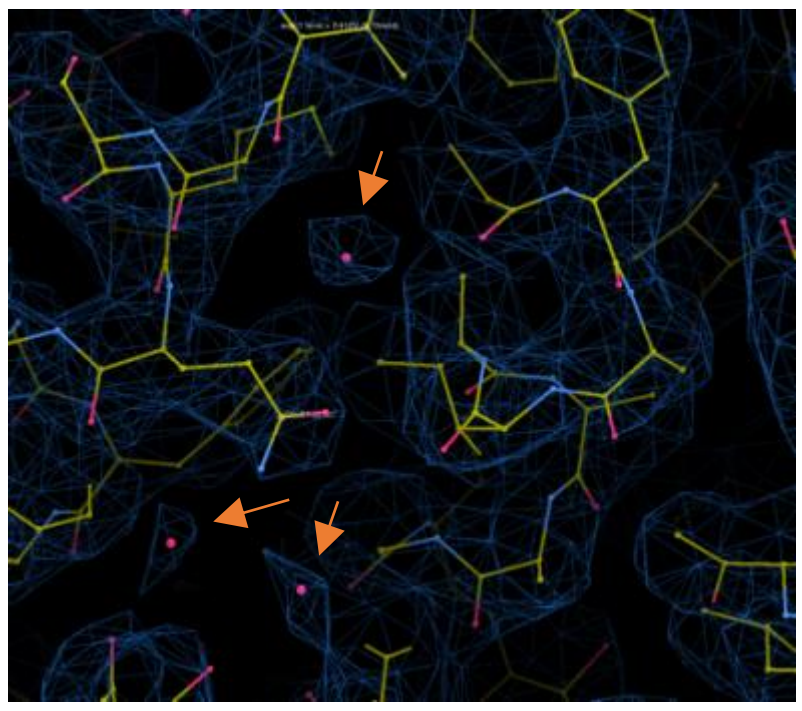

chain C

In all these 3 pictures above, the N1/N2 site has three unmodelled densities. Arrows show where the reviewer modelled “water” (orange arrow). The sigma contour is 5.7 for all.

The authors have made no effort to model any non-protein volume, except at the recruitment site (an ammonium although this is pure imagination given the experimental volumes there) and below the second twin His (an ammonia – improperly modelled as seen above).

PDB: 22NK
EMD-68512

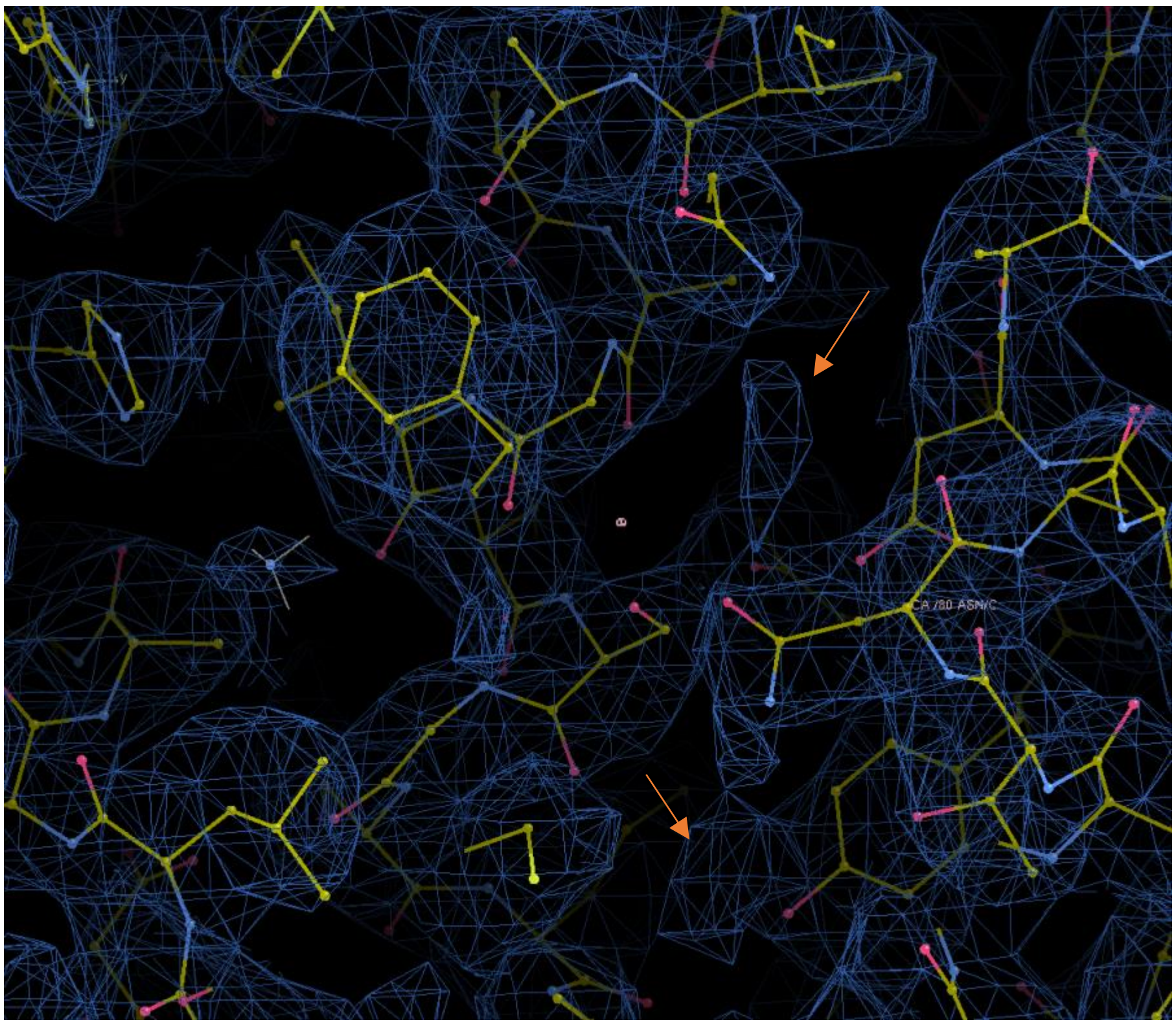

At 3.9 sigma, some unassigned volumes are shown by orange arrows.

Comparison of PDB 22NK (above) to the PDB 22MG (below, in identical position): green arrows show the “waters” modelled by the reviewer below in unassigned volumes:

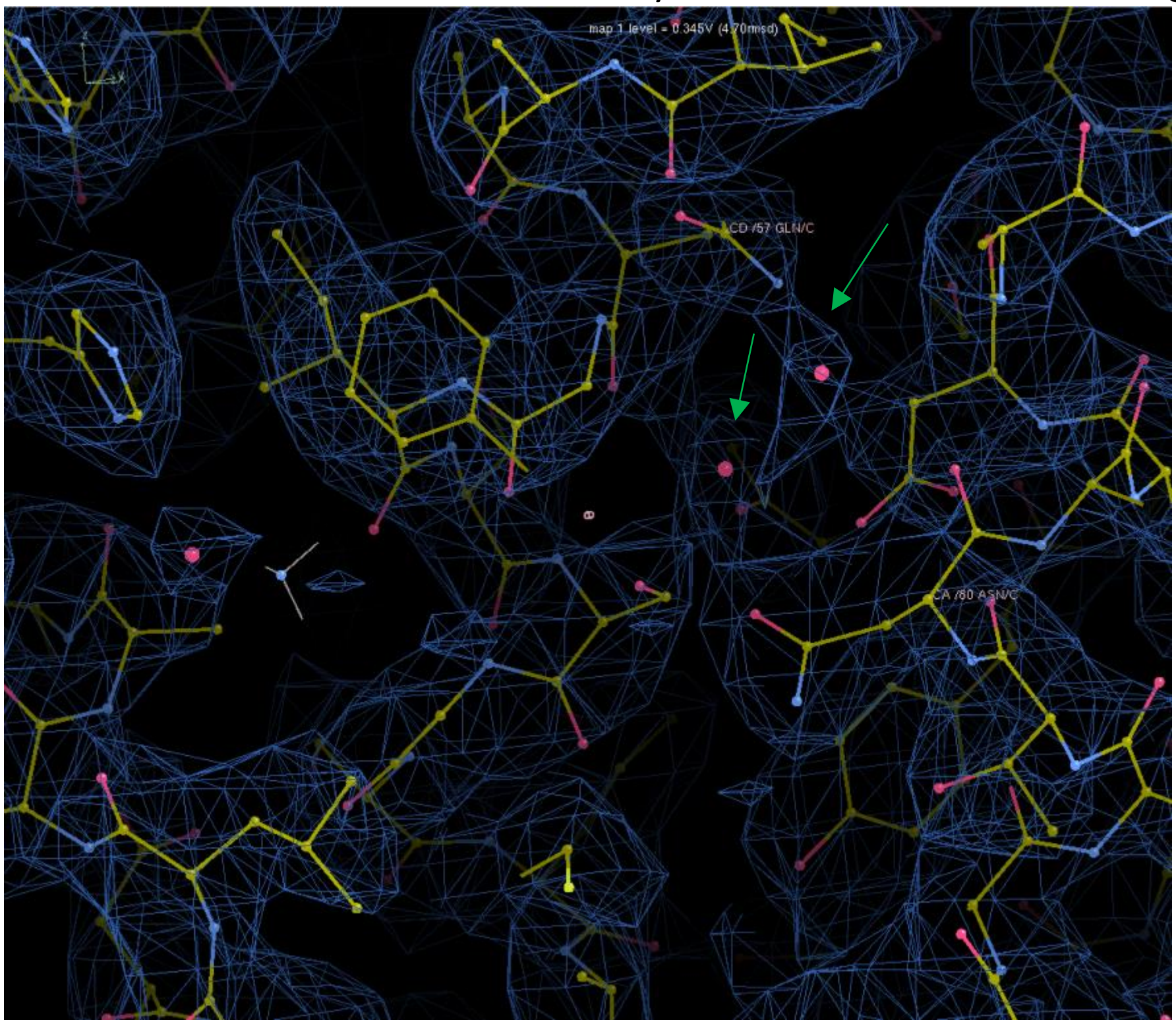

Clearly interesting to compare situations, conformations of again, unassigned volumes near this region of a potential ammonium binding site.

Another example of some densities left unassigned between PDB 22MG (top), 22NK (middle) and 22MV (bottom), around Gln57 (near N1/N2 site) with potential functional significance. Quality of the maps vary but at least two significant volumes can be seen (arrow):

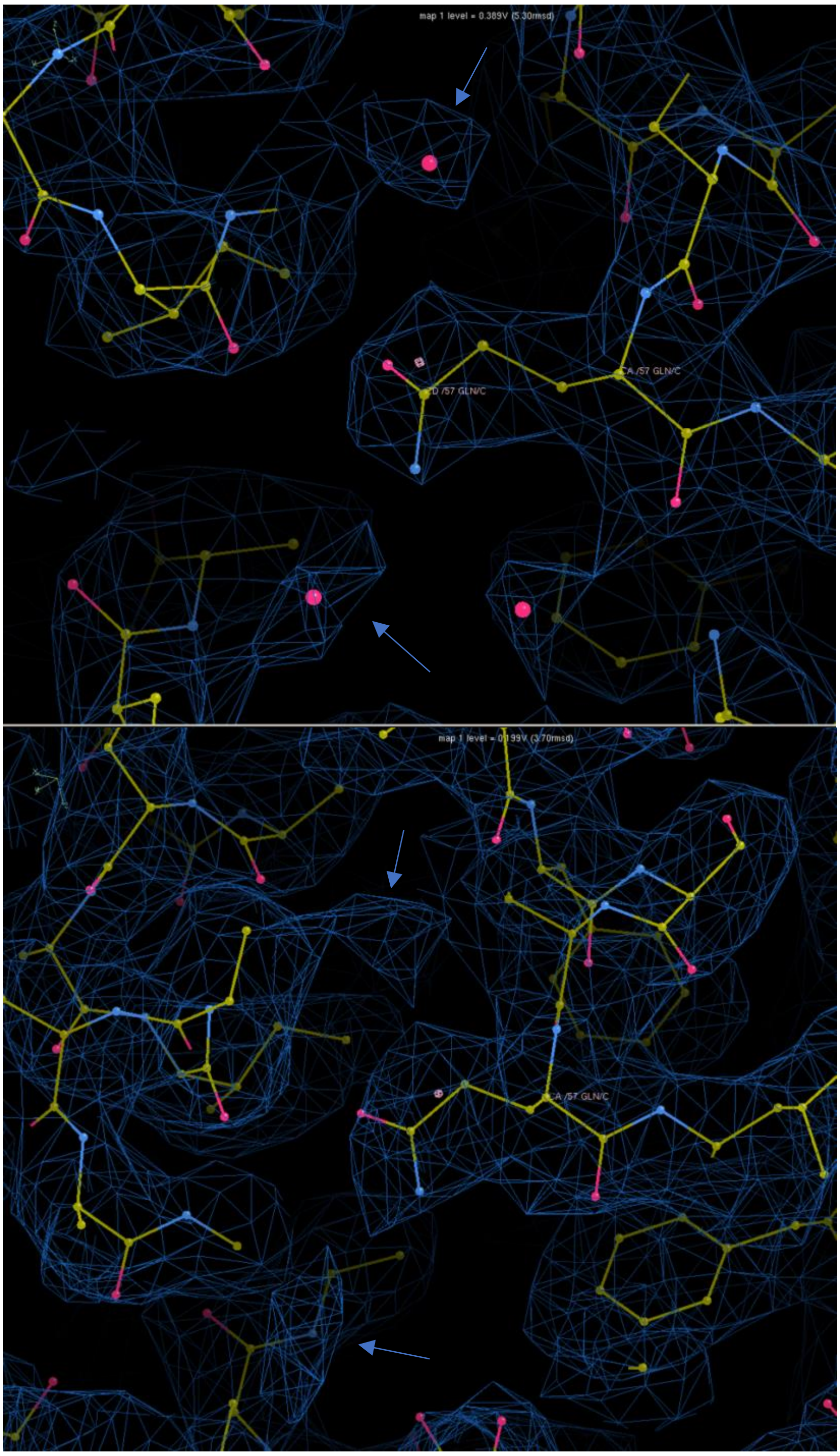
map 1 level = 0.389V (5.30rmsd)
/57 GLN/C
CA /57 GLN/C
map 1 level = 0.199V (3.70rmsd)
CA /57 GLN/C

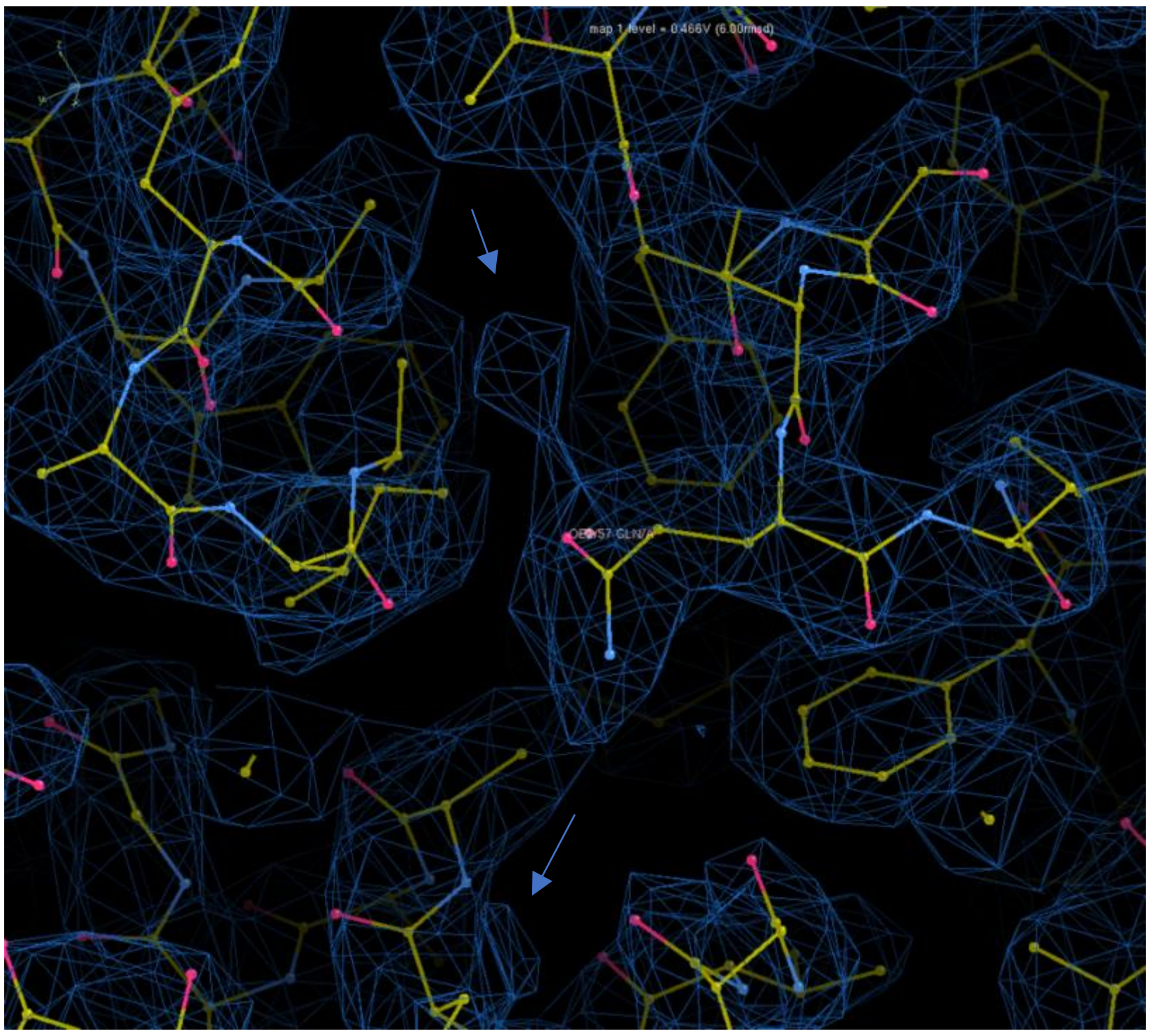

Only one chain is shown, but similar volumes are seen in all chains.

To this reviewer, AtAmt1;1 residues: Q57, C64, N80, D83, A 84, G1540 and S143 should be thoroughly analyzed as very likely N1/N2 ammonium ligands based on recent literature dealing with sequence analysis. Here, the structures show clear volumes in positions that have previously been assigned to water or ammonium in ammonium receptors. They are however, left unassigned and unanalyzed by the authors, which stands as a lost opportunity. The authors claim that they do not show conformational changes, but this is not the decisive point to disclaim this important point. The authors should analyze and describe the site properly. Further investigations may address the transceptors question further, but structurally, the data description is essentially absent and this is not acceptable.

<u>General structural issues:</u>
This reviewer notes a worrisome feature in all structures; Besides the fact that there was absolutely no attempt to model any volume resembling water as water, all monomers in the trimeric models are rigid, appearing in the same exact copied position in the trimer, despite visible changes occur in the volumes for the different monomers in the structure. Were the monomers copied and not further modelled? The authors describe using Phoenix in the materials and methods, but it seems that refinement was not done?
How can the observations of static monomers be explained?

Why is the **PBD: 22OY** (MeA, pH 7.4) a dimer? Why was the trimer not modelled? There is volume for it – as expected since all Amts are obligatory trimers.
The structural work is sloppy, not well done and needs improvements. Please also take care of chain annotations in the PDB files.

No PDB or EMD files of the new lower pH dataset were provided for consideration.

On the recruitment site volumes:

The figure showing NH4+/H2O or MeA at the recruitment vestibule is misleading (Ext-Fig 11). The volume is more complex and does not fit such molecules. The sigma level of the maps is not shown in the figures or legends but (see below), even at 6 sigma, the volumes (surrounded in red) do not match the model.

The figures below show the PDB: 22MG (top) and PDB:22OY (bottom), at different sigma contour levels (written on top of the figure as sigma 4 on the left, 5 in the middle and 6 on the rigth for the top case of 22MG):

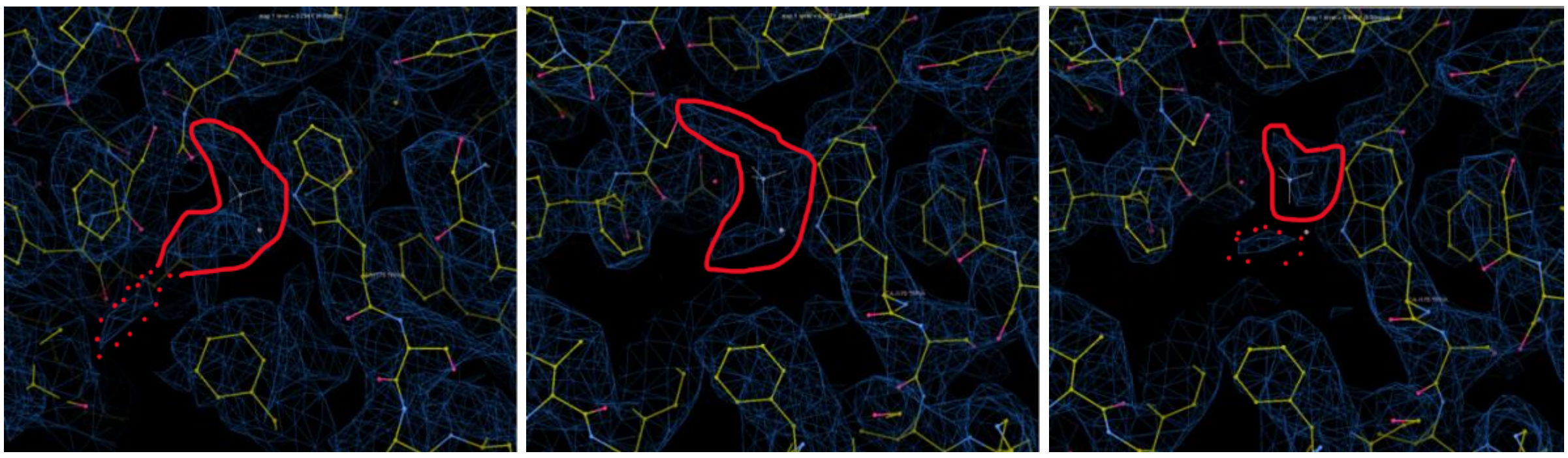

In both (top and down) cases, the view is from outside looking into the recruitment side with W157 located on the right side of the red marks.

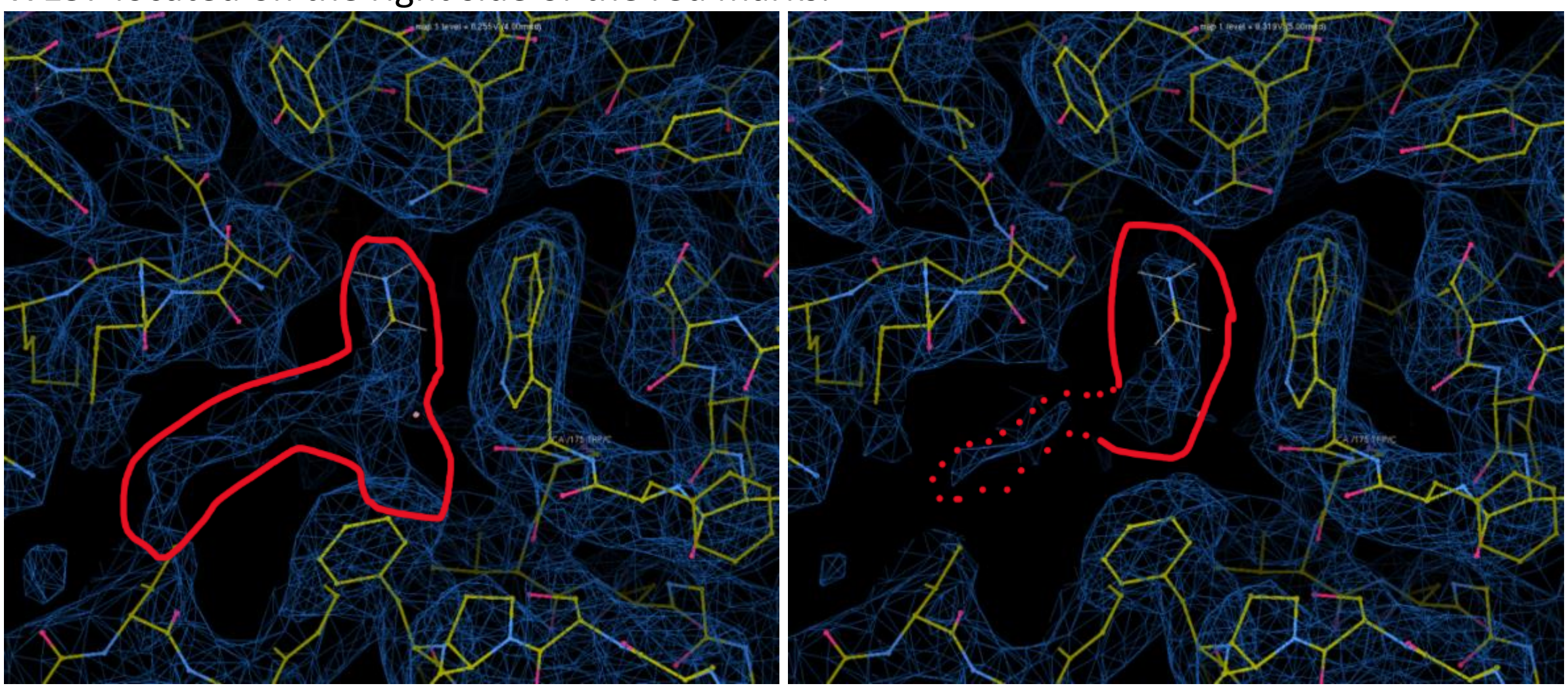

Clearly, the volumes do not correspond neither to MeA nor something as simple and round as H2O, NH3 or NH4+ and this cannot be written in the text.

The new low pH dataset:

Given that the two Phe have their aromatic rings staked, it is not straightforwardly understandable what the authors mean by “ the Phe-gate was slightly narrowed”? What does the site look like if the low pH structure is superimposed with any other? Such situation has not been described before and a detailed view showing that on Fig 5 would be very adequate.

B - MASS PHOTOMETRY DATA:

Line 175 and ext-Fig 7b,c: the MP interpretation data remains debatable: This reviewer finds it unclear why the authors assign the large MW peak to a trimer instead of a hexamer – this MP data should be taken together with size exclusion chromatography results of pure protein in detergent micelles). Moreover, micelles (from detergent-only or from protein in detergent suspensions) will reveal background contributions that depend on the concentration of detergent used and will produce symmetric profiles in the negative x axis scale, while the same is not true for protein contributions. Can the authors verify this?

C – N-TERM CYS:

In the experiments done using (i) DDT / (ii) H2O2 to (i) reduce the disulfide bond and break the cystine and (ii) re-oxidize the thiols and regenerate the cystine, H2O2 is a very poor choice as it is too strong oxidizing. DTT (not 50 or 100 mM but less, 5 – 20 mM can be used at neutral pH) but DTT needs to be removed (desalting or dialysis) before adding (not 30 mM, but 0.1 -1 mM) H2O2. If it is not removed, DTT will mostly react with the added H2O2 and rapidly deactivate it. Also, H2O2 near lipids and aminoacids is well known to be extremely destructive. Therefore, instead of this assay which can produce irreproducible and non linear results, a glutathione redox buffer system or the use of diamide instead of H2O2 would have been a much better choice.

D - OTHER ISSUES:

Please replace “substrate” with “cargo” as this is the correct description for the molecule/ion that a transporter recognizes and mediates transport of, in contrast to a substrate molecule that is catalyzed into a product by an enzyme.

Line 302: When mentioning computation data on the hypothesis of protonation/deprotonation of the twinHis motif during transport, it is a must to cite Ullmann et al, J Phys Chem B, 2012, 116:9690 or similar publications, which focused precisely on this issue.

Line 394: replace “is” by “are”

Line 455: why plural? Which W do the authors refer to here with the plural?

Line 456: replace “gate” by “vestibule”.

459 an 496: “continuous .. conduction pathway”? The two Phe interrupt the pore opening, it is not exactly continuous – or explain better.

Line 484: Wasn´t there a reference from 2011 (ref 48) showing that N-terminal Cys affect oligomer stability in LeAMT1;1? Rephrase accordingly this paragraph.

Fig 2a, and b are redundant.
